# Global Burden, Risk Factors, and Trends of Esophageal Cancer: An Analysis of Cancer Registries from 48 Countries

**DOI:** 10.3390/cancers13010141

**Published:** 2021-01-05

**Authors:** Junjie Huang, Anastasios Koulaouzidis, Wojciech Marlicz, Veeleah Lok, Cedric Chu, Chun Ho Ngai, Lin Zhang, Ping Chen, Shanjuan Wang, Jinqiu Yuan, Xiang-Qian Lao, Shelly L.A. Tse, Wanghong Xu, Zhi-Jie Zheng, Shao-Hua Xie, Martin C.S. Wong

**Affiliations:** 1 The Jockey Club School of Public Health and Primary Care, Faculty of Medicine, Chinese University of Hong Kong, Hong Kong SAR 999077, China; junjie_huang@link.cuhk.edu.hk (J.H.); cedricchu@cuhk.edu.hk (C.C.); alfonsengai@cuhk.edu.hk (C.H.N.); xqlao@cuhk.edu.hk (X.-Q.L.); shelly@cuhk.edu.hk (S.L.A.T.); 2 Endoscopy Unit, The Royal Infirmary of Edinburgh, Edinburgh, Scotland EH4 2XU, UK; Tassos.Koulaouzidis@luht.scot.nhs.uk; 3 Department of Gastroenterology, Pomeranian Medical University in Szczecin, 71-252 Szczecin, Poland; wojciech.marlicz@pum.edu.pl; 4 The Centre for Digestive Diseases Endoklinika, 70-535 Szczecin, Poland; 5 Department of Global Public Health, Karolinska Institute, Karolinska University Hospital, 171 77 Stockholm, Sweden; veeleah.lok@stud.ki.se; 6 Centre of Cancer Research, Victorian Comprehensive Cancer Centre, Melbourne, VIC 3000, Australia; tony1982110@gmail.com; 7 Melbourne School of Population and Global Health, The University of Melbourne, Melbourne, VIC 3053, Australia; 8 School of Population Medicine and Public Health, The Chinese Academy of Medical Sciences and The Peking Union Medical College, Beijing 100005, China; 9 Department of Gastroenterology, Ruijing Hospital North, School of Medicine, Shanghai Jiaotong University, Shanghai 200240, China; cpb1001@rjhn.com.cn; 10 Department of Gastroenterology, Jiading District Hospital, Shanghai 201800, China; wangshanjuan@jdhospital.com; 11 Clinical Research Centre, Scientific Research Centre, The Seventh Affiliated Hospital, Sun Yat-sen University, Shenzhen 518107, China; yuanjq5@mail.sysu.edu.cn; 12 School of Public Health, Fudan University, Shanghai 200433, China; wanghong.xu@fudan.edu.cn; 13 Department of Global Health, School of Public Health, Peking University, Beijing 100191, China; zhengzj@bjmu.edu.cn; 14 Upper Gastrointestinal Surgery, Department of Molecular Medicine and Surgery, Karolinska Institute, Karolinska University Hospital, 171 77 Stockholm, Sweden; shaohua.xie@ki.se

**Keywords:** esophageal cancer, incidence, mortality, histological subtypes, risk factors

## Abstract

**Simple Summary:**

Esophageal cancer is the seventh most common cancer globally. Preventive measures and clinical management differ based on histologic subtype. However, information has been lacking on its most recent patterns according to histological subtype, associated risk factors, and epidemiological trends on a global scale. This study is a global analysis of the incidence/mortality trends of esophageal cancer in more than 48 countries/regions based on high quality population-based registries. We conclude that adenocarcinoma has already surpassed squamous cell carcinoma as the most frequent type of esophageal cancer in some western countries and is expected to increase in other countries. It is important to closely monitor and slow down the growing rates of obesity and metabolic syndrome, which are the important risk factors for adenocarcinoma. With the development of more advanced and less invasive technology, population-based targeted screening endoscopy would be recommended for high-risk individuals.

**Abstract:**

This study aimed to examine the global burden, risk factors, and trends of esophageal cancer based on age, sex, and histological subtype. The data were retrieved from cancer registries database from 48 countries in the period 1980–2017. Temporal patterns of incidence and mortality were evaluated by average annual percent change (AAPC) using joinpoint regression. Associations with risk factors were examined by linear regression. The highest incidence of esophageal cancer was observed in Eastern Asia. The highest incidence of adenocarcinoma (AC) was found in the Netherlands, the United Kingdom, and Ireland. A higher AC/squamous cell carcinoma (SCC) incidence ratio was associated with a higher prevalence of obesity and elevated cholesterol. We observed an incidence increase (including AC and SCC) in some countries, with the Czech Republic (female: AAPC 4.66), Spain (female: 3.41), Norway (male: 3.10), Japan (female: 2.18), Thailand (male: 2.17), the Netherlands (male: 2.11; female: 1.88), and Canada (male: 1.51) showing the most significant increase. Countries with increasing mortality included Thailand (male: 5.24), Austria (female: 3.67), Latvia (male: 2.33), and Portugal (male: 1.12). Although the incidence of esophageal cancer showed an overall decreasing trend, an increasing trend was observed in some countries with high AC/SCC incidence ratios. More preventive measures are needed for these countries.

## 1. Introduction

Esophageal cancer is the seventh most common malignancy globally, with more than 500,000 new cases diagnosed annually [1]. It is the sixth leading cause of cancer mortality, accounting for over 500,000 cancer deaths each year [1]. Patients with esophageal cancer have an overall 5-year survival below 20% [2], although the ChemoRadiotherapy for Esophageal cancer followed by Surgery Study (CROSS) and docetaxel-based triplet (fluorouracil plus leucovorin, oxaliplatin, and docetaxel (FLOT)) improved the rate to 47% and 45%, respectively [3,4]. Worldwide, it remains an important public health and clinical issue due to its aggressive nature and low survival rate. Most esophageal cancer cases are diagnosed in less developed countries [5]. However, its global burden varies greatly, ranging from the second most frequent malignancy in some regions to one of the least frequent malignancies in other regions [6].

The two main histologic subtypes of esophageal cancer are adenocarcinoma (AC) and squamous cell carcinoma (SCC) [7]. Despite SCC accounting for most of all esophageal cancer cases, recent years have witnessed an increasing trend of AC in western populations [8]. The risk factors for SCC include male gender, family history, smoking, alcohol drinking, certain dietary factors, and possibly poor oral hygiene, whereas gastro-esophageal reflux disease (GERD) and obesity are the two main established risk factors for AC, and Barrett’s esophagus is a well-known premalignant condition [9,10,11,12]. As a substantial proportion of these risk factors are modifiable, there is the potential for public health interventions to prevent cancer development.

Monitoring the epidemiological trend of esophageal cancer is crucial, as it can inform the formulation of effective public health and clinical strategies as well as provide etiological clues. Owing to its high disparity in epidemiology across different populations, a comprehensive evaluation of its global burden and recent trends using high quality cancer registry data could benefit resource planning and allocation. Determining the updated incidence of esophageal subtypes and their associated preventable risk factors is also important, as the preventive measures and clinical management would be different for AC and SCC. Nevertheless, few studies reported the disease burden by histological subtypes, associated risk factors, and recent epidemiological trends of esophageal cancer on a global scale. The previous analysis estimated the incidence by histological subtypes without trend analysis [13,14,15], or evaluated the epidemiological trend using cancer registry data up to 2007 only [5,16,17]. The Global Burden of Disease (GBD) Study [18] evaluated the pattern of esophageal disease based on modeling. Furthermore, none of these studies investigated the country-level association between the AC/SCC incidence ratio and lifestyle and metabolic risk factors for esophageal cancer. Therefore, there exists a knowledge gap on the most updated global patterns of esophageal cancer by histological subtypes and associated risk factors. The objective of this study is to evaluate the global profile, associated risk factors, and recent epidemiological trends of esophageal cancer by region, histological subtype, gender, and age using cancer registries data for 48 countries.

## 2. Methods

### 2.1. Data Source

We used a methodology adapted from our previous studies on the epidemiological trends of esophageal cancer [5], prostate cancer [19], and colorectal cancer [20] by the same team. The data on incidence/mortality of esophageal cancer from national and global registries between 1980 and 2017 were retrieved (Table S1). Specifically, Cancer Incidence in Five Continents (CI5) volumes I–XI contains data on cancer incidence from cancer registries of high quality in a substantial proportion of the countries/regions around the world [21]. Data on the population size, numbers of new cancer cases, crude incidence rate, adjusted incidence rates, and standard errors reported by age, gender, cancer type, country, region, and calendar year were available in CI5. The Surveillance, Epidemiology, and End Results (SEER) *9* program were searched to obtain the most recent cancer figures for the United States (US) [22]. The Nordic Cancer Registries *(NORDCAN)* were searched for the most recent data for the Northern European countries, including Finland, Denmark, Norway, Sweden, Iceland, Greenland, and the Faroe Islands [23,24]. Furthermore, we searched the World Health Organization (WHO) Mortality database for the most recent mortality figures for other countries/regions [25]. The figures in the WHO Mortality database were generated from national civil causes of death registries in different countries/regions. The WHO Mortality collected different causes of medically diagnosed deaths from national level registry databases from different countries/regions yearly. Only data with a quality level at medium or above were used in the WHO Mortality database [26]. The burden of total esophageal cancer and the two major histological types, adenocarcinoma (AC) and squamous cell carcinoma (SCC), in 2018 was estimated from GLOBOLCAN [27], CI5 [21], and previous studies [5,14]. The prevalence of lifestyle (smoking, alcohol consumption, and physical inactivity) and metabolic (obesity, diabetes, and high cholesterol) risk factors were extracted from the Global Health Observatory (GHO) data (Table S2). The International Classification of Diseases and Related Health Problem, 10th Revision (ICD-10) code C15 was used to identify “malignant neoplasm of the esophagus” in the study [28]. The current study calculated age-standardized rates (ASRs) for incidence and mortality of esophageal cancer using the Segi–Doll world standard population [29].

### 2.2. Statistical Analysis

The temporal patterns of incidence and mortality of esophageal cancer in the past ten years were assessed for each country/region using joinpoint regression analysis [30]. The results were presented as the average annual percent changes (AAPCs) in the ASR of incidence/mortality with 95% confidence intervals (CIs) [30]. The joinpoint regression analysis allows a more accurate interpretation of trends in cancer incidence and mortality using AAPCs and also to determine if the trend is statistically significant [31]. A logarithmic transformation of the incidence and mortality figures for each county/region was conducted before calculating the standard errors by binomial approximation. Weights equivalent to each segment’s length were apportioned for the most recent ten-year period [32]. Countries/regions/population groups with “zero” or “missing” values in incidence/mortality figures for any year during the most recent decade were excluded from the regression analysis. A maximum of three joinpoints was adopted as the parameter of trend analysis in the current study. The AAPC was estimated as the mean of annual percent changes (APCs) using geometric weighting in populations of different age groups (≥50 years and <50 years), genders (males and females), and countries/regions. The association between AC/SCC incidence ratio for esophageal cancer and prevalence of lifestyle and metabolic risk factors was examined using multivariable linear regression analysis. Missing data in the regression on risk association were handled by complete-case analysis using listwise deletion. A two-sided *p* value less than 0.05 was considered statistically significant. The analysis was performed using Joinpoint Regression Program (Version 4.8.0.1; Surveillance Research Program, National Cancer Institute of US) and Stata (Release 14; College Station, TX: StataCorp LP).

## 3. Results

### 3.1. Global Burden

#### 3.1.1. Incidence

**Figure 1:**
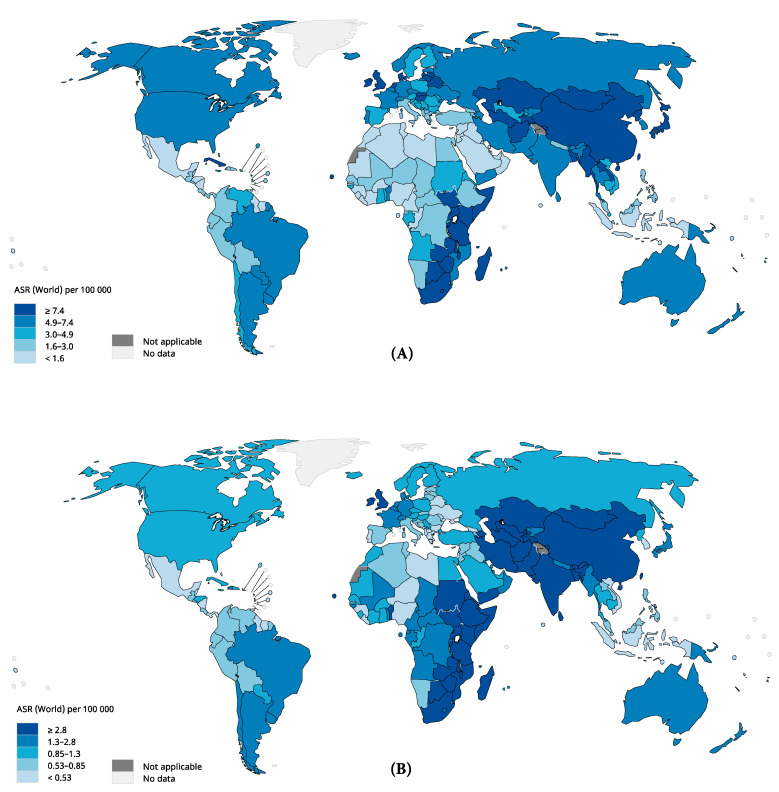
The global estimated incidence of esophageal cancer in 2018, both sexes, all ages: (**A**) male incidence; (**B**) female incidence. ASR, age-standardized rate. Data source: GLOBOCAN 2018, IARC (http://gco.iarc.fr/today), World Health Organization.

In 2018, a total of 572,034 new cases of esophageal cancer (all types) were recorded, and the ASR of incidence was 6.3 per 100,000 persons, showing about thirteen-fold variation globally (Table S3, Figure 1). The highest rates were observed in Eastern Asia (ASR 12.2), Eastern Africa (ASR 8.3), Southern Africa (ASR 7.4), and Northern Europe (ASR 5.5), whilst the lowest rates were found in Central America (ASR 0.96), Western Africa (ASR 1.2), Northern Africa (ASR 1.5) and Western Asia (ASR 1.7). This geographical difference was more pronounced among males than females. The incidence rates were higher in males than in females in all regions.

#### 3.1.2. Mortality

**Figure 2:**
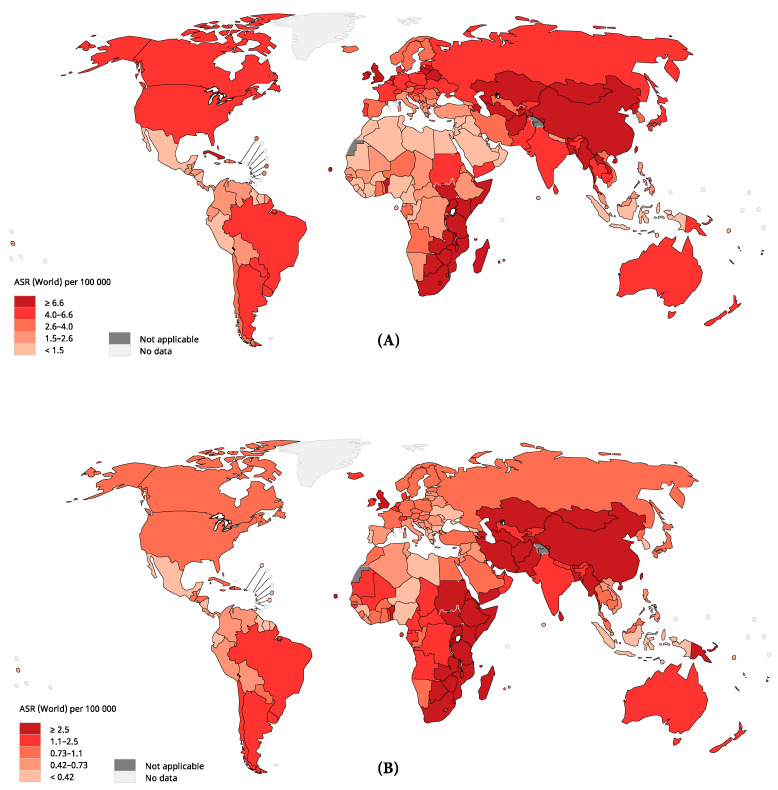
The global estimated mortality of esophageal cancer in 2018, both sexes, all ages: (**A**) male mortality; (**B**) female mortality. ASR, age-standardized rate. Data source: GLOBOCAN 2018, IARC (http://gco.iarc.fr/today), World Health Organization.

Globally, a total of 508,585 related deaths occurred in 2018. The ASR of mortality was 5.5 per 100,000 persons and varied by twelve-fold (Figure 2). The highest mortality rates were found in Eastern Asia (ASR 10.7), Eastern Africa (ASR 8.2), Southern Africa (ASR 7.2), and Northern Europe (ASR 4.3), whilst the lowest rates were found in Central America (ASR 0.92), Western Africa (ASR 1.2), Northern Africa (ASR 1.5) and Western Asia (ASR 1.5). The geographical variation in mortality rates was much more marked in males than in females, whilst the mortality rates were higher in males than in females in all regions.

#### 3.1.3. Incidence by Histologic Subtypes

Table S4 shows the gender-specific estimated number of new cases, ASR, and the AC/SCC incidence ratio by country in 2018. Among males, highest ASRs of AC were observed in the Netherlands (ASR 7.7, ratio 2.5), United Kingdom (ASR 7.5, ratio 2.9), Ireland (ASR 4.9, ratio 1.9), Denmark (ASR 4.6, ratio 1.3), New Zealand (ASR 4.2, ratio 2.7), and Iceland (ASR 4.1, ratio 1.4), whilst the highest ASRs of SCC were observed in Malawi (ASR 21.0, ratio 0.053), Mongolia (ASR 20.9, ratio 0.050), Kenya (ASR 20.3, ratio 0.057), China (ASR 18.8, ratio 0.046), Uganda (ASR 17.0, ratio 0.056), and Bangladesh (ASR 16.7, ratio 0.121). Among females, the ASRs of AC were highest in the United Kingdom (ASR 1.4, ratio 0.70), Zimbabwe (ASR 1.4, ratio 0.13), the Netherlands (ASR 1.2, ratio 0.80), and Ireland (ASR 1.0, ratio 0.46), whilst the highest ASRs of SCC were observed in Malawi (ASR 15.5, ratio 0.045), Mongolia (ASR 15.1, ratio 0.058), Kenya (ASR 15.1, ratio 0.049), Zimbabwe (ASR 10.7, ratio 0.13), and Bangladesh (ASR 10.0, ratio 0.067).

### 3.2. Associations between Risk Factors and AC/SCC Incidence Ratio 

**Figure 3:**
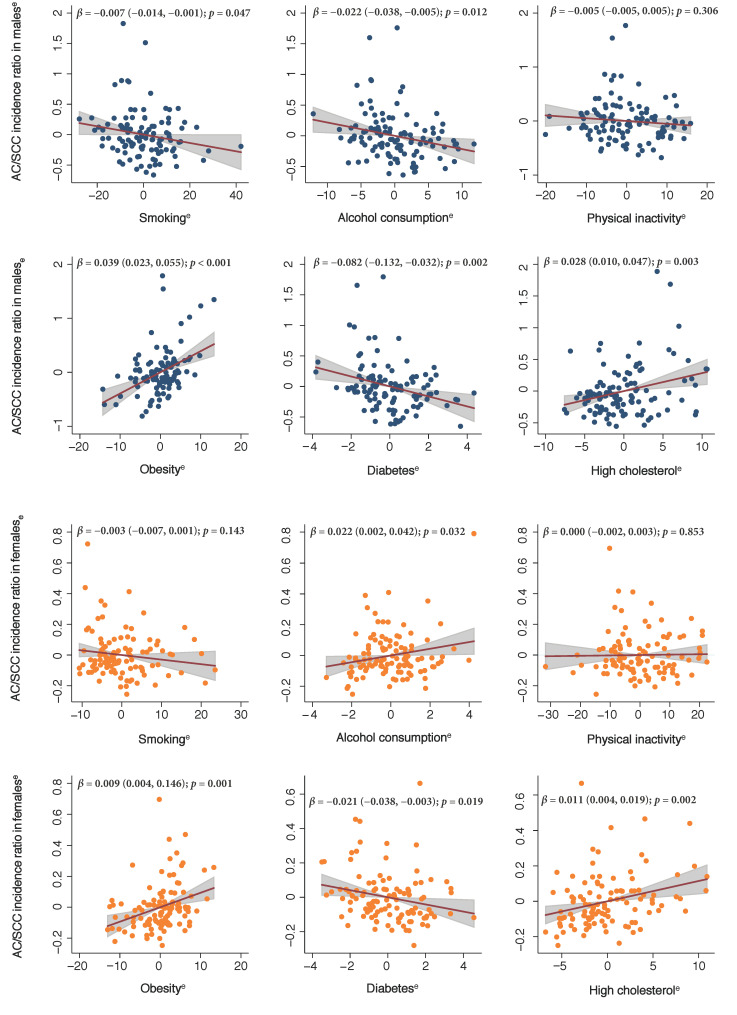
The risk factors associated with histological subtype ratio of esophageal cancer. These added-variable plots were generated using multi-variable linear regression models. ^e^, residuals; AC, adenocarcinoma; SCC, squamous cell carcinoma; β, β coefficient in the linear regression.

Among males, a higher AC/SCC incidence ratio was associated with a higher prevalence of obesity (β 0.039, 95% CI 0.023 to 0.055) and high cholesterol (β 0.028, 95% CI 0.010 to 0.047), but a lower prevalence of smoking (β −0.007, 95% CI −0.014 to −0.001), alcohol consumption (β −0.022, 95% CI −0.038 to −0.005), and diabetes (β −0.082, 95% CI −0.132 to −0.032) in different countries (Figure 3). Among females, a higher AC/SCC incidence ratio was associated with a higher prevalence of alcohol consumption (β 0.022, 95% CI 0.002 to 0.042), obesity (β 0.009, 95% CI 0.004 to 0.146), and high cholesterol (β 0.011, 95% CI 0.004 to 0.019), but a lower prevalence of diabetes (β −0.021, 95% CI −0.038 to −0.003).

### 3.3. Temporal Trends

The incidence and mortality trends of esophageal cancer for each country/region between 1980 and 2017 are shown in Figure S1, and the results from the joinpoint regression analysis are presented in Table S5 and Figure S2.

#### 3.3.1. Incidence Trend

**Figure 4:**
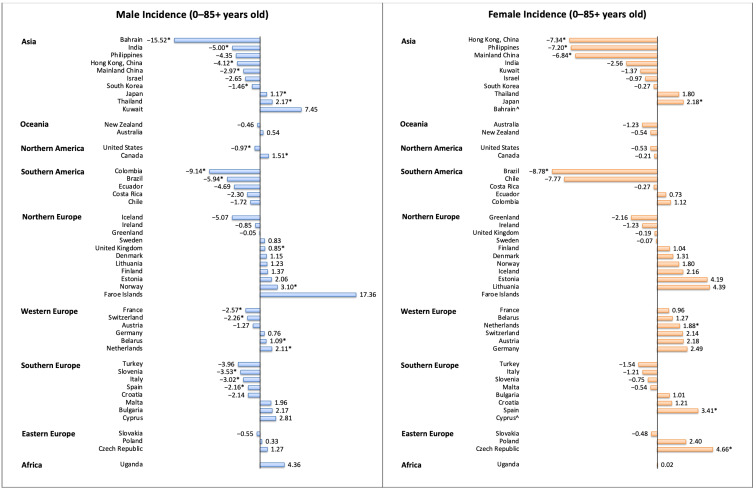
The AAPC of the incidence of esophageal cancer in individuals aged 0–85+ years. AAPC, annual percentage change; * *p* values less than 0.05; ^ AAPC for these countries could not be generated, as zero or missing values were identified in any year of trend analysis; the 95% confidence intervals and *p* values for the tests of AAPC are presented in Table S5.

Among males, 10 regions had a decrease in incidence, and 32 regions reported stable trends (Figure 4). Regions with the most drastic decrease were Bahrain (AAPC −15.52, 95% CI −26.15 to −3.36), Colombia (AAPC −9.14, 95% CI −12.96 to −5.16), Brazil (AAPC −5.94, 95% CI −10.44 to −1.21), and India (AAPC −5.00, 95% CI −8.18 to −1.70). In contrast, six regions showed an increasing trend. Norway (AAPC 3.10, 95% CI 1.23 to 5.00), Thailand (AAPC 2.17, 95% CI 0.74 to 3.62), the Netherlands (AAPC 2.11, 95% CI 1.25 to 2.98), and Canada (AAPC 1.51, 95% CI 0.54 to 2.49) showed the most significant increases. Among females, six regions had a decrease in incidence. Regions with the most drastic decrease were reported in Brazil (AAPC −8.78, 95% CI −14.32 to −2.89), India (AAPC −7.34, 95% CI −10.25 to −4.34), the Philippines (AAPC −7.20, 95% CI −12.21 to −1.90), and Hong Kong Special Administrative Region (HKSAR) of China (AAPC −6.84, 95% CI −7.79 to −5.88). In contrast, four regions showed an increasing trend, including the Czech Republic (AAPC 4.66, 95% CI 0.47 to 9.03), Spain (AAPC 3.41, 95% CI 0.90 to 5.99), Japan (AAPC 2.18, 95% CI 0.58 to 3.80), and the Netherlands (AAPC 1.88, 95% CI 0.51 to 3.27).

#### 3.3.2. Mortality Trend

**Figure 5:**
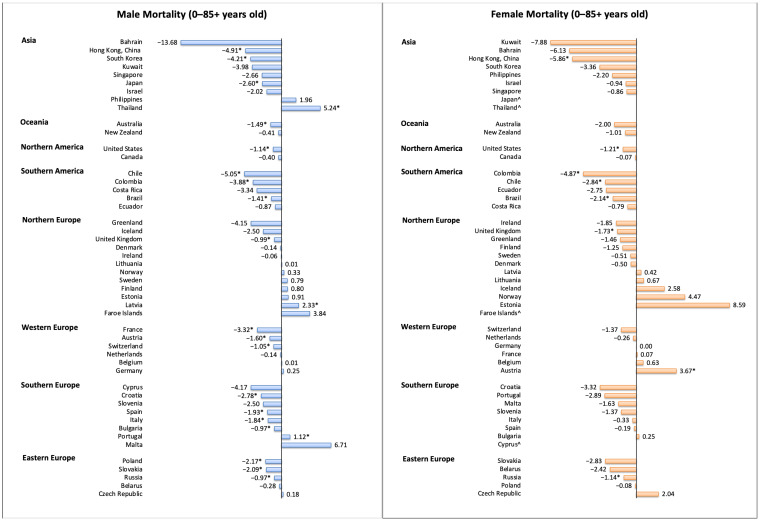
The AAPC of the mortality of esophageal cancer in individuals aged 0–85+ years. AAPC, annual percentage change; * *p* values less than 0.05; ^ AAPC for these countries could not be generated, as zero or missing values were identified in any year of trend analysis; the 95% confidence intervals and *p* values for the tests of AAPC are presented in Table S5.

Among males, 21 regions had a decrease in mortality, and 24 regions reported stable trends (Figure 5). Regions with the most drastic decrease were reported in USA (AAPC −5.24, 95% CI −6.01 to −4.45), Chile (AAPC −5.05, 95% CI −5.89 to −4.21), HKSAR of China (AAPC −4.91, 95% CI −5.73 to −4.08), Korea (AAPC −4.21, 95% CI −5.19 to −3.22), Colombia (AAPC −3.88, 95% CI −5.67 to −2.06), and France (AAPC −3.32, 95% CI −3.71 to −2.93). In contrast, three regions showed an increasing trend, including Thailand (AAPC 5.24, 95% CI 4.76 to 5.72), Latvia (AAPC 2.33, 95% CI 0.45 to 4.25), and Portugal (AAPC 1.12, 95% CI 0.24 to 2.01). Among females, eight regions had a decrease in mortality, and 36 regions reported stable trends. Regions with the most drastic decrease were reported in HKSAR of China (AAPC −5.86, 95% CI −8.79 to −2.84), Colombia (AAPC −4.87, 95% CI −7.12 to −2.57), the USA (black, AAPC −3.91, 95% CI −5.19 to −2.62), and Chile (AAPC −2.84, 95% CI −4.35 to −1.30). In contrast, only one region showed an increasing trend (Austria, AAPC 3.67, 95% CI 0.76 to 6.67).

#### 3.3.3. Incidence Trend by Age Groups

**Figure 6:**
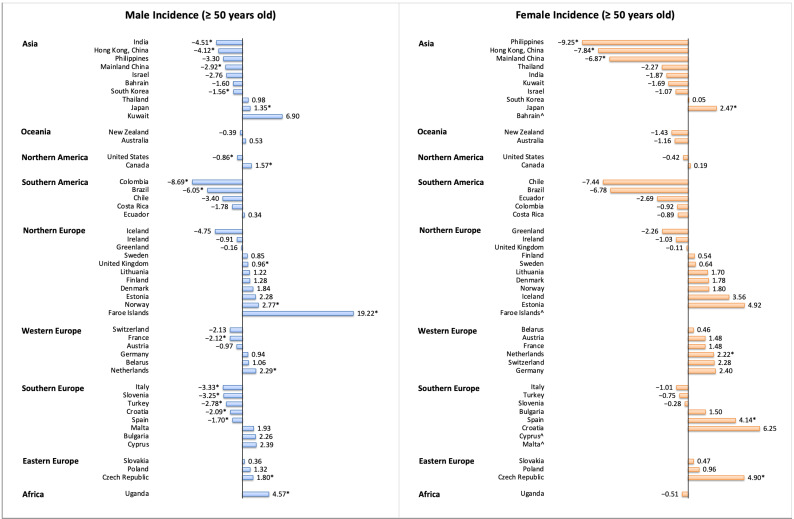
The AAPC of the incidence of esophageal cancer in individuals aged ≥50 years. AAPC, annual percentage change; * *p* values less than 0.05; ^ AAPC for these countries could not be generated, as zero or missing values were identified in any year of trend analysis; the 95% confidence intervals and *p* values for the tests of AAPC are presented in Table S5.

The incidence decreased in 16 countries among individuals aged ≥50 years, and nine countries reported increasing trends (Figure 6). The most marked decrease was observed in the Philippines (female: AAPC −9.25, 95% CI −14.33 to −3.87), Colombia (male: AAPC −8.69, 95% CI −12.23 to −5.01), HKSAR of China (male: AAPC −4.12, 95% CI −5.22 to −3.00; female: AAPC −7.84, 95% CI −10.67 to −4.92), Mainland China (male: AAPC 2.92, 95% CI −3.80 to −2.02; female: AAPC −6.87, 95% CI −7.97 to −5.76), and Brazil (male: AAPC −6.05, 95% CI −10.66 to −1.20). The most marked increase was observed in Faroe Islands (male: AAPC 19.22, 95% CI 1.17 to 40.49), the Czech Republic (male: AAPC 1.80, 95% CI 0.37 to 3.24; female: AAPC 4.90, 95% CI 2.44 to 7.42), Uganda (male: AAPC 4.57, 95% CI 0.09 to 9.25), Spain (female: AAPC 4.14, 95% CI 0.47 to 7.93), and Norway (male: AAPC 2.77, 95% CI 0.86 to 4.73). As for individuals aged <50 years, the incidence of esophageal cancer decreased in seven countries and increased in two countries (Figure S3). The most marked decrease was observed in Mainland China (male: AAPC −3.60, 95% CI −6.53 to −0.58; female: AAPC −7.67, 95% CI −13.26 to −1.73), Slovakia (male: AAPC −7.45, 95% CI −12.02 to −2.63), Turkey (male: AAPC −7.21, 95% CI −13.70 to −0.24), Spain (male: AAPC −6.56, 95% CI −10.13 to −2.86), and France (male: AAPC −6.40, 95% CI −9.15 to −3.56). The increase was observed in Thailand (male: AAPC 12.01, 95% CI 5.57 to 18.84) and Norway (male: AAPC 6.79, 95% CI 0.10 to 13.93).

## 4. Discussion

This study provides the most updated assessment of the global burden, risk factors, as well as the epidemiological trends of esophageal cancer by gender, age, and histological type using data from cancer registries in 48 countries/regions. This study highlights several major epidemiological patterns of esophageal cancer. Firstly, the highest incidence and mortality rates of esophageal cancer were observed in Eastern Asia and Eastern Africa, while the lowest rates were found in Central America and Western Africa. Secondly, the highest incidence rates of AC were observed in the Netherlands, the United Kingdom, and Ireland, with a higher AC/SCC incidence ratio associated with a higher prevalence of obesity and elevated cholesterol. Thirdly, most countries/regions showed a decreasing trend in the incidence and mortality of esophageal cancer in the recent past decade, especially among males and those aged 50 years or older. Nevertheless, an increasing incidence and mortality of esophageal cancer were observed in some developed countries, especially for regions with high AC/SCC incidence ratios.

There was a substantial disparity in the epidemiology of esophageal cancer across different regions in 2018. Our results showed the highest disease burden of esophageal cancer in Eastern Asia, Eastern Africa, South Africa, and Northern Europe, and lower burden in Central America, Western Africa, Northern Africa, and Western Asia. These findings are generally consistent with those of a GBD study for esophageal cancer in 2017, although there was a slight difference in the order (Eastern Asia, South Africa, and Eastern Africa) [18]. This may be caused by the different time frames, data sources, and methods of modelling used for the estimation. For several decades, many places of Eastern Asia, Southeast Asia, South Asia, and Eastern Africa have been shown to have a high prevalence of esophageal malignancy [33]. These regions are mostly along the routes of the Silk Road and have been referred to as the “Asian esophageal cancer belt” [33]. It has been hypothesized that populations in these countries may share some common genetic predispositions for the increased risk of esophageal cancer [33]. Moreover, the variation may be attributed to the disparity in the prevalence of dietary and environmental risk factors across regions. For instance, Asian population had a higher prevalence of drinking hot beverages and lower intakes of fruit and vegetables, which were risk factors for SCC [34]. The lower male to female ratio of esophageal cancer in the African population could be attributed to etiological insights (e.g., lifestyle and environmental factors) and referral bias caused by gender inequalities in healthcare [35]. Results from the current study suggest SCC remains the predominant subtype of esophageal malignancy globally when compared to AC. While the prevalence of SCC was the highest among Asian countries, AC was more frequent in western countries. This was also reported in the previous analysis, although the timeframes and methods used for the estimation varied between studies [13,14,15]. The results also indicated that males had a substantially higher incidence than females for both histological subtypes, but this phenomenon was more marked for AC. The male-to-female ratio in the incidence of AC was up to 9:1 in the United States [36]. The reasons for this remarkable gender difference in the incidence of AC remain unclear, and sex hormones may play a role [37,38,39]. As for the risk factors, our results indicated that obesity and high cholesterol were more associated with AC, while smoking and alcohol drinking were more associated with SCC among males. This supports previous findings that obesity and metabolic syndrome were more common in AC than in SCC [40,41]. In contrast, studies identified the two strongest risk factors for SCC to be tobacco use and alcohol drinking, accounting for over 70% of total SCC cases among high-income countries or regions [42]. However, we did not identify a positive association between prevalence of smoking, alcohol drinking, and incidence of SCC among females. This is consistent with a previous case-control study that although the incidence of SCC attributable to smoking and heavy alcohol consumption is evident among males, such associations were not observed for females, and suboptimal nutrition may play a role in the difference [42].

Despite the substantial regional variation in the distribution of esophageal cancer burden, our findings demonstrate an overall decreasing trend of its incidence and mortality for the past decade. This trend was already identified in the earlier studies using data on incidence and mortality of esophageal cancer up to 2007 [5,16,17]. We also observed a more evident incidence increase among the male population, older adults, and countries with low AC/SCC incidence ratios. Probable reasons for the decreasing incidence and mortality observed may include the reduction in major risk factors, socioeconomic development, and the advancement of treatment. The prevalence of tobacco use and alcohol drinking has been decreasing globally since the 1980s, which could be relate to the general declining trend of SCC since the 1990s [13,17]. The observed decrease in incidence of SCC has been associated with socioeconomic development of many countries. We previously found that there was an inverse association between the incidence of SCC and level of GDP and HDI at the country level [5]. The substantial reduction in the incidence of SCC in many less developed regions might be attributed to the decreased prevalence of risk factors associated with improving socioeconomic status [5,18]. Although the prognosis of esophageal cancer remains poor, the general decrease in mortality may, at least to some extent, be explained by the advancement of diagnostic technology and treatment, such as detection and treatment for early-stage esophageal cancer by upper endoscopy, neoadjuvant chemotherapy, and minimally invasive esophagectomy [7]. For instance, higher survival rates can be reached by the CROSS and docetaxel-based FLOT according to the evidence from randomized controlled trials [3,4].

Notably, there is an increasing trend identified in a substantial proportion of countries, especially for the high-income regions and populations with high AC/SCC incidence ratios. The reasons behind the increasing trend of esophageal cancer of AC remain speculative. This is likely due to the recent increasing prevalence of obesity and metabolic syndrome, which were significant risk factors for AC but not SCC. It was shown that an increase in BMI for every 5 units contributed to a 52% increase in risk for AC [43]. The association was even stronger for waist circumference [44]. In contrast, obesity or central obesity could, however, reduce the risk for SCC [45]. According to a recent estimation on worldwide obesity from the WHO in 2016, the global prevalence of obesity has nearly tripled since 1975 [46]. Another study on the global burden of central obesity also found that the prevalence increased from 16.3% to 33.9% during 1985–2014 [47]. The past two decades also witnessed a marked increase in the prevalence of metabolic syndrome (from 22.8% to 27.0%) [48]. The increase in AC may also be related to the high prevalence of GERD and Barrett’s esophagus, which are strong risk factors for AC, especially among the high-income regions [49]. Obesity is also associated with a higher risk of GERD and Barrett’s esophagus [50]. *H. pylori* is also associated with the reduced risk of AC, since it leads to atrophic gastritis and achlorhydria [51]. *H. pylori* can lower gastric acid secretion, and its eradication was associated with a higher risk of GERD [52]. Although there was an increasing incidence, its mortality was decreasing in some countries, including Japan and the UK. This could be attributable to their successful measures in treatment and prevention for esophageal cancer [53,54].

This study used data from population-based cancer registries of high quality with a sum of over one million cases. This study is large-scale with a comprehensive evaluation of the recent temporal patterns of esophageal cancer among 48 countries/regions. However, there exist some limitations. First, there could be under-reporting of the incidence/mortality in less developed regions, which might be attributed to the underdevelopment of infrastructure and mechanisms of cancer reporting in these places. Second, the numbers might have been overestimated or underestimated, since the data were represented by cancer registries of major cities in some regions. Third, a direct comparison between some regions could be limited, as the cancer registration system might change across regions and over time. However, this limitation is of less concern, as we compared the incidence and mortality according to age and gender groups within the same country. Furthermore, there was a lack of analysis on the trends of the causes and tumor stages of esophageal cancer, which is also important for public health measurements and clinical practice. Lastly, most of the cancer registries used for trend analysis were only updated to 2012. Further investigation is needed when more updated data are available.

Esophageal cancer remains a major cause of cancer burden. With the global ageing in population and expansion in population size, a further increase in its disease burden could be expected—especially for AC, which is positively associated with an increasing prevalence of obesity, metabolic syndrome, and socioeconomic development. AC has already surpassed SCC to be the most frequent histological subtype of esophageal cancer in some western countries and is expected to increase in other countries. More resources should also be committed to the formulation of evidence-based prevention strategies for both SCC and AC. Smoking cessation can decrease the risk of SCC, particularly among populations from western countries [55], and control of alcohol consumption is also recommended. To cope with the future global increase of AC, it is important to closely monitor and slow down the growing rates of obesity and metabolic syndrome, which are also the risk factors for cardiovascular diseases and other cancers. The low survival rate of esophageal cancer might also call for early detection by screening, particularly for the high-risk populations. The Cytosponge can collect a large sample size of esophageal cells for analysis and be managed in the primary care settings [56]. The novel device has been regarded as a safe and accurate method of specimen collection for the screening of Barrett’s esophagus [57]. It could also potentially be used in the future for the detection of squamous dysplasia [58]. The premalignant lesions of esophageal cancer can now be treated with more advanced technology that is much less invasive than open surgery [7]. Population-based targeted screening endoscopy would be feasible in improving survival and would be cost-effective for high-risk individuals for both SCC [59,60] and AC [61]. Future studies should investigate the reasons behind these epidemiological changes and the cost-effectiveness of different preventive strategies, which may offer further insights into the specific etiology and management of esophageal cancer by histological subtypes. The recent COVID-19 pandemic has substantially increased the avoidable cancer mortality [62]. How it affects the global epidemiology of SCC and AC in the near future remains to be explored.

## 5. Conclusions

We conclude that although the incidence of esophageal cancer showed an overall decreasing trend, an increasing trend was observed in some countries with high AC/SCC incidence ratios. It is important to closely monitor and slow down the growing rates of obesity and metabolic syndrome, which are the important risk factors for adenocarcinoma. Population-based targeted screening endoscopy would be recommended for high-risk individuals with more advanced and less invasive technology developed. How the recent pandemic of COVID-19 affects the global epidemiology of SCC and AC in the near future remains to be explored.

## Data Availability

The data used for the analyses are publicly available from the World Health Organization websites (https://gco.iarc.fr/).

## Supplementary Materials

The following are available online at https://www.mdpi.com/2072-6694/13/1/141/s1, Table S1: The data source of the analysis of esophageal cancer, Table S2: The data source of the analysis of esophageal cancer, Table S3: The incidence and mortality of esophageal cancer by region, Table S4: The incidence of esophageal cancer by histological subtype, Table S5: The trend analysis of esophageal cancer by country, Figure S1: The incidence and mortality trends of esophageal cancer, Figure S2: The joinpoint regression analysis of esophageal cancer, Figure S3: The AAPC of the incidence of esophageal cancer in individuals aged <50 years.

## Abbreviations

AAPC	average annual percent change
AC	adenocarcinoma
ASR	age-standardized rates
CI	confidence interval
CI5	Cancer Incidence in Five Continents
CROSS	ChemoRadiotherapy for Esophageal cancer followed by Surgery Study
FLOT	fluorouracil plus leucovorin, oxaliplatin, and docetaxel
GERD	gastro-esophageal reflux disease
GHO	Global Health Observatory
NORDCAN	Nordic Cancer Registries
SCC	squamous cell carcinoma
SEER	surveillance, epidemiology, and end results

## References

[1] Bray F., Ferlay J., Soerjomataram I., Siegel R.L., Torre L.A., Jemal A. (2018). Global cancer statistics 2018: GLOBOCAN estimates of incidence and mortality worldwide for 36 cancers in 185 countries. CA A Cancer J. Clin..

[2] American Cancer Society (2020). Survival Rates for Esophageal Cancer. https://www.cancer.org/cancer/esophagus-cancer/detection-diagnosis-staging/survival-rates.html#references.

[3] Shapiro J., van Lanschot J.J.B., Hulshof M., van Hagen P., van Berge Henegouwen M.I., Wijnhoven B.P.L., van Laarhoven H.W.M., Nieuwenhuijzen G.A.P., Hospers G.A.P., Bonenkamp J.J. (2015). Neoadjuvant chemoradiotherapy plus surgery versus surgery alone for oesophageal or junctional cancer (CROSS): Long-term results of a randomised controlled trial. Lancet Oncol..

[4] Al-Batran S.E., Homann N., Pauligk C., Goetze T.O., Meiler J., Kasper S., Kopp H.G., Mayer F., Haag G.M., Luley K. (2019). Perioperative chemotherapy with fluorouracil plus leucovorin, oxaliplatin, and docetaxel versus fluorouracil or capecitabine plus cisplatin and epirubicin for locally advanced, resectable gastric or gastro-oesophageal junction adenocarcinoma (FLOT4): A randomised, phase 2/3 trial. Lancet.

[5] Wong M.C.S., Hamilton W., Whiteman D.C., Jiang J.Y., Qiao Y., Fung F.D.H., Wang H.H.X., Chiu P.W.Y., Ng E.K.W., Wu J.C.Y. (2018). Global Incidence and mortality of oesophageal cancer and their correlation with socioeconomic indicators temporal patterns and trends in 41 countries. Sci. Rep..

[6] Fitzmaurice C., Dicker D., Pain A., Hamavid H., Moradi-Lakeh M., MacIntyre M.F., Allen C., Hansen G., Woodbrook R., Wolfe C. (2015). The Global Burden of Cancer 2013. JAMA Oncol..

[7] Pennathur A., Gibson M.K., Jobe B.A., Luketich J.D. (2013). Oesophageal carcinoma. Lancet.

[8] Lepage C., Rachet B., Jooste V., Faivre J., Coleman M.P. (2008). Continuing rapid increase in esophageal adenocarcinoma in England and Wales. Am. J. Gastroenterol..

[9] Eslick G.D. (2009). Epidemiology of esophageal cancer. Gastroenterol. Clin. N. Am..

[10] Abnet C.C., Arnold M., Wei W.Q. (2018). Epidemiology of Esophageal Squamous Cell Carcinoma. Gastroenterology.

[11] Coleman H.G., Xie S.H., Lagergren J. (2018). The Epidemiology of Esophageal Adenocarcinoma. Gastroenterology.

[12] Xie S.H., Lagergren J. (2018). Risk factors for oesophageal cancer. Best Pr. Res. Clin. Gastroenterol..

[13] Wang Q.L., Xie S.H., Wahlin K., Lagergren J. (2018). Global time trends in the incidence of esophageal squamous cell carcinoma. Clin. Epidemiol..

[14] Arnold M., Soerjomataram I., Ferlay J., Forman D. (2015). Global incidence of oesophageal cancer by histological subtype in 2012. Gut.

[15] Arnold M., Ferlay J., van Berge Henegouwen M.I., Soerjomataram I. (2020). Global burden of oesophageal and gastric cancer by histology and subsite in 2018. Gut.

[16] Malhotra G.K., Yanala U., Ravipati A., Follet M., Vijayakumar M., Are C. (2017). Global trends in esophageal cancer. J. Surg. Oncol..

[17] Arnold M., Laversanne M., Brown L.M., Devesa S.S., Bray F. (2017). Predicting the Future Burden of Esophageal Cancer by Histological Subtype: International Trends in Incidence up to 2030. Am. J. Gastroenterol..

[18] GBD 2017 Oesophageal Cancer Collaborators (2020). The global, regional, and national burden of oesophageal cancer and its attributable risk factors in 195 countries and territories, 1990–2017: A systematic analysis for the Global Burden of Disease Study 2017. Lancet Gastroenterol. Hepatol..

[19] Wong M.C., Goggins W.B., Wang H.H., Fung F.D., Leung C., Wong S.Y., Ng C.F., Sung J.J. (2016). Global Incidence and Mortality for Prostate Cancer: Analysis of Temporal Patterns and Trends in 36 Countries. Eur. Urol..

[20] Wong M.C., Huang J., Lok V., Wang J., Fung F., Ding H., Zheng Z.J. (2020). Differences in Incidence and Mortality Trends of Colorectal Cancer, Worldwide, Based on Sex, Age, and Anatomic Location. Clin. Gastroenterol. Hepatol..

[21] Cancer Incidence in Five Continents. https://ci5.iarc.fr.

[22] Surveillance, Epidemiology, and End Results (SEER) Program. https://seer.cancer.gov/about/.

[23] The NORDCAN Project. https://www-dep.iarc.fr/NORDCAN/english/frame.asp.

[24] Engholm G., Ferlay J., Christensen N., Bray F., Gjerstorff M.L., Klint A., Kotlum J.E., Olafsdottir E., Pukkala E., Storm H.H. (2010). NORDCAN—a Nordic tool for cancer information, planning, quality control and research. Acta Oncol..

[25] World Health Organization WHO Mortality Database. https://www.who.int/healthinfo/mortality_data/en/.

[26] Mathers C.D., Fat D.M., Inoue M., Rao C., Lopez A.D. (2005). Counting the dead and what they died from: An assessment of the global status of cause of death data. Bull World Health Organ.

[27] International Agency for Research on Cancer, W.H.O. Data Visualization Tools for Exploring the Global Cancer Burden in 2018. https://gco.iarc.fr/today/home.

[28] World Health Organization International Statistical Classification of Diseases and Related Health Problems 10th Revision. https://icd.who.int/browse10/2016/en.

[29] Segi M., Fujisaku S., Kurihara M. (1957). Geographical observation on cancer mortality by selected sites on the basis of standardised death rate. Gan.

[30] Kim H.J., Fay M.P., Feuer E.J., Midthune D.N. (2000). Permutation tests for joinpoint regression with applications to cancer rates. Stat. Med..

[31] Division of Health Informatics, C.o.P. Cancer Trend Analysis Using Joinpoint Regression. https://www.health.pa.gov/topics/HealthStatistics/Statistical-Resources/UnderstandingHealthStats/Documents/Cancer_Trend_Analysis_Using_Joinpoint_Regression_Part_1_The_Basics.pdf.

[32] Clegg L.X., Hankey B.F., Tiwari R., Feuer E.J., Edwards B.K. (2009). Estimating average annual per cent change in trend analysis. Stat. Med..

[33] Kmet J., Mahboubi E. (1972). Esophageal cancer in the Caspian littoral of Iran: Initial studies. Science.

[34] Zhang H.-Z., Jin G.-F., Shen H.-B. (2012). Epidemiologic differences in esophageal cancer between Asian and Western populations. Chin. J. Cancer.

[35] Middleton D.R.S., Bouaoun L., Hanisch R., Bray F., Dzamalala C., Chasimpha S., Menya D., Mbalawa C.G., N’Da G., Woldegeorgis M.A. (2018). Esophageal cancer male to female incidence ratios in Africa: A systematic review and meta-analysis of geographic, time and age trends. Cancer Epidemiol..

[36] Lagergren J., Lagergren P. (2013). Recent developments in esophageal adenocarcinoma. CA A Cancer J. Clin..

[37] Xie S.H., Lagergren J. (2016). The Male Predominance in Esophageal Adenocarcinoma. Clin. Gastroenterol. Hepatol..

[38] Xie S.H., Ness-Jensen E., Rabbani S., Langseth H., Gislefoss R.E., Mattsson F., Lagergren J. (2020). Circulating Sex Hormone Levels and Risk of Esophageal Adenocarcinoma in a Prospective Study in Men. Am. J. Gastroenterol..

[39] Cronin-Fenton D.P., Murray L.J., Whiteman D.C., Cardwell C., Webb P.M., Jordan S.J., Corley D.A., Sharp L., Lagergren J. (2010). Reproductive and sex hormonal factors and oesophageal and gastric junction adenocarcinoma: A pooled analysis. Eur. J. Cancer.

[40] Lindkvist B., Johansen D., Stocks T., Concin H., Bjørge T., Almquist M., Häggström C., Engeland A., Hallmans G., Nagel G. (2014). Metabolic risk factors for esophageal squamous cell carcinoma and adenocarcinoma: A prospective study of 580,000 subjects within the Me-Can project. BMC Cancer.

[41] Agrawal K., Markert R.J., Agrawal S. (2018). Risk factors for adenocarcinoma and squamous cell carcinoma of the esophagus and lung. Hypertension.

[42] Pandeya N., Olsen C.M., Whiteman D.C. (2013). Sex differences in the proportion of esophageal squamous cell carcinoma cases attributable to tobacco smoking and alcohol consumption. Cancer Epidemiol..

[43] Renehan A.G., Tyson M., Egger M., Heller R.F., Zwahlen M. (2008). Body-mass index and incidence of cancer: A systematic review and meta-analysis of prospective observational studies. Lancet.

[44] Steffen A., Schulze M.B., Pischon T., Dietrich T., Molina E., Chirlaque M.D., Barricarte A., Amiano P., Quirós J.R., Tumino R. (2009). Anthropometry and esophageal cancer risk in the European prospective investigation into cancer and nutrition. Cancer Epidemiol. Biomark. Prev..

[45] Lahmann P.H., Pandeya N., Webb P.M., Green A.C., Whiteman D.C. (2012). Body mass index, long-term weight change, and esophageal squamous cell carcinoma: Is the inverse association modified by smoking status?. Cancer.

[46] World Health Organization (2019). Obesity and Overweight. https://www.who.int/news-room/fact-sheets/detail/obesity-and-overweight.

[47] Wong M.C., Huang J., Wang J., Chan P.S., Lok V., Chen X., Leung C., Wang H.H.X., Lao X.Q., Zheng Z.J. (2020). Global, regional and time-trend prevalence of central obesity: A systematic review and meta-analysis of 13.2 million subjects. Eur. J. Epi..

[48] Wong M.C., Huang J., Pang T.W., Lok V., Chen X., Choi P., Leung C., Wang H.H.X., Lao X.Q., Zheng Z.J. (2020). Worldwide incidence and prevalence of metabolic syndrome: A systematic review and meta-analysis of 14.6 million individuals. Gastroenterology.

[49] Lagergren J., Bergström R., Lindgren A., Nyrén O. (1999). Symptomatic gastroesophageal reflux as a risk factor for esophageal adenocarcinoma. N. Engl. J. Med..

[50] Lagergren J. (2011). Influence of obesity on the risk of esophageal disorders. Nat. Rev. Gastroenterol. Hepatol..

[51] McColl K.E., Going J.J. (2010). Aetiology and classification of adenocarcinoma of the gastro-oesophageal junction/cardia. Gut.

[52] Khalifa M.M., Sharaf R.R., Aziz R.K. (2010). Helicobacter pylori: A poor man’s gut pathogen?. Gut Pathog..

[53] Thrumurthy S.G., Chaudry M.A., Thrumurthy S.S.D., Mughal M. (2019). Oesophageal cancer: Risks, prevention, and diagnosis. BMJ.

[54] Tanaka Y., Yoshida K., Suetsugu T., Imai T., Matsuhashi N., Yamaguchi K. (2018). Recent advancements in esophageal cancer treatment in Japan. Ann. Gastroenterol. Surg..

[55] Wang Q.-L., Xie S.-H., Li W.-T., Lagergren J. (2017). Smoking Cessation and Risk of Esophageal Cancer by Histological Type: Systematic Review and Meta-analysis. Jnci J. Natl. Cancer Inst..

[56] di Pietro M., Canto M.I., Fitzgerald R.C. (2018). Endoscopic Management of Early Adenocarcinoma and Squamous Cell Carcinoma of the Esophagus: Screening, Diagnosis, and Therapy. Gastroenterology.

[57] Fitzgerald R.C., di Pietro M., O’Donovan M., Maroni R., Muldrew B., Debiram-Beecham I., Gehrung M., Offman J., Tripathi M., Smith S.G. (2020). Cytosponge-trefoil factor 3 versus usual care to identify Barrett’s oesophagus in a primary care setting: A multicentre, pragmatic, randomised controlled trial. Lancet.

[58] Roshandel G., Merat S., Sotoudeh M., Khoshnia M., Poustchi H., Lao-Sirieix P., Malhotra S., O’Donovan M., Etemadi A., Nickmanesh A. (2014). Pilot study of cytological testing for oesophageal squamous cell dysplasia in a high-risk area in Northern Iran. Br. J. Cancer.

[59] Wei W.Q., Chen Z.F., He Y.T., Feng H., Hou J., Lin D.M., Li X.Q., Guo C.L., Li S.S., Wang G.Q. (2015). Long-Term Follow-Up of a Community Assignment, One-Time Endoscopic Screening Study of Esophageal Cancer in China. J. Clin. Oncol..

[60] Yang J., Wei W.Q., Niu J., Liu Z.C., Yang C.X., Qiao Y.L. (2012). Cost-benefit analysis of esophageal cancer endoscopic screening in high-risk areas of China. World J. Gastroenterol..

[61] Xie S.H., Ness-Jensen E., Medefelt N., Lagergren J. (2018). Assessing the feasibility of targeted screening for esophageal adenocarcinoma based on individual risk assessment in a population-based cohort study in Norway (The HUNT Study). Am. J. Gastroenterol..

[62] Maringe C., Spicer J., Morris M., Purushotham A., Nolte E., Sullivan R., Rachet B., Aggarwal A. (2020). The impact of the COVID-19 pandemic on cancer deaths due to delays in diagnosis in England, UK: A national, population-based, modelling study. Lancet Oncol..

