# Supplementary material for: Global Burden, Risk Factors, and Trends of Esophageal Cancer: An Analysis of Cancer Registries from 48 Countries
Source: Cancers (Basel). 2021 Jan 5;13(1):141. doi: 10.3390/cancers13010141 (PMC7795486; doi:10.3390/cancers13010141)
Supplement: Supplementary file 1 [file cancers-13-00141-s001.pdf]

Supplementary Materials

Table S1. The data source of the analysis of esophageal cancer

Table S2. The data source of the analysis of esophageal cancer

Table S3. The incidence and mortality of esophageal cancer by region

Table S4. The incidence of esophageal cancer by histological subtype

Table S5. The trend analysis of esophageal cancer by country

Figure S1. The incidence and mortality trends of esophageal cancer

Figure S2. The joinpoint regression analysis of esophageal cancer

Figure S3. The AAPC of the incidence of esophageal cancer in individuals aged < 50 years

Table S1. The data source of the analysis of esophageal cancer

	Incidence	Mortality
Australia	CI5 (1993-2012)	WHO (1979-2015)
Austria	CI5 (1998-2012)	WHO (1980-2016)
Bahrain	CI5 (1998-2012)	WHO (1997-2014)
Belarus	CI5 (1983-2012)	WHO (1981-2001)
Belgium	n/a	WHO(1979-2015)
Brazil	CI5 (1993-2012)[1]	WHO (1979-2015)
Bulgaria	CI5 (1998-2012)	WHO (1980-2014)
Canada	CI5 (1983-2012)[2]	WHO (1979-2013)
Chile	CI5 (1998-2012)[3]	WHO (1980-2015)
China	CI5 (1998-2012)[4]	n/a
Colombia	CI5 (1983-2012)[5]	WHO (1984-2015)
Costa Rica	CI5 (1982-2011)	WHO (1980-2014)
Croatia	CI5 (1988-2012)	WHO (1985-2016)
Cyprus	CI5 (1998-2012)	WHO (2004-2016)
Czech Republic	CI5 (1983-2012)	WHO (1986-2016)
Denmark	NORDCAN (1943-2016)	NORDCAN (1951-2016)
Ecuador	CI5 (1985-2012)[6]	WHO (1979-2015)
Estonia	CI5 (1983-2012)	WHO (1981-2015)
Faroe Islands	NORDCAN (1960-2015)	NORDCAN (1983-2013)
Finland	NORDCAN (1953-2016)	NORDCAN (1953-2016)
France	CI5 (1998-2011)[7]	WHO (1979-2014)
Germany	CI5 (1998-2012)[8]	WHO (1990-2015)

Greenland	NORDCAN (1968-2016)	NORDCAN (1983-2016)
Hong Kong	CI5 (1983-2012)	WHO (1979-2015)
Iceland	NORDCAN (1955-2016)	NORDCAN (1955-2016)
India	CI5(1983-2012)[9]	n/a
Ireland	CI5 (1994-2012)	WHO (1979-2014)
Israel	CI5 (1963-2012)	WHO (1979-2015)
Italy	CI5 (1998-2010) [10]	WHO (1979-2015)
Japan	CI5 (1998-2010)[11]	WHO (1979-2015)
Korea	CI5 (1999-2012)[12]	WHO (1985-2015)[13]
Kuwait	CI5 (1998-2012)	WHO (1979-2014)
Latvia	n/a	WHO (1980-2015)
Lithuania	CI5 (1988-2012)	WHO (1981-2016)
Malta	CI5 (1993-2012)	WHO(1979-2015)
Netherlands	CI5 (1989-2012)	WHO (1979-2016)
New Zealand	CI5 (1983-2012)	WHO (1979-2013)
Norway	NORDCAN (1953-2016)	NORDCAN (1953-2016)
Philippines	CI5 (1983-2012)[14]	WHO (1992-2011)
Poland	CI5 (1998-2012)[15]	WHO (1980-2015)
Portugal	n/a	WHO (1980-2014)
Russian Federation	n/a	WHO (1980-1998)
Singapore	n/a	WHO (1979-2015)
Slovakia	CI5 (1971-2010)	WHO (1992-2014)
Slovenia	CI5 (1983-2012)	WHO (1985-2015)
Spain	CI5 (1993-2010)[16]	WHO (1980-2015)

Sweden	NORDCAN (1960-2016)	NORDCAN (1960-2016)
Switzerland	CI5 (1998-2012)[17]	WHO (1995-2015)
Thailand	CI5 (1993-2012)[18]	WHO (1979-2016)
Turkey	CI5 (1998-2012)[19]	WHO (2009-2015)
Uganda	CI5 (1993-2012)[20]	n/a
United Kingdom	CI5 (1995-2012)[21]	WHO (1979-2015)
USA	SEER(1975-2017) [22]	SEER(1970-2017) [22]
USA Black	SEER(1975-2017) [22]	n/a
USA White	SEER(1975-2017) [22]	n/a

n/a" not available; CI5: Cancer Incidence in Five Continents V; NORDCAN: Nordic Cancer Registries' SEER: USA: National Institutes of Health (NIH); WHO: World Health Organization

1. Brazil, Goiania
2. Canada (excl. Nunavut, Quebec and Yukon)
3. Chile, Valdivia
4. China (5 registries)
5. Colombia, Cali
6. Ecuador, Quito
7. France (9 registries)
8. Germany (2 registries)
9. India, Chennai
10. Italy (8 registries)
11. Japan (4 registries)

12. Korea (5 registries)

13. Republic of Korea

14. Philippines, Manila

15. Poland, Kielce

16. Spain (9 registries)

17. Switzerland (6 registries)

18. Thailand (4 registries)

19. Turkey (2 registries)

20. Uganda, Kampala

21. UK, England

22. USA, (9 registries)

Reference:

a. CI5: http://ci5.iarc.fr/CI5plus/Pages/table1_sel.aspx

b. NORDCAN: http://www-dep.iarc.fr/NORDCAN/english/frame.asp

c. SEER: http://seer.cancer.gov/data/seerstat/

d. WHO: http://apps.who.int/healthinfo/statistics/mortality/whodpms/

Table S2. The data source of the analysis of esophageal cancer

Risk factors	Measurement	Year
Smoking [a]	Current smoking of any tobacco product (age-standardized rate)	2010
Alcohol [b]	Total per capita consumption	2010
Physical inactivity [c]	Prevalence of insufficient physical activity (age-standardized rate)	2016*
Obesity [d]	Prevalence of obesity (age-standardized rate)	2010
Diabetes [e]	Raised fasting blood glucose (≥ 7.0 mmol/L or on medication, age-standardized rate)	2010
High cholesterol [f]	Raised total cholesterol (≥6.2 mmol/L, age-standardized)	2008*

*Data are only available for this year.

Reference

a. https://www.who.int/data/gho/indicator-metadata-registry/imr-details/346

b. https://www.who.int/data/gho/indicator-metadata-registry/imr-details/4759

c. https://apps.who.int/gho/data/node.main.A893?lang=en

d. https://apps.who.int/gho/data/node.main.BMIOBESITYA?lang=en

e. https://apps.who.int/gho/data/node.main.NCDRGLUCA?lang=en

f. https://apps.who.int/gho/data/node.main.A887?lang=en

Table S3. The incidence and mortality of esophageal cancer by region

	Incidence						Mortality					
	Both sexes		Males		Females		Both sexes		Males		Females	
Region	New cases	ASR	New cases	ASR	New cases	ASR	Deaths	ASR	Deaths	ASR	Deaths	ASR
Eastern Africa	17 792	8.3	9 657	9.7	8 135	7.1	17 564	8.2	9 574	9.7	7 990	7.0
Middle Africa	1 564	1.9	855	2.1	709	1.7	1 450	1.8	772	2.0	678	1.7
Northern Africa	3 093	1.5	1 627	1.7	1 466	1.4	2 900	1.5	1 509	1.6	1 391	1.3
Southern Africa	3 913	7.4	2 331	11.1	1 582	5.0	3 793	7.2	2 297	11.0	1 496	4.8
Western Africa	2 132	1.2	1 358	1.6	774	0.8	1 996	1.2	1 292	1.6	704	0.8
Caribbean	1 882	3.3	1 488	5.6	394	1.2	1 645	2.8	1 315	4.9	330	1.0
Central America	1 756	1.0	1 289	1.6	467	0.5	1 661	0.9	1 257	1.5	404	0.4
South America	17 337	3.3	12 673	5.5	4 664	1.5	14 588	2.8	10 935	4.7	3 653	1.2
North America	22 689	3.3	18 052	5.6	4 637	1.2	18 222	2.5	14 564	4.3	3 658	0.9
Eastern Asia	335 080	12.2	237 160	17.9	97 920	6.8	300 878	10.7	212 430	15.8	88 448	5.9
South-Eastern Asia	12 864	1.9	10 418	3.3	2 446	0.7	11 857	1.8	9 614	3.1	2 243	0.6
South-Central Asia	92 741	5.3	58 395	6.7	34 346	3.8	81 432	4.6	53 404	6.2	28 028	3.1
Western Asia	3 912	1.7	2 055	1.9	1 857	1.5	3 578	1.5	1 924	1.8	1 654	1.3
Central and Eastern Europe	15 616	3.0	12 723	5.9	2 893	0.8	14 140	2.7	11 631	5.3	2 509	0.7
Western Europe	17 980	4.2	13 732	6.8	4 248	1.7	14 423	3.1	11 177	5.2	3 246	1.2
Southern Europe	6 449	1.9	5 153	3.3	1 296	0.6	5 644	1.5	4 545	2.8	1 099	0.5
Northern Europe	12 919	5.5	9 059	8.5	3 860	2.7	10 854	4.3	7 579	6.7	3 275	2.1
Australia and New Zealand	2 048	3.5	1 492	5.5	556	1.6	1 708	2.7	1 201	4.2	507	1.3
Melanesia	238	3.4	155	4.9	83	2.2	227	3.3	147	4.8	80	2.1
Polynesia	17	2.5	17	5.0	NA	NA	13	1.9	13	3.9	NA	NA
Micronesia	12	2.2	10	3.8	2	0.8	12	2.2	10	3.8	2	0.8
World	572 034	6.3	399 699	9.3	172 335	3.5	508 585	5.5	357 190	8.3	151 395	3.0

ASR, age-standardized rate; NA, not available.

Table S4. The incidence of esophageal cancer by histological subtype

Country	Male					Female				
	AC		SCC		AC:SCC (ASR)	AC		SCC		AC:SCC (ASR)
	ASR	N	ASR	N		ASR	N	ASR	N	
Afghanistan	1.1	79	8.6	670	0.124	0.5	36	6.3	527	0.071
Albania	0.4	12	1.2	33	0.308	0.0	2	0.2	6	0.200
Algeria	0.3	54	0.6	118	0.500	0.1	20	0.6	129	0.154
Angola	0.2	10	3.4	203	0.051	0.1	5	1.6	119	0.061
Argentina	1.8	477	3.7	1028	0.476	0.4	169	1.5	625	0.235
Armenia	0.6	11	1.2	24	0.467	0.1	2	0.5	15	0.200
Australia	3.5	790	1.9	439	1.789	0.5	132	1.0	316	0.455
Austria	1.8	155	2.7	223	0.667	0.3	33	0.7	69	0.400
Azerbaijan	2.1	99	4.8	218	0.452	0.7	39	3.8	238	0.179
Bahamas	0.8	2	2.8	7	0.286	0.1	0	0.3	1	0.250
Bahrain	0.9	3	0.4	2	2.143	0.3	1	0.9	3	0.300
Bangladesh	2.0	1426	16.7	12057	0.121	0.7	472	10.0	6951	0.067
Barbados	0.5	2	1.5	4	0.320	0.1	1	0.3	1	0.400
Belarus	0.9	64	7.0	459	0.136	0.1	13	0.3	32	0.333
Belgium	3.4	390	3.7	398	0.897	0.7	103	1.6	206	0.429
Belize	0.4	1	1.2	2	0.281	NA	NA	NA	NA	0.250
Benin	0.5	6	6.5	184	0.077	0.0	0	4.6	148	0.000
Bhutan	0.8	3	6.6	23	0.121	0.4	1	5.7	17	0.067
Bolivia	0.5	26	1.8	85	0.300	0.2	8	0.5	28	0.333
Bosnia Herzegovina	0.9	32	3.1	92	0.308	0.1	6	0.6	21	0.200
Botswana	0.6	4	11.2	69	0.056	0.2	2	3.4	30	0.050
Brazil	1.5	1675	5.9	6981	0.247	0.4	611	1.4	2138	0.286
Brunei	0.0	0	3.2	5	0.000	NA	NA	NA	NA	0.091

Bulgaria	1.0	63	2.2	142	0.429	0.1	15	0.4	29	0.333
Burkina Faso	0.1	4	1.4	60	0.050	0.0	2	0.9	52	0.034
Burundi	0.8	17	14.0	342	0.056	0.3	7	5.7	154	0.051
Cambodia	0.2	10	4.4	213	0.051	0.1	3	0.9	62	0.083
Cameroon	0.1	3	1.0	68	0.071	0.0	2	1.4	93	0.025
Canada	3.7	1344	1.8	691	2.000	0.5	216	0.8	368	0.571
Central African Republic	0.1	1	1.9	23	0.074	0.1	1	2.3	33	0.059
Chad	0.1	4	2.2	65	0.050	0.1	3	1.7	56	0.071
Chile	0.5	62	2.6	350	0.171	0.3	54	1.6	318	0.200
China	0.9	9488	18.8	204602	0.046	0.4	3737	7.8	89532	0.048
Colombia	0.9	247	1.6	407	0.579	0.1	52	0.7	216	0.222
Comoros	0.5	1	8.1	18	0.059	0.3	1	6.8	15	0.045
Congo, Democratic	0.1	22	1.9	415	0.059	0.1	16	1.7	352	0.045
Congo, Republic of	0.0	0	0.5	7	0.071	0.0	0	0.9	13	0.000
Costa Rica	0.8	26	1.2	41	0.667	0.2	7	0.5	23	0.500
Cote d Ivoire	0.0	2	0.5	32	0.067	0.0	2	1.1	53	0.040
Croatia	0.7	26	3.5	135	0.200	0.1	10	0.7	36	0.200
Cuba	2.1	215	7.4	744	0.283	0.3	34	0.9	117	0.308
Cyprus	1.0	8	0.7	7	1.333	0.3	3	0.4	4	0.667
Czech Republic	2.5	235	3.8	357	0.647	0.4	50	0.8	100	0.429
Denmark	4.6	286	3.6	206	1.292	0.9	63	1.6	115	0.533
Djibouti	0.2	1	2.7	9	0.056	0.2	1	4.2	14	0.053
Dominican Republic	0.7	35	2.3	114	0.308	0.3	14	0.8	45	0.333
Ecuador	0.4	38	1.5	128	0.273	0.2	21	0.3	32	0.667
Egypt	0.3	106	1.3	474	0.217	0.2	86	0.9	368	0.250
El Salvador	0.4	16	1.4	47	0.313	0.1	8	0.6	30	0.200
Equatorial Guinea	0.1	0	2.2	8	0.048	0.0	0	1.5	4	0.000

Eritrea	0.2	2	3.1	42	0.053	0.2	3	4.3	61	0.044
Estonia	0.5	6	5.3	55	0.100	0.1	1	0.6	14	0.200
Eswatini	0.3	1	4.6	14	0.055	0.0	0	1.1	5	0.034
Ethiopia	0.1	26	2.1	581	0.053	0.2	47	3.6	1098	0.045
Fiji	0.4	2	2.8	12	0.154	NA	NA	NA	NA	NA
Finland	1.8	105	1.8	108	1.000	0.3	28	0.8	67	0.375
France (metropolitan)	1.7	1152	4.8	3032	0.364	0.3	285	1.3	943	0.214
France, Guadeloupe	1.4	5	4.8	18	0.289	0.2	2	0.6	3	0.286
France, La Reunion	0.4	3	8.3	51	0.051	0.0	0	1.3	9	0.000
France, Martinique	0.9	4	3.2	13	0.286	0.1	1	0.5	3	0.250
France, New Caledonia	0.7	1	4.7	9	0.149	NA	NA	NA	NA	NA
French Guyana	1.4	1	4.8	6	0.292	NA	NA	NA	NA	NA
French Polynesia	0.9	2	6.0	11	0.146	NA	NA	NA	NA	NA
Gabon	0.1	2	3.5	24	0.034	0.1	1	1.6	11	0.067
Gaza Strip and West Bank	0.3	3	0.6	8	0.429	0.1	1	0.5	6	0.154
Georgia	0.5	17	1.3	37	0.400	0.1	3	0.4	18	0.125
Germany	2.1	1952	4.5	3992	0.468	0.4	457	1.3	1435	0.273
Ghana	0.1	9	3.3	215	0.044	0.1	5	0.9	77	0.059
Greece	0.5	53	1.4	156	0.333	0.1	14	0.2	42	0.500
Guam	0.5	1	3.2	3	0.160	NA	NA	NA	NA	NA
Guatemala	0.4	22	1.2	71	0.313	0.2	12	0.6	41	0.333
Guinea	0.1	1	0.7	22	0.125	0.0	0	0.1	4	0.000
Guinea-Bissau	0.0	0	3.0	10	0.000	NA	NA	NA	NA	NA
Guyana	0.3	1	0.8	3	0.316	0.1	0	0.4	2	0.250
Haiti	0.5	19	1.9	71	0.278	0.5	24	1.8	77	0.300
Honduras	0.4	11	1.1	38	0.333	0.2	8	0.8	28	0.250
Hungary	1.5	118	6.6	510	0.232	0.2	31	0.9	87	0.286

Iceland	4.1	12	2.8	9	1.444	0.5	2	0.6	3	0.750
India	0.4	2726	5.1	31164	0.082	0.1	864	2.8	17642	0.038
Indonesia	0.1	39	0.7	836	0.071	0.0	14	0.2	265	0.052
Iran, Islamic Republic of	0.7	233	5.9	2249	0.110	0.6	196	4.6	1694	0.125
Iraq	0.5	50	0.4	39	1.286	0.2	22	0.6	61	0.333
Ireland	4.9	197	2.6	103	1.862	1.0	50	2.1	107	0.455
Israel	0.5	28	1.0	61	0.500	0.1	10	0.6	45	0.200
Italy	0.7	476	1.6	1082	0.400	0.1	114	0.5	455	0.250
Jamaica	0.8	15	2.5	46	0.310	0.3	5	0.8	17	0.333
Japan	0.3	632	9.0	15609	0.038	0.1	202	1.8	3576	0.063
Jordan	0.4	11	0.9	27	0.400	0.1	6	0.6	17	0.125
Kazakhstan	1.2	96	10.2	799	0.121	0.4	51	5.5	669	0.081
Kenya	1.2	118	20.3	2266	0.057	0.7	86	15.1	1910	0.049
Korea, South	0.3	53	7.7	1128	0.044	0.1	14	1.0	245	0.056
Korea, Republic of	0.2	92	5.0	2260	0.035	0.0	27	0.4	214	0.127
Kuwait	0.6	7	1.2	15	0.500	0.2	1	1.1	10	0.200
Kyrgyzstan	0.5	10	4.6	84	0.115	0.2	5	2.6	72	0.063
Lao PDR	0.5	11	7.7	179	0.063	0.0	0	0.7	17	0.000
Latvia	1.0	17	7.2	108	0.145	0.1	3	0.5	14	0.222
Lebanon	0.3	10	0.6	22	0.455	0.1	3	0.5	18	0.143
Lesotho	0.5	3	8.6	47	0.056	0.1	1	2.1	18	0.047
Liberia	0.0	1	0.5	5	0.091	0.0	0	0.5	7	0.000
Libya	0.5	10	1.0	21	0.455	0.0	1	0.2	5	0.143
Lithuania	1.2	28	7.1	154	0.175	0.2	6	0.5	21	0.333
Luxembourg	3.2	17	2.7	14	1.172	0.5	3	0.9	5	0.545
Macedonia	0.6	8	1.7	28	0.333	0.1	2	0.2	5	0.500
Madagascar	0.5	29	8.6	564	0.057	0.3	21	6.4	471	0.047

Malawi	1.1	46	21.0	915	0.053	0.7	38	15.5	845	0.045
Malaysia	0.8	121	1.1	177	0.667	0.3	42	0.5	82	0.500
Mali	0.1	4	1.9	90	0.071	0.1	5	1.7	91	0.050
Malta	2.2	11	1.2	6	1.769	0.1	1	0.5	4	0.167
Mauritania	0.1	1	1.1	14	0.091	0.0	0	1.1	16	0.000
Mauritius	0.3	3	4.4	39	0.071	0.1	1	1.4	17	0.048
Mexico	0.4	219	1.1	719	0.333	0.1	66	0.3	221	0.250
Mongolia	1.1	10	20.9	193	0.050	0.9	10	15.1	184	0.058
Montenegro	0.6	4	2.4	12	0.273	0.1	1	0.3	2	0.286
Morocco	0.5	79	0.9	179	0.500	0.2	28	0.8	170	0.200
Mozambique	0.3	22	6.5	437	0.051	0.3	27	6.5	570	0.045
Myanmar	0.5	111	10.3	2592	0.046	0.1	33	2.7	747	0.051
Namibia	0.1	1	2.4	14	0.045	0.0	0	0.9	7	0.000
Nepal	0.2	25	1.8	206	0.125	0.2	23	2.3	311	0.067
New Zealand	4.2	191	1.6	72	2.667	0.6	34	1.3	74	0.500
Nicaragua	0.2	6	0.8	19	0.286	0.0	0	0.2	8	0.000
Niger	0.3	5	2.8	123	0.091	0.0	0	0.2	9	0.000
Nigeria	0.0	19	0.9	370	0.051	0.0	9	0.4	171	0.053
Norway	3.3	169	2.1	108	1.533	0.4	30	0.8	43	0.571
Oman	0.3	6	0.7	11	0.500	0.2	4	1.4	11	0.160
Pakistan	0.7	497	5.8	4216	0.114	0.3	202	4.1	3038	0.073
Panama	0.6	13	1.7	39	0.333	0.1	3	0.4	12	0.400
Papua New Guinea	0.7	16	5.1	114	0.143	0.4	12	2.3	64	0.188
Paraguay	1.5	48	5.4	163	0.286	0.3	10	0.9	33	0.300
Peru	0.5	73	1.3	229	0.357	0.1	33	0.7	110	0.200
Philippines	0.7	253	1.5	566	0.500	0.1	62	0.4	178	0.250
Poland	0.6	182	3.8	1215	0.147	0.1	49	0.8	357	0.143

Portugal	1.5	147	5.4	491	0.277	0.1	16	0.3	52	0.250
Puerto Rico	1.1	31	2.8	81	0.375	0.3	11	0.5	24	0.600
Qatar	0.4	3	0.9	5	0.400	0.3	0	2.2	4	0.154
Republic of Moldova	0.8	21	3.3	87	0.259	0.2	7	0.2	13	1.000
Romania	0.7	112	3.1	487	0.229	0.1	37	0.5	96	0.250
Russian Federation	0.8	777	5.5	5508	0.143	0.1	261	0.8	1424	0.143
Rwanda	0.2	6	3.3	120	0.057	0.1	4	2.6	88	0.048
Saudi Arabia	0.4	52	0.9	112	0.438	0.1	12	1.0	86	0.143
Senegal	0.1	4	1.6	56	0.091	0.0	0	1.0	48	0.000
Serbia	0.7	49	4.0	278	0.176	0.1	12	0.8	65	0.143
Sierra Leone	0.1	2	0.8	18	0.083	0.0	0	0.9	18	0.000
Singapore	0.4	24	3.6	183	0.120	0.1	6	0.7	55	0.200
Slovakia	1.0	39	6.7	284	0.143	0.2	7	0.5	32	0.286
Slovenia	0.9	17	3.7	77	0.233	0.1	4	0.7	19	0.200
Solomon Islands	0.0	0	0.7	1	0.000	NA	NA	NA	NA	NA
Somalia	0.4	13	7.4	256	0.057	0.3	10	7.0	245	0.043
South African Republic	0.8	155	10.6	2023	0.078	0.5	155	4.9	1364	0.113
South Sudan	0.5	16	8.9	292	0.055	0.3	10	5.8	205	0.043
Spain	1.2	580	3.0	1329	0.419	0.2	114	0.5	288	0.400
Sri Lanka	0.6	83	5.4	688	0.120	0.3	45	4.0	625	0.078
Sudan	1.4	162	3.1	359	0.450	0.6	81	4.4	530	0.146
Suriname	0.3	1	0.8	2	0.333	NA	NA	NA	NA	0.200
Sweden	2.3	249	1.6	177	1.400	0.4	45	0.6	81	0.571
Switzerland	2.5	222	3.2	267	0.788	0.4	49	1.2	117	0.357
Syrian Arab Republic	0.3	18	0.7	41	0.444	0.1	6	0.6	39	0.143
Tajikistan	1.4	37	11.8	309	0.120	0.6	18	8.6	255	0.070
Tanzania	0.6	76	10.8	1396	0.057	0.3	47	6.4	997	0.052

Thailand	0.4	185	5.5	2836	0.073	0.1	49	0.8	493	0.125
The Gambia	0.1	0	0.5	4	0.100	NA	NA	NA	NA	NA
The Netherlands	7.7	1373	3.0	540	2.536	1.2	251	1.4	292	0.800
Timor-Leste	0.0	0	0.9	3	0.042	0.0	0	0.3	1	0.125
Togo	0.2	4	3.1	60	0.053	0.1	2	1.5	37	0.053
Trinidad and Tobago	0.5	4	1.7	16	0.294	NA	NA	NA	NA	NA
Tunisia	0.3	20	0.6	43	0.500	0.1	7	0.5	41	0.250
Turkey	0.5	201	1.4	583	0.355	0.1	49	1.2	637	0.071
Turkmenistan	1.2	22	10.0	191	0.122	0.5	13	7.2	182	0.072
Uganda	0.9	63	17.0	1219	0.056	0.3	22	4.9	445	0.051
Ukraine	1.1	347	5.5	1716	0.200	0.2	90	0.3	163	0.667
United Arab Emirates	0.4	8	1.1	19	0.417	0.2	1	1.0	9	0.182
United Kingdom	7.5	5146	2.6	1786	2.880	1.4	1291	2.1	1865	0.700
United States of America	3.7	10722	1.9	5289	2.000	0.4	1561	0.8	2492	0.571
Uruguay	1.7	44	5.2	135	0.318	0.4	20	1.5	59	0.286
Uzbekistan	0.5	54	4.1	448	0.125	0.2	29	3.0	408	0.064
Venezuela	0.8	106	2.3	357	0.333	0.1	28	0.5	97	0.250
Yemen	1.6	96	3.4	208	0.467	0.9	64	5.9	417	0.158
Zambia	0.4	13	7.5	235	0.058	0.1	6	3.3	135	0.042
Zimbabwe	2.9	92	10.1	314	0.289	1.4	62	10.7	452	0.127

AC adenocarcinoma; SCC, squamous cell carcinoma; N, number of cases; NA, not applicable; ASR, age-standardised rate.

Table S5. The trend analysis of esophageal cancer by country

a. **Incidence trend in individuals aged 0-85+ years**

Region	Male: AAPC (95% CI), p	Female: AAPC (95% CI), p
Asia		
Bahrain	**-15.52*(-26.15 to -3.36), p=0.020**	NA
Mainland China	**-2.97*(-3.80 to -2.13), p<0.001**	**-6.84*(-7.79 to -5.88), p<0.001**
Hong Kong, China	**-4.12*(-4.98 to -3.26), p<0.001**	**-7.34*(-10.25 to -4.34), p=0.001**
India	**-5.00*(-8.18 to -1.70), p=0.008**	-2.56 (-5.15 to 0.12), p=0.058
Israel	-2.65 (-5.39 to 0.16), p=0.061	-0.97 (-5.63 to 3.91), p=0.652
Japan	**1.17*(0.41 to 1.94), p=0.007**	**2.18*(0.58 to 3.80), p=0.014**
South Korea	**-1.46*(-2.64 to -0.25), p=0.018**	-0.27 (-3.31 to 2.87), p=0.845
Kuwait	7.45 (-4.15 to 20.46), p=0.185	-1.37 (-14.71 to 14.05), p=0.832
Philippines	-4.35 (-8.83 to 0.36), p=0.065	**-7.20*(-12.21 to -1.90), p=0.015**
Thailand	**2.17*(0.74 to 3.62), p=0.008**	1.80 (-7.89 to 4.70), p=0.532
Oceania		
Australia	0.54 (-0.17 to 1.26), p=0.119	-1.23 (-2.63 to 0.20), p=0.082
New Zealand	-0.46 (-2.42 to 1.53), p=0.603	-0.54 (-4.05 to 3.10), p=0.736
Northern America		
Canada	**1.51*(0.54 to 2.49), p=0.007**	-0.21 (-1.88 to 1.49), p=0.782
United States	**-0.97*(-1.60 to -0.33), p=0.008**	-0.53 (-2.54 to 1.51), p=0.561
Southern America		
Brazil	**-5.94*(-10.44 to -1.21), p=0.021**	-8.78*(-14.32 to -2.89), p=0.010
Chile	-1.72 (-6.94 to 3.80), p=0.534	-7.77 (-16.01 to 1.27), p=0.081
Colombia	**-9.14*(-12.96 to -5.16), p=0.001**	1.12 (-11.23 to 15.2), p=0.848
Costa Rica	-2.30 (-5.36 to 0.86), p=0.131	-0.27 (-7.34 to 7.33), p=0.934
Ecuador	-4.69 (-15.02 to 6.89), p=0.362	0.73 (-8.11 to 10.43), p=0.859

Northern Europe		
Denmark	1.15 (-2.19 to 4.60), p=0.506	1.31 (-0.90 to 3.55), p=0.210
Estonia	2.06 (-2.01 to 6.30), p=0.281	4.19 (-3.11 to 12.04), p=0.229
Faroe Islands	17.36 (-1.97 to 40.49), p=0.074	NA
Finland	1.37 (-0.16 to 2.93), p=0.073	1.04 (-3.40 to 5.69), p=0.609
Greenland	-0.05 (-18.94 to 23.26), p=0.996	-2.16(-15.46 to 13.23), p=0.739
Iceland	-5.07 (-10.52 to 0.73), p=0.078	2.16 (-11.73 to 18.23), p=0.745
Ireland	-0.85 (-2.70 to 1.04), p=0.329	-1.23 (-2.71 to 0.28), p=0.097
Lithuania	1.23 (-1.13 to 3.63), p=0.266	4.39 (-0.39 to 9.39), p=0.067
Norway	**3.10*(1.23 to 5.00), p=0.005**	1.80 (-0.22 to 3.86), p=0.074
Sweden	0.83 (-1.22 to 2.92), p=0.381	-0.07 (-2.56 to 2.49), p=0.950
United Kingdom	**0.85*(0.44 to 1.26), p=0.001**	-0.19 (-0.69 to 0.31), p=0.401
Western Europe		
Austria	-1.27 (-2.55 to 0.03), p=0.055	2.18 (-1.86 to 6.39), p=0.252
Belarus	1.09*(0.00 to 2.20), p=0.050	1.27 (-4.61 to 7.51), p=0.640
France	**-2.57*(-3.68 to -1.44), p=0.001**	0.96 (-2.19 to 4.20), p=0.508
Germany	0.76 (-1.40 to 2.97), p=0.443	2.49 (-1.19 to 6.30), p=0.159
Netherlands	**2.11*(1.25 to 2.98), p<0.001**	**1.88*(0.51 to 3.27), p=0.013**
Switzerland	-2.26*(-4.03 to -0.47), p=0.020	2.14 (-1.61 to 6.03), p=0.228
Southern Europe		
Bulgaria	2.17 (-0.24 to 4.64), p=0.072	1.01 (-2.79 to 4.97), p=0.562
Croatia	-2.14 (-4.32 to 0.09), p=0.057	1.21 (-5.81 to 8.76), p=0.709
Cyprus	2.81 (-12.83 to 21.25), p=0.709	NA
Italy	**-3.02*(-5.77 to -0.20), p=0.036**	-1.21 (-3.96 to 1.63), p=0.352
Malta	1.96 (-7.48 to 12.36), p=0.657	-0.54 (-23.51 to 29.32), p=0.968
Slovenia	**-3.53*(-6.38 to -0.58), p=0.025**	-0.75 (-6.98 to 5.89), p=0.795
Spain	**-2.16*(-3.46 to -0.85), p=0.005**	**3.41*(0.90 to 5.99), p=0.014**

Turkey	-3.96 (-7.83 to 0.08), p=0.054	-1.54 (-4.88 to 1.91), p=0.329
Eastern Europe		
Czech Republic	1.27 (-0.25 to 2.81), p=0.091	**4.66*(0.47 to 9.03), p=0.033**
Poland	0.33 (-4.01 to 4.87), p=0.868	2.40 (-12.89 to 20.37), p=0.744
Slovakia	-0.55 (-2.43 to 1.36), p=0.568	-0.48 (-5.41 to 4.70), p=0.831
Africa		
Uganda	4.36 (-0.27 to 9.20), p=0.062	0.02 (-7.28 to 7.90), p=0.995

AAPC, annual percentage change; CI, confidence interval; NA, not applicable; * p values less than 0.05.

b. Mortality trend in individuals aged 0-85+ years

Region	Male: AAPC (95% CI), p	Female: AAPC (95% CI), p
Asia		
Bahrain	-13.68(-26.96 to 2.02), p=0.077	-6.13(-16.32 to 5.31), p=0.240
Hong Kong, China	**-4.91*(-5.73 to -4.08), p<0.001**	-5.86*(-8.79 to -2.84), p=0.002
Israel	-2.02 (-4.53 to 0.55), p=0.107	-0.94 (-4.08 to 2.30), p=0.517
Japan	**-2.60*(-2.98 to -2.21), p<0.001**	NA
South Korea	-4.21*(-5.19 to -3.22), p<0.001	-3.36 (-7.96 to 1.47), p=0.145
Kuwait	-3.98 (-15.43 to 9.02), p=0.482	-7.88 (-23.12 to 10.38), p=0.326
Philippines	1.96 (-0.54 to 4.51), p=0.109	-2.20 (-6.20 to 1.98), p=0.256
Singapore	-2.66 (-6.52 to 1.36), p=0.163	-0.86 (-4.21 to 2.60), p=0.576
Thailand	**5.24*(4.76 to 5.72), p<0.001**	NA
Oceania		

Australia	-1.49*(-2.25 to -0.72), p=0.002	-2.00 (-3.99 to 0.03), p=0.053
New Zealand	-0.41 (-2.57 to 1.79), p=0.674	-1.01 (-3.43 to 1.46), p=0.370
Northern America		
Canada	-0.40 (-1.29 to 0.50), p=0.335	-0.07 (-1.55 to 1.44), p=0.917
United States	**-1.14*(-1.45 to -0.83), p<0.001**	-1.21*(-2.36 to -0.04), p=0.044
Southern America		
Brazil	**-1.41*(-1.75 to -1.06), p<0.001**	**-2.14*(-2.58 to -1.70), p<0.001**
Chile	**-5.05*(-5.89 to -4.21), p<0.001**	-2.84*(-4.35 to -1.30), p=0.003
Colombia	**-3.88*(-5.67 to -2.06), p<0.001**	-4.87*(-7.12 to -2.57), p=0.001
Costa Rica	-3.34 (-7.27 to 0.76), p=0.096	-0.79 (-6.87 to 5.68), p=0.780
Ecuador	-0.87 (-5.47 to 3.96), p=0.684	-2.75 (-6.51 to 1.17), p=0.142
Northern Europe		
Denmark	-0.14 (-2.33 to 2.09), p=0.885	-0.50 (-4.30 to 3.44), p=0.772
Estonia	0.91 (-3.72 to 5.77), p=0.668	8.59 (-1.01 to 19.11), p=0.074
Faroe Islands	3.84 (-9.88 to 19.64), p=0.557	NA
Finland	0.80 (-1.87 to 3.55), p=0.513	-1.25 (-4.63 to 2.25), p=0.429
Greenland	-4.15(-13.40 to 6.09), p=0.364	-1.46(-13.72 to 12.55), p=0.805
Iceland	-2.50 (-8.69 to 4.11), p=0.399	2.58 (-8.74 to 15.30), p=0.629
Ireland	-0.06 (-1.94 to 1.86), p=0.947	-1.85 (-3.80 to 0.14), p=0.064
Latvia	2.33*(0.45 to 4.25), p=0.021	0.42 (-3.85 to 4.88), p=0.830
Lithuania	0.01 (-1.79 to 1.84), p=0.995	0.67 (-4.52 to 6.14), p=0.778
Norway	0.33 (-1.63 to 2.33), p=0.712	4.47 (-3.85 to 13.51), p=0.302
Sweden	0.79 (-0.45 to 2.04), p=0.18	-0.51 (-3.46 to 2.54), p=0.708
United Kingdom	**-0.99*(-1.38 to -0.60), p<0.001**	**-1.73*(-2.24 to -1.23), p<0.001**
Western Europe		
Austria	-1.60*(-2.73 to -0.47), p=0.012	3.67*(0.76 to 6.67), p=0.019
Belgium	0.01 (-1.07 to 1.10), p=0.983	0.63 (-0.79 to 2.07), p=0.340

France	**-3.32*(-3.71 to -2.93), p<0.001**	0.07 (-1.72 to 1.89), p=0.928
Germany	0.25 (-0.43 to 0.94), p=0.425	0.00 (-0.98 to 0.99), p=1.000
Netherlands	-0.14 (-0.64 to 0.37), p=0.542	-0.26 (-0.83 to 0.31), p=0.316
Switzerland	-1.05*(-1.79 to -0.31), p=0.011	-1.37 (-3.52 to 0.82), p=0.185
Southern Europe		
Bulgaria	-0.97*(-1.85 to -0.08), p=0.037	0.25 (-4.32 to 5.04), p=0.904
Croatia	-2.78*(-5.01 to -0.50), p=0.023	-3.32 (-8.83 to 2.52), p=0.221
Cyprus	-4.17 (-13.84 to 6.59), p=0.383	NA
Italy	-1.84*(-2.63 to -1.05), p=0.001	-0.33 (-3.88 to 3.36), p=0.860
Malta	6.71 (-9.80 to 26.23), p=0.399	-1.63 (-12.58 to 10.69), p=0.756
Portugal	1.12*(0.24 to 2.01), p=0.018	-2.89 (-6.24 to 0.57), p=0.089
Slovenia	-2.50 (-5.66 to 0.75), p=0.113	-1.37 (-5.78 to 3.24), p=0.506
Spain	-1.93*(-2.74 to -1.10), p=0.001	-0.19 (-3.74 to 3.50), p=0.909
Eastern Europe		
Slovakia	-2.09*(-3.75 to -0.40), p=0.022	-2.83 (-8.56 to 3.26), p=0.307
Czech Republic	0.18 (-1.71 to 2.11), p=0.851	2.04 (-1.39 to 5.59), p=0.210
Poland	-2.17*(-3.23 to -1.11), p=0.002	-0.08 (-1.96 to 1.83), p=0.924
Russian Federation	-0.97*(-1.45 to -0.48), p=0.002	-1.14*(-2.25 to -0.02), p=0.046
Belarus	-0.28(-1.66 to 1.13), p=0.661	-2.42(-7.59 to 3.03), p=0.329

AAPC, annual percentage change; CI, confidence interval; NA, not applicable; * p values less than 0.05.

c. **Incidence trend in individuals aged ≥ 50 years**

Region	Male: AAPC (95% CI), p	Female: AAPC (95% CI), p
Asia		
Bahrain	-1.60 (-19.77 to 20.69), p=0.877	NA
Mainland China	**-2.92*(-3.80 to -2.02), p<0.001**	**-6.87*(-7.97 to -5.76), p<0.001**
Hong Kong, China	**-4.12*(-5.22 to -3.00), p<0.001**	**-7.84*(-10.67 to -4.92), p<0.001**
India	-4.51*(-7.31 to -1.63), p=0.007	-1.87 (-4.17 to 0.49), p=0.105
Israel	-2.76 (-5.49 to 0.04), p=0.053	-1.07 (-4.08 to 2.02), p=0.443
Japan	1.35*(0.55 to 2.16), p=0.005	2.47*(1.02 to 3.93), p=0.004
South Korea	-1.56*(-2.47 to -0.63), p=0.001	0.05 (-3.29 to 3.49), p=0.976
Kuwait	6.90 (-8.66 to 25.11), p=0.357	-1.69 (-14.64 to 13.22), p=0.788
Philippines	-3.30 (-7.35 to 0.92), p=0.108	-9.25*(-14.33 to -3.87), p=0.005
Thailand	0.98 (-0.75 to 2.74), p=0.228	-2.27 (-7.40 to 3.15), p=0.356
Oceania		
Australia	0.53(-0.02 to 1.10), p=0.058	-1.16 (-2.41 to 0.11), p=0.069
New Zealand	-0.39 (-2.19 to 1.44), p=0.633	-1.43 (-5.23 to 2.53), p=0.425
Northern America		
Canada	1.57*(0.54 to 2.60), p=0.008	0.19 (-1.39 to 1.81), p=0.786
United States	-0.86*(-1.66 to -0.06), p=0.039	-0.42 (-2.25 to 1.44), p=0.613
Southern America		
Brazil	-6.05*(-10.66 to -1.20), p=0.021	-6.78 (-16.06 to 3.52), p=0.189
Chile	-3.40 (-10.62 to 4.40), p=0.382	-7.44 (-15.90 to 1.86), p=0.100
Colombia	-8.69*(-12.23 to -5.01), p=0.001	-0.92 (-13.95 to 14.09), p=0.884
Costa Rica	-1.78 (-5.22 to 1.78), p=0.278	-0.89 (-7.99 to 6.75), p=0.787
Ecuador	0.34 (-19.61 to 25.23), p=0.976	-2.69 (-12.36 to 8.05), p=0.565
Northern Europe		
Denmark	1.84 (-1.86 to 5.68), p=0.334	1.78 (-0.39 to 4.01), p=0.096

Estonia	2.28 (-1.49 to 6.19), p=0.204	4.92 (-4.61 to 15.40), p=0.278
Faroe Islands	19.22*(1.17 to 40.49), p=0.039	NA
Finland	1.28 (-0.04 to 2.61), p=0.055	0.54 (-4.06 to 5.37), p=0.797
Greenland	-0.16 (-19.03 to 23.1), p=0.986	-2.26(-15.26 to 12.73), p=0.722
Iceland	-4.75 (-10.71 to 1.61), p=0.120	3.56 (-9.21 to 18.12), p=0.557
Ireland	-0.91 (-2.42 to 0.62), p=0.206	-1.03 (-2.98 to 0.96), p=0.265
Lithuania	1.22 (-1.23 to 3.72), p=0.287	1.70 (-3.22 to 6.87), p=0.456
Norway	**2.77*(0.86 to 4.73), p=0.010**	1.80 (-0.18 to 3.82), p=0.070
Sweden	0.85 (-1.59 to 3.35), p=0.449	0.64 (-2.12 to 3.48), p=0.610
United Kingdom	**0.96*(0.60 to 1.33), p<0.001**	-0.11 (-0.62 to 0.40), p=0.634
Western Europe		
Austria	-0.97 (-2.66 to 0.74), p=0.225	1.48 (-1.54 to 4.58), p=0.295
Belarus	1.06 (-0.12 to 2.25), p=0.071	0.46 (-5.44 to 6.74), p=0.865
France	-2.12*(-3.50 to -0.72), p=0.008	1.48 (-1.26 to 4.29), p=0.250
Germany	0.94 (-1.21 to 3.13), p=0.347	2.40 (-0.58 to 5.46), p=0.101
Netherlands	2.29*(1.30 to 3.30), p=0.001	2.22*(0.94 to 3.51), p=0.004
Switzerland	-2.13 (-4.42 to 0.21), p=0.069	2.28 (-1.65 to 6.35), p=0.221
Southern Europe		
Bulgaria	2.26 (-0.22 to 4.81), p=0.069	1.50 (-2.80 to 5.99), p=0.451
Croatia	-2.09*(-4.06 to -0.08), p=0.044	6.25 (-8.34 to 23.15), p=0.421
Cyprus	2.39 (-14.97 to 23.29), p=0.777	NA
Italy	-3.33*(-4.90 to -1.74), p=0.001	-1.01 (-3.86 to 1.93), p=0.447
Malta	1.93 (-8.23 to 13.21), p=0.686	NA
Slovenia	-3.25*(-6.28 to -0.12), p=0.044	-0.28 (-5.33 to 5.05), p=0.905
Spain	-1.70*(-2.70 to -0.69), p=0.005	4.14*(0.47 to 7.93), p=0.031
Turkey	-2.78*(-5.13 to -0.38), p=0.029	-0.75 (-4.80 to 3.47), p=0.688
Eastern Europe		

Czech Republic	1.80*(0.37 to 3.24), p=0.020	4.90*(2.44 to 7.42), p=0.002
Poland	1.32 (-3.74 to 6.65), p=0.571	0.96 (-12.81 to 16.9), p=0.885
Slovakia	0.36 (-1.19 to 1.93), p=0.652	0.47 (-4.39 to 5.58), p=0.831
Africa		
Uganda	**4.57*(0.09 to 9.25), p=0.047**	-0.51 (-8.28 to 7.92), p=0.888

AAPC, annual percentage change; CI, confidence interval; NA, not applicable; * p values less than 0.05.

d. Incidence trend in individuals aged < 50 years

Region	Male: AAPC (95% CI), p	Female: AAPC (95% CI), p
Asia		
Bahrain	NA	NA
Mainland China	-3.60*(-6.53 to -0.58), p=0.026	-7.67*(-13.26 to -1.73), p=0.018
Hong Kong, China	-3.77 (-8.60 to 1.32), p=0.124	**-5.86*(-5.86 to -5.86), p<0.001**
India	-11.07 (-26.08 to 6.98), p=0.213	-5.36 (-11.34 to 1.02), p=0.087
Israel	0.42 (-9.67 to 11.64), p=0.929	NA
Japan	-2.66 (-7.64 to 2.60), p=0.271	4.27 (-2.92 to 11.98), p=0.214
South Korea	2.24 (-2.71 to 7.43), p=0.334	NA
Kuwait	NA	NA
Philippines	-12.28 (-27.23 to 5.74), p=0.145	NA
Thailand	12.01*(5.57 to 18.84), p=0.002	NA
Oceania		

Australia	-0.10 (-3.71 to 3.65), p=0.953	0.42 (-5.33 to 6.52), p=0.873
New Zealand	-0.28 (-11.79 to 12.73), p=0.959	8.40 (-2.85 to 20.95), p=0.128
Northern America		
Canada	2.26 (-0.92 to 5.55), p=0.142	0.00 (0.00 to 0.00), p=0.235
United States	-0.41 (-3.24 to 2.51), p=0.754	0.00 (0.00 to 0.00), p=0.235
Southern America		
Brazil	-5.91 (-13.44 to 2.29), p=0.131	NA
Chile	NA	NA
Colombia	-12.29 (-23.97 to 1.19), p=0.067	NA
Costa Rica	-0.34 (-13.70 to 15.08), p=0.958	NA
Ecuador	NA	NA
Northern Europe		
Denmark	-9.79 (-19.09 to 0.58), p=0.061	-3.85 (-13.80 to 7.26), p=0.432
Estonia	-3.05 (-12.83 to 7.83), p=0.521	NA
Faroe Islands	NA	NA
Finland	-2.07 (-10.55 to 7.22), p=0.609	-0.84 (-8.31 to 7.25), p=0.811
Greenland	NA	NA
Iceland	NA	NA
Ireland	1.47 (-6.59 to 10.23), p=0.695	-3.79 (-15.56 to 9.63), p=0.514
Lithuania	0.96 (-1.81 to 3.81), p=0.451	NA
Norway	6.79*(0.10 to 13.93), p=0.047	NA
Sweden	-3.84 (-12.95 to 6.22), p=0.391	2.12 (-3.49 to 8.06), p=0.416
United Kingdom	-0.57 (-2.82 to 1.75), p=0.585	-5.50*(-8.70 to -2.18), p=0.005
Western Europe		
Austria	-2.57 (-11.36 to 7.09), p=0.543	NA
Belarus	0.62 (-2.39 to 3.72), p=0.650	NA
France	-6.40*(-9.15 to -3.56), p=0.001	-7.00 (-16.07 to 3.06), p=0.142

Germany	-1.54 (-8.48 to 5.94), p=0.639	1.59 (-10.95 to 15.89), p=0.789
Netherlands	0.22 (-3.16 to 3.71), p=0.888	-2.26 (-10.25 to 6.45), p=0.554
Switzerland	-5.78 (-15.52 to 5.09), p=0.244	NA
Southern Europe		
Bulgaria	2.88 (-2.66 to 8.74), p=0.270	NA
Croatia	-0.22 (-4.53 to 4.29), p=0.911	NA
Cyprus	NA	NA
Italy	-5.29 (-12.49 to 2.50), p=0.152	-2.08 (-10.37 to 6.99), p=0.599
Malta	NA	NA
Slovenia	-3.53 (-15.77 to 10.49), p=0.558	NA
Spain	-6.56*(-10.13 to -2.86), p=0.004	-1.25 (-10.46 to 8.90), p=0.774
Turkey	-7.21*(-13.70 to -0.24), p=0.044	-4.68 (-12.29 to 3.59), p=0.220
Eastern Europe	NA	NA
Czech Republic	-3.22 (-8.71 to 2.59), p=0.231	NA
Poland	-6.66 (-22.21 to 12.02), p=0.409	NA
Slovakia	-7.45*(-12.02 to -2.63), p=0.008	2.30 (-8.67 to 14.58), p=0.656
Africa	NA	NA
Uganda	2.92 (-3.34 to 9.585), p=0.321	8.99(-0.75 to 19.68), p=0.067

AAPC, annual percentage change; CI, confidence interval; NA, not applicable; * p values less than 0.05.

Figure S1. The incidence and mortality trends of esophageal cancer

Male

Asia

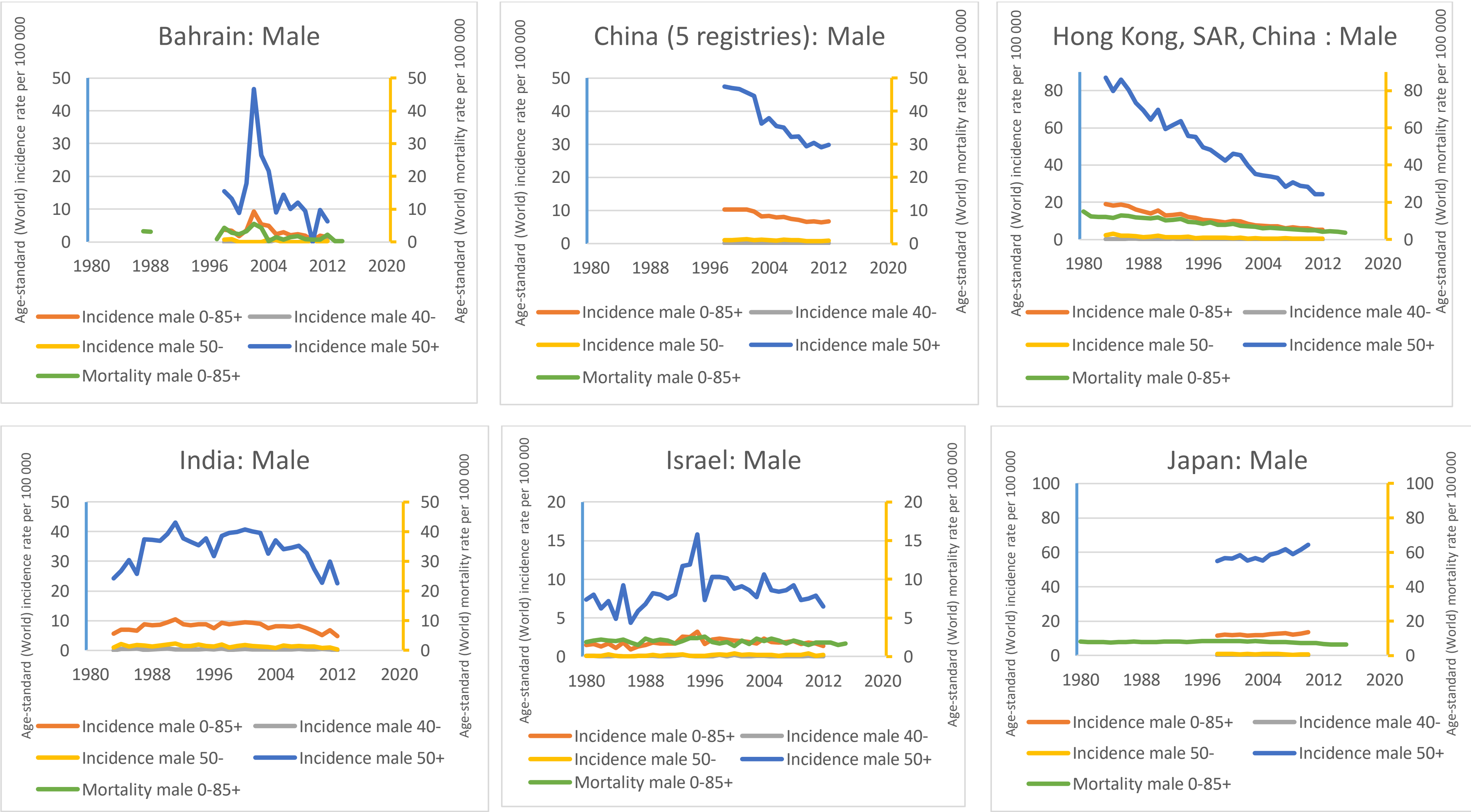

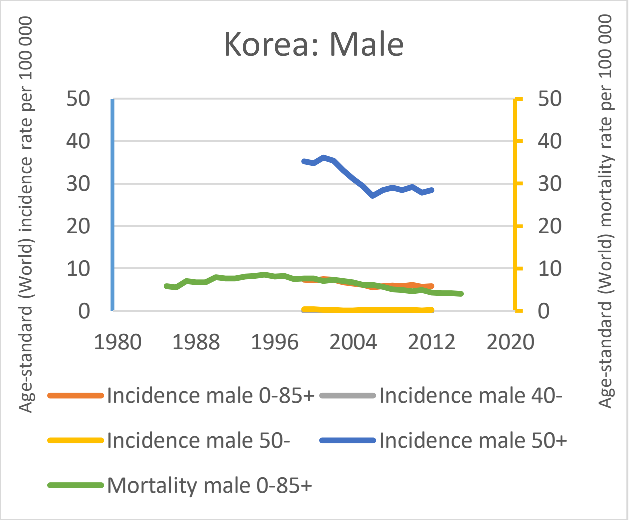
Korea: Male
Age-standard (World) incidence rate per 100 000
Age-standard (World) mortality rate per 100 000
50
40
30
20
10
0
1980 1988 1996 2004 2012 2020
Incidence male 0-85+
Incidence male 40-
Incidence male 50-
Incidence male 50+
Mortality male 0-85+

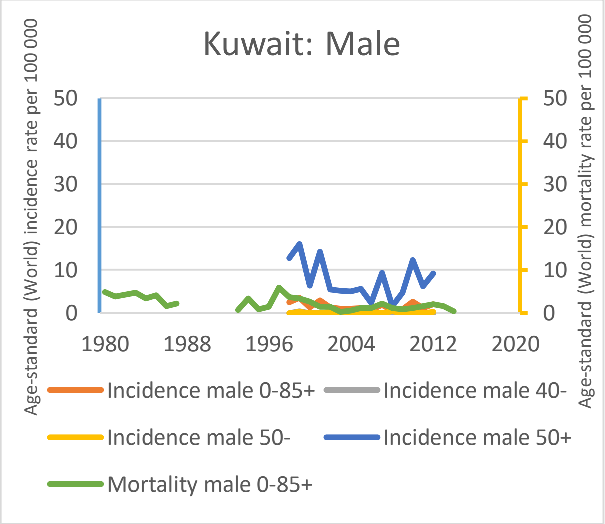
Kuwait: Male
Age-standard (World) incidence rate per 100 000
Age-standard (World) mortality rate per 100 000
50
40
30
20
10
0
1980 1988 1996 2004 2012 2020
Incidence male 0-85+
Incidence male 40-
Incidence male 50-
Incidence male 50+
Mortality male 0-85+

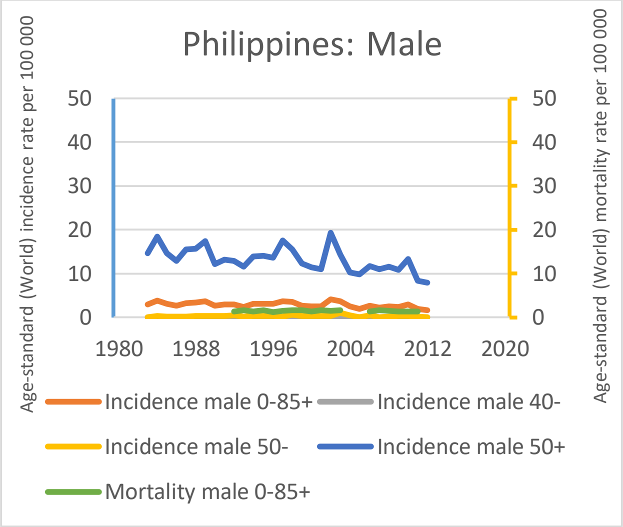
Philippines: Male
Age-standard (World) incidence rate per 100 000
Age-standard (World) mortality rate per 100 000
50
40
30
20
10
0
1980 1988 1996 2004 2012 2020
Incidence male 0-85+
Incidence male 40-
Incidence male 50-
Incidence male 50+
Mortality male 0-85+

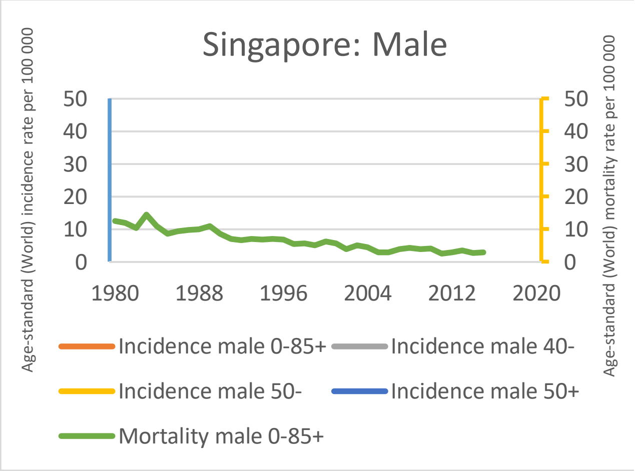
Singapore: Male
Age-standard (World) incidence rate per 100 000
Age-standard (World) mortality rate per 100 000
50
40
30
20
10
0
1980 1988 1996 2004 2012 2020
Incidence male 0-85+
Incidence male 40-
Incidence male 50-
Incidence male 50+
Mortality male 0-85+

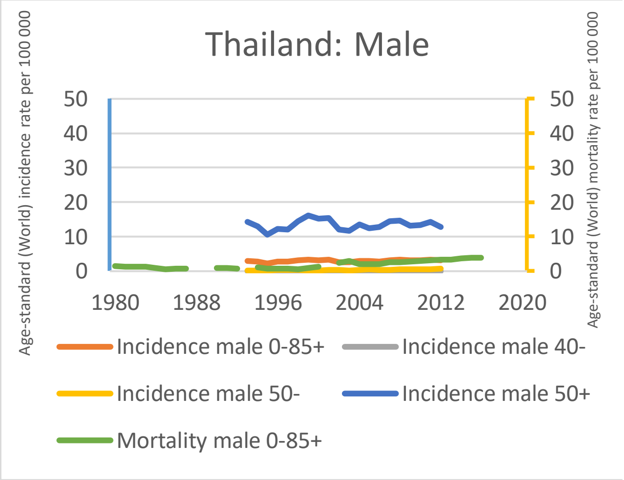
Thailand: Male
Age-standard (World) incidence rate per 100 000
Age-standard (World) mortality rate per 100 000
50
40
30
20
10
0
1980 1988 1996 2004 2012 2020
Incidence male 0-85+
Incidence male 40-
Incidence male 50-
Incidence male 50+
Mortality male 0-85+

Oceania

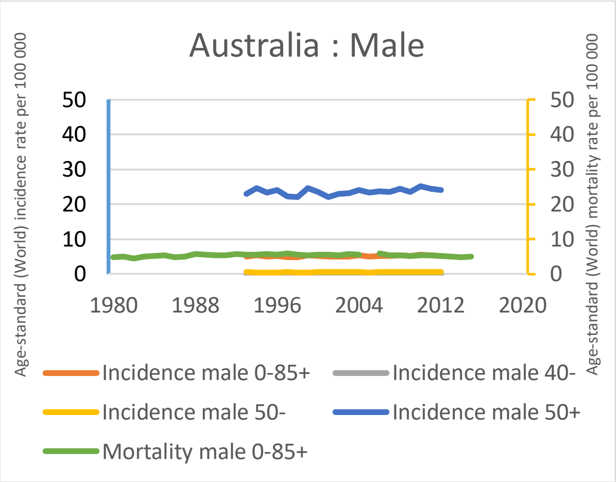

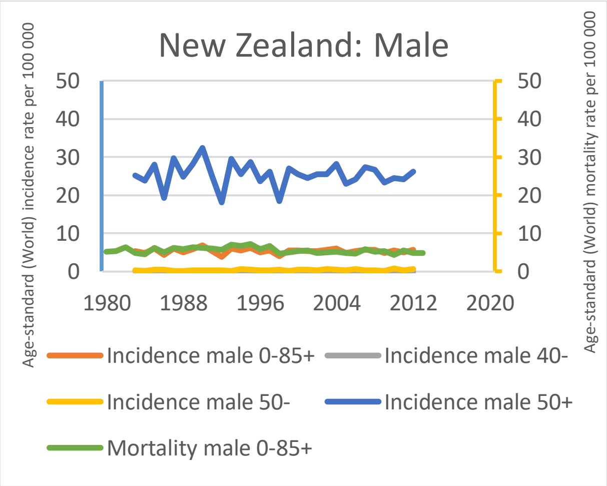

Northern America

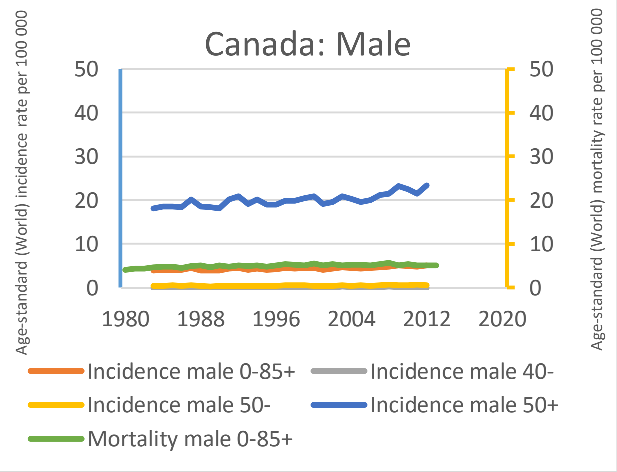

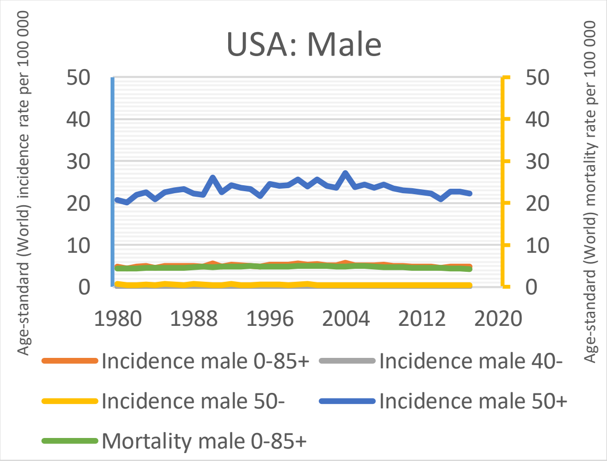

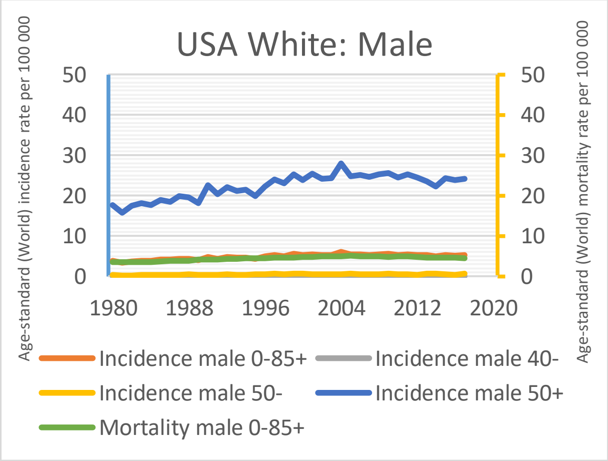

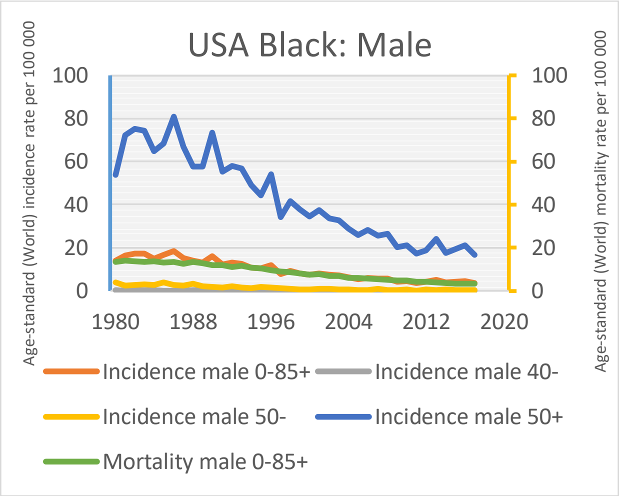

Southern America

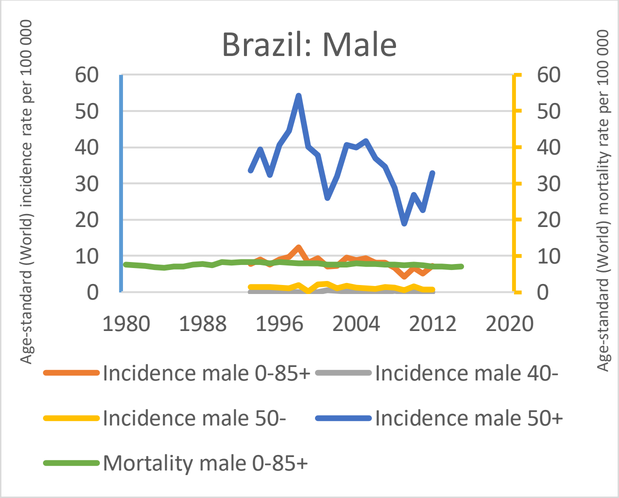

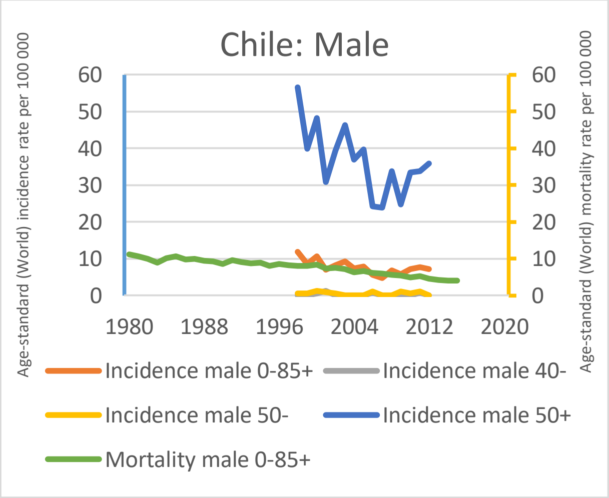

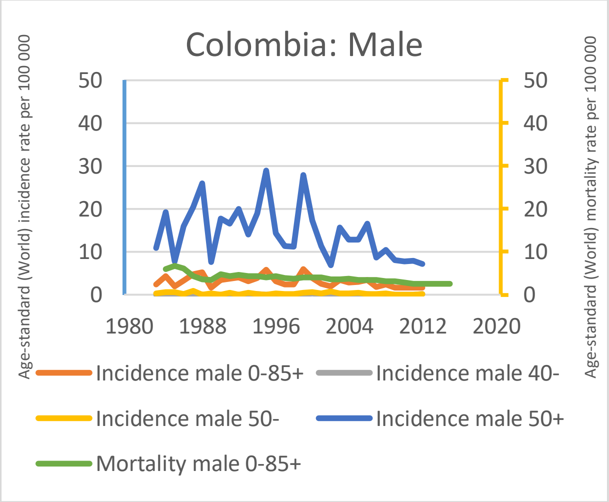

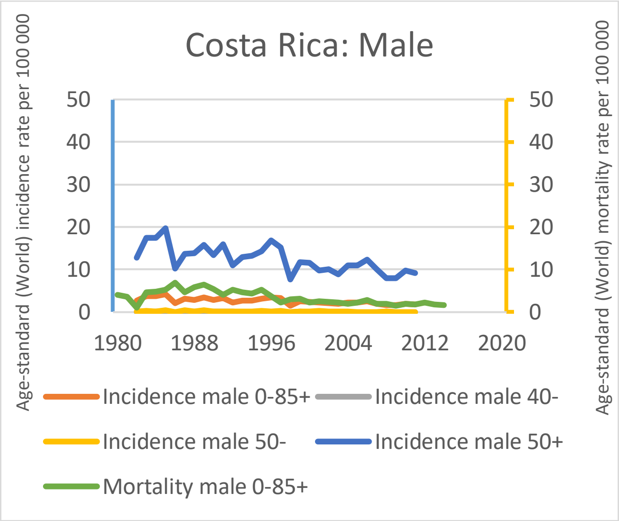

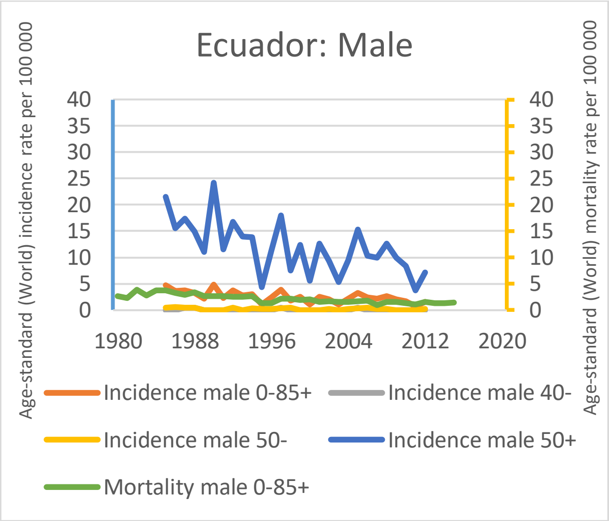

Northern Europe

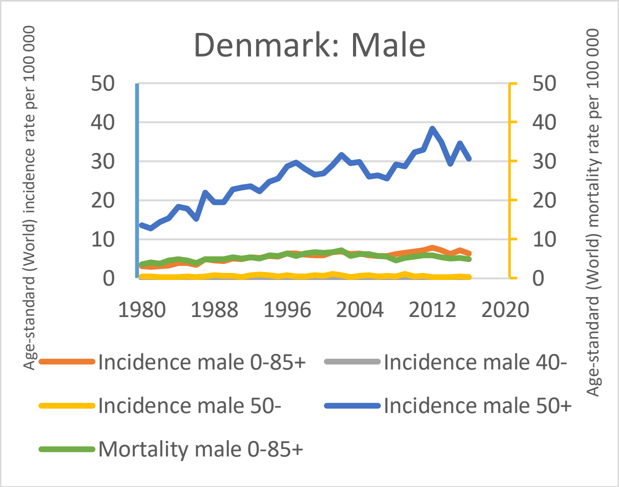

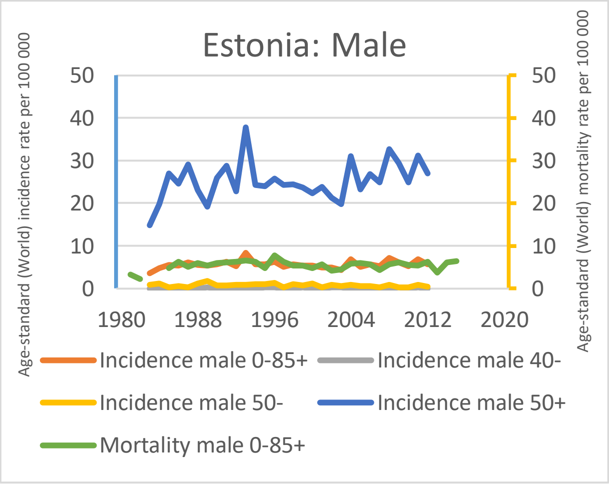

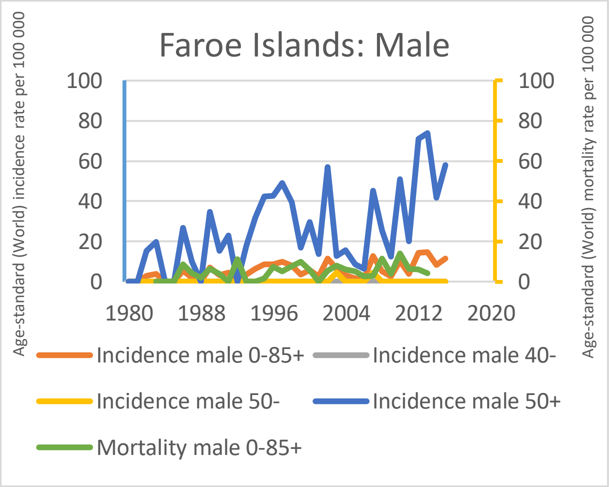

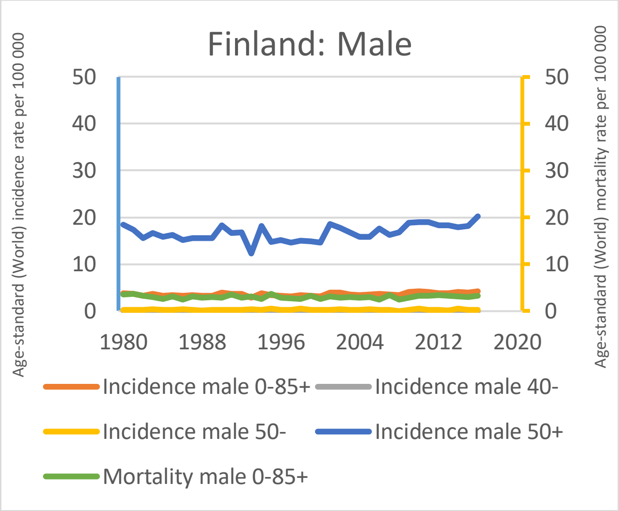
Finland: Male
Age-standard (World) incidence rate per 100 000
Age-standard (World) mortality rate per 100 000
50
40
30
20
10
0
1980
1988
1996
2004
2012
2020
Incidence male 0-85+
Incidence male 40-
Incidence male 50-
Incidence male 50+
Mortality male 0-85+

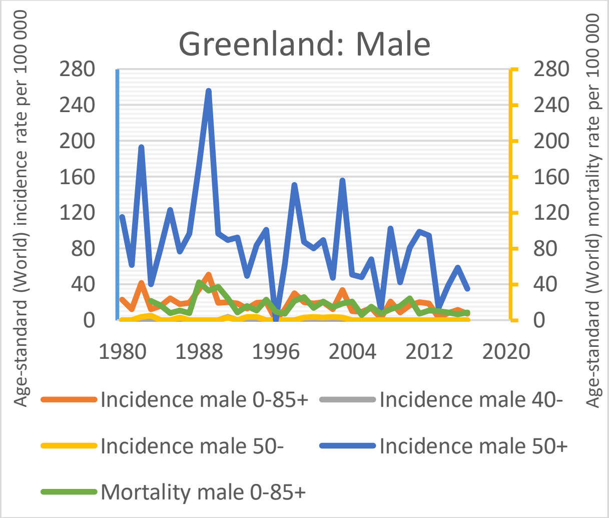
Greenland: Male
Age-standard (World) incidence rate per 100 000
Age-standard (World) mortality rate per 100 000
280
240
200
160
120
80
40
0
1980
1988
1996
2004
2012
2020
Incidence male 0-85+
Incidence male 40-
Incidence male 50-
Incidence male 50+
Mortality male 0-85+

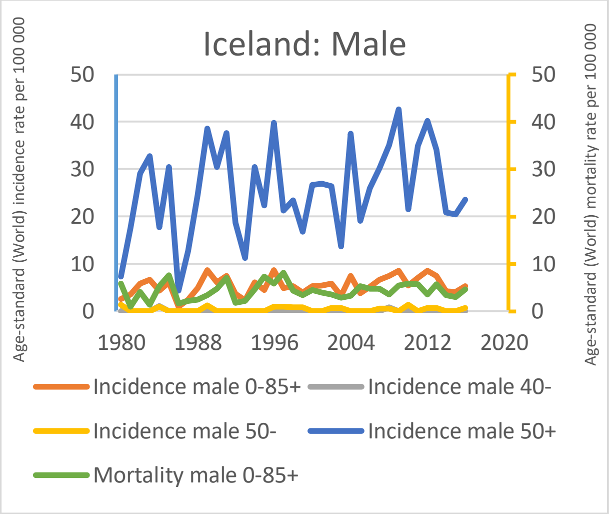
Iceland: Male
Age-standard (World) incidence rate per 100 000
Age-standard (World) mortality rate per 100 000
50
40
30
20
10
0
1980
1988
1996
2004
2012
2020
Incidence male 0-85+
Incidence male 40-
Incidence male 50-
Incidence male 50+
Mortality male 0-85+

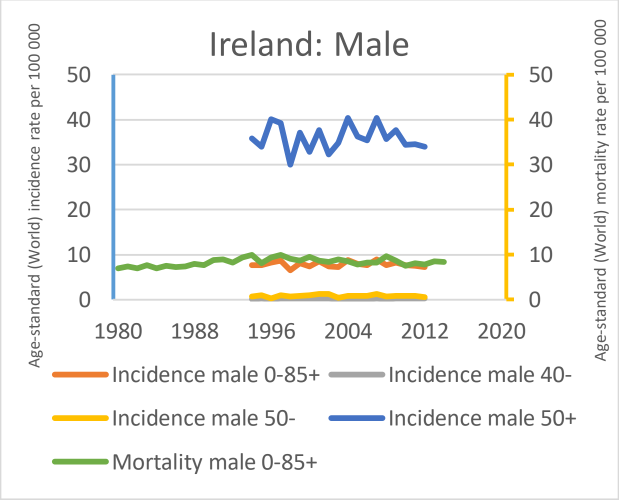
Ireland: Male
Age-standard (World) incidence rate per 100 000
Age-standard (World) mortality rate per 100 000
50
40
30
20
10
0
1980
1988
1996
2004
2012
2020
Incidence male 0-85+
Incidence male 40-
Incidence male 50-
Incidence male 50+
Mortality male 0-85+

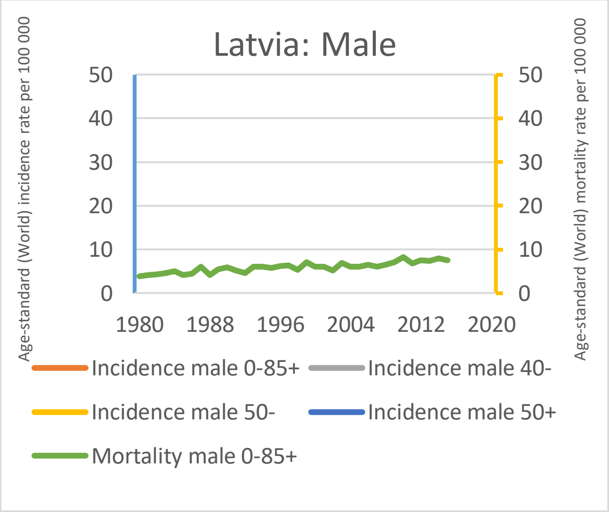
Latvia: Male
Age-standard (World) incidence rate per 100 000
Age-standard (World) mortality rate per 100 000
50
40
30
20
10
0
1980
1988
1996
2004
2012
2020
Incidence male 0-85+
Incidence male 40-
Incidence male 50-
Incidence male 50+
Mortality male 0-85+

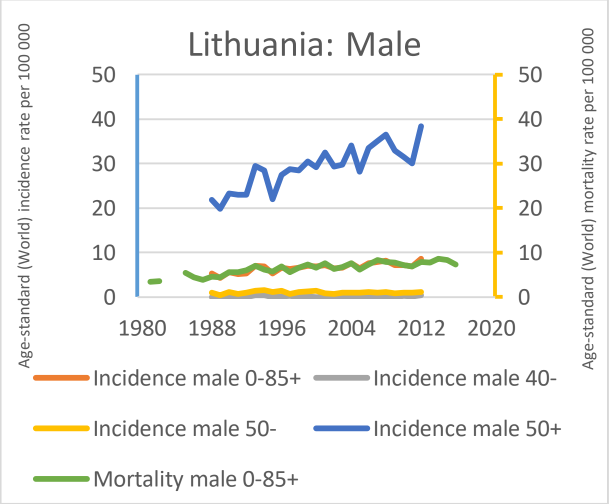
Lithuania: Male
Age-standard (World) incidence rate per 100 000
Age-standard (World) mortality rate per 100 000
50
40
30
20
10
0
1980
1988
1996
2004
2012
2020
Incidence male 0-85+
Incidence male 40-
Incidence male 50-
Incidence male 50+
Mortality male 0-85+

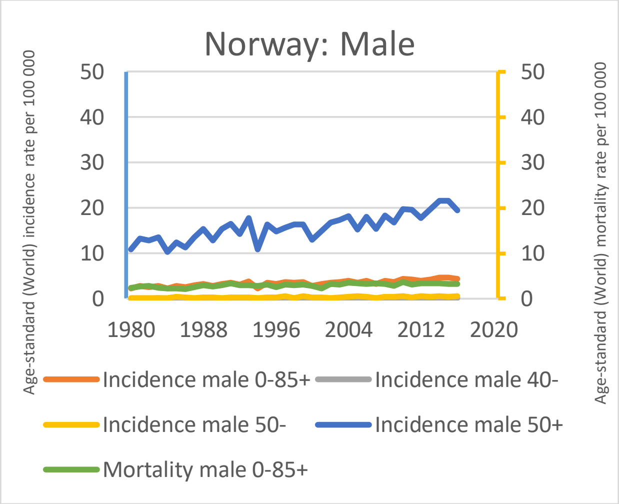

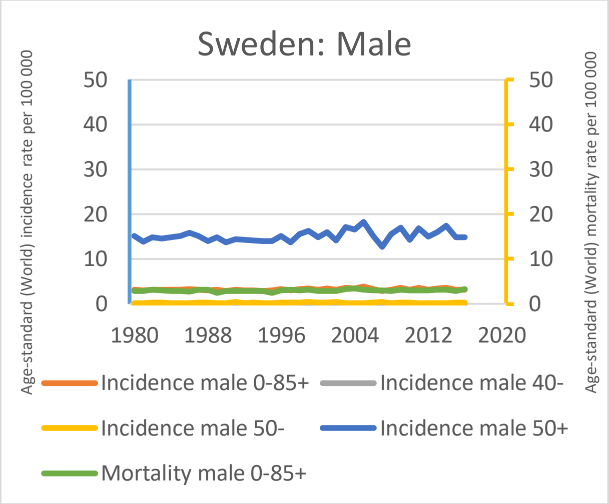

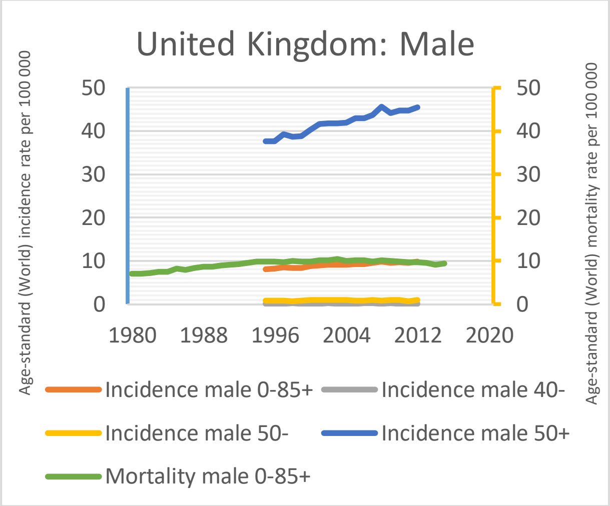

Western Europe

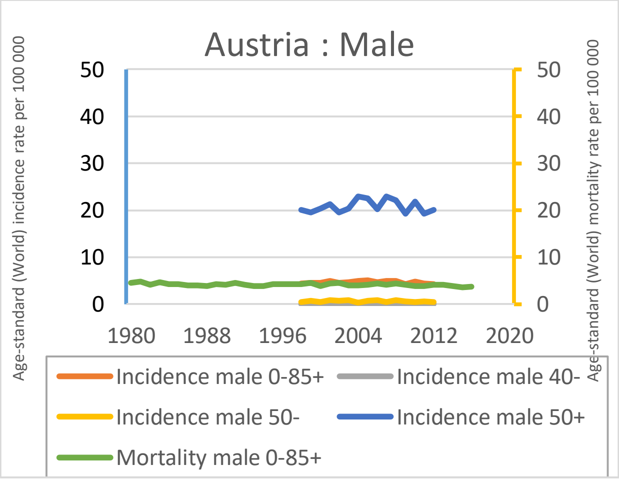

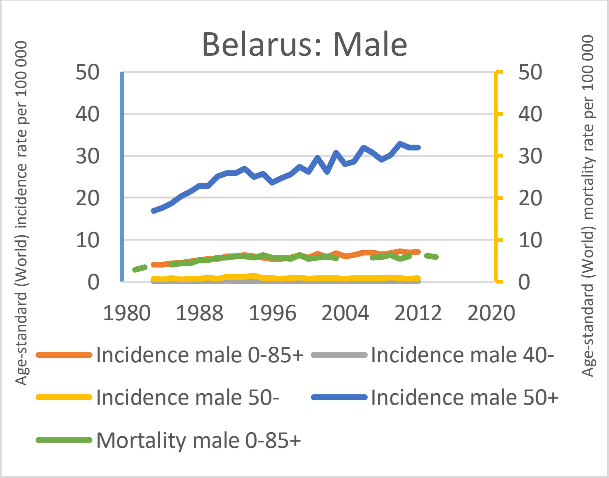

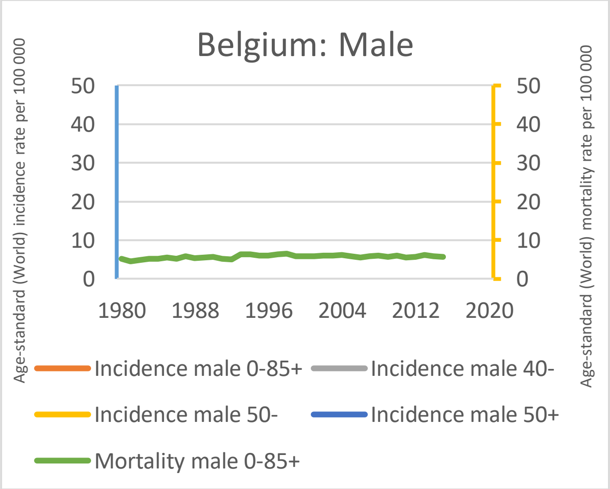

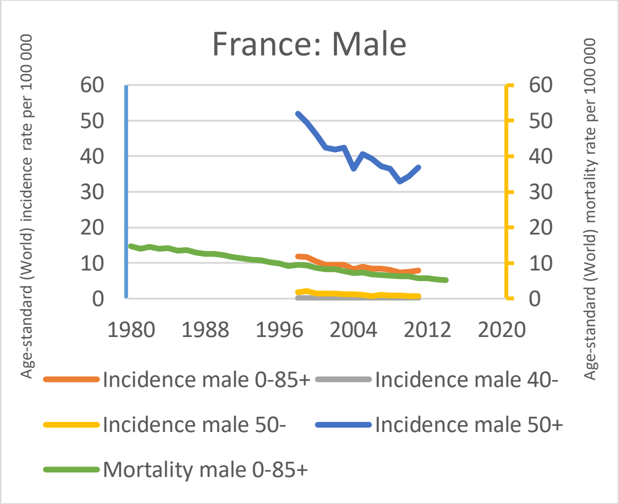
France: Male
Age-standard (World) incidence rate per 100 000
Age-standard (World) mortality rate per 100 000
60
50
40
30
20
10
0
1980
1988
1996
2004
2012
2020
Incidence male 0-85+
Incidence male 40-
Incidence male 50-
Incidence male 50+
Mortality male 0-85+

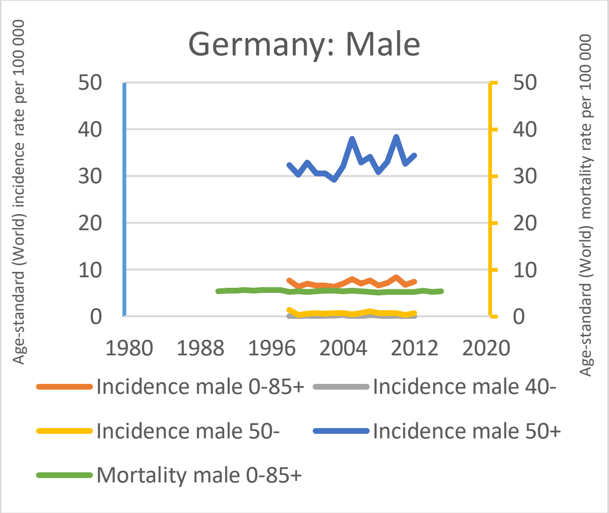
Germany: Male
Age-standard (World) incidence rate per 100 000
Age-standard (World) mortality rate per 100 000
50
40
30
20
10
0
1980
1988
1996
2004
2012
2020
Incidence male 0-85+
Incidence male 40-
Incidence male 50-
Incidence male 50+
Mortality male 0-85+

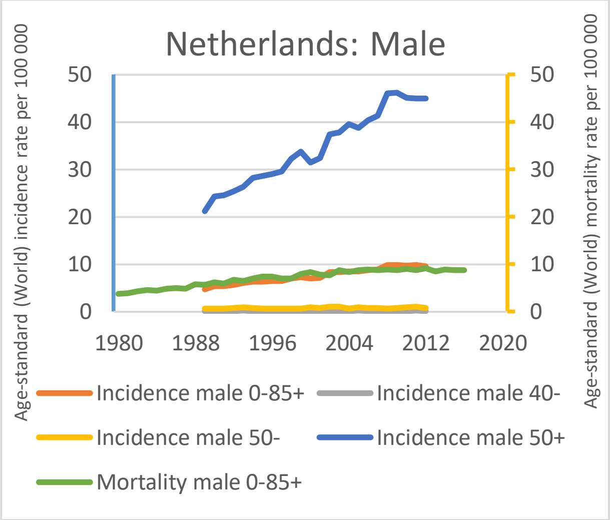
Netherlands: Male
Age-standard (World) incidence rate per 100 000
Age-standard (World) mortality rate per 100 000
50
40
30
20
10
0
1980
1988
1996
2004
2012
2020
Incidence male 0-85+
Incidence male 40-
Incidence male 50-
Incidence male 50+
Mortality male 0-85+

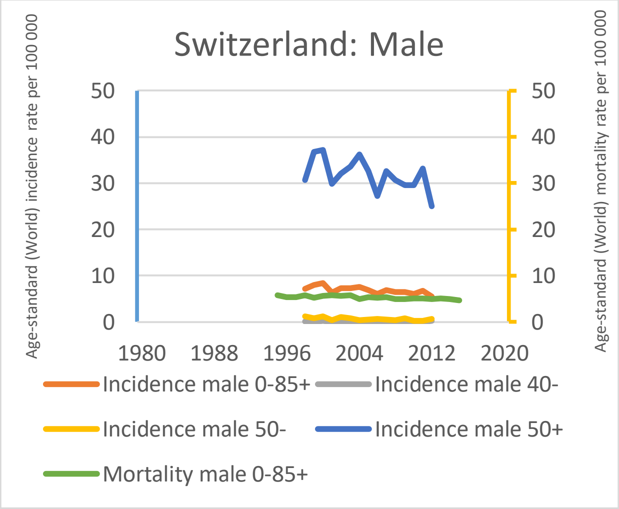
Switzerland: Male
Age-standard (World) incidence rate per 100 000
Age-standard (World) mortality rate per 100 000
50
40
30
20
10
0
1980
1988
1996
2004
2012
2020
Incidence male 0-85+
Incidence male 40-
Incidence male 50-
Incidence male 50+
Mortality male 0-85+

Southern Europe

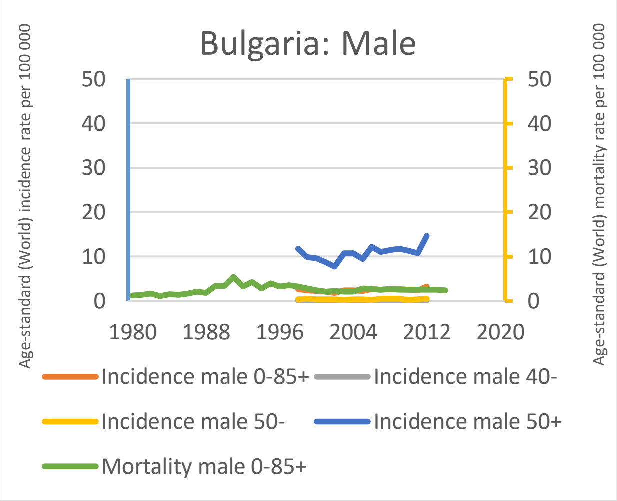

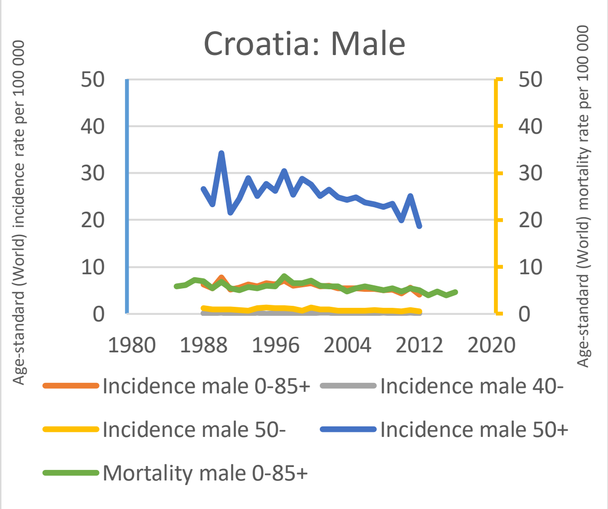

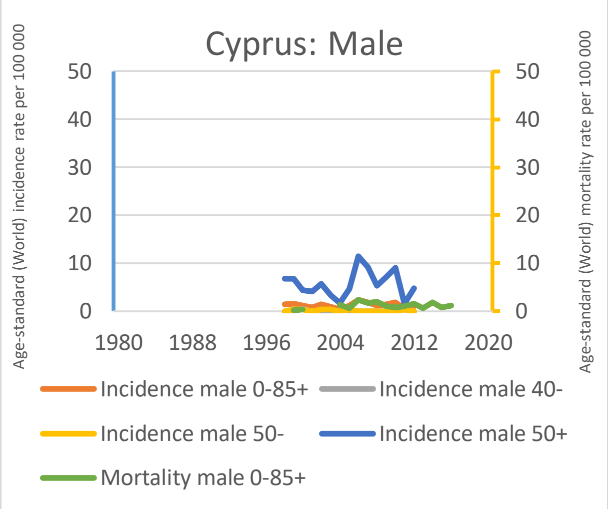

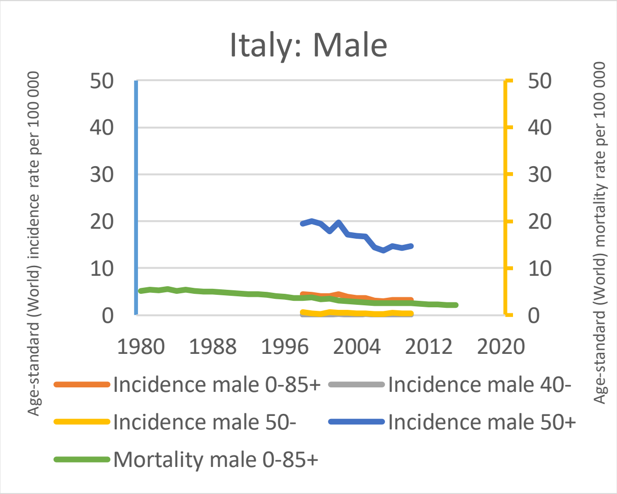

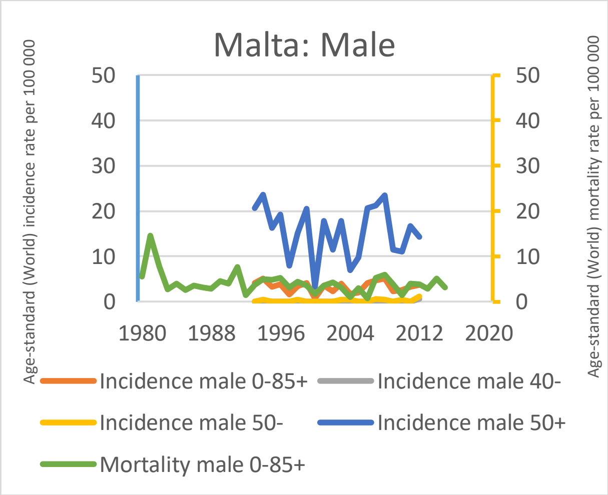

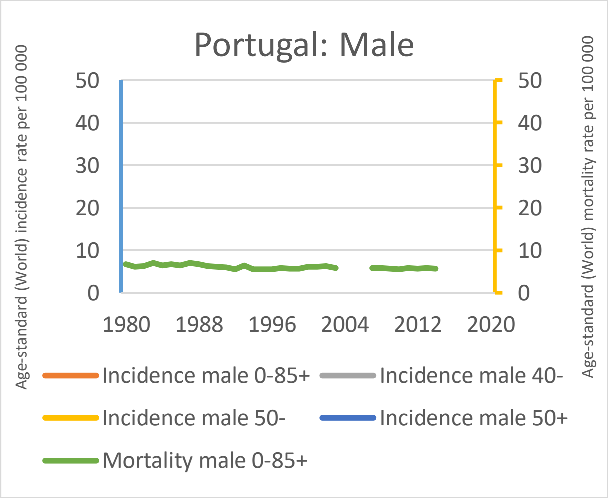

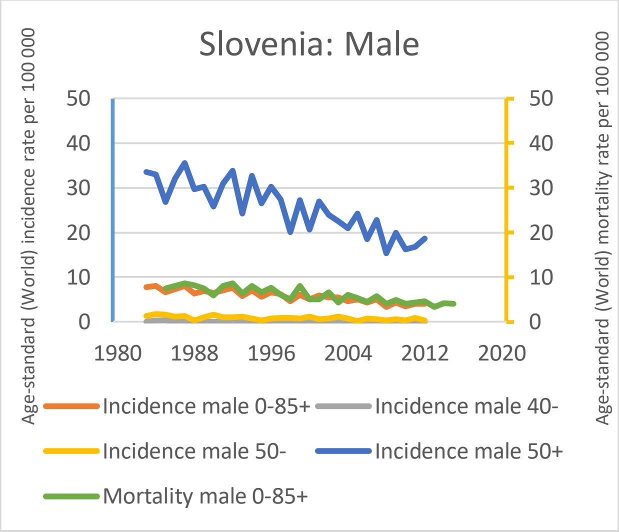

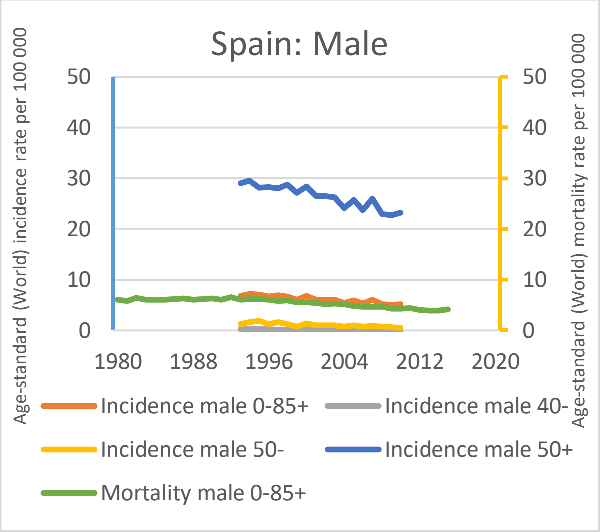

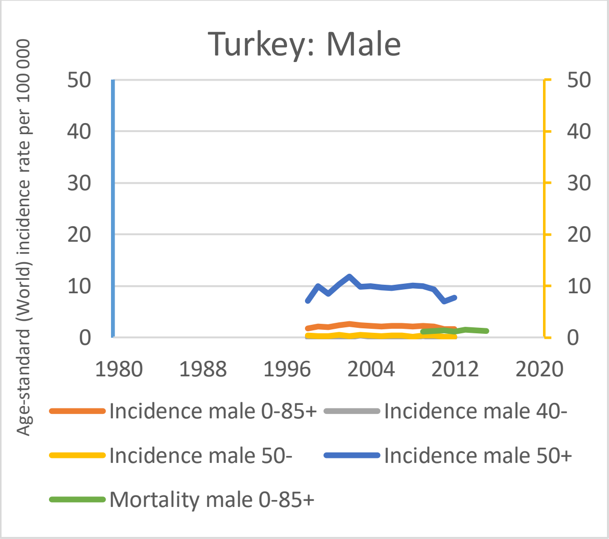

Eastern Europe

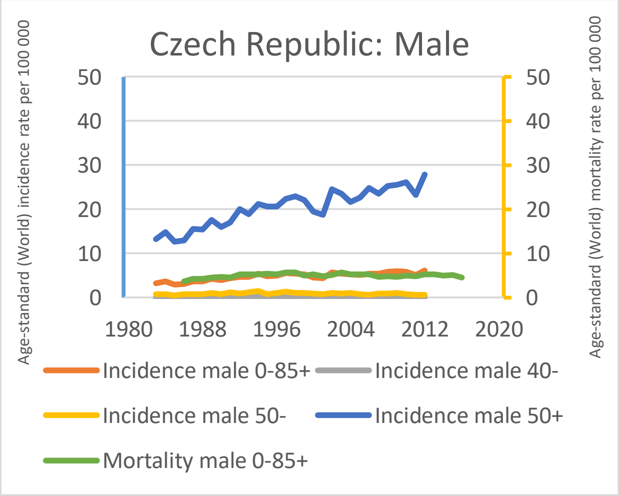

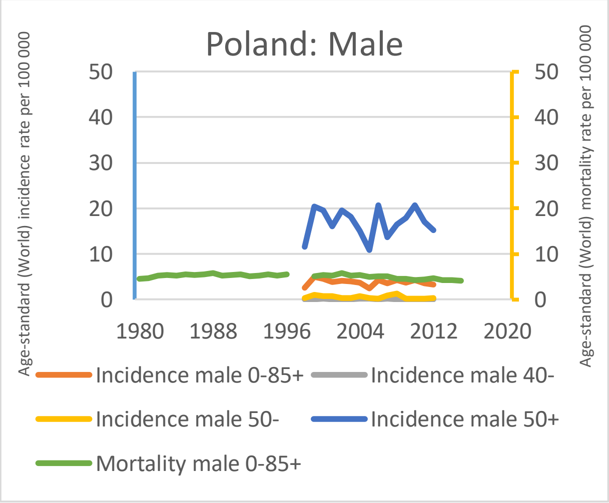

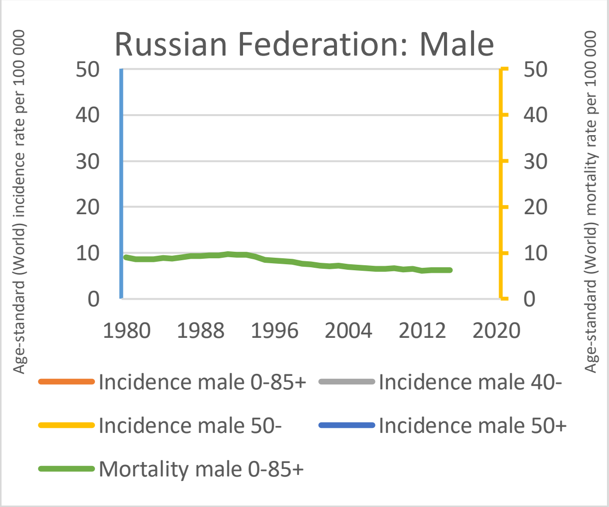

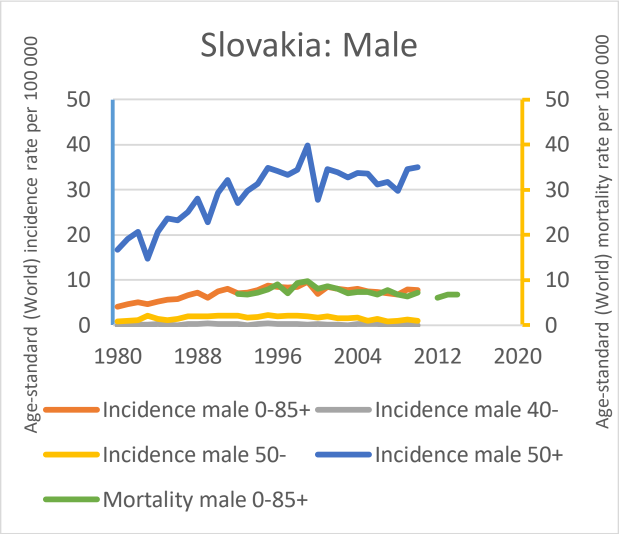
Slovakia: Male
Age-standard (World) incidence rate per 100 000
Age-standard (World) mortality rate per 100 000
50
40
30
20
10
0
1980
1988
1996
2004
2012
2020
Incidence male 0-85+
Incidence male 40-
Incidence male 50-
Incidence male 50+
Mortality male 0-85+

Africa

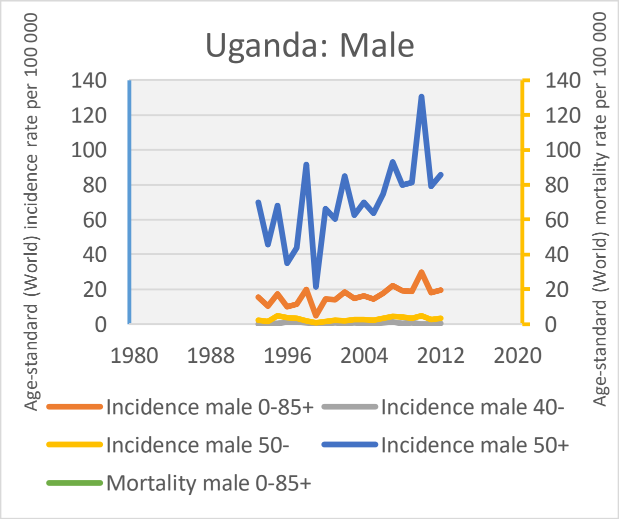
Uganda: Male
Age-standard (World) incidence rate per 100 000
Age-standard (World) mortality rate per 100 000
140
120
100
80
60
40
20
0
1980
1988
1996
2004
2012
2020
Incidence male 0-85+
Incidence male 40-
Incidence male 50-
Incidence male 50+
Mortality male 0-85+

Female

Asia

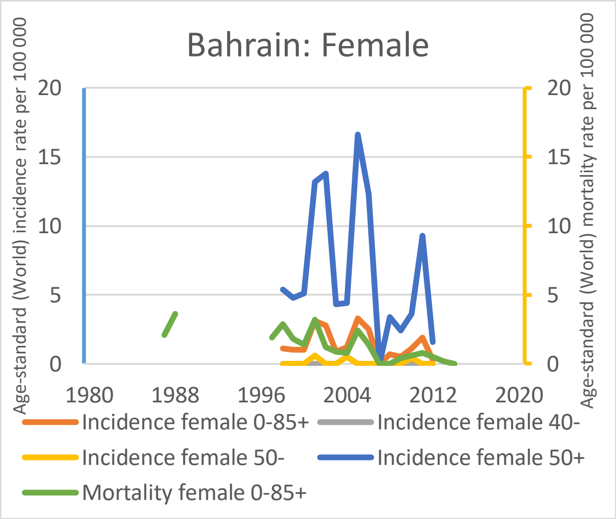
Bahrain: Female
Age-standard (World) incidence rate per 100 000
Age-standard (World) mortality rate per 100 000
20
15
10
5
0
1980
1988
1996
2004
2012
2020
Incidence female 0-85+
Incidence female 40-
Incidence female 50-
Incidence female 50+
Mortality female 0-85+

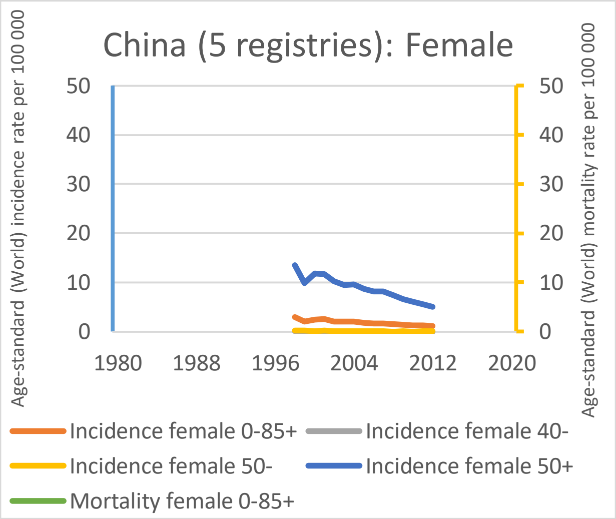
China (5 registries): Female
Age-standard (World) incidence rate per 100 000
Age-standard (World) mortality rate per 100 000
50
40
30
20
10
0
1980
1988
1996
2004
2012
2020
Incidence female 0-85+
Incidence female 40-
Incidence female 50-
Incidence female 50+
Mortality female 0-85+

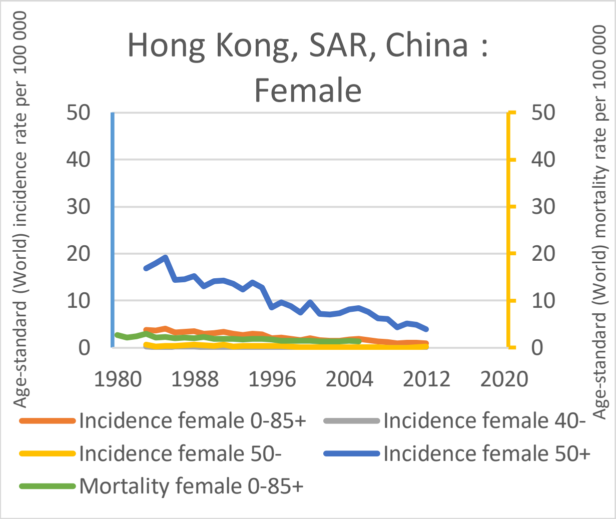
Hong Kong, SAR, China : Female
Age-standard (World) incidence rate per 100 000
Age-standard (World) mortality rate per 100 000
50
40
30
20
10
0
1980
1988
1996
2004
2012
2020
Incidence female 0-85+
Incidence female 40-
Incidence female 50-
Incidence female 50+
Mortality female 0-85+

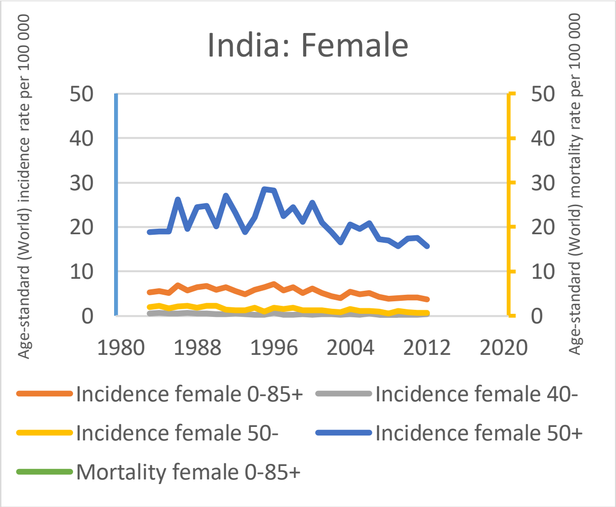
India: Female
Age-standard (World) incidence rate per 100 000
Age-standard (World) mortality rate per 100 000
50
40
30
20
10
0
1980
1988
1996
2004
2012
2020
Incidence female 0-85+
Incidence female 40-
Incidence female 50-
Incidence female 50+
Mortality female 0-85+

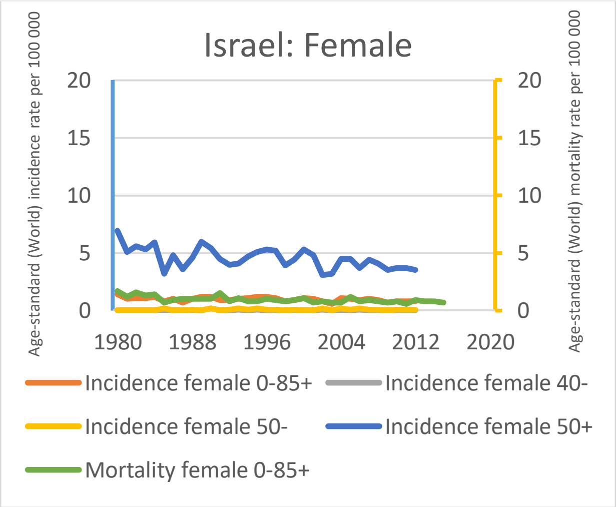
Israel: Female
Age-standard (World) incidence rate per 100 000
Age-standard (World) mortality rate per 100 000
20
15
10
5
0
1980
1988
1996
2004
2012
2020
Incidence female 0-85+
Incidence female 40-
Incidence female 50-
Incidence female 50+
Mortality female 0-85+

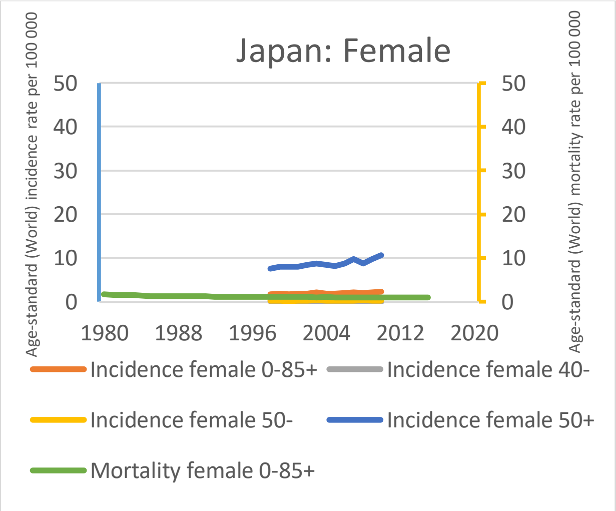
Japan: Female
Age-standard (World) incidence rate per 100 000
Age-standard (World) mortality rate per 100 000
50
40
30
20
10
0
1980
1988
1996
2004
2012
2020
Incidence female 0-85+
Incidence female 40-
Incidence female 50-
Incidence female 50+
Mortality female 0-85+

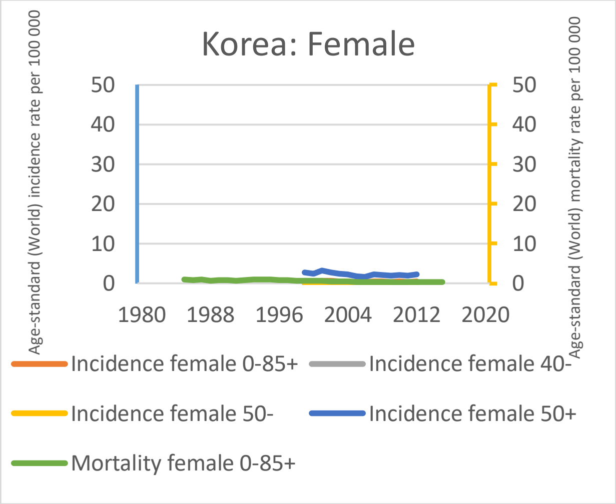
Korea: Female
Age-standard (World) incidence rate per 100 000
Age-standard (World) mortality rate per 100 000
50
40
30
20
10
0
1980
1988
1996
2004
2012
2020
Incidence female 0-85+
Incidence female 40-
Incidence female 50-
Incidence female 50+
Mortality female 0-85+

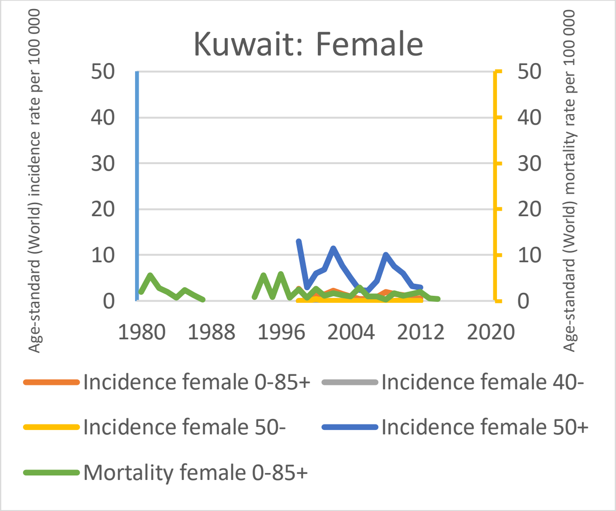
Kuwait: Female
Age-standard (World) incidence rate per 100 000
Age-standard (World) mortality rate per 100 000
50
40
30
20
10
0
1980
1988
1996
2004
2012
2020
Incidence female 0-85+
Incidence female 40-
Incidence female 50-
Incidence female 50+
Mortality female 0-85+

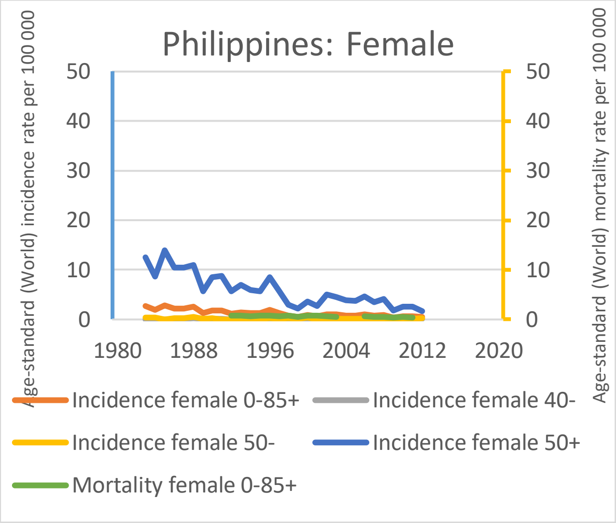
Philippines: Female
Age-standard (World) incidence rate per 100 000
Age-standard (World) mortality rate per 100 000
50
40
30
20
10
0
1980
1988
1996
2004
2012
2020
Incidence female 0-85+
Incidence female 40-
Incidence female 50-
Incidence female 50+
Mortality female 0-85+

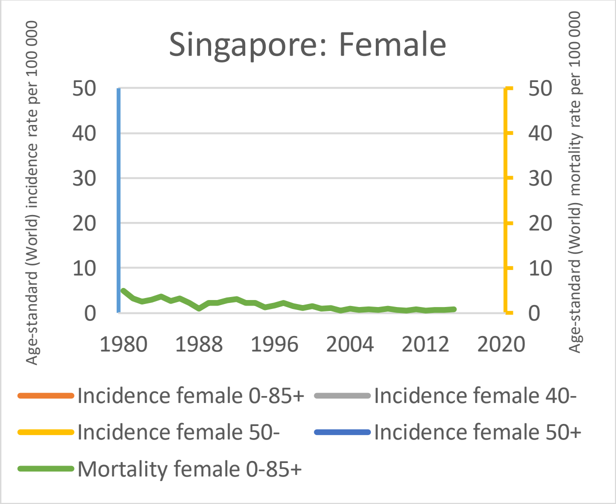
Singapore: Female
Age-standard (World) incidence rate per 100 000
Age-standard (World) mortality rate per 100 000
50
40
30
20
10
0
1980
1988
1996
2004
2012
2020
Incidence female 0-85+
Incidence female 40-
Incidence female 50-
Incidence female 50+
Mortality female 0-85+

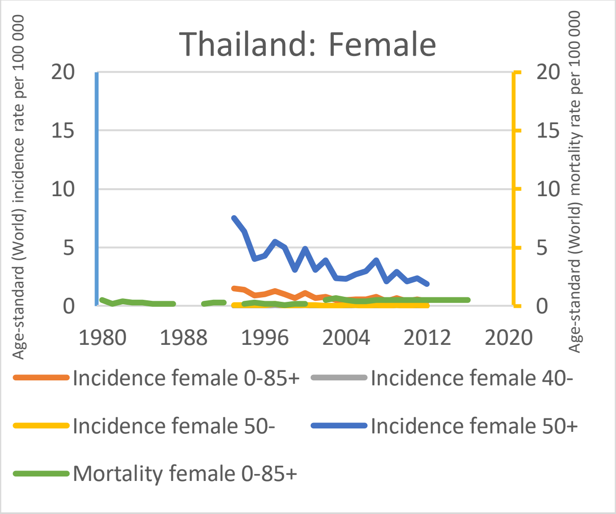
Thailand: Female
Age-standard (World) incidence rate per 100 000
Age-standard (World) mortality rate per 100 000
20
15
10
5
0
1980
1988
1996
2004
2012
2020
Incidence female 0-85+
Incidence female 40-
Incidence female 50-
Incidence female 50+
Mortality female 0-85+

Oceania

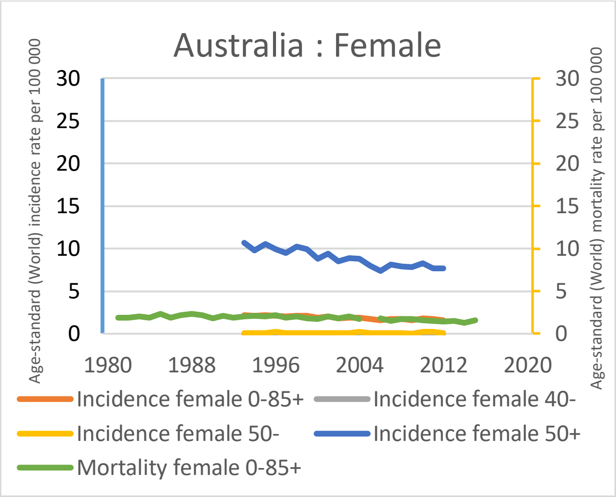

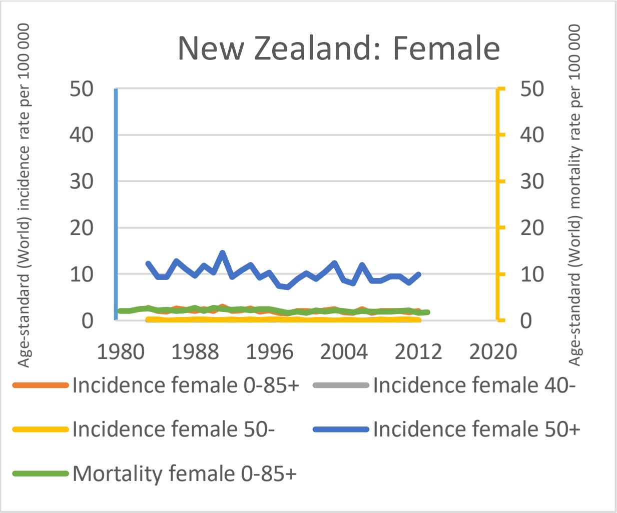

Northern America

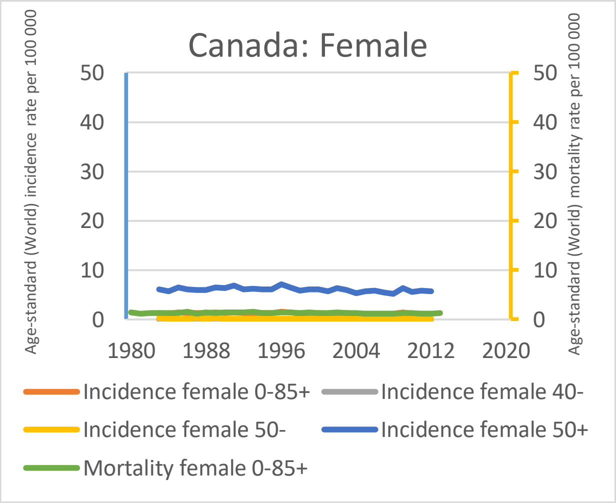

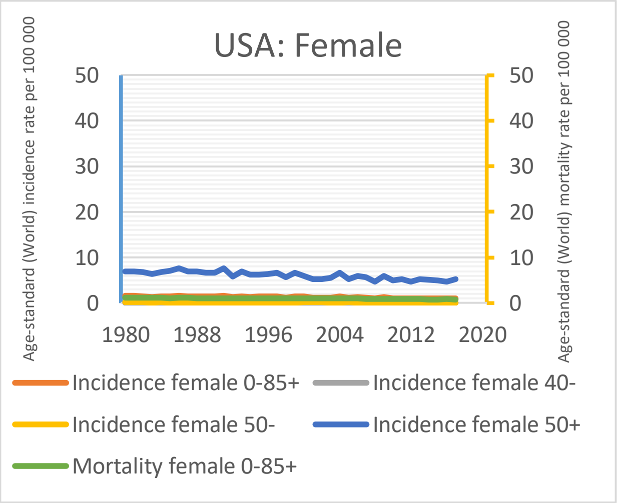

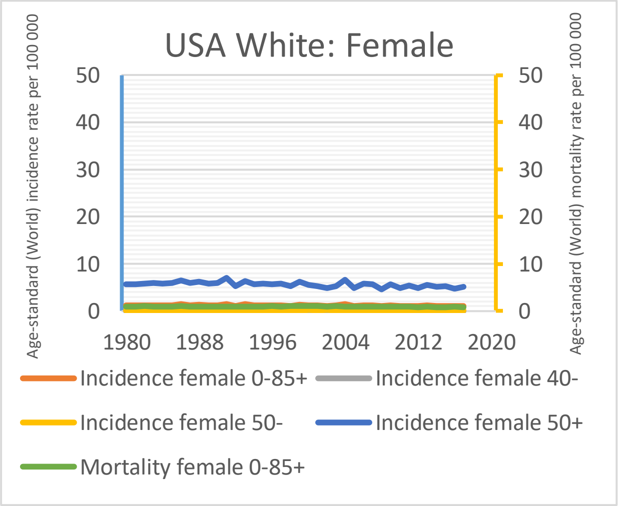

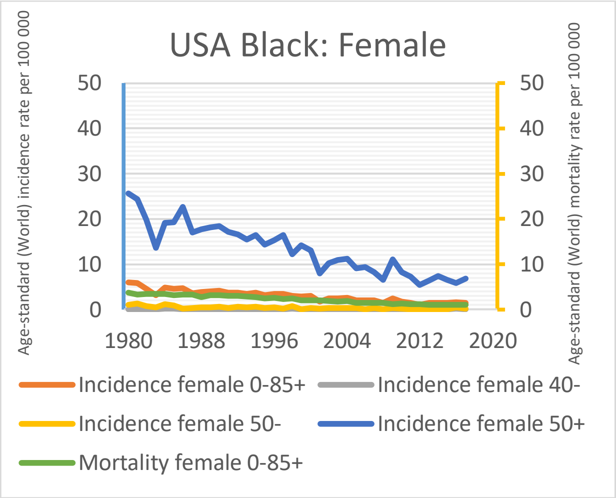

Southern America

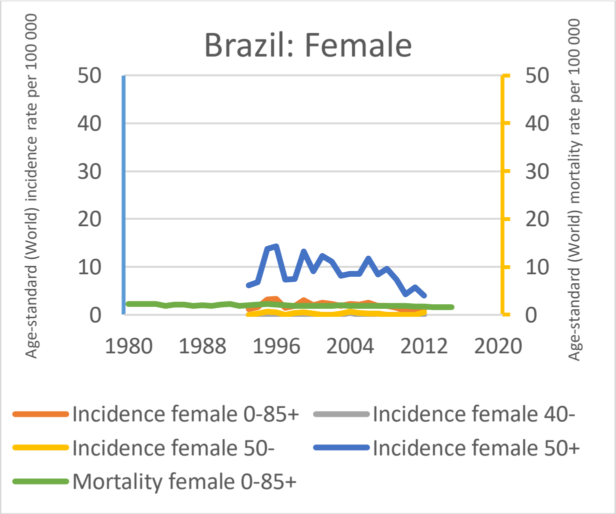

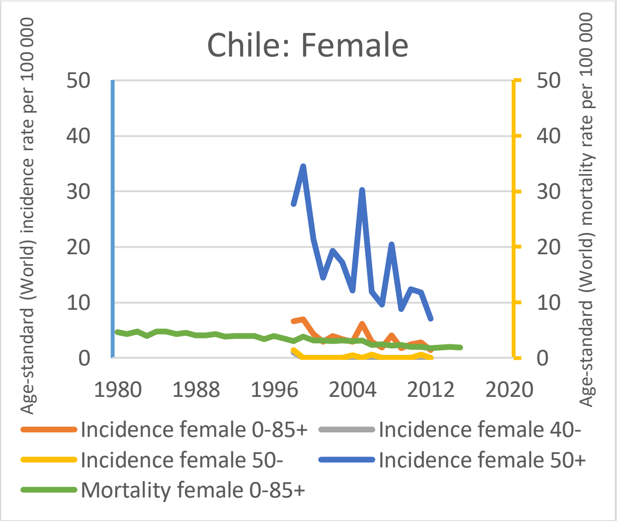

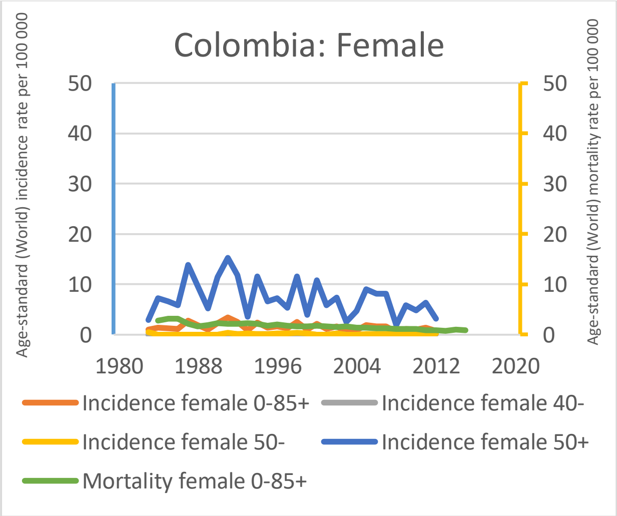

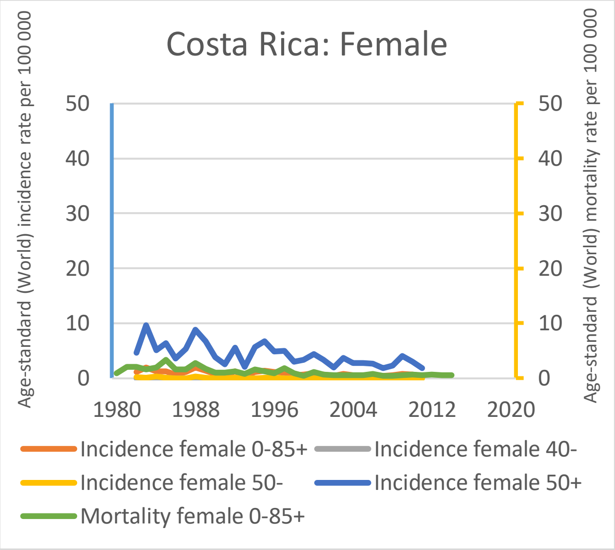

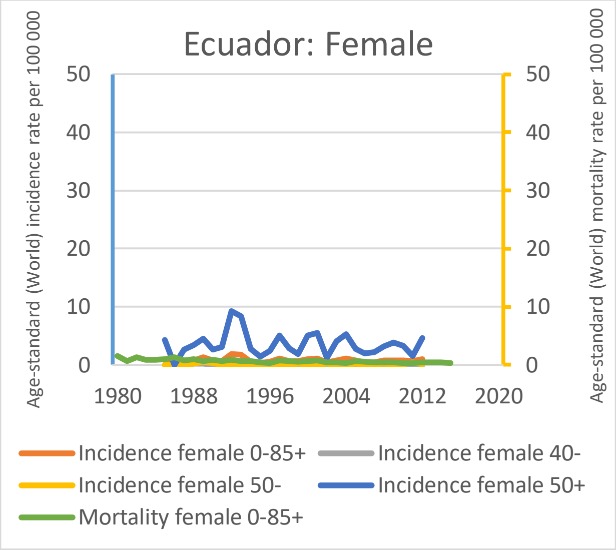

Northern Europe

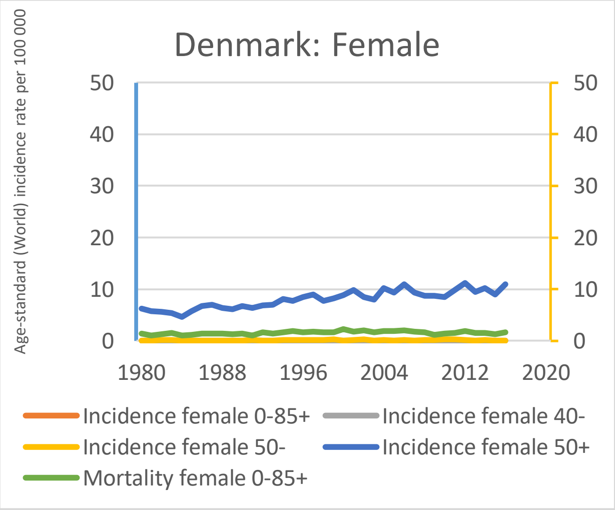

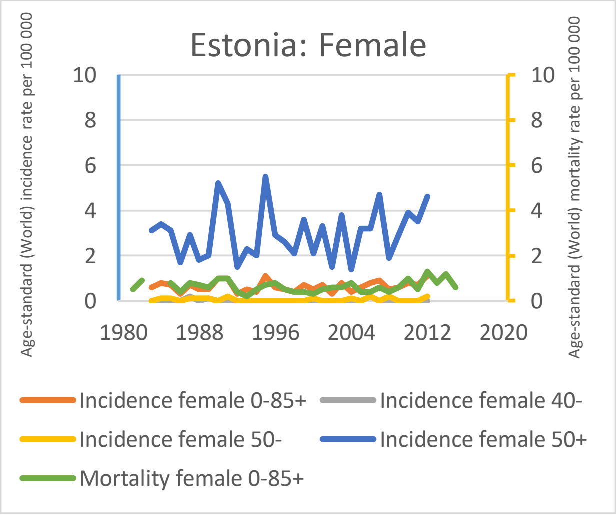

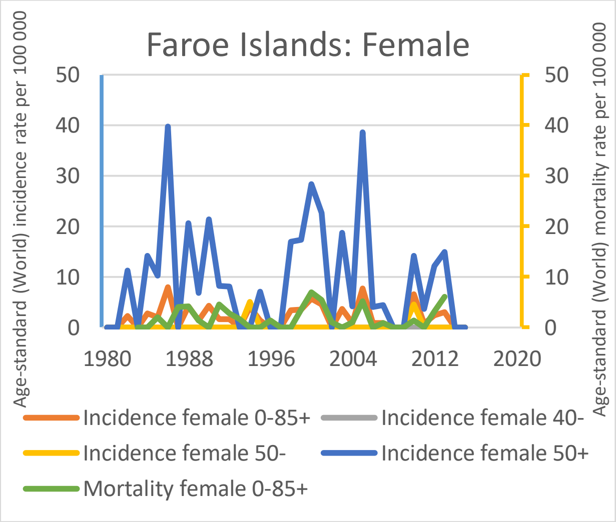

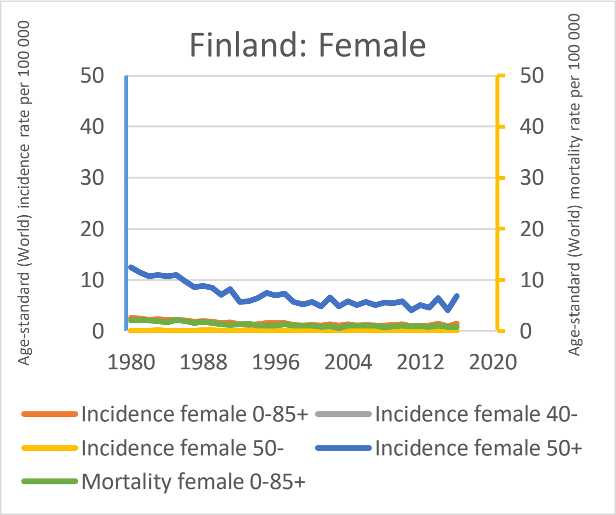
Finland: Female
Age-standard (World) incidence rate per 100 000
Age-standard (World) mortality rate per 100 000
50
40
30
20
10
0
1980 1988 1996 2004 2012 2020
Incidence female 0-85+
Incidence female 40-
Incidence female 50-
Incidence female 50+
Mortality female 0-85+

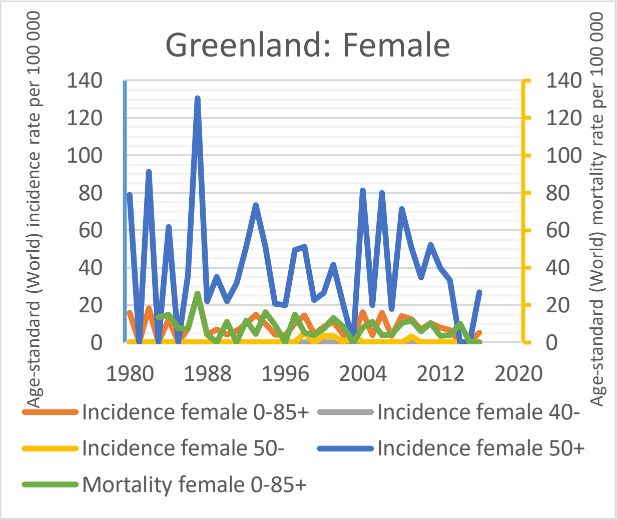
Greenland: Female
Age-standard (World) incidence rate per 100 000
Age-standard (World) mortality rate per 100 000
140
120
100
80
60
40
20
0
1980 1988 1996 2004 2012 2020
Incidence female 0-85+
Incidence female 40-
Incidence female 50-
Incidence female 50+
Mortality female 0-85+

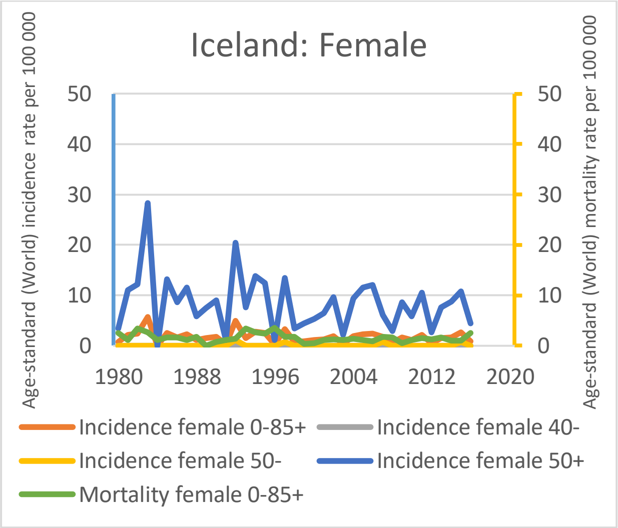
Iceland: Female
Age-standard (World) incidence rate per 100 000
Age-standard (World) mortality rate per 100 000
50
40
30
20
10
0
1980 1988 1996 2004 2012 2020
Incidence female 0-85+
Incidence female 40-
Incidence female 50-
Incidence female 50+
Mortality female 0-85+

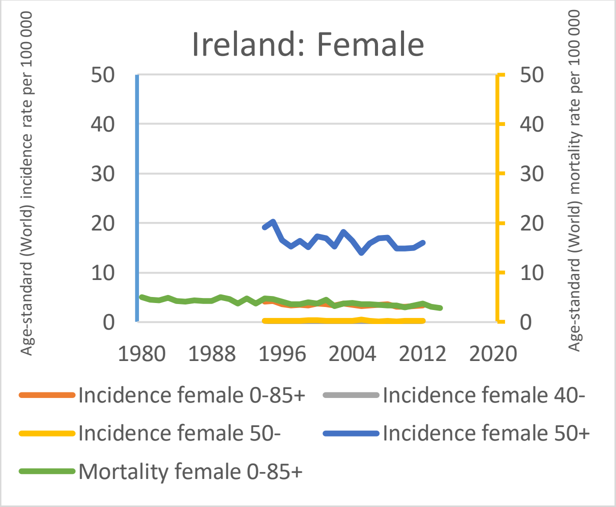
Ireland: Female
Age-standard (World) incidence rate per 100 000
Age-standard (World) mortality rate per 100 000
50
40
30
20
10
0
1980 1988 1996 2004 2012 2020
Incidence female 0-85+
Incidence female 40-
Incidence female 50-
Incidence female 50+
Mortality female 0-85+

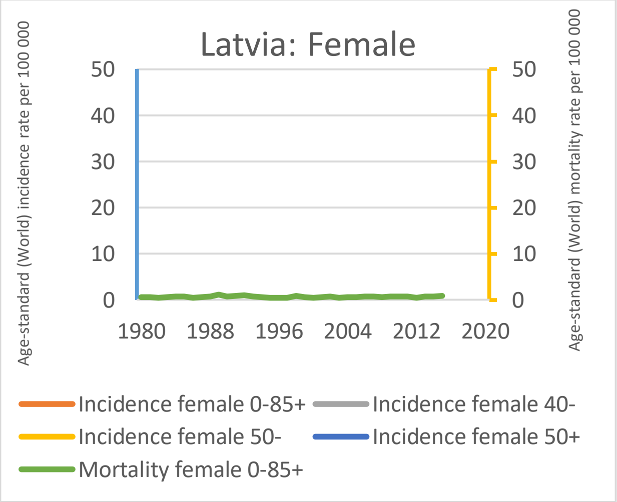
Latvia: Female
Age-standard (World) incidence rate per 100 000
Age-standard (World) mortality rate per 100 000
50
40
30
20
10
0
1980 1988 1996 2004 2012 2020
Incidence female 0-85+
Incidence female 40-
Incidence female 50-
Incidence female 50+
Mortality female 0-85+

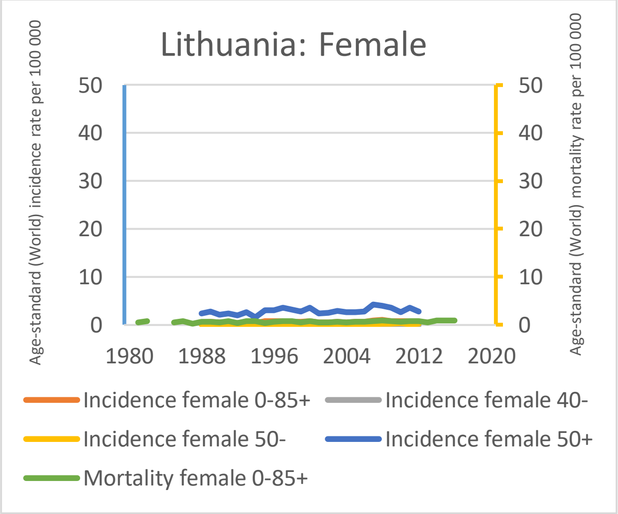
Lithuania: Female
Age-standard (World) incidence rate per 100 000
Age-standard (World) mortality rate per 100 000
50
40
30
20
10
0
1980 1988 1996 2004 2012 2020
Incidence female 0-85+
Incidence female 40-
Incidence female 50-
Incidence female 50+
Mortality female 0-85+

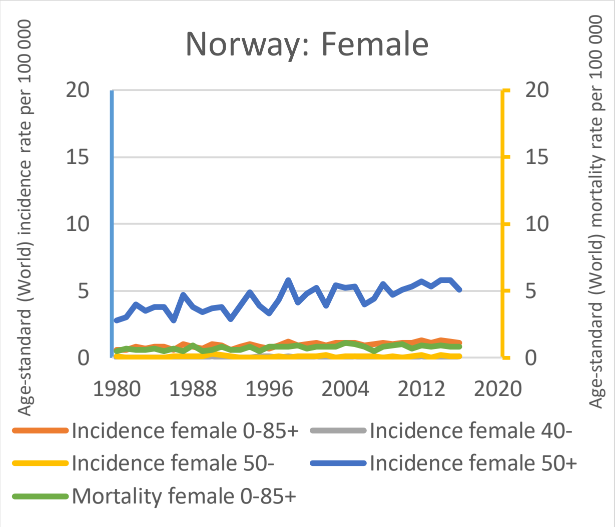

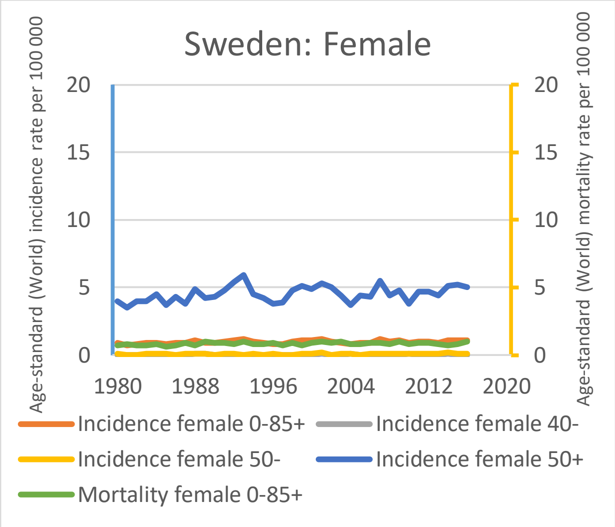

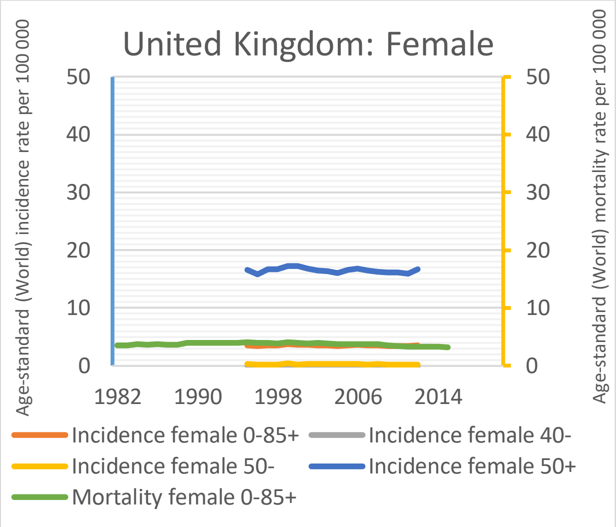

Western Europe

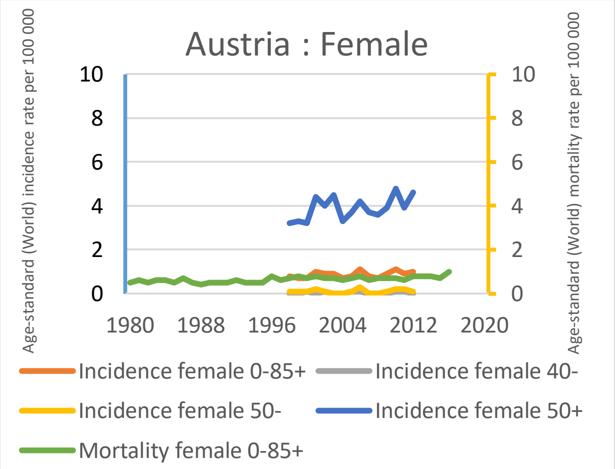

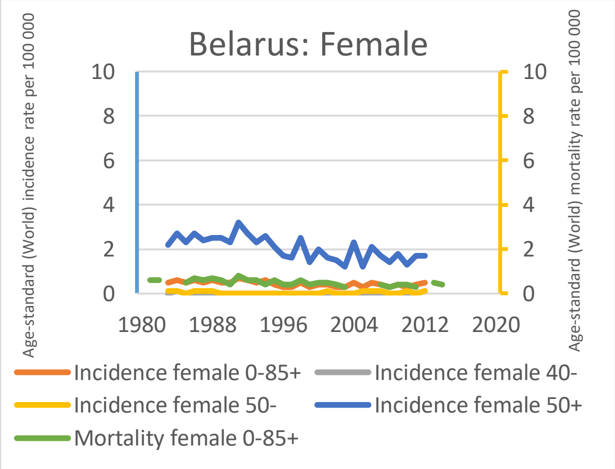

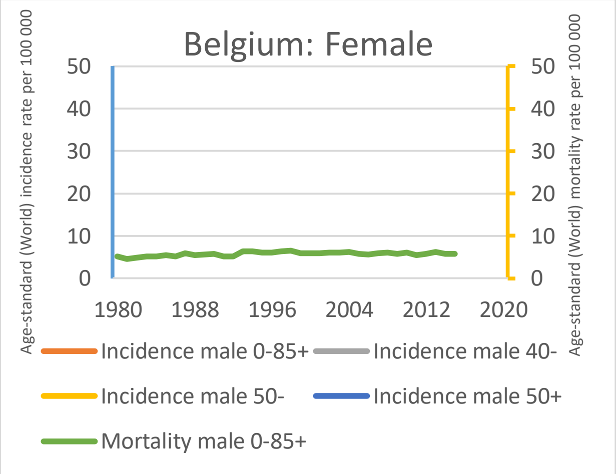

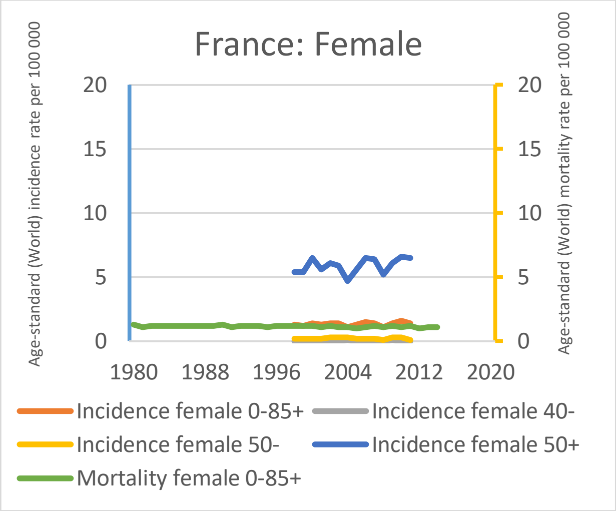
France: Female
Age-standard (World) incidence rate per 100 000
Age-standard (World) mortality rate per 100 000
20
15
10
5
0
1980
1988
1996
2004
2012
2020
Incidence female 0-85+
Incidence female 40-
Incidence female 50-
Incidence female 50+
Mortality female 0-85+

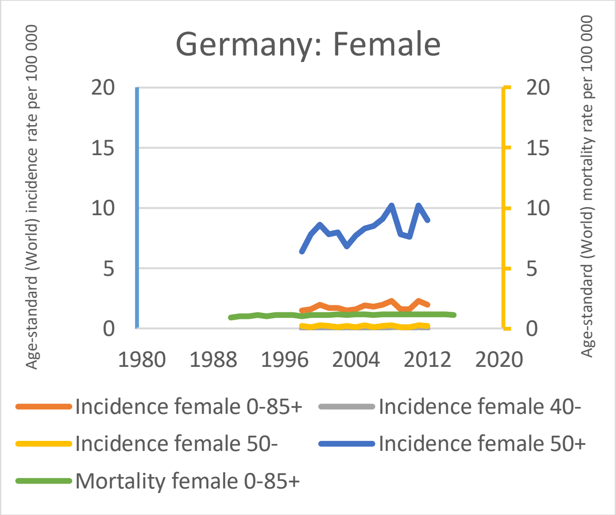
Germany: Female
Age-standard (World) incidence rate per 100 000
Age-standard (World) mortality rate per 100 000
20
15
10
5
0
1980
1988
1996
2004
2012
2020
Incidence female 0-85+
Incidence female 40-
Incidence female 50-
Incidence female 50+
Mortality female 0-85+

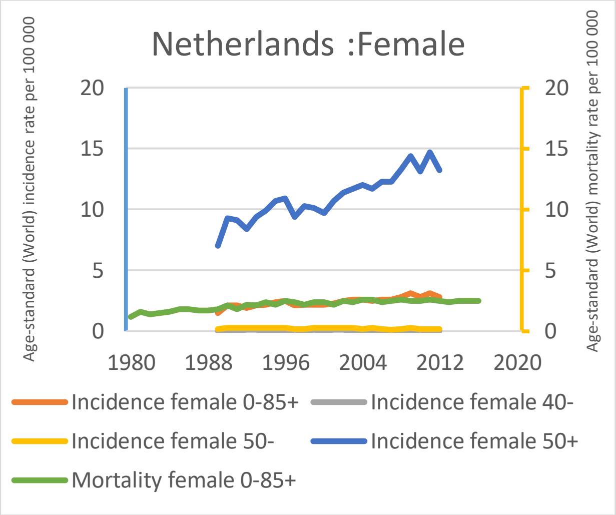
Netherlands :Female
Age-standard (World) incidence rate per 100 000
Age-standard (World) mortality rate per 100 000
20
15
10
5
0
1980
1988
1996
2004
2012
2020
Incidence female 0-85+
Incidence female 40-
Incidence female 50-
Incidence female 50+
Mortality female 0-85+

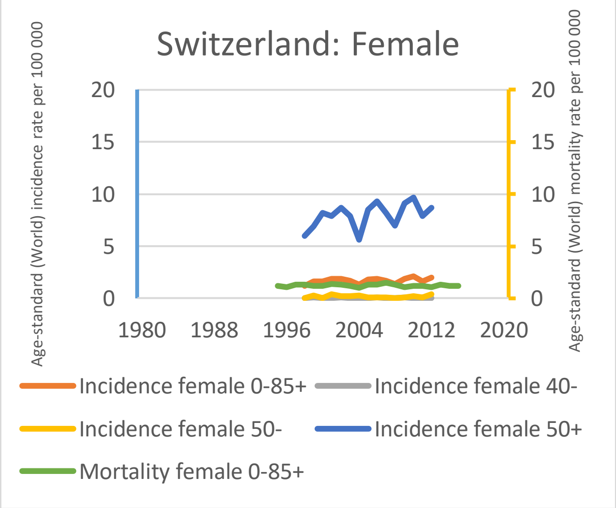
Switzerland: Female
Age-standard (World) incidence rate per 100 000
Age-standard (World) mortality rate per 100 000
20
15
10
5
0
1980
1988
1996
2004
2012
2020
Incidence female 0-85+
Incidence female 40-
Incidence female 50-
Incidence female 50+
Mortality female 0-85+

Southern Europe

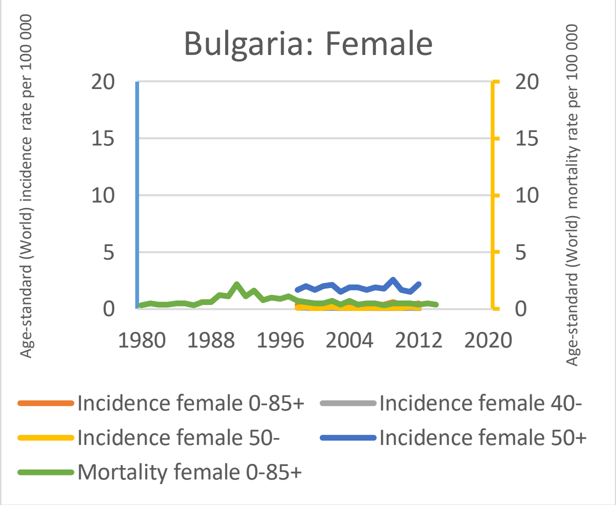

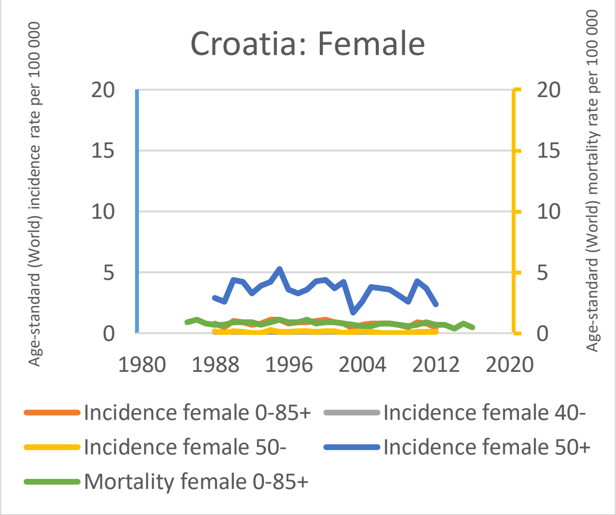

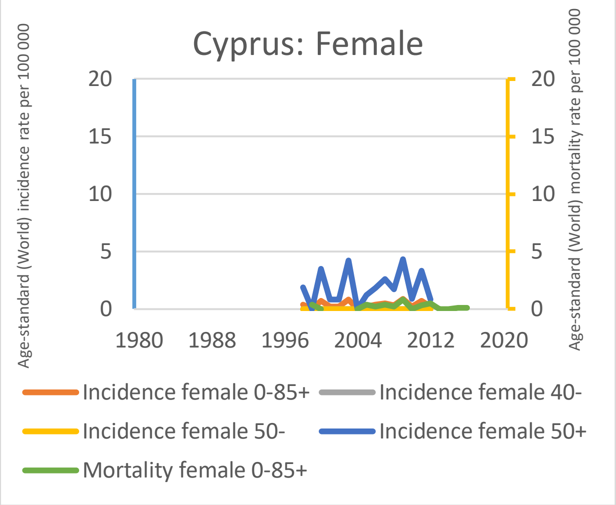

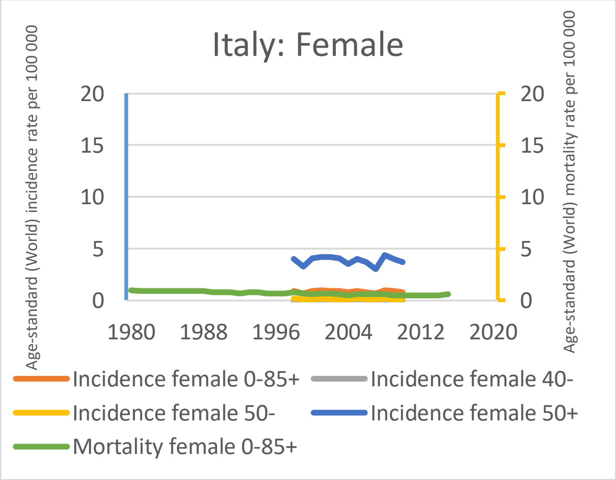

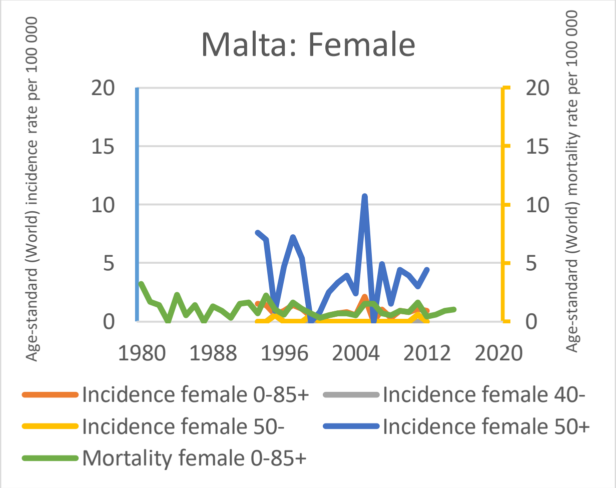

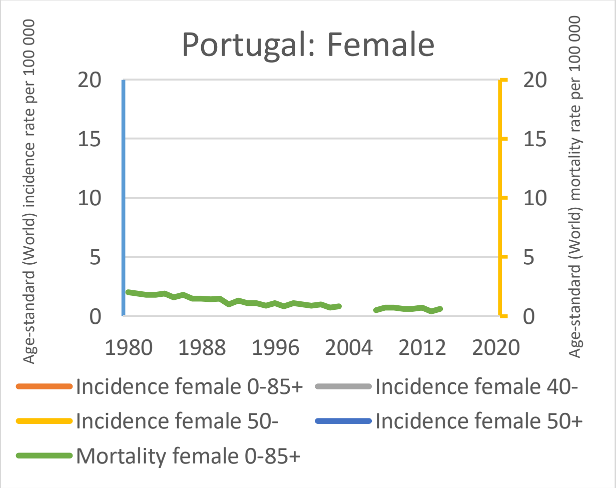

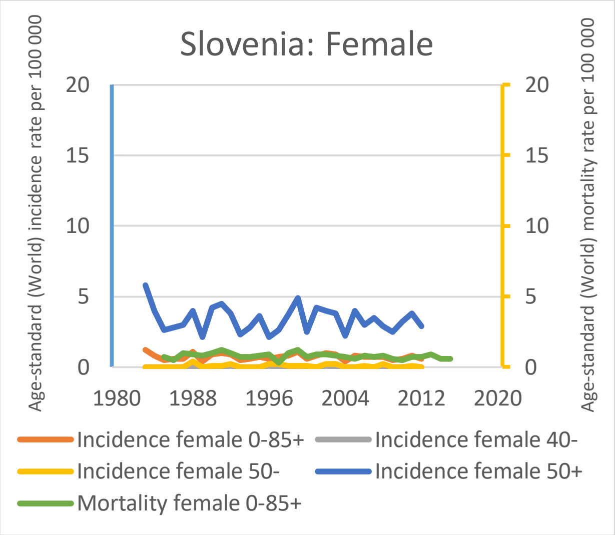

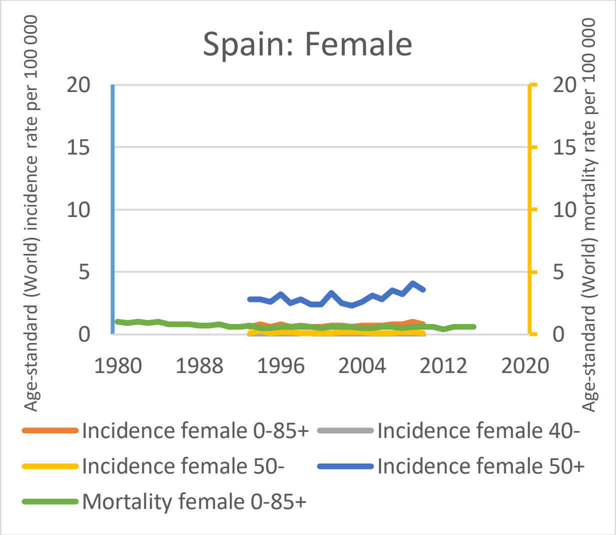

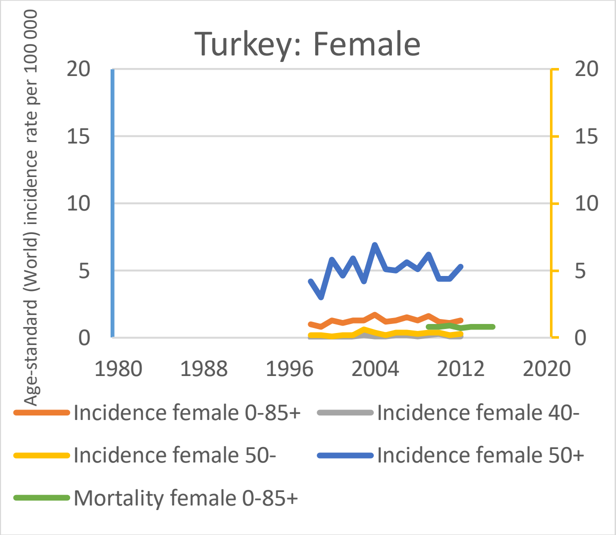

Eastern Europe

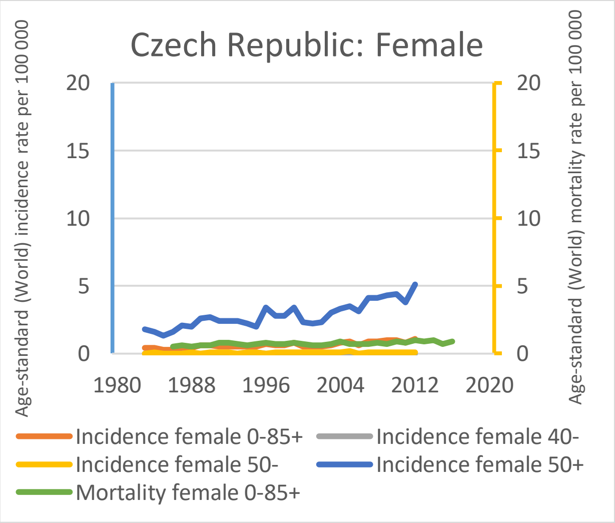

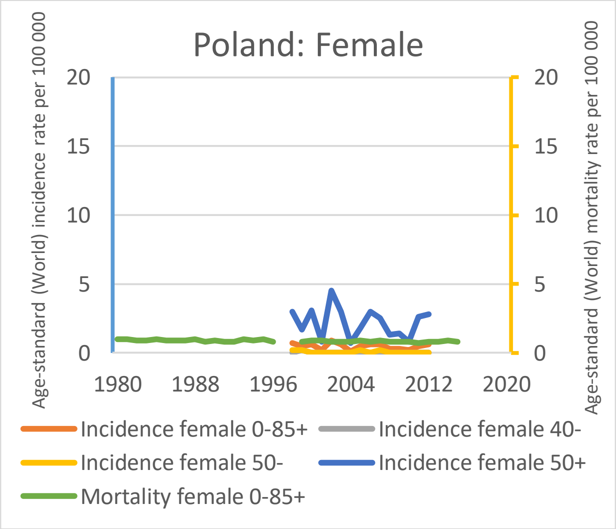

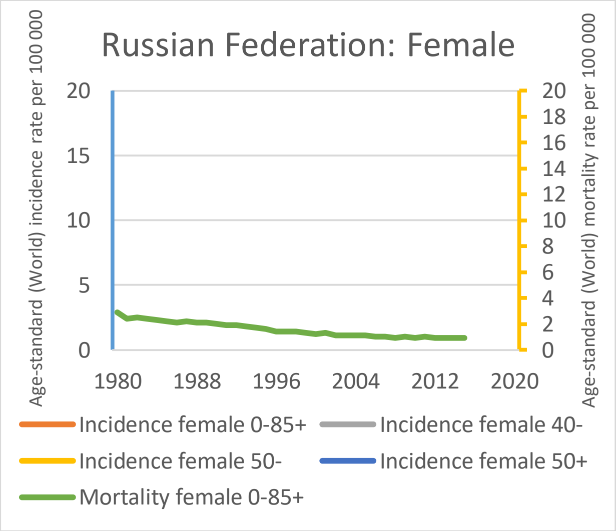

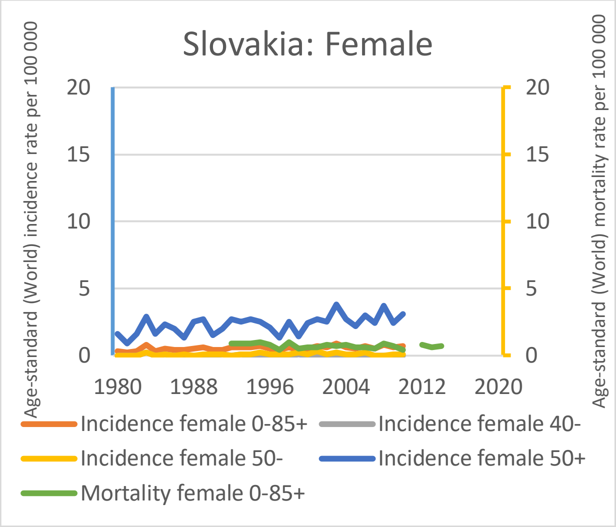
Slovakia: Female
Age-standard (World) incidence rate per 100 000
Age-standard (World) mortality rate per 100 000
20
15
10
5
0
1980
1988
1996
2004
2012
2020
Incidence female 0-85+
Incidence female 40-
Incidence female 50-
Incidence female 50+
Mortality female 0-85+

Africa

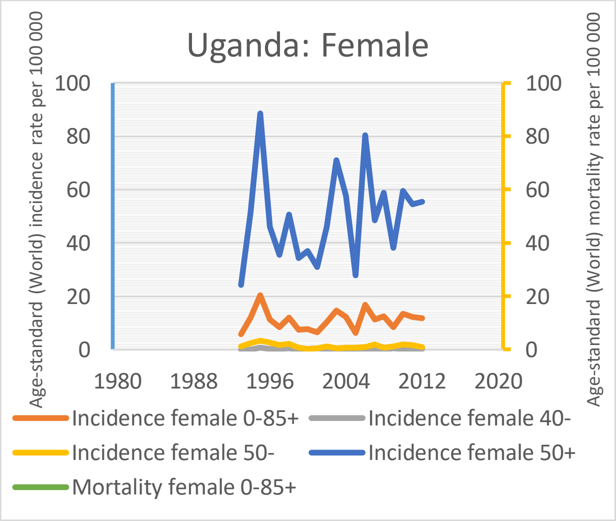
Uganda: Female
Age-standard (World) incidence rate per 100 000
Age-standard (World) mortality rate per 100 000
100
80
60
40
20
0
1980
1988
1996
2004
2012
2020
Incidence female 0-85+
Incidence female 40-
Incidence female 50-
Incidence female 50+
Mortality female 0-85+

Figure S2. The joinpoint regression analysis of esophageal cancer

a.) Incidence male aged 0 to 85 years old

Asia

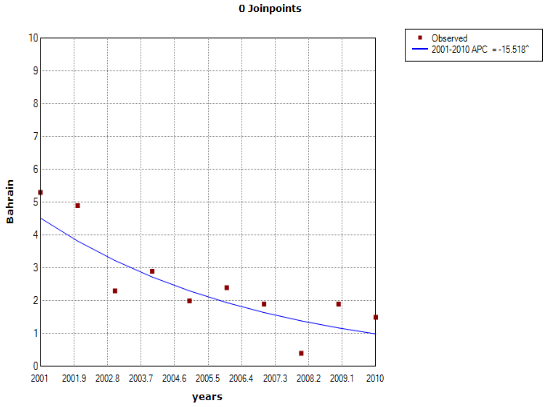

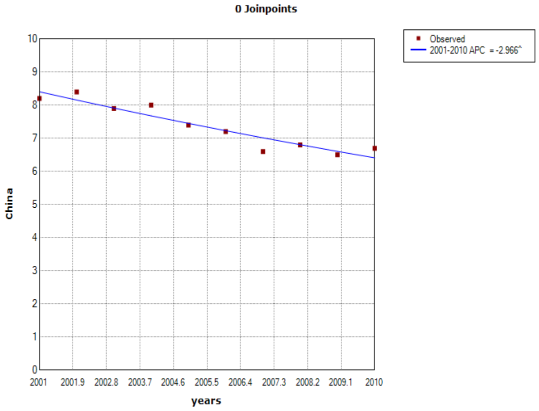

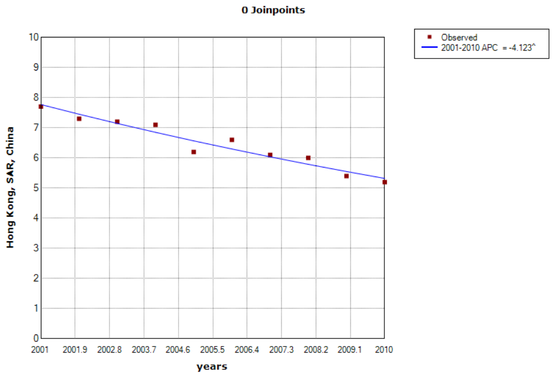

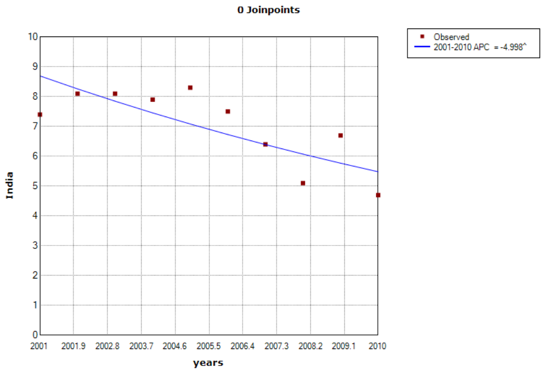

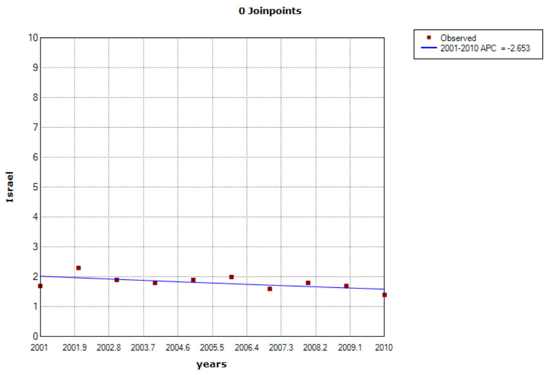

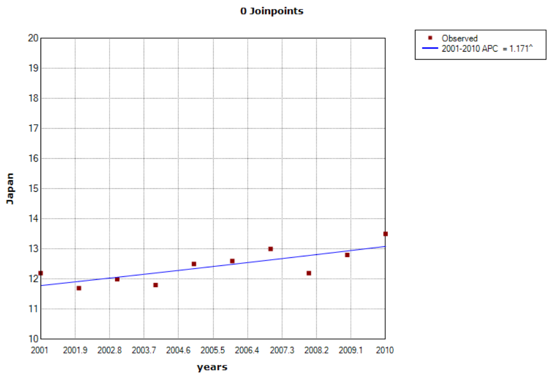

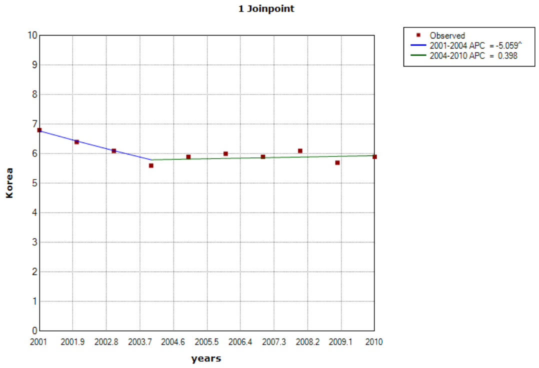
1 Joinpoint
Observed
2001-2004 APC = -5.059^
2004-2010 APC = 0.398
Korea
years

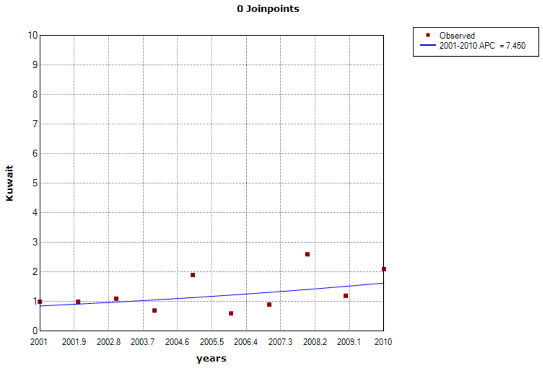
0 Joinpoints
Observed
2001-2010 APC = 7.450
Kuwait
years

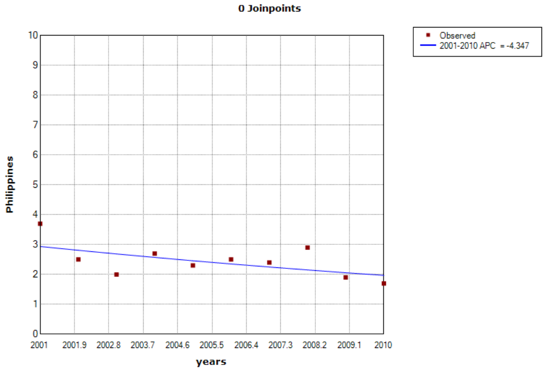
0 Joinpoints
Observed
2001-2010 APC = -4.347
Philippines
years

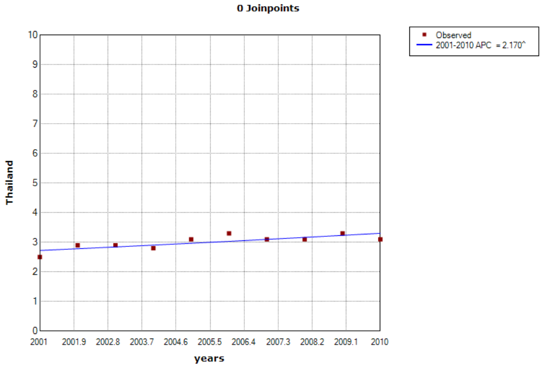
0 Joinpoints
Observed
2001-2010 APC = 2.170^
Thailand
years

Oceania

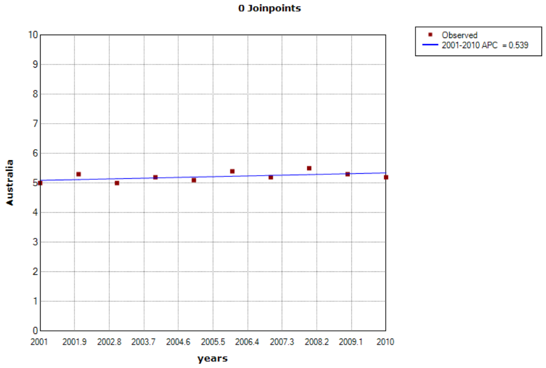
0 Joinpoints
Observed
2001-2010 APC = 0.539
Australia
years

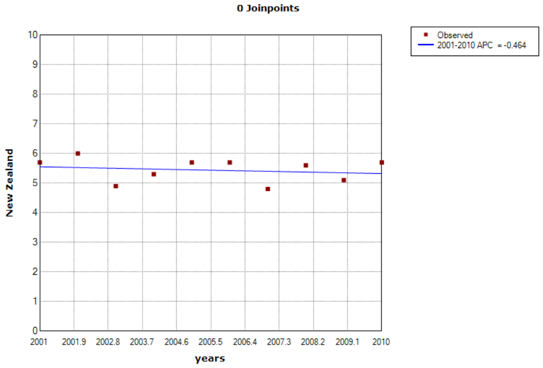
0 Joinpoints
Observed
2001-2010 APC = -0.464
New Zealand
years

Northern America

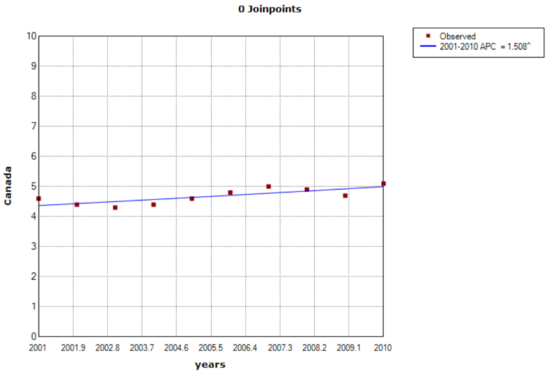

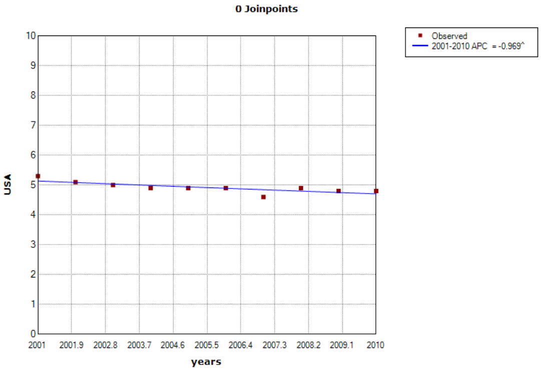

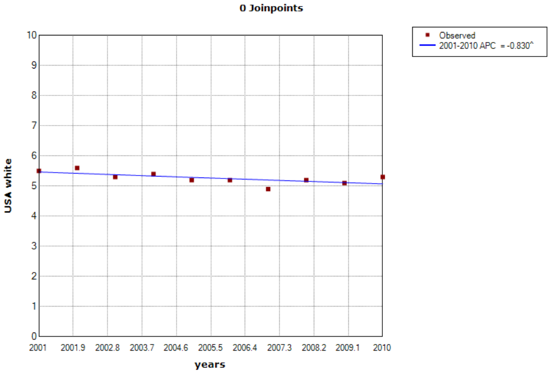

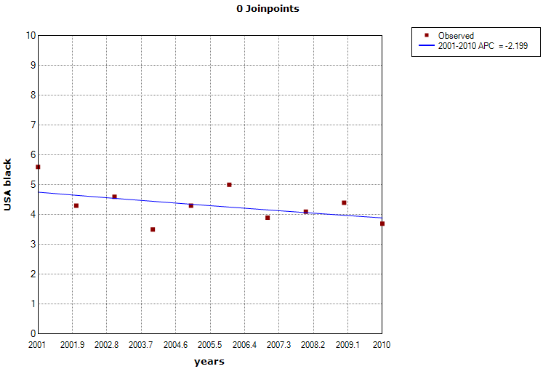

Southern America

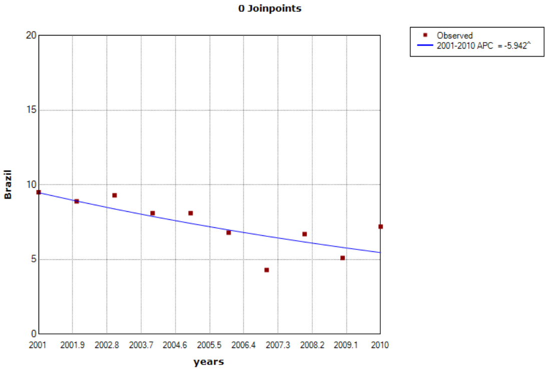

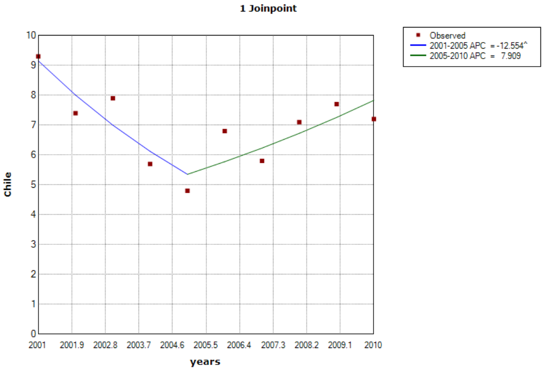

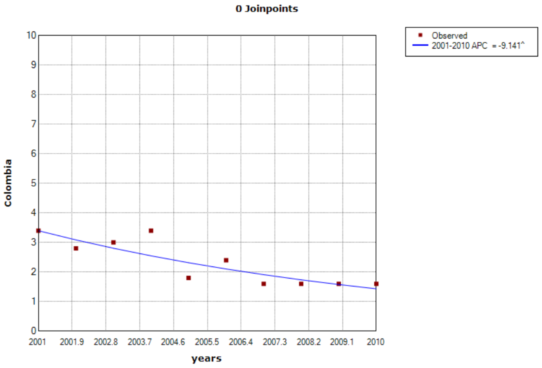

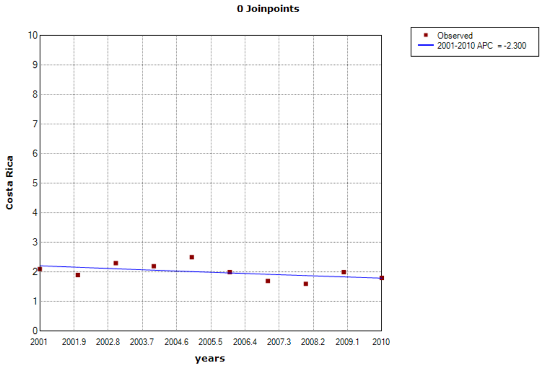

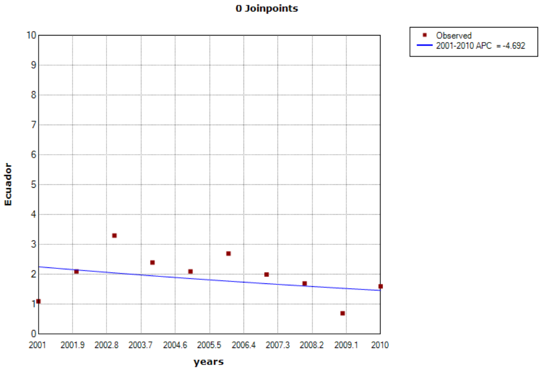

Northern Europe

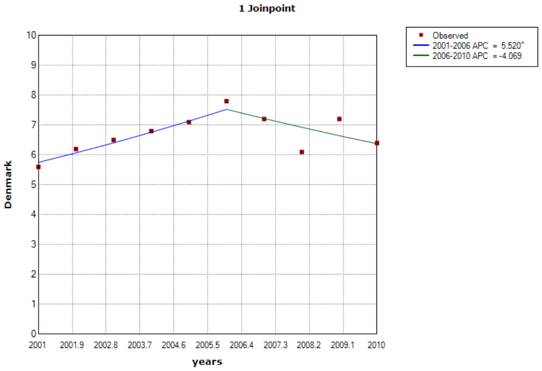

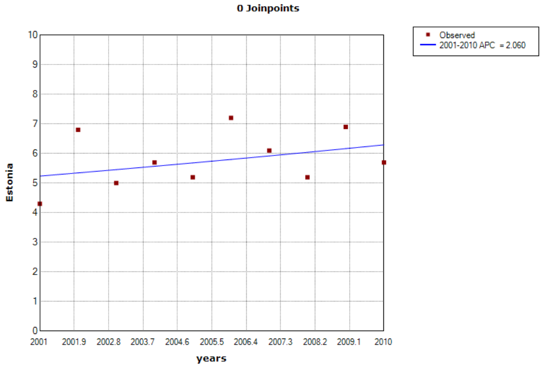

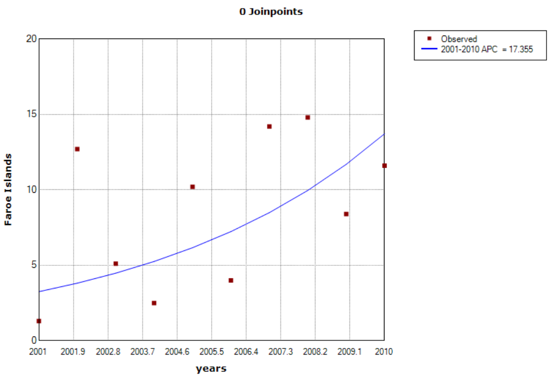
0 Joinpoints
Observed
2001-2010 APC = 17.355
Faroe Islands
years

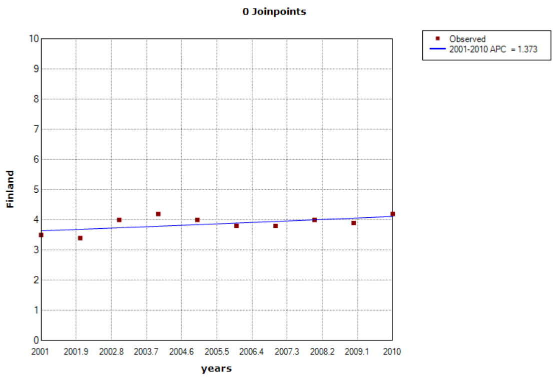
0 Joinpoints
Observed
2001-2010 APC = 1.373
Finland
years

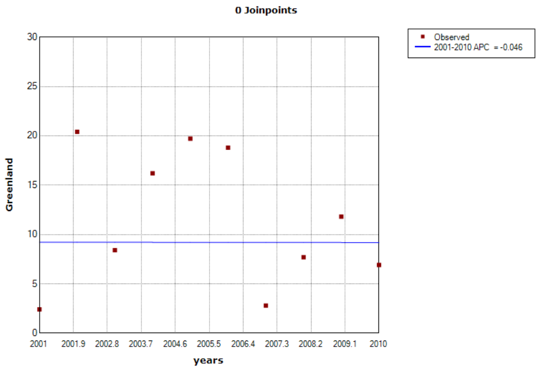
0 Joinpoints
Observed
2001-2010 APC = -0.046
Greenland
years

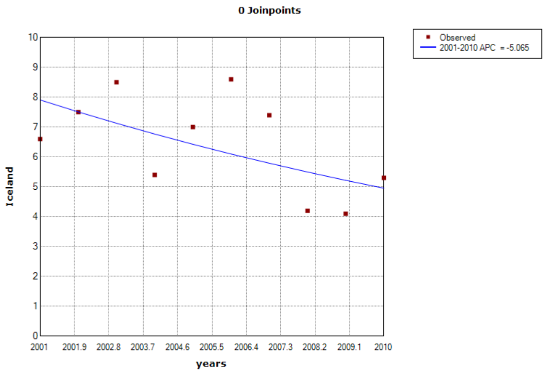
0 Joinpoints
Observed
2001-2010 APC = -5.065
Iceland
years

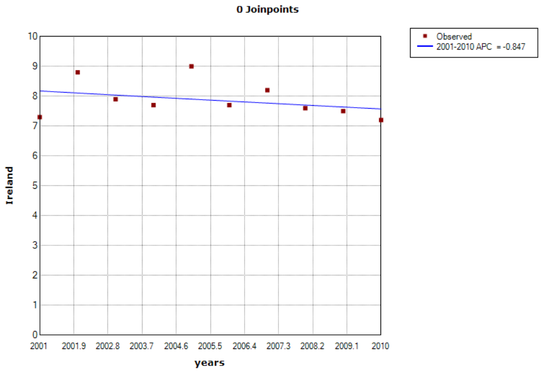
0 Joinpoints
Observed
2001-2010 APC = -0.847
Ireland
years

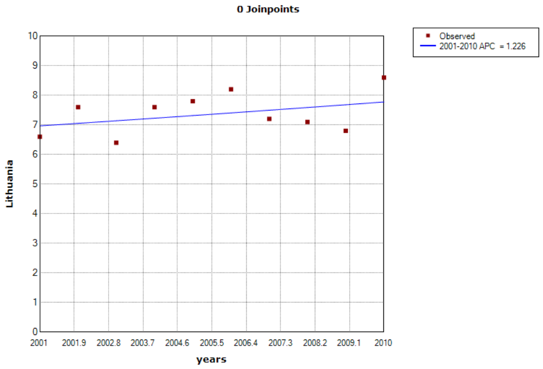
0 Joinpoints
Observed
2001-2010 APC = 1.226
Lithuania
years

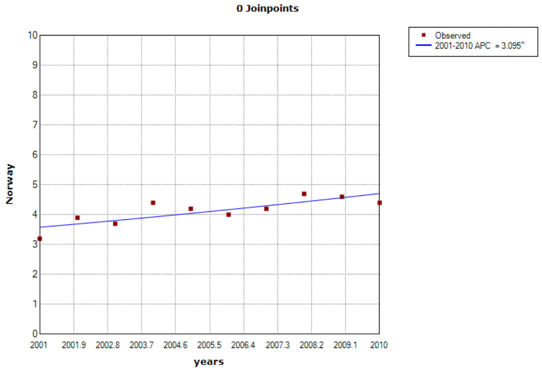
0 Joinpoints
Observed
2001-2010 APC = 3.095^
Norway
years

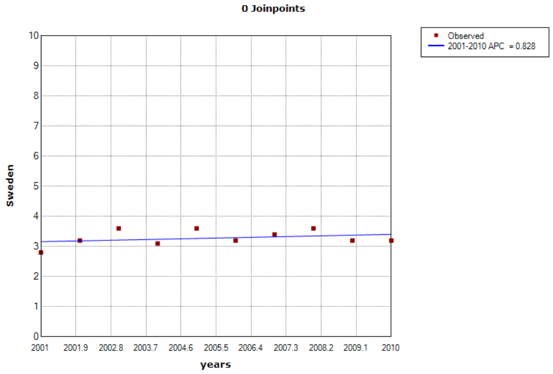
0 Joinpoints
Observed
2001-2010 APC = 0.828
Sweden
years

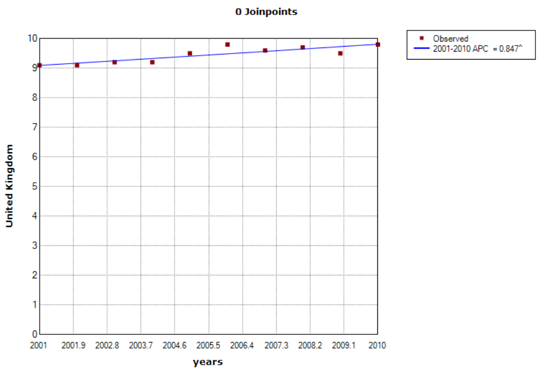

Western Europe

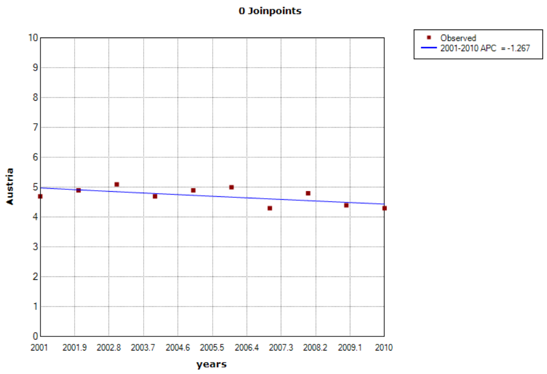

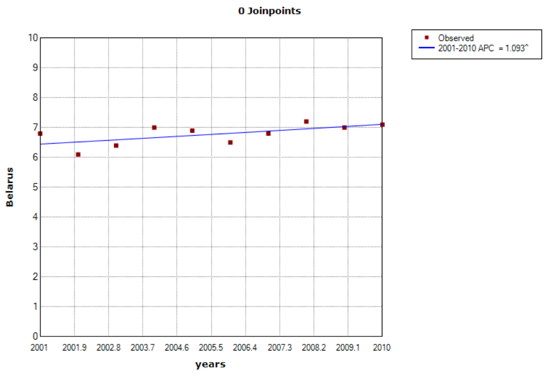

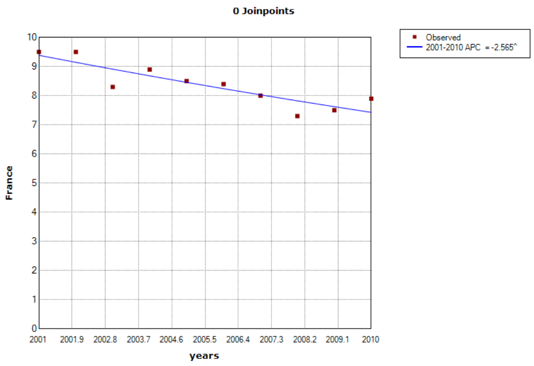

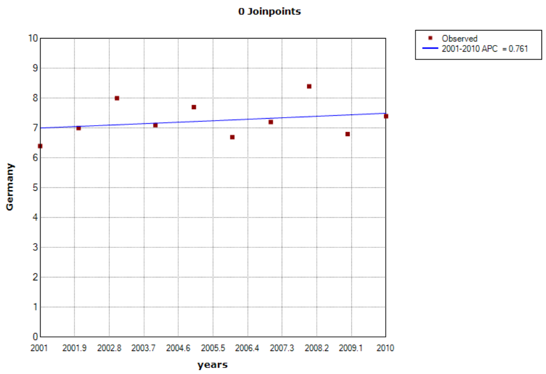

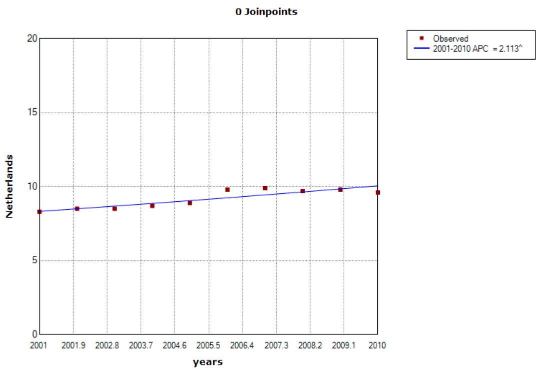

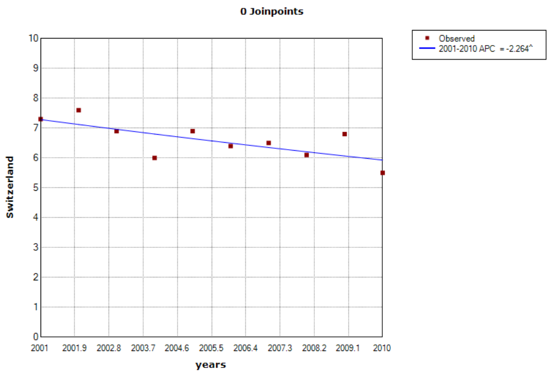

Southern Europe

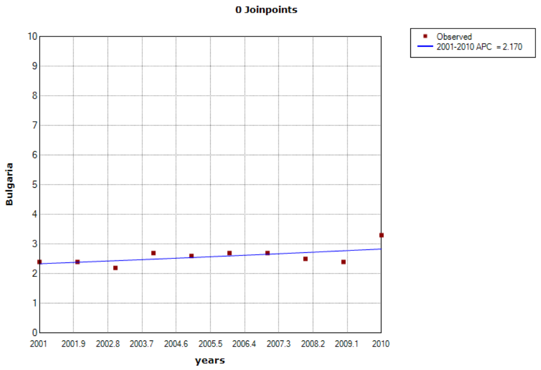

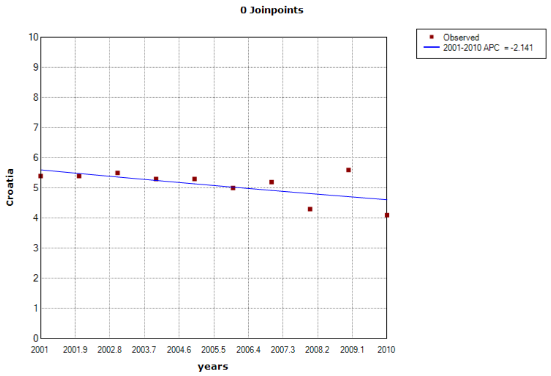

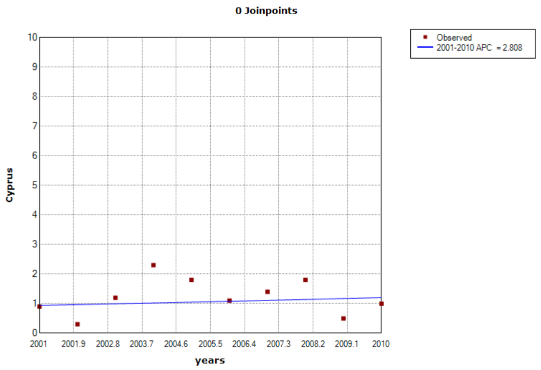

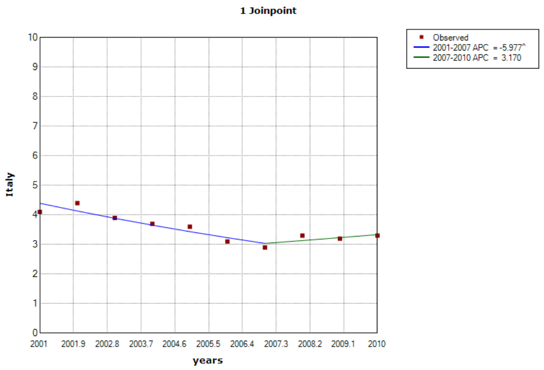

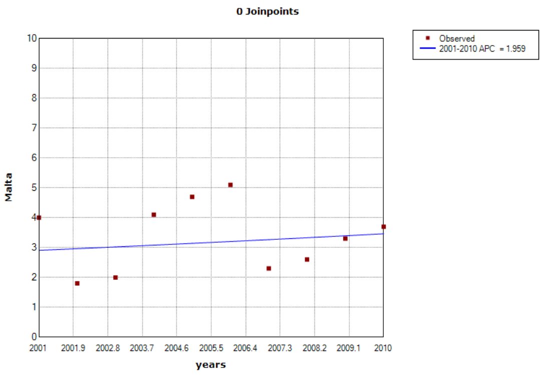

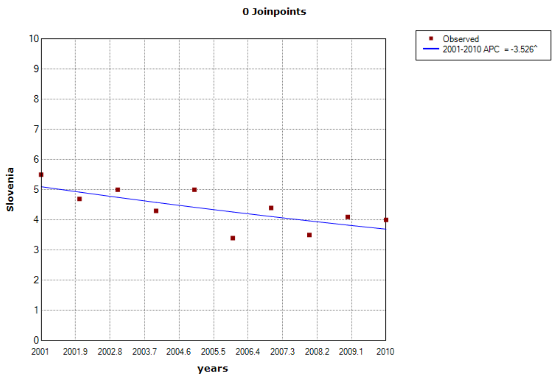

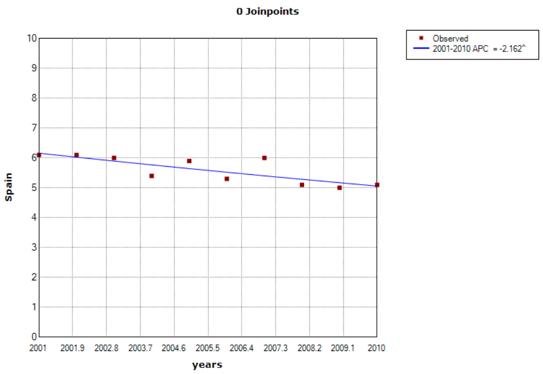

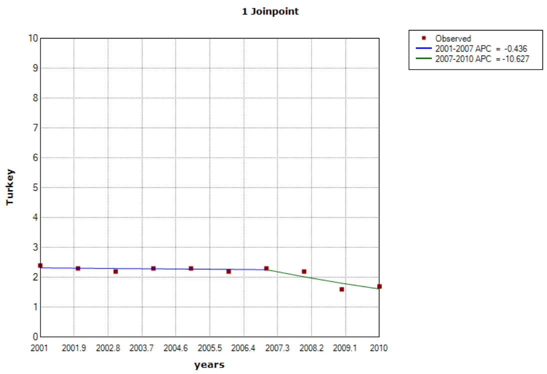

Eastern Europe

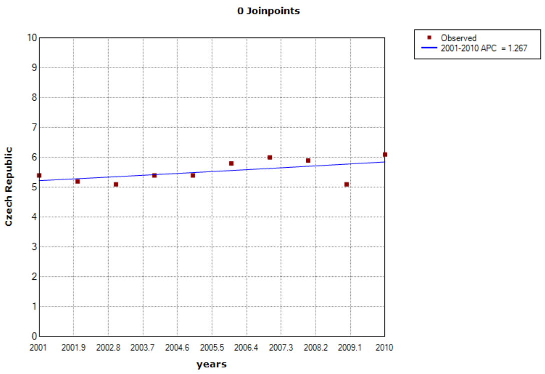

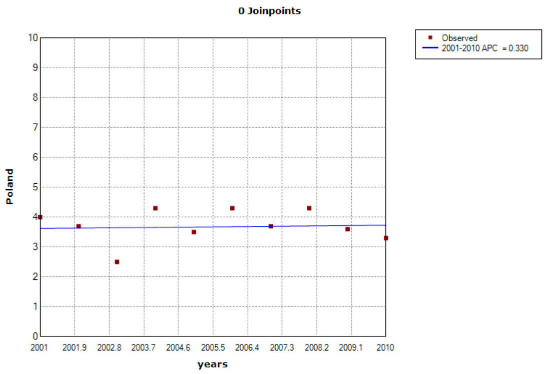

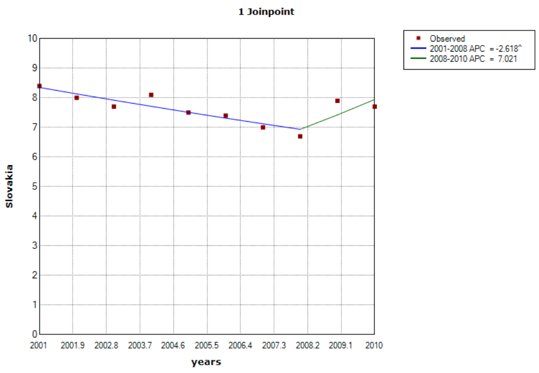
1 Joinpoint
Observed
2001-2008 APC = -2.618^
2008-2010 APC = 7.021
Slovakia
years
2001
2001.9
2002.8
2003.7
2004.6
2005.5
2006.4
2007.3
2008.2
2009.1
2010

Africa

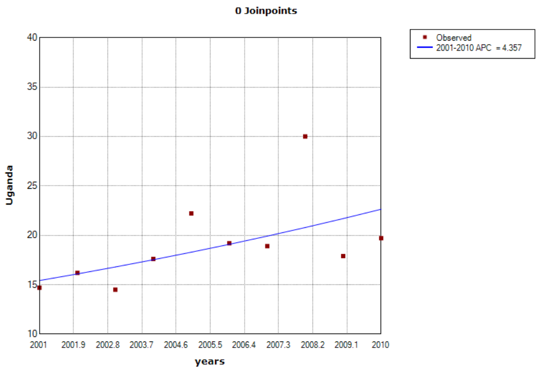
0 Joinpoints
Observed
2001-2010 APC = 4.357
Uganda
years
2001
2001.9
2002.8
2003.7
2004.6
2005.5
2006.4
2007.3
2008.2
2009.1
2010

b.) Incidence female aged 0 to 85 years old

Asia

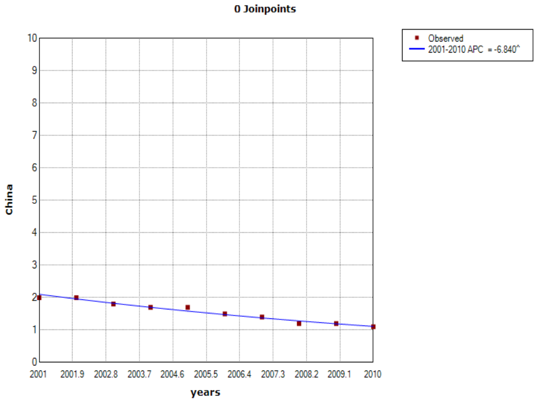

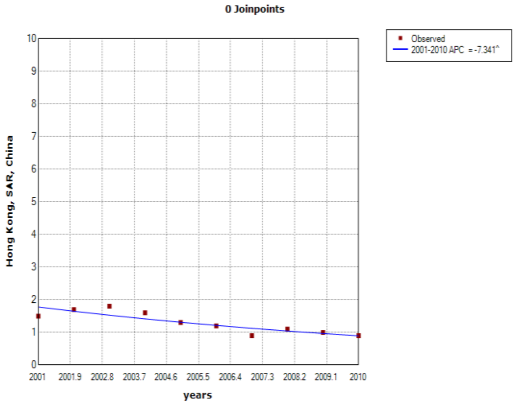

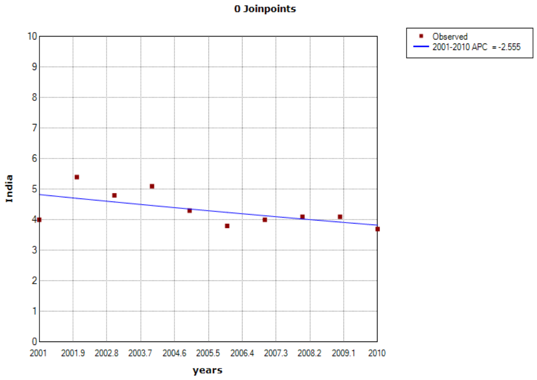

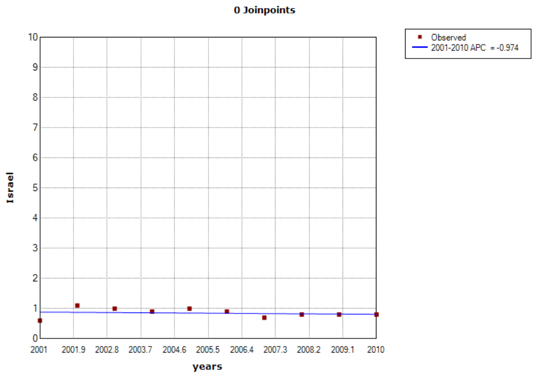

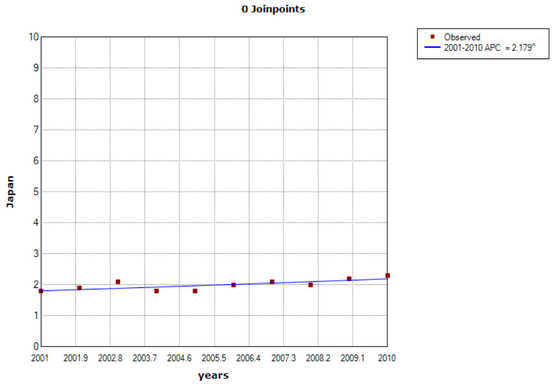

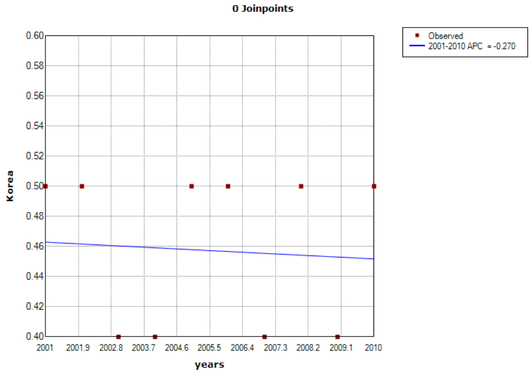

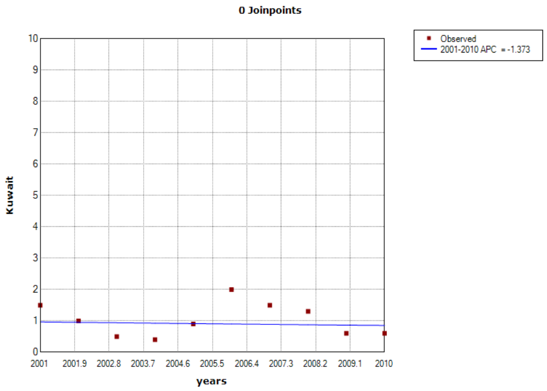
0 Joinpoints
Observed
2001-2010 APC = -1.373
Kuwait
years

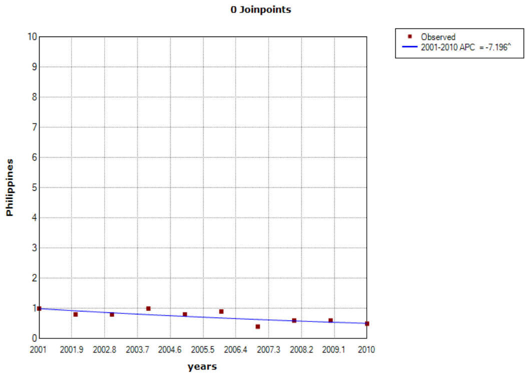
0 Joinpoints
Observed
2001-2010 APC = -7.196^
Philippines
years

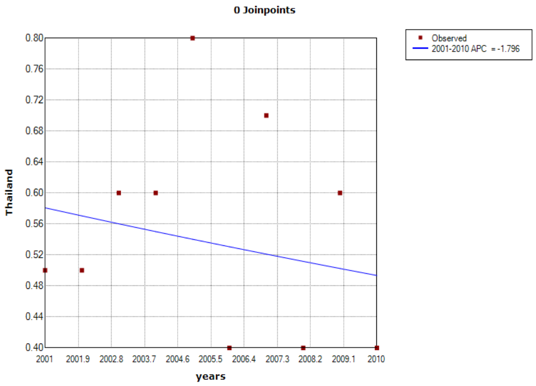
0 Joinpoints
Observed
2001-2010 APC = -1.796
Thailand
years

Oceania

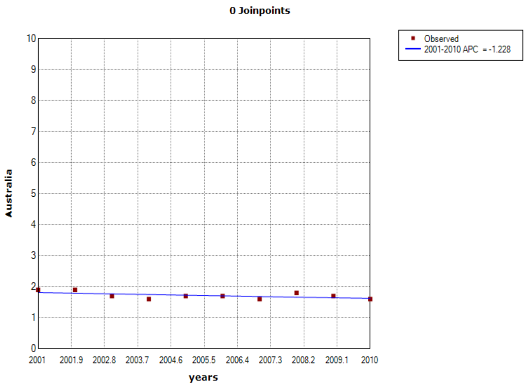
0 Joinpoints
Observed
2001-2010 APC = -1.228
Australia
years

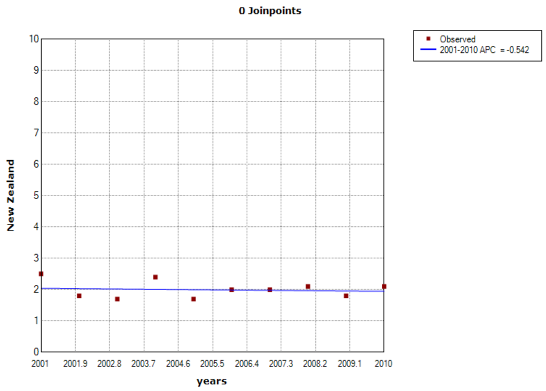
0 Joinpoints
Observed
2001-2010 APC = -0.542
New Zealand
years

Northern America

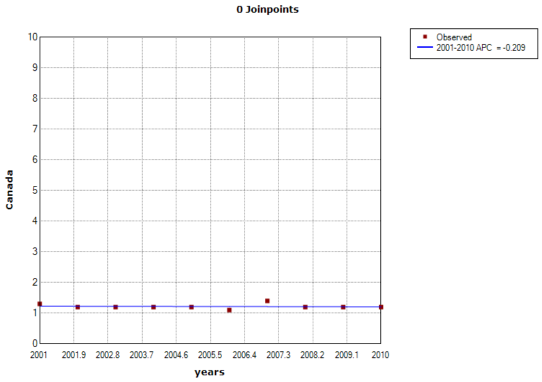

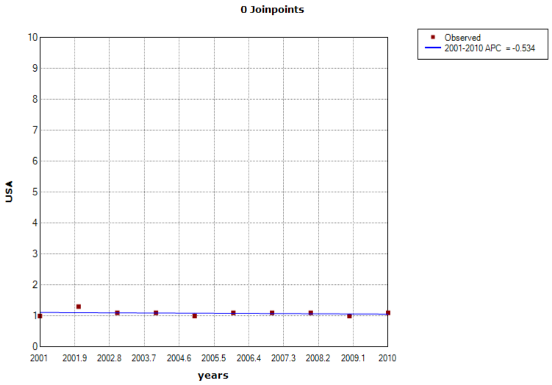

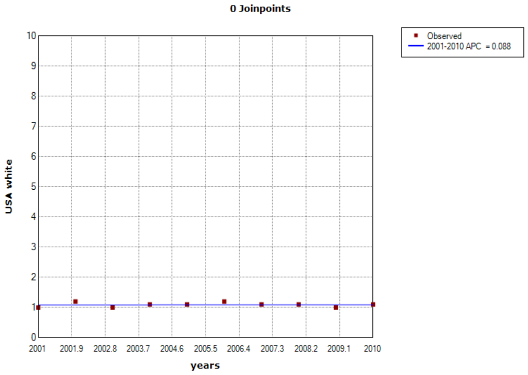

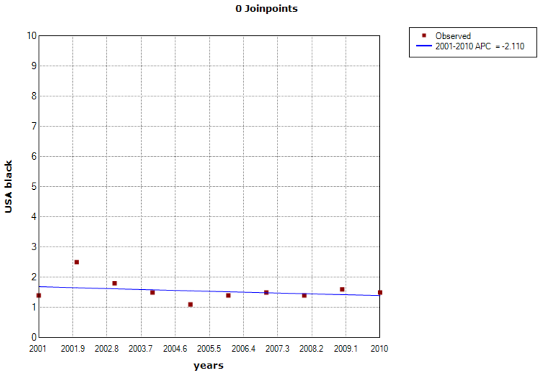

Southern America

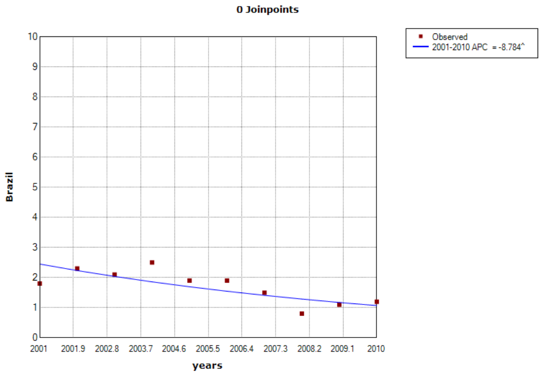

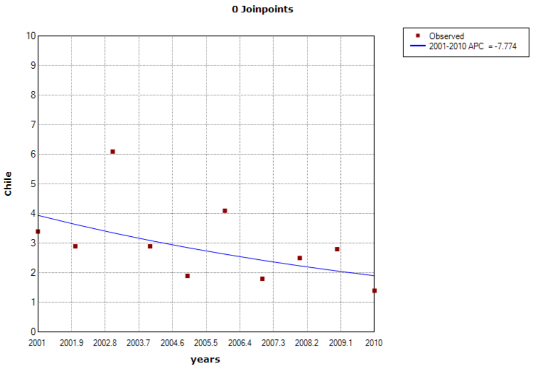

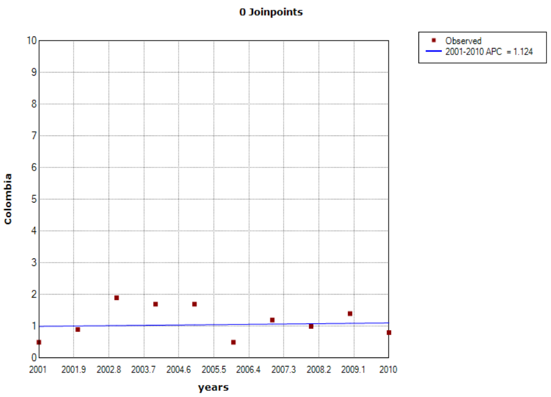

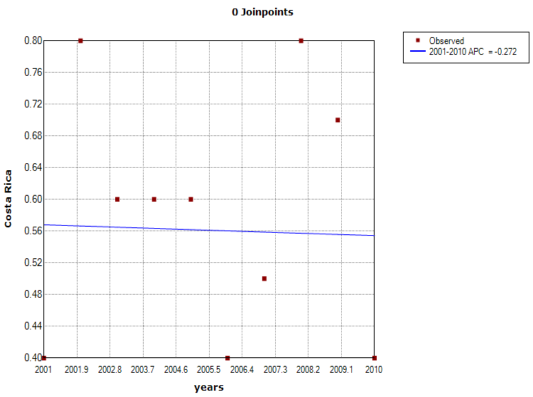

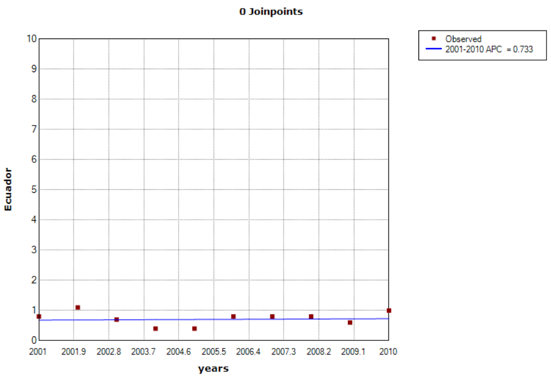

Northern Europe

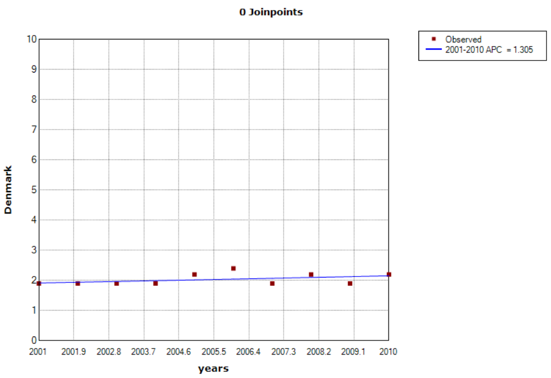

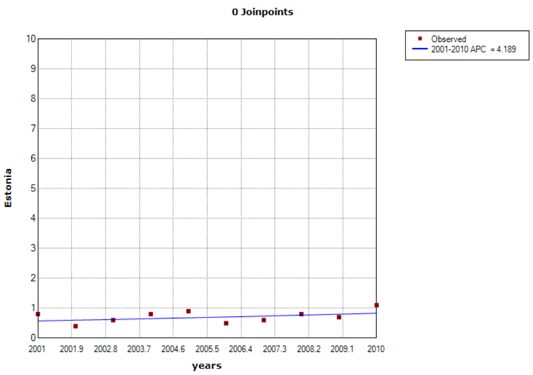

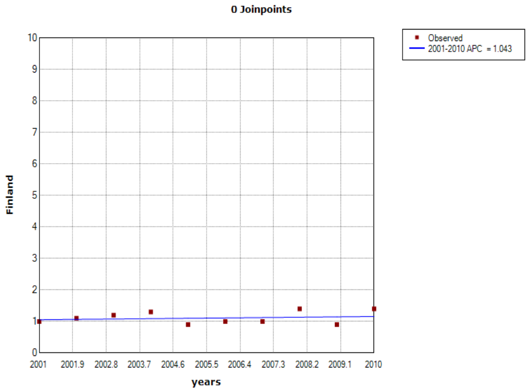
0 Joinpoints
Observed
2001-2010 APC = 1.043
Finland
years

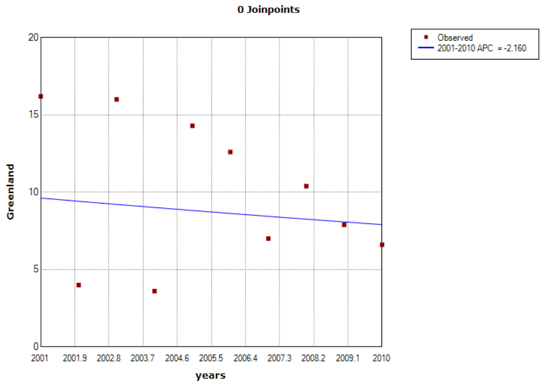
0 Joinpoints
Observed
2001-2010 APC = -2.160
Greenland
years

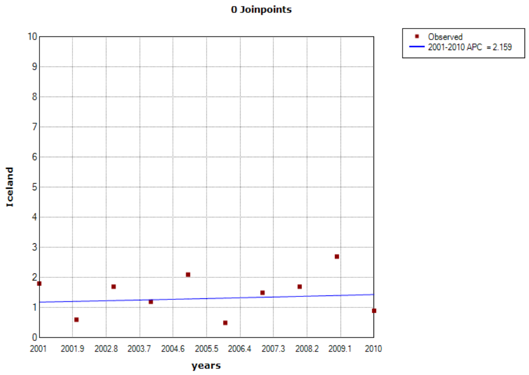
0 Joinpoints
Observed
2001-2010 APC = 2.159
Iceland
years

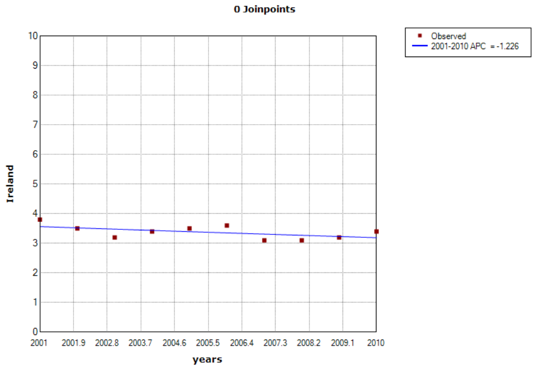
0 Joinpoints
Observed
2001-2010 APC = -1.226
Ireland
years

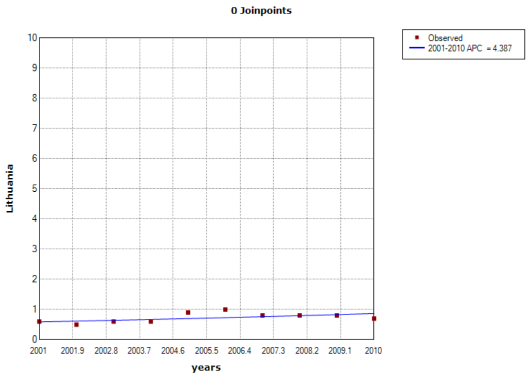
0 Joinpoints
Observed
2001-2010 APC = 4.387
Lithuania
years

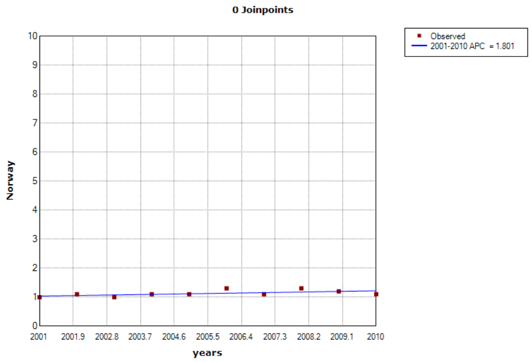
0 Joinpoints
Observed
2001-2010 APC = 1.801
Norway
years

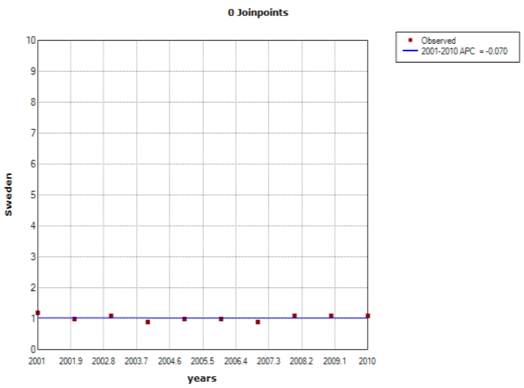

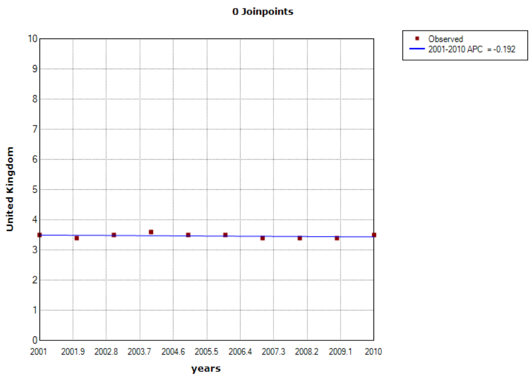

Western Europe

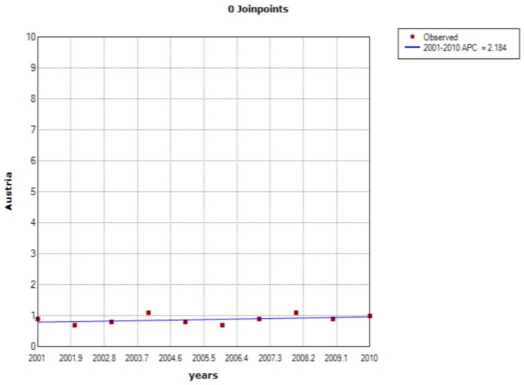

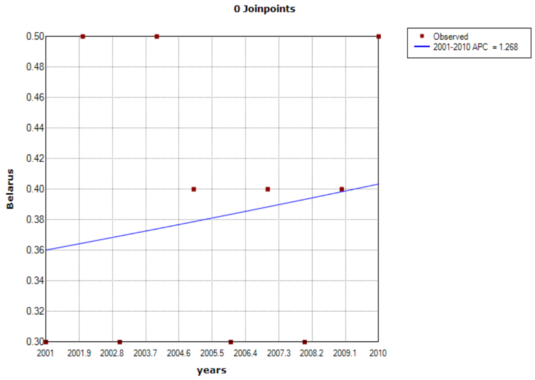

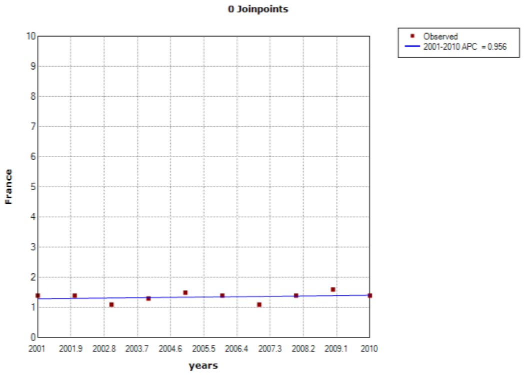

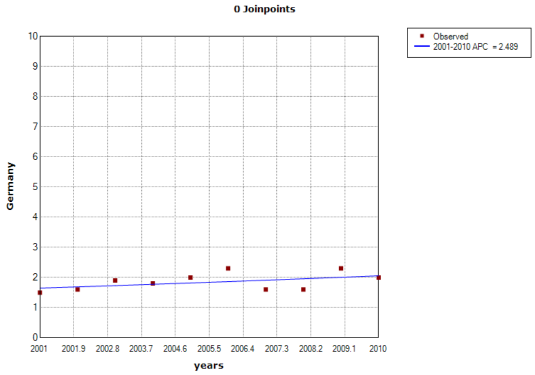

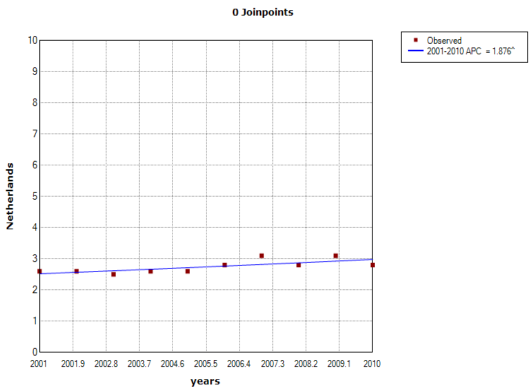

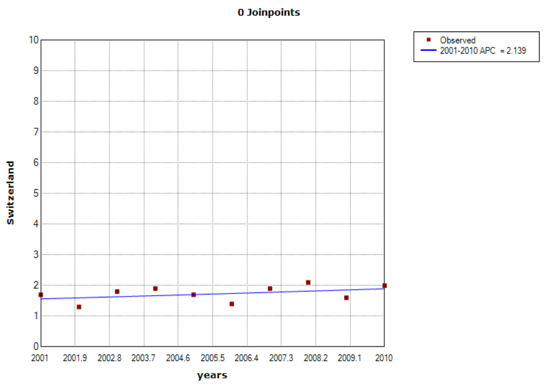

Southern Europe

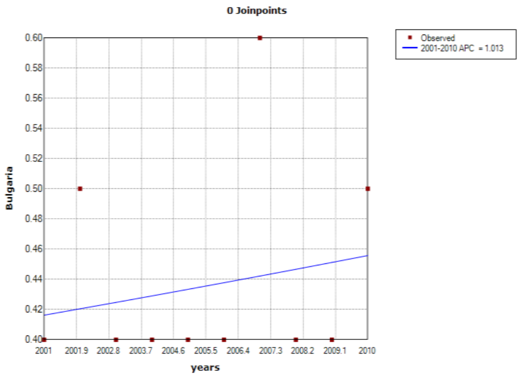

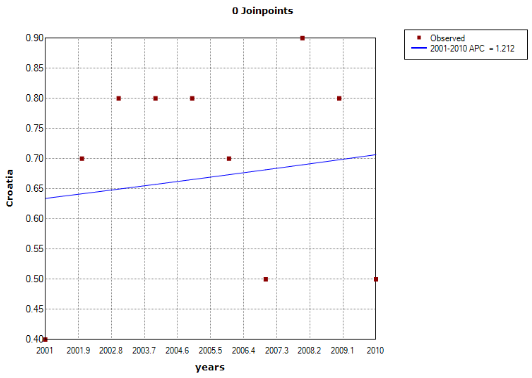

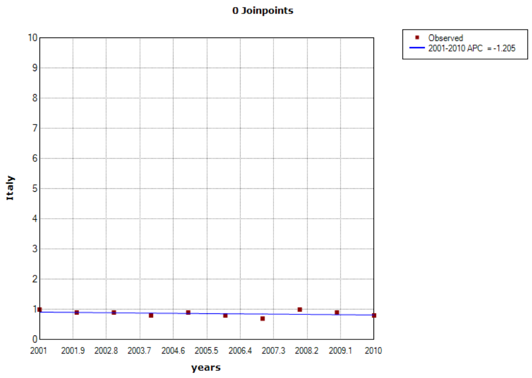

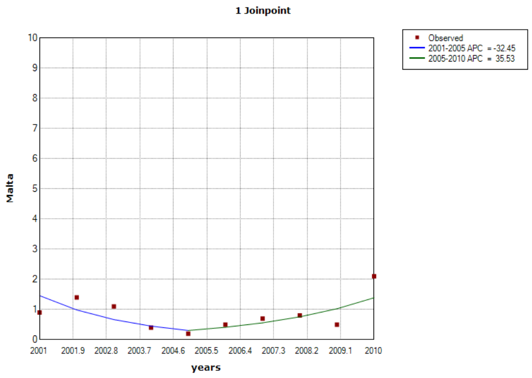

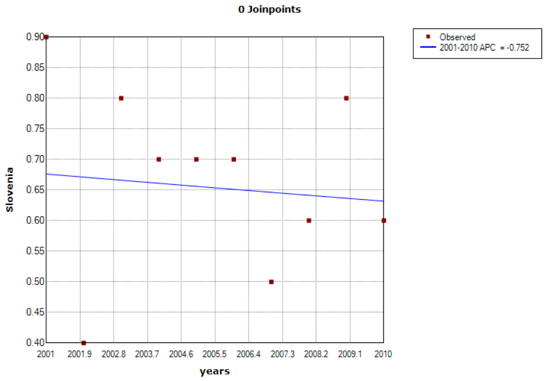

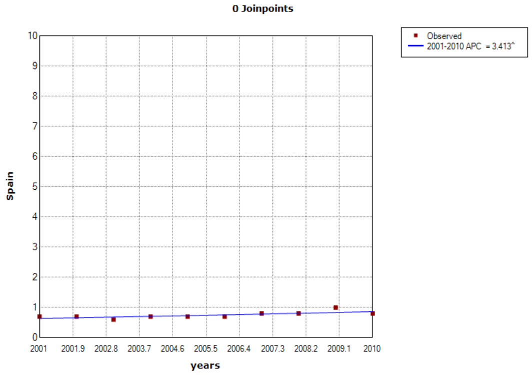

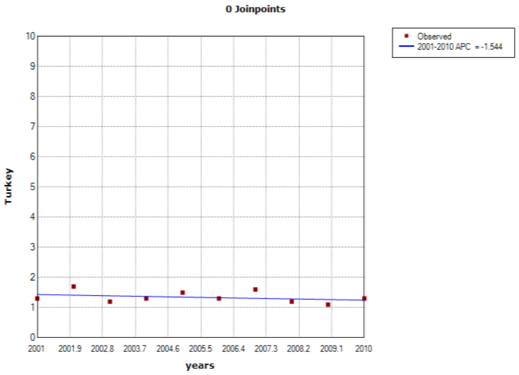

Eastern Europe

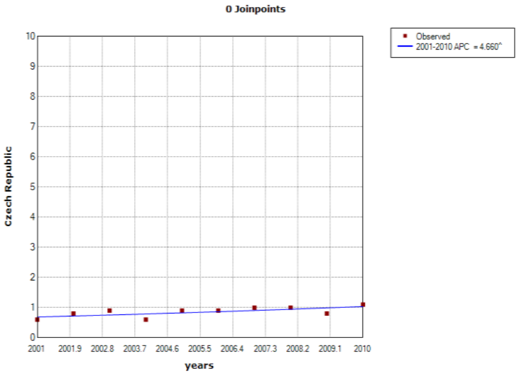

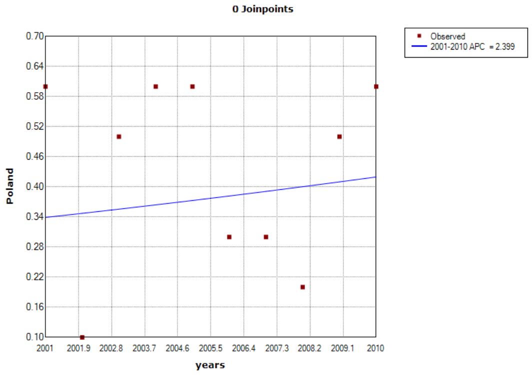

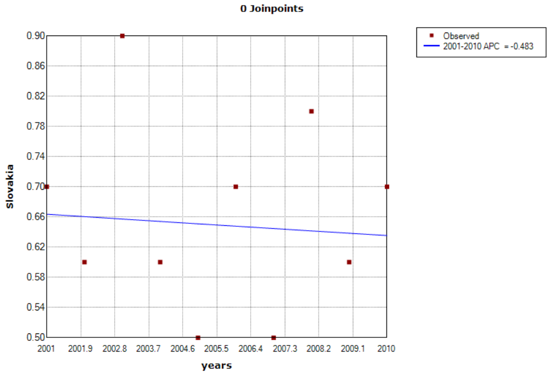
0 Joinpoints
Observed
2001-2010 APC = -0.483
Slovakia
0.90
0.86
0.82
0.78
0.74
0.70
0.66
0.62
0.58
0.54
0.50
2001
2001.9
2002.8
2003.7
2004.6
2005.5
2006.4
2007.3
2008.2
2009.1
2010
years

Africa

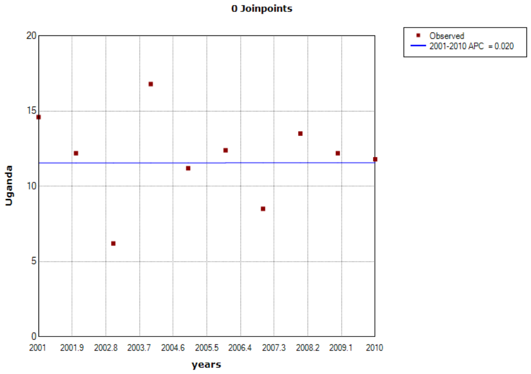
0 Joinpoints
Observed
2001-2010 APC = 0.020
Uganda
20
15
10
5
0
2001
2001.9
2002.8
2003.7
2004.6
2005.5
2006.4
2007.3
2008.2
2009.1
2010
years

c.) Incidence male aged below 40 years old

Asia

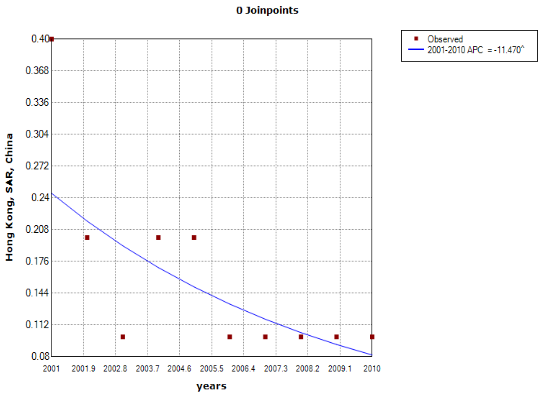

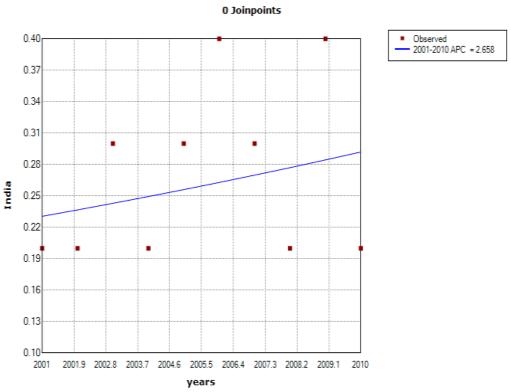

Northern America

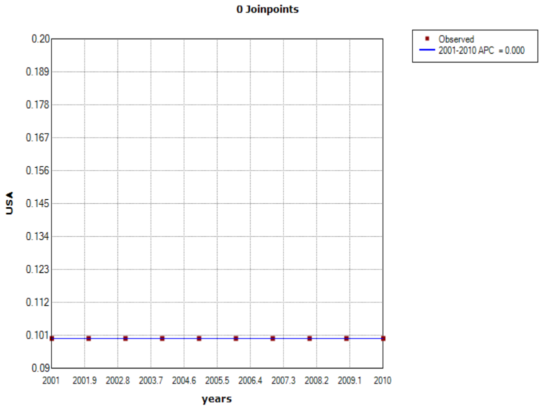

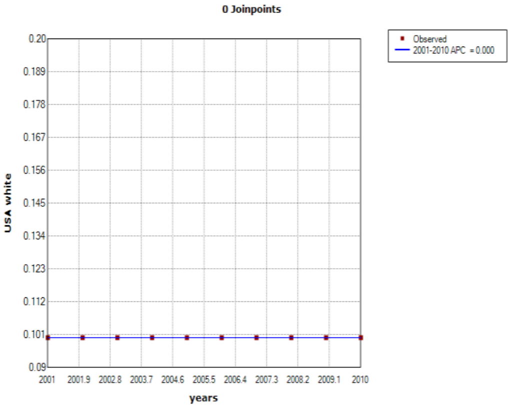

Northern Europe

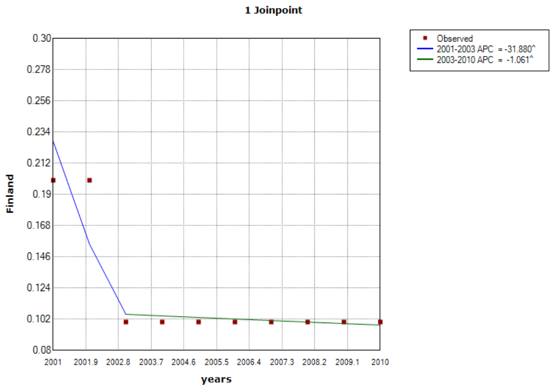

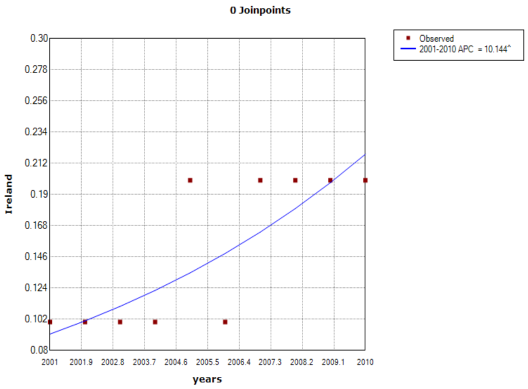

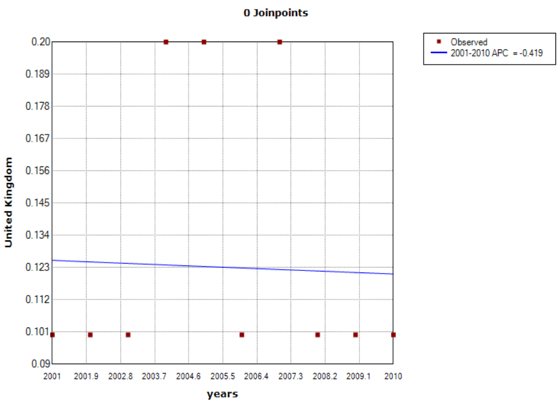

Western Europe

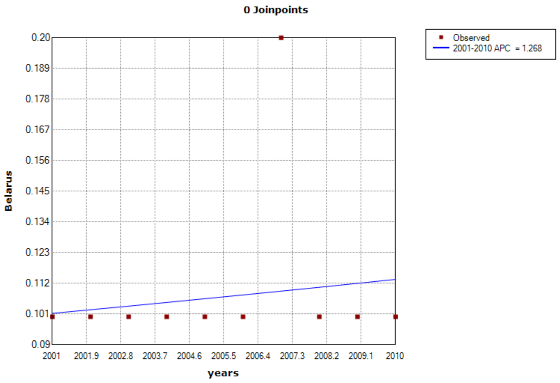

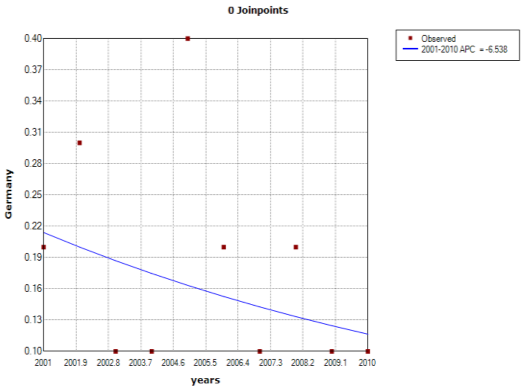

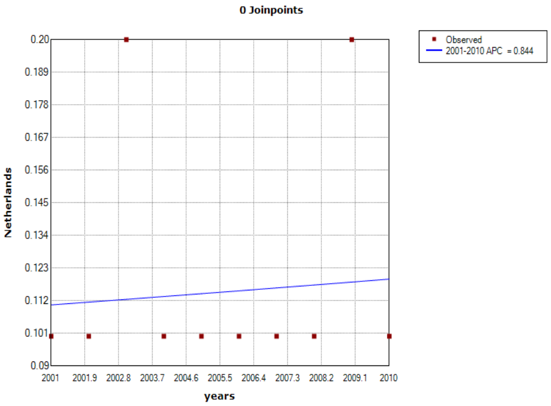

Southern Europe

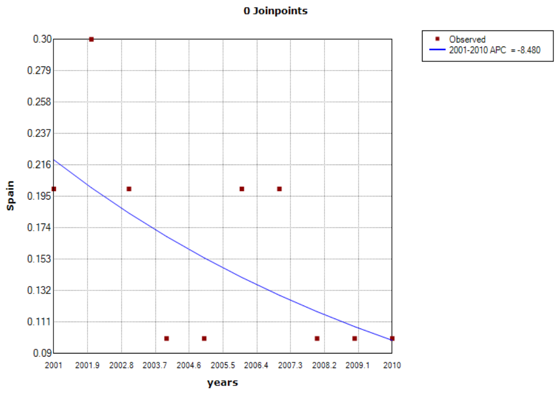

Eastern Europe

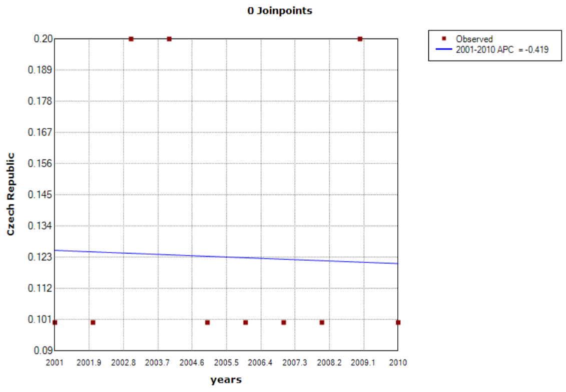

Africa

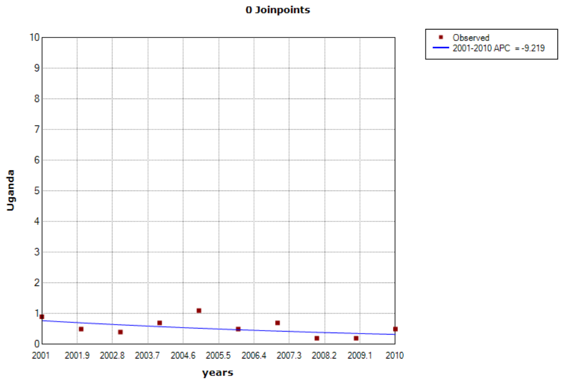

d.) Incidence female aged below 40 years old

Asia

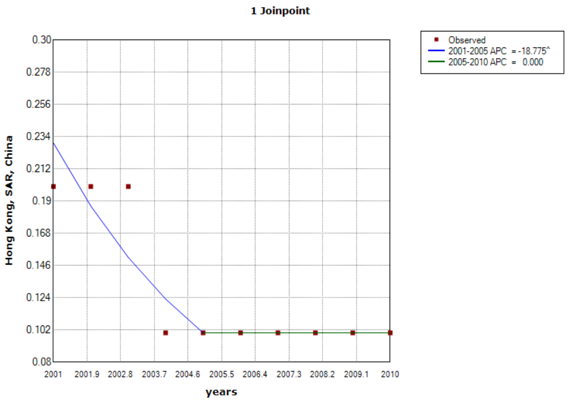

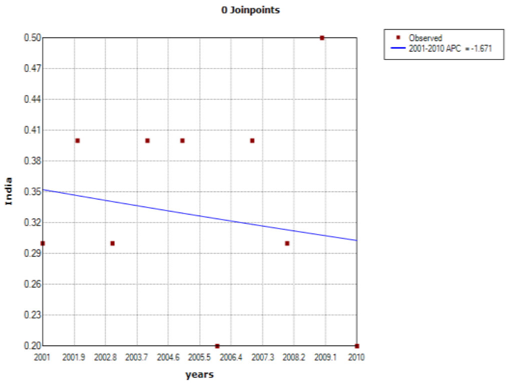

Southern Europe

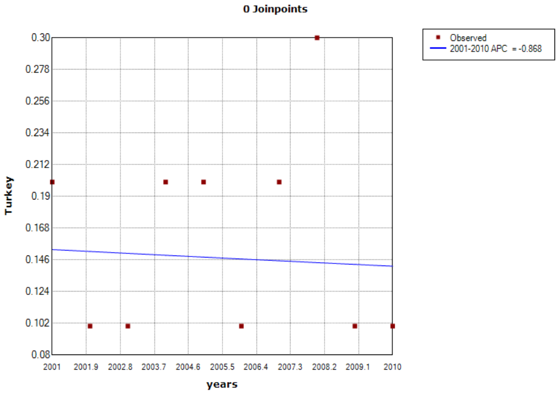

e.) Incidence male aged below 50 years old

Asia

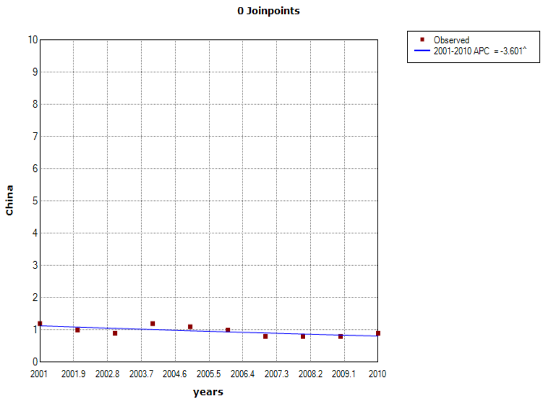

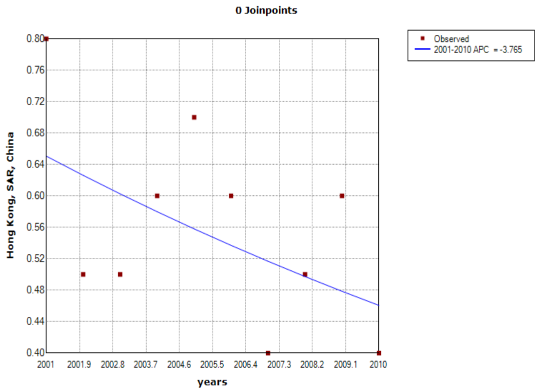

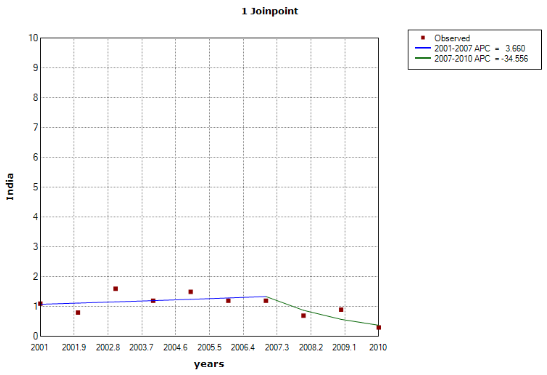

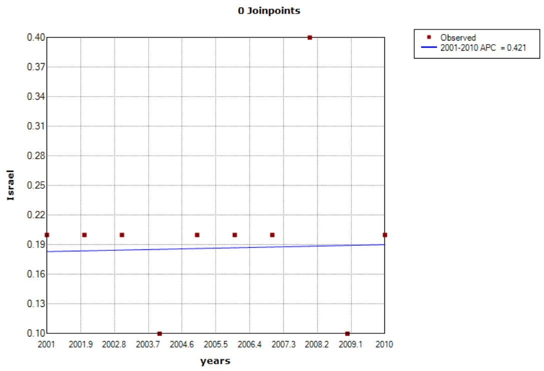

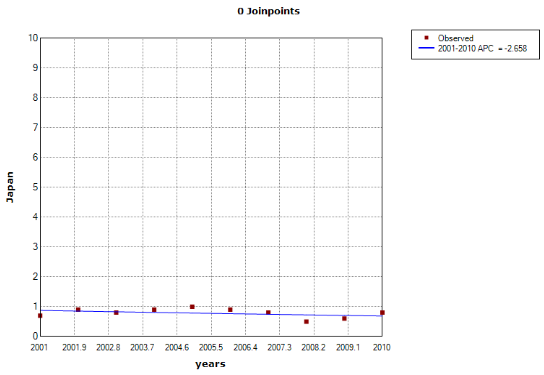

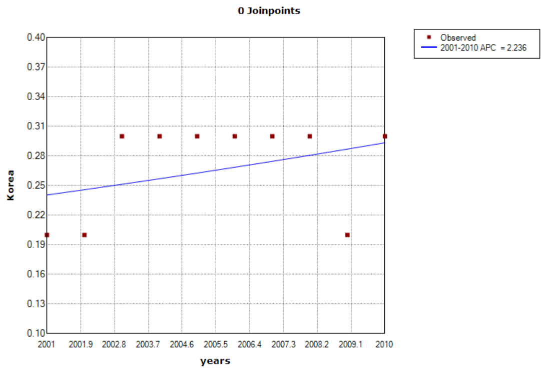

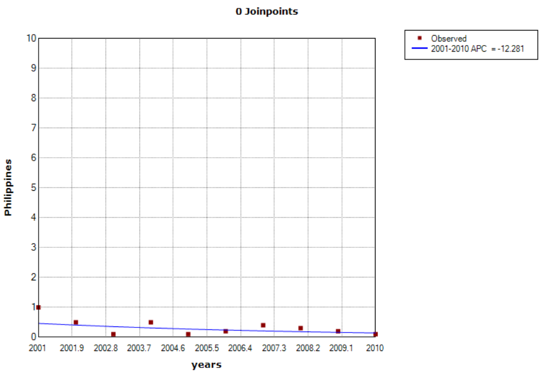

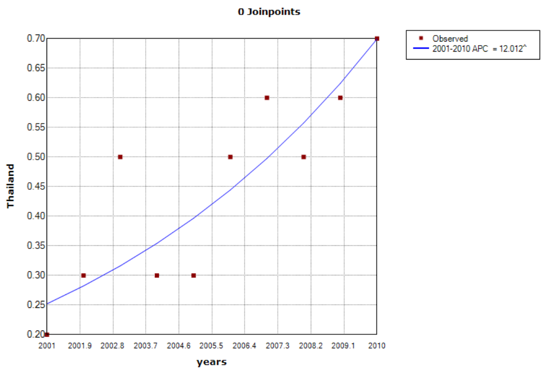

Oceania

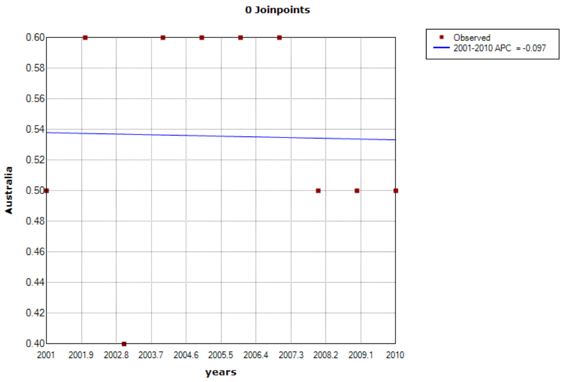

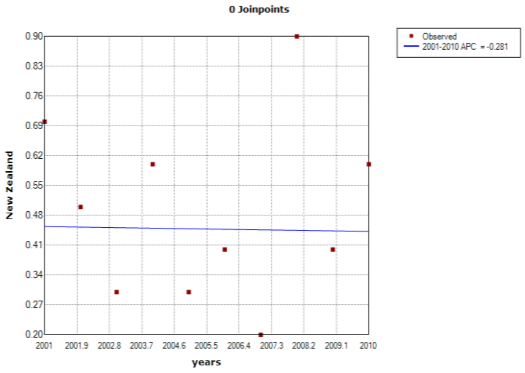

Northern America

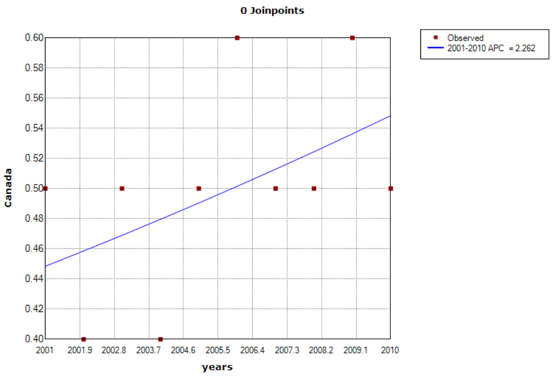

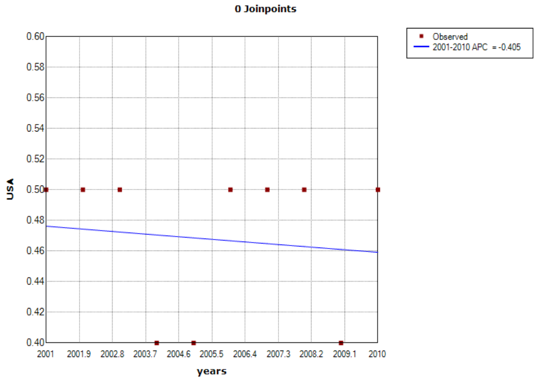

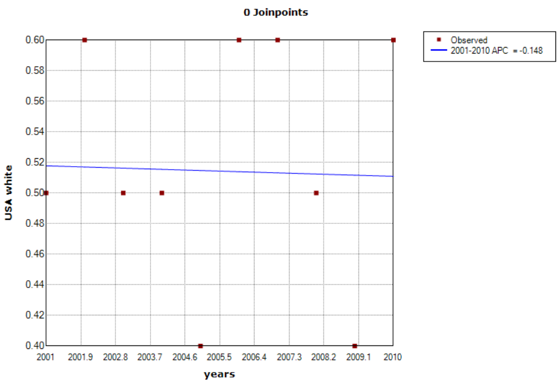

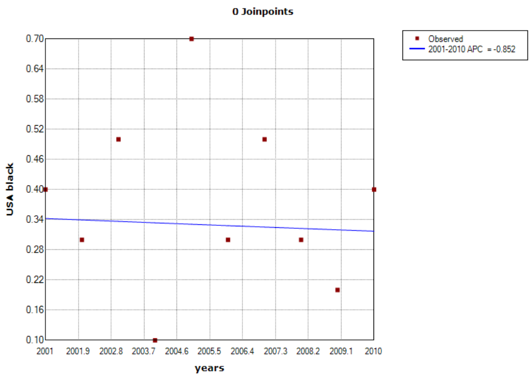

Southern America

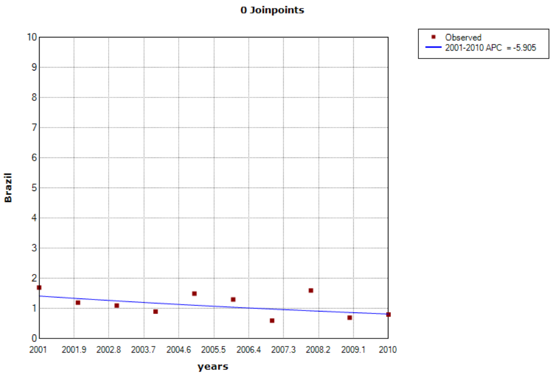

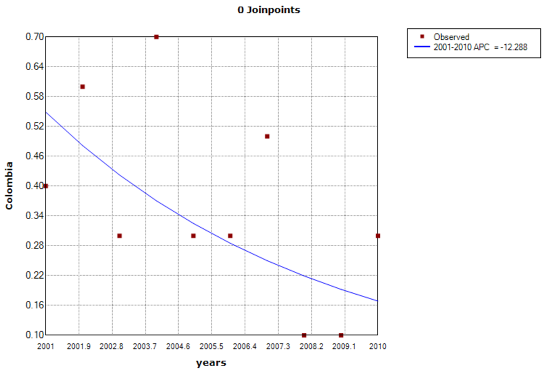

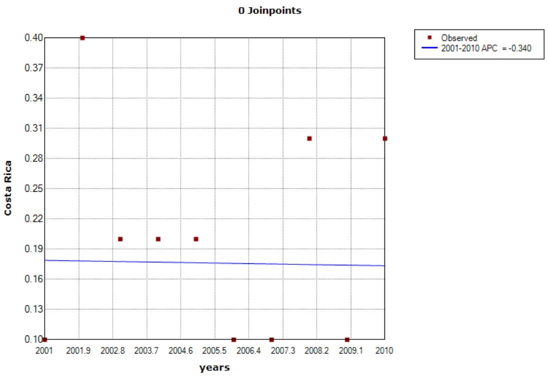

Northern Europe

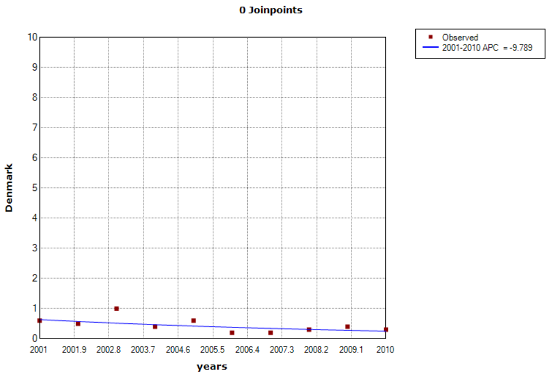

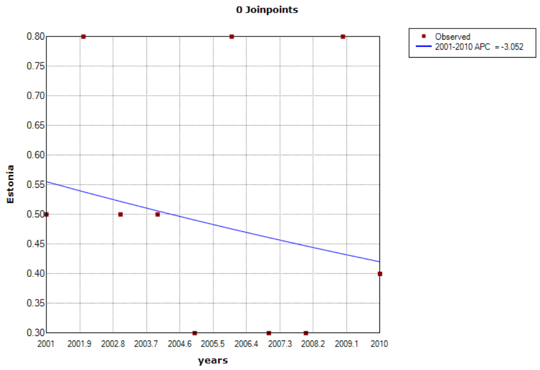

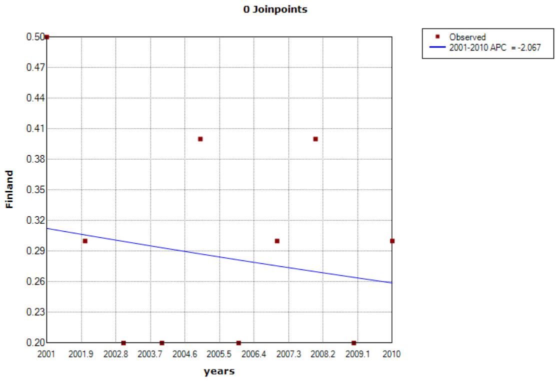

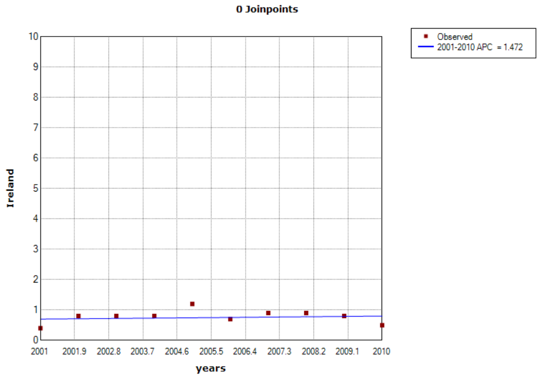

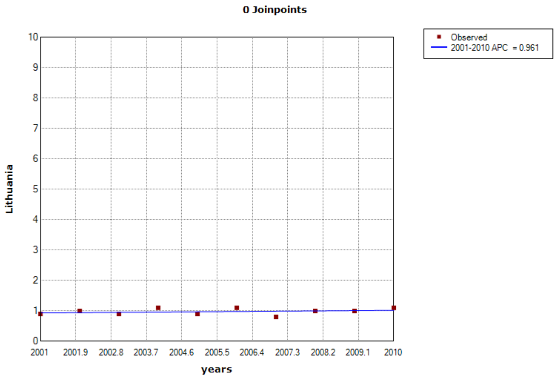

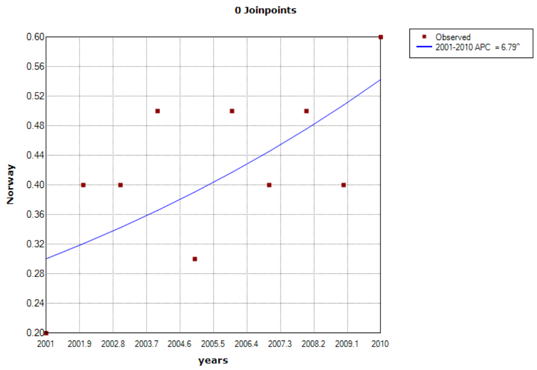

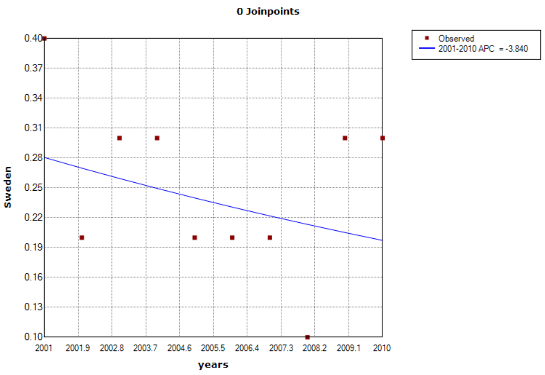

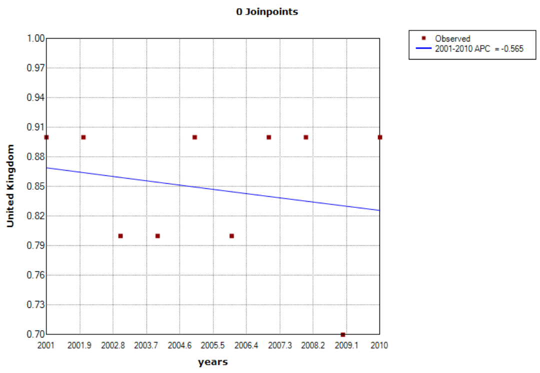

Western Europe

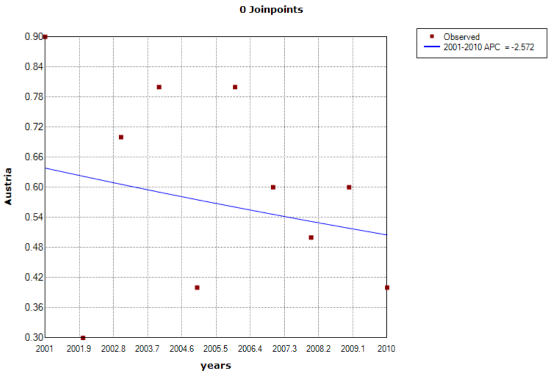

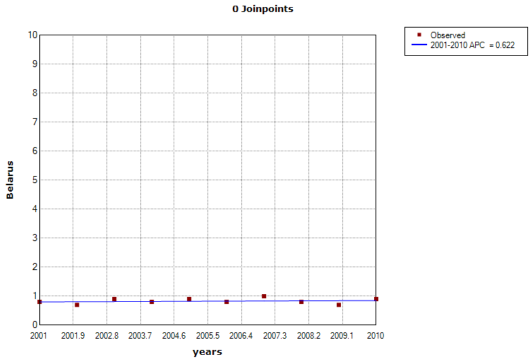

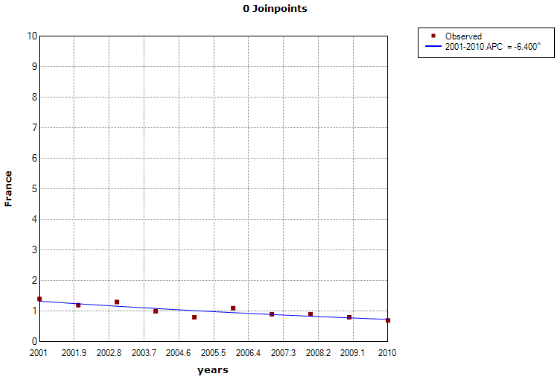

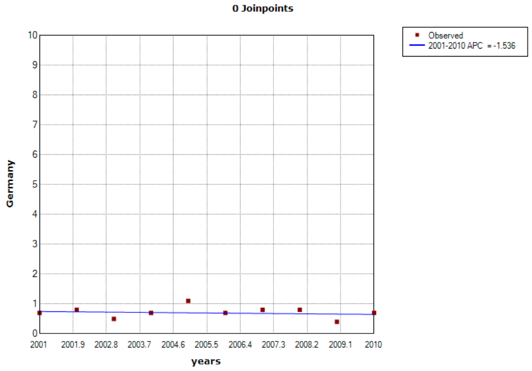

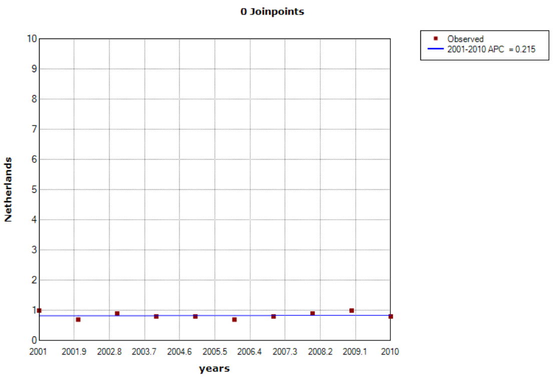

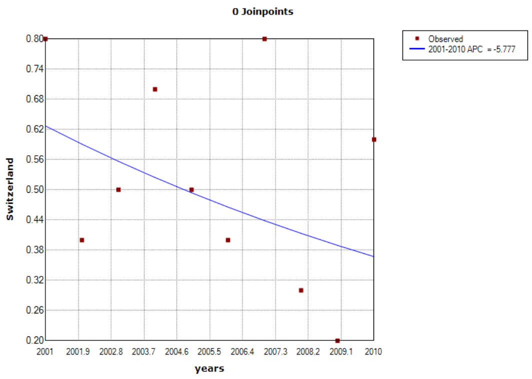

Southern Europe

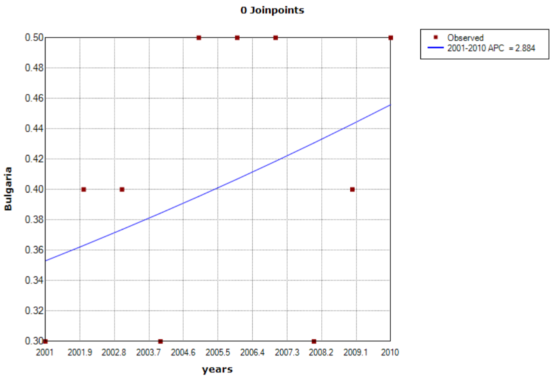

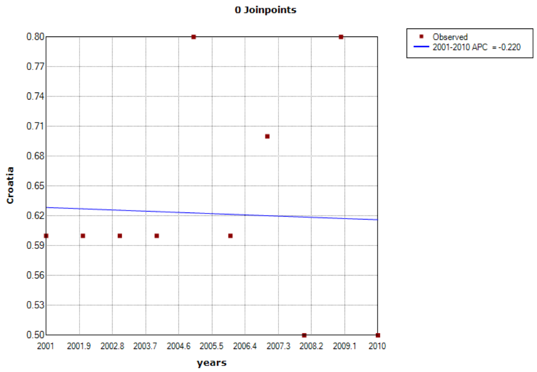

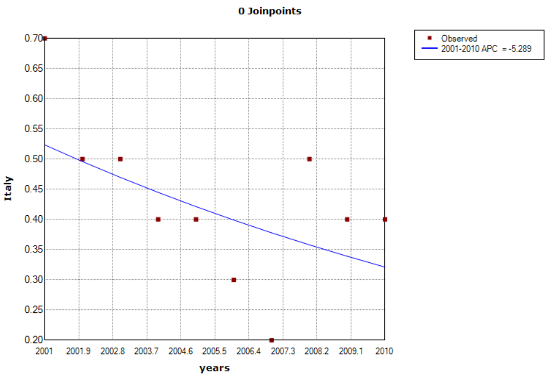

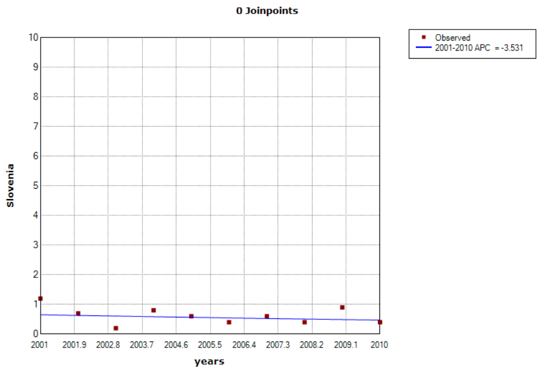

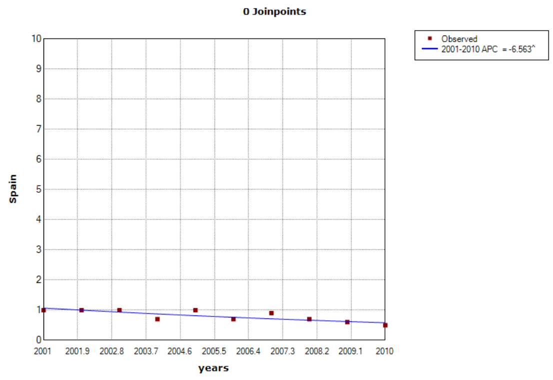

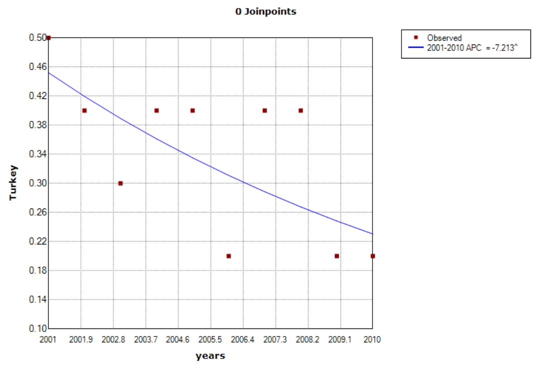

Eastern Europe

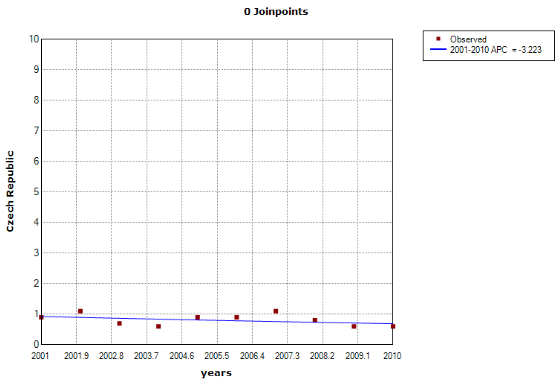

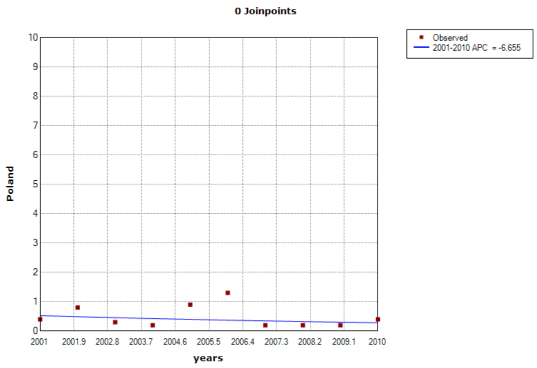

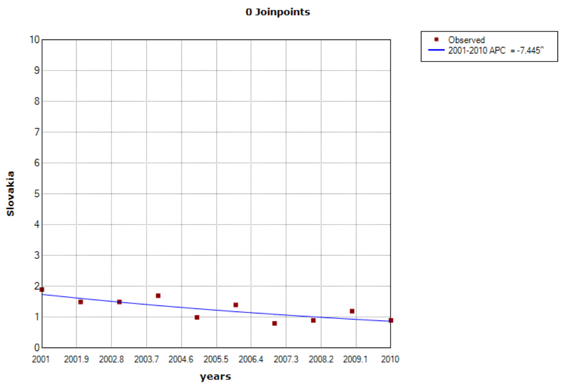

Africa

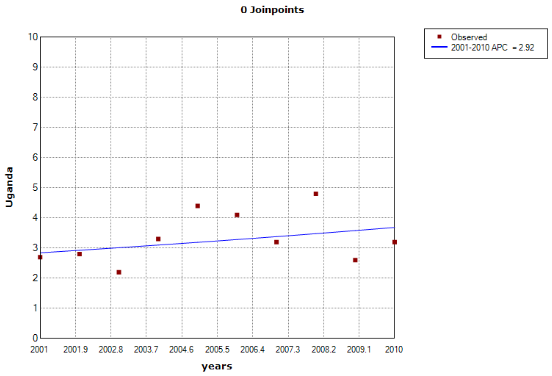
0 Joinpoints
Observed
2001-2010 APC = 2.92
Uganda
years
10
9
8
7
6
5
4
3
2
1
0
2001
2001.9
2002.8
2003.7
2004.6
2005.5
2006.4
2007.3
2008.2
2009.1
2010

f.) Incidence female aged below 50 years old

Asia

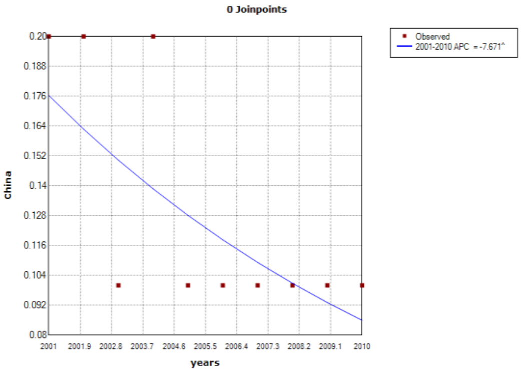

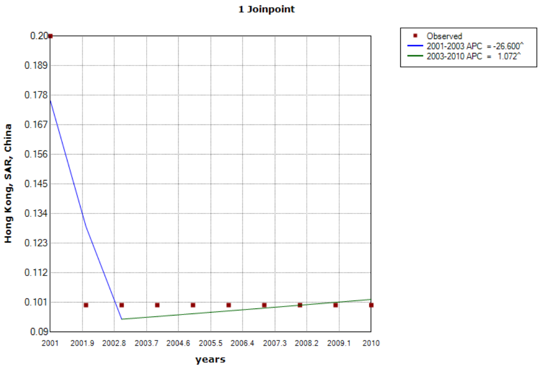

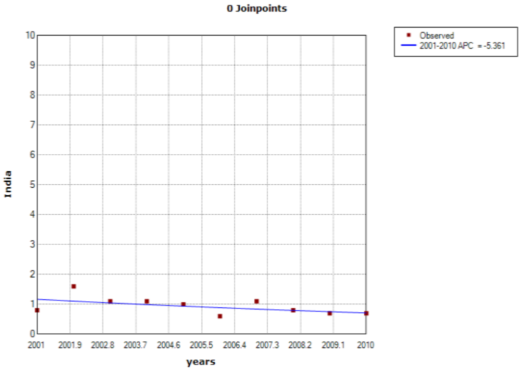

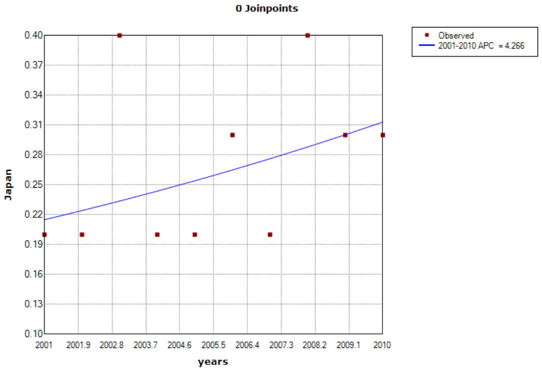

Oceania

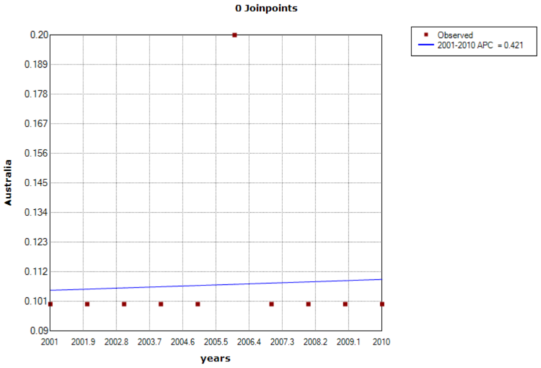

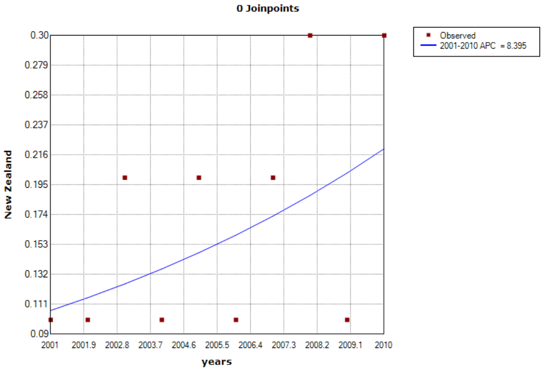

Northern America

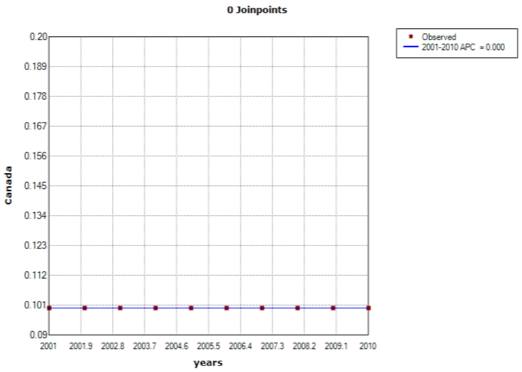

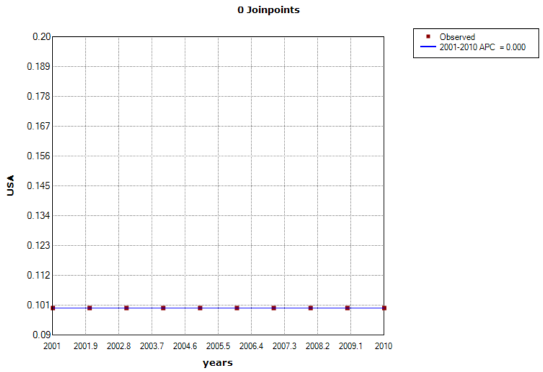

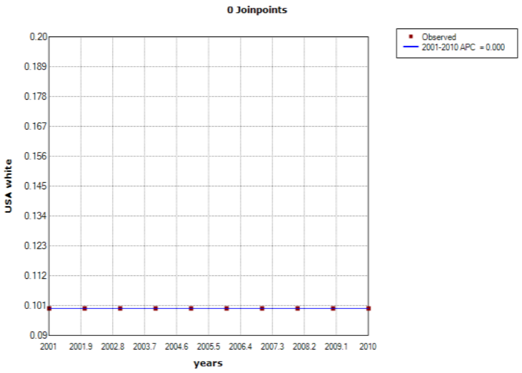

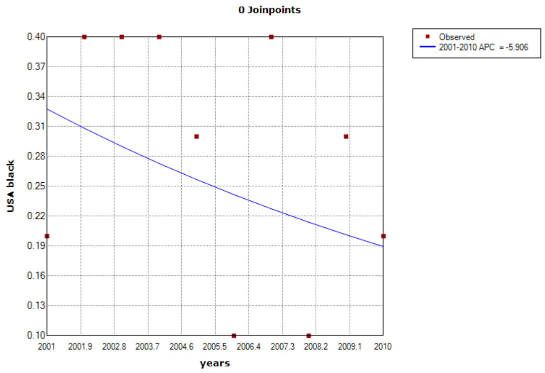

Northern Europe

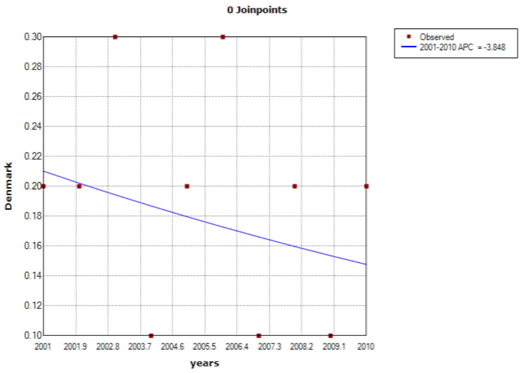

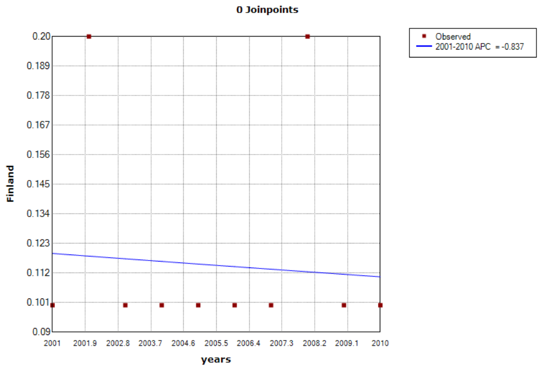

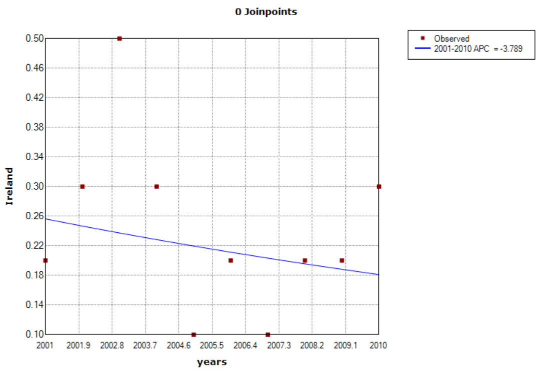

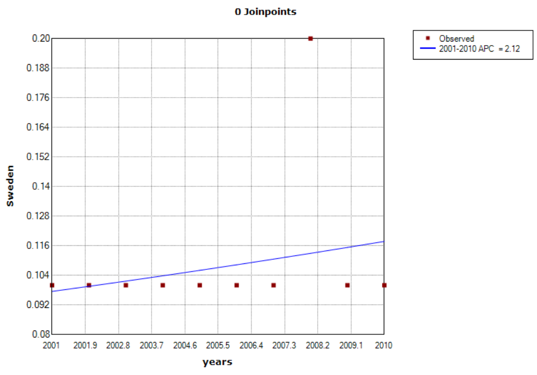

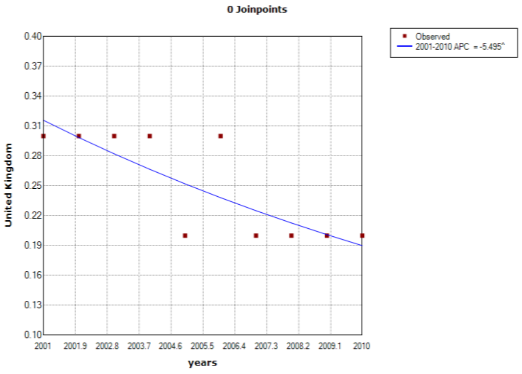

Western Europe

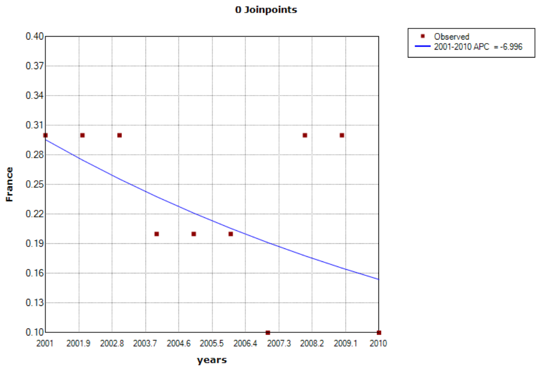

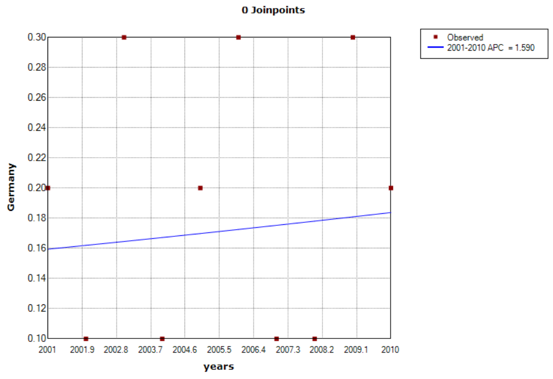

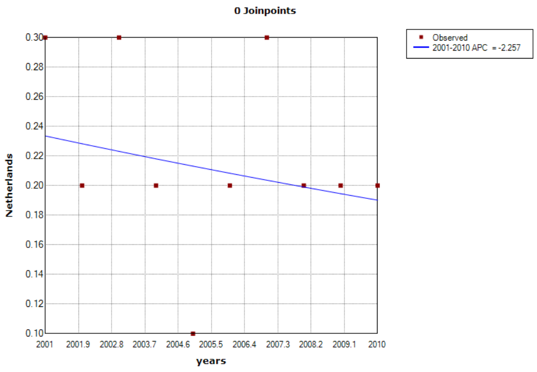

Southern Europe

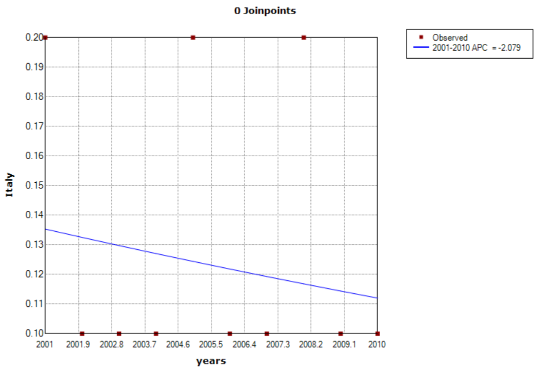

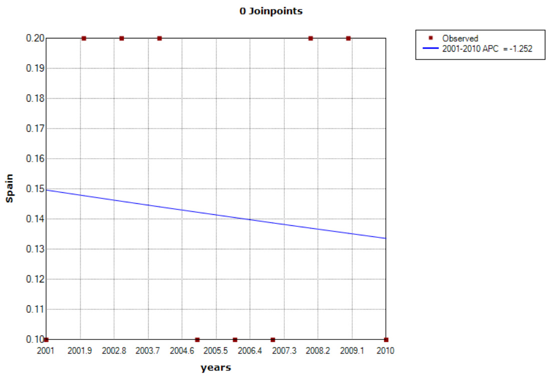

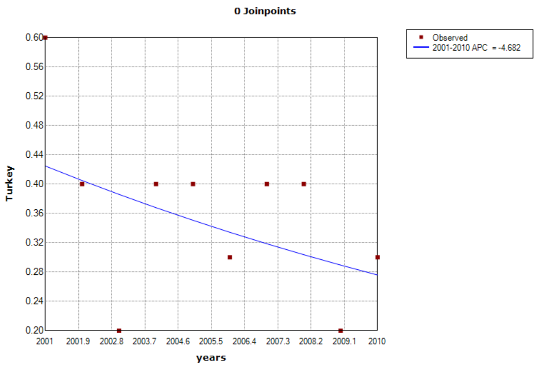

Eastern Europe

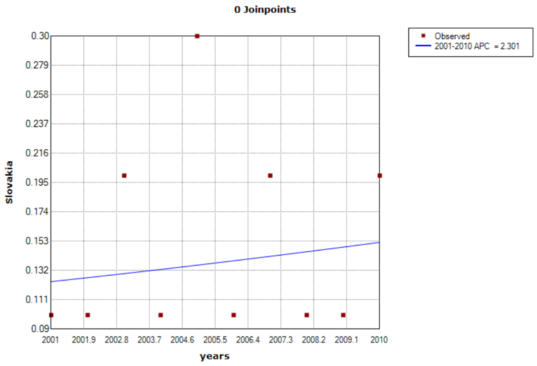

Africa

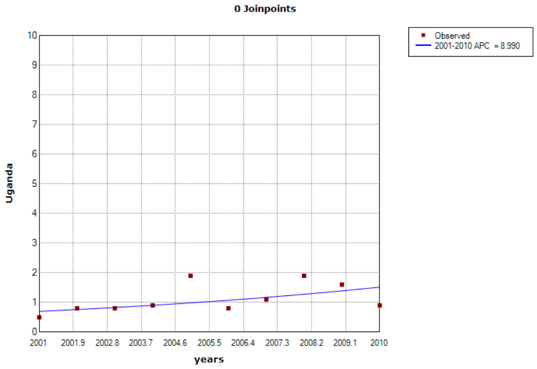

g.) Incidence male aged above 50 years old

Asia

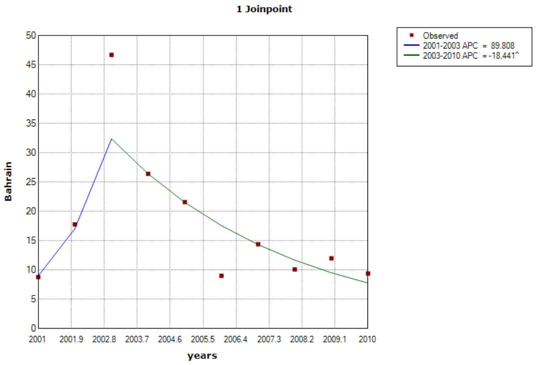

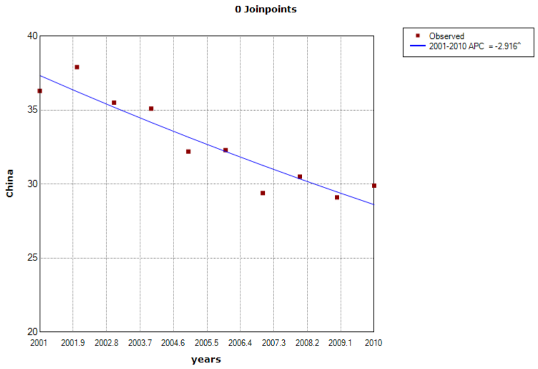

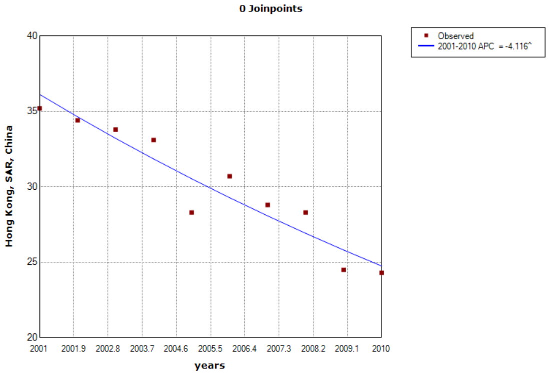

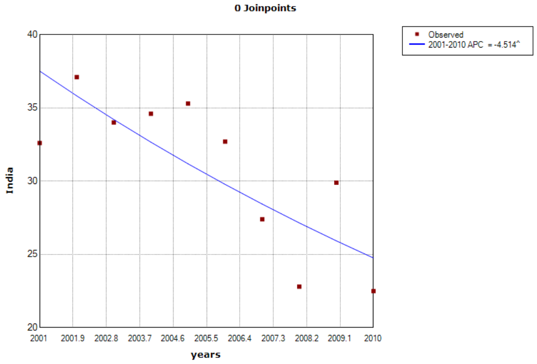

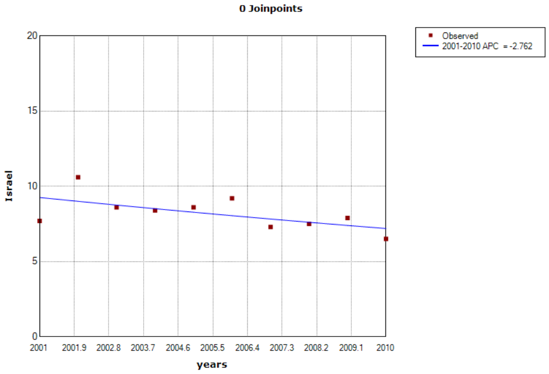

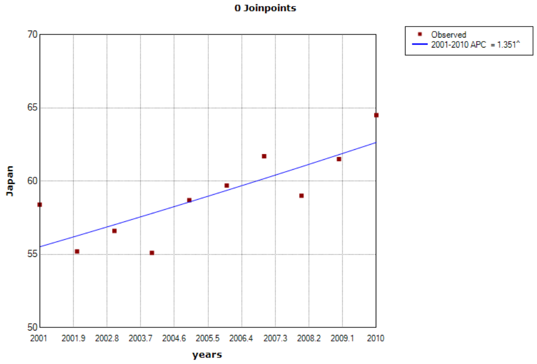

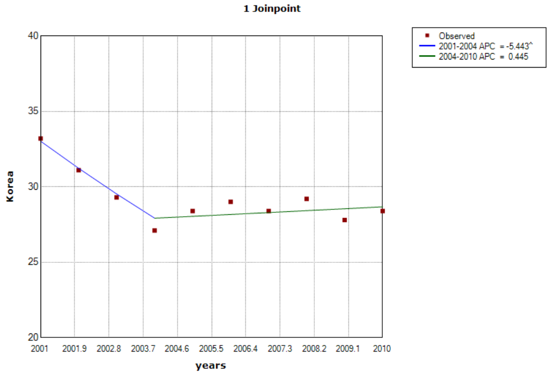
1 Joinpoint
Observed
2001-2004 APC = -5.443^
2004-2010 APC = 0.445
Korea
years

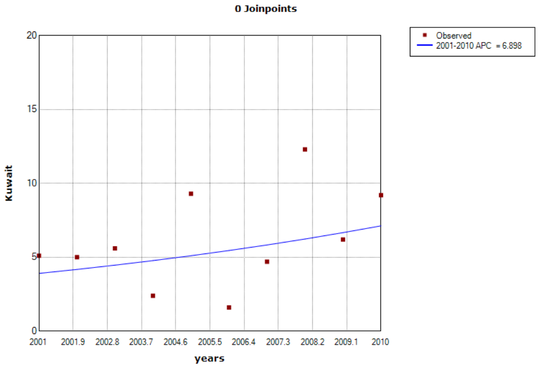
0 Joinpoints
Observed
2001-2010 APC = 6.898
Kuwait
years

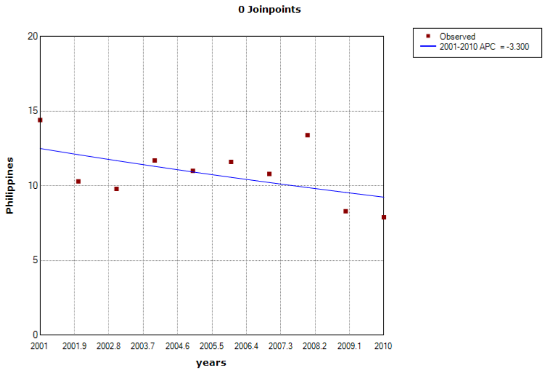
0 Joinpoints
Observed
2001-2010 APC = -3.300
Philippines
years

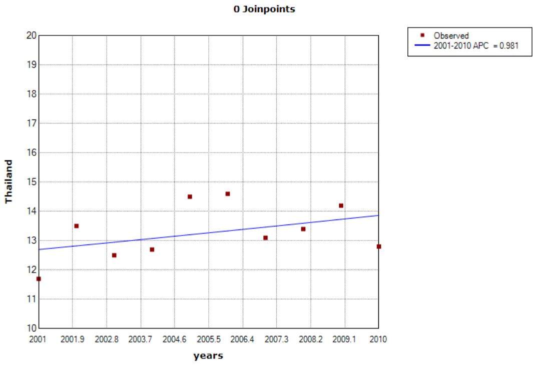
0 Joinpoints
Observed
2001-2010 APC = 0.981
Thailand
years

Oceania

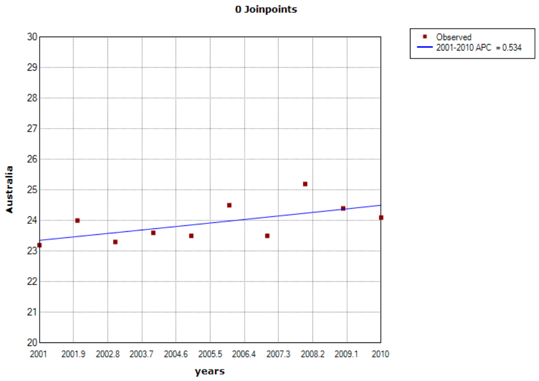
0 Joinpoints
Observed
2001-2010 APC = 0.534
Australia
years

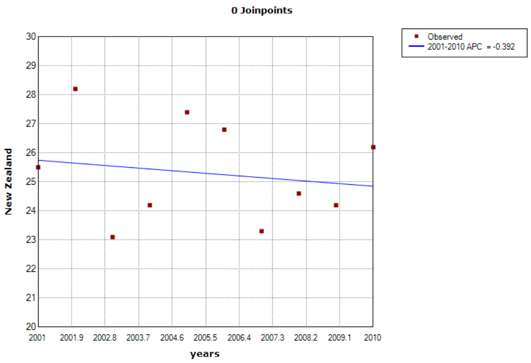
0 Joinpoints
Observed
2001-2010 APC = -0.392
New Zealand
years

Northern America

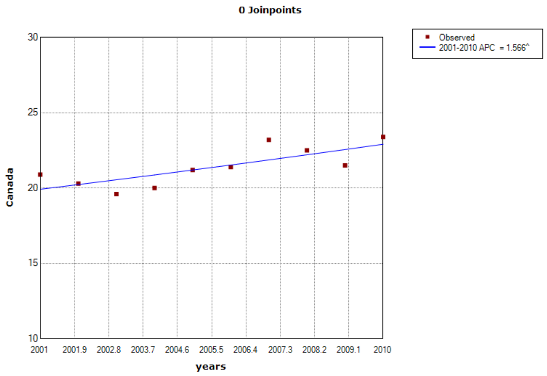

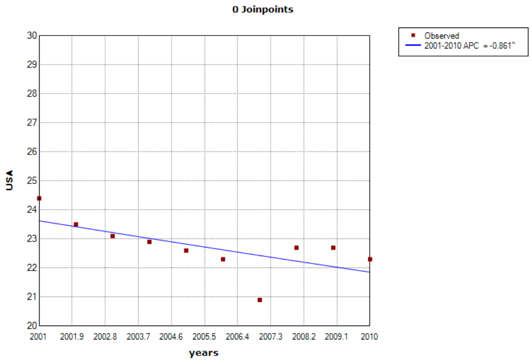

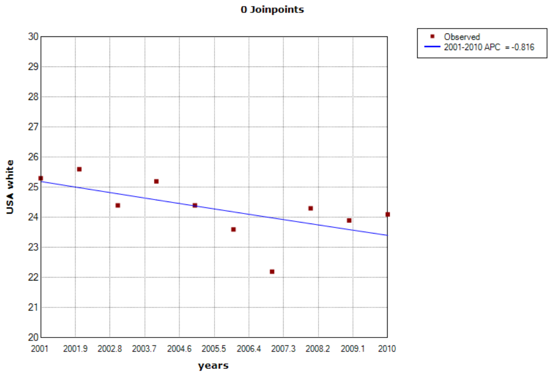

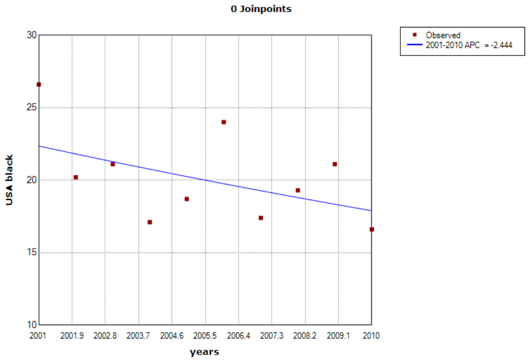

Southern America

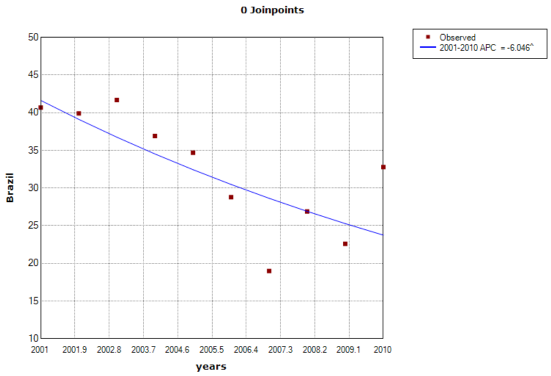

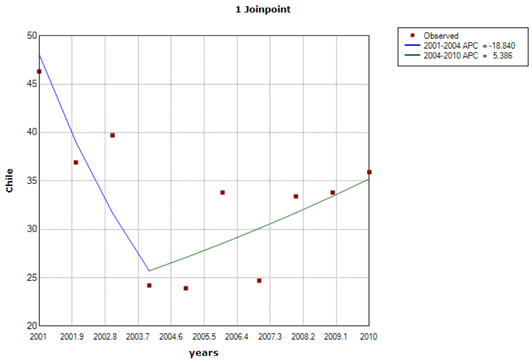

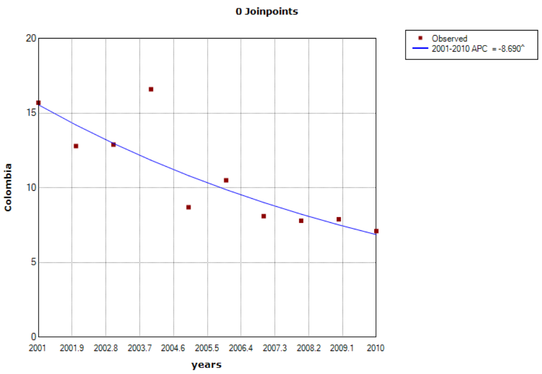

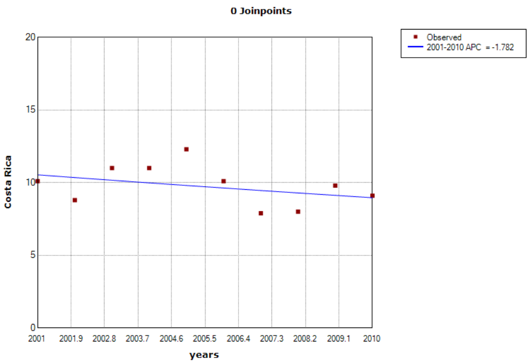

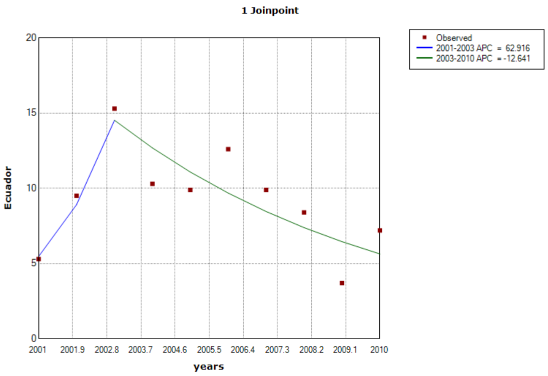

Northern Europe

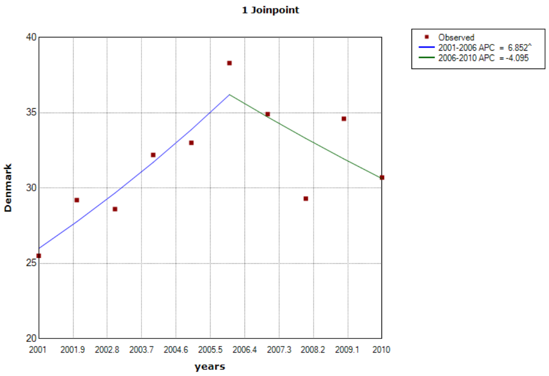

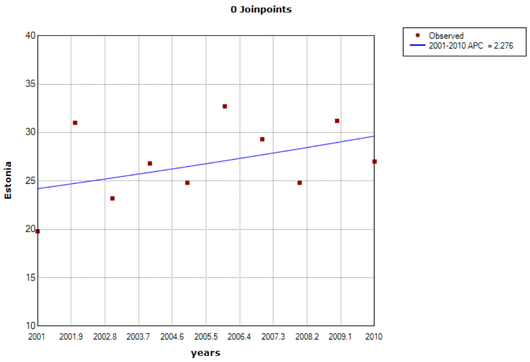

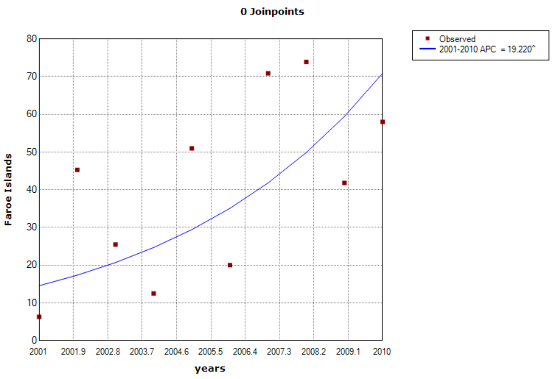
0 Joinpoints
Observed
2001-2010 APC = 19.220^
Faroe Islands
years
2001 2001.9 2002.8 2003.7 2004.6 2005.5 2006.4 2007.3 2008.2 2009.1 2010

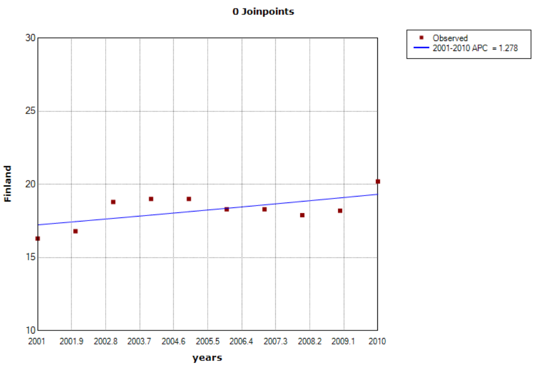
0 Joinpoints
Observed
2001-2010 APC = 1.278
Finland
years
2001 2001.9 2002.8 2003.7 2004.6 2005.5 2006.4 2007.3 2008.2 2009.1 2010

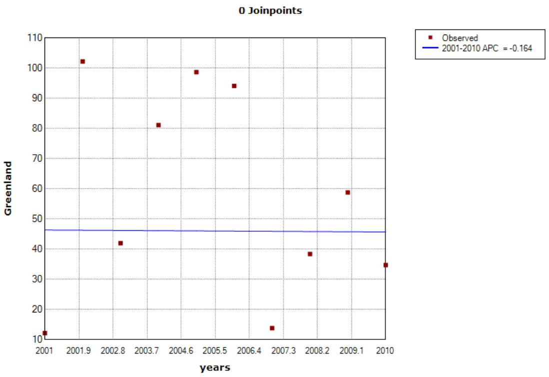
0 Joinpoints
Observed
2001-2010 APC = -0.164
Greenland
years
2001 2001.9 2002.8 2003.7 2004.6 2005.5 2006.4 2007.3 2008.2 2009.1 2010

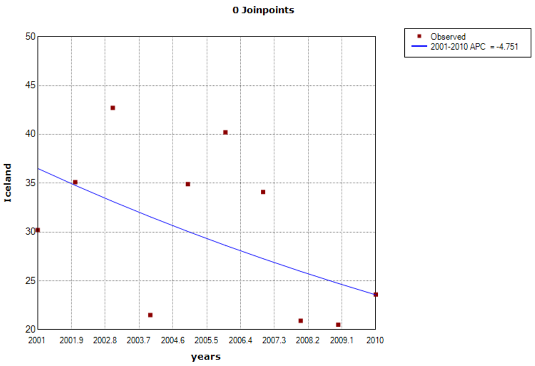
0 Joinpoints
Observed
2001-2010 APC = -4.751
Iceland
years
2001 2001.9 2002.8 2003.7 2004.6 2005.5 2006.4 2007.3 2008.2 2009.1 2010

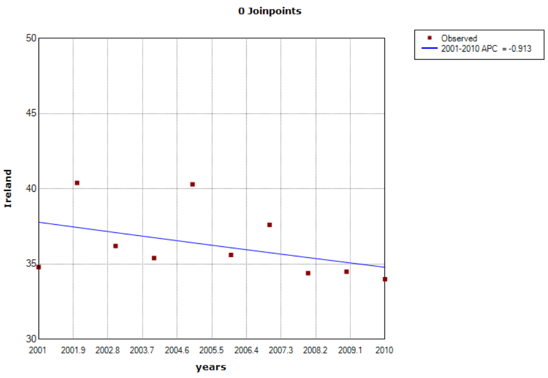
0 Joinpoints
Observed
2001-2010 APC = -0.913
Ireland
years
2001 2001.9 2002.8 2003.7 2004.6 2005.5 2006.4 2007.3 2008.2 2009.1 2010

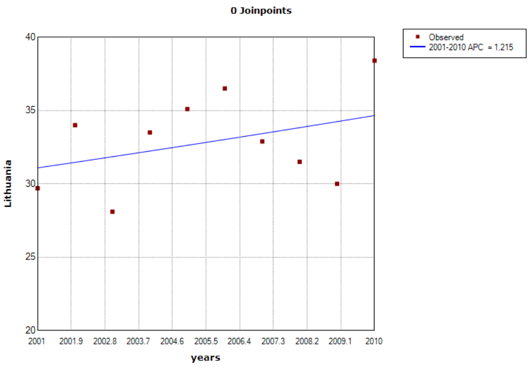
0 Joinpoints
Observed
2001-2010 APC = 1.215
Lithuania
years
2001 2001.9 2002.8 2003.7 2004.6 2005.5 2006.4 2007.3 2008.2 2009.1 2010

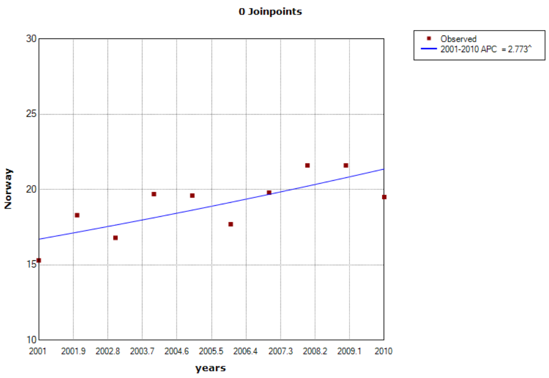

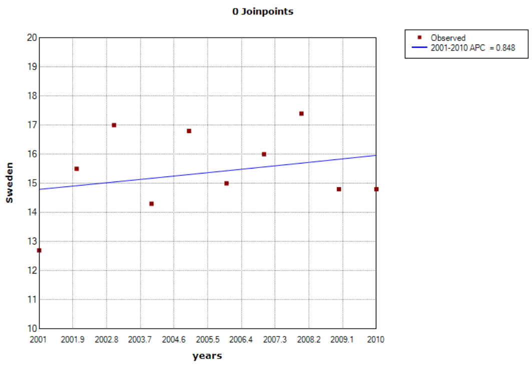

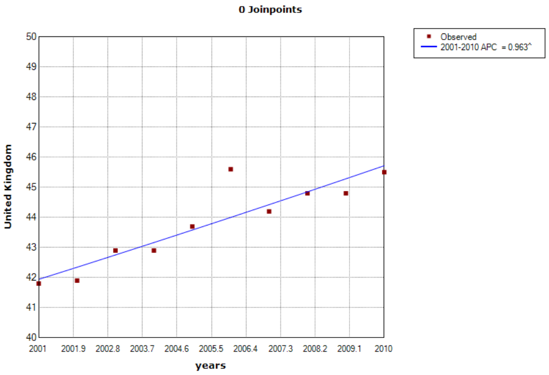

Western Europe

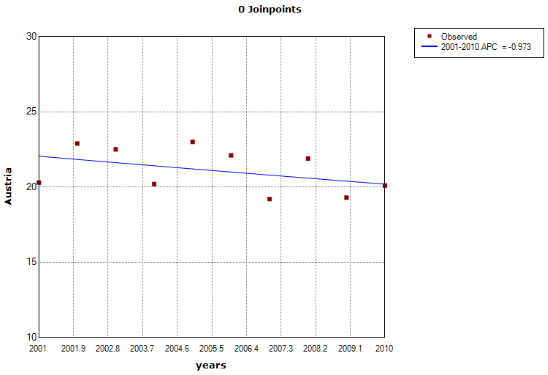

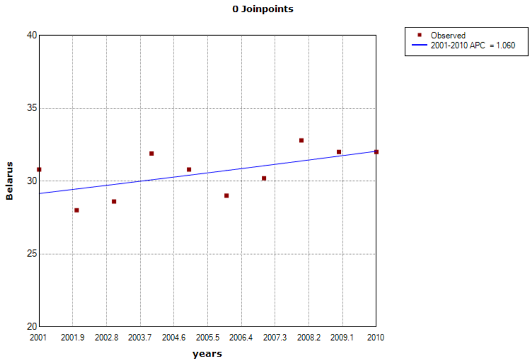

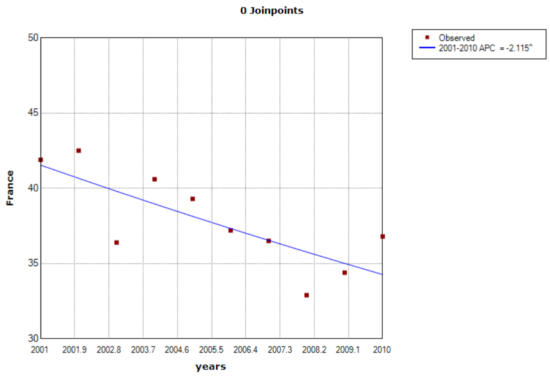

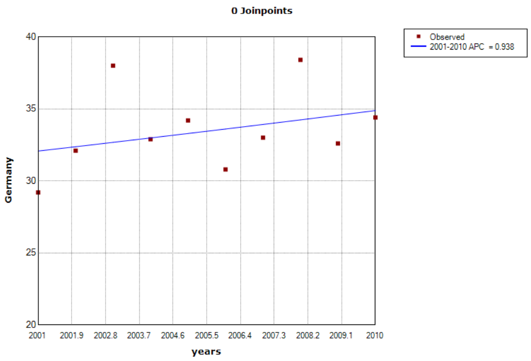

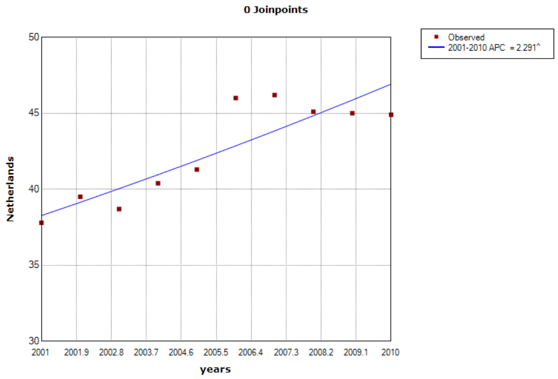

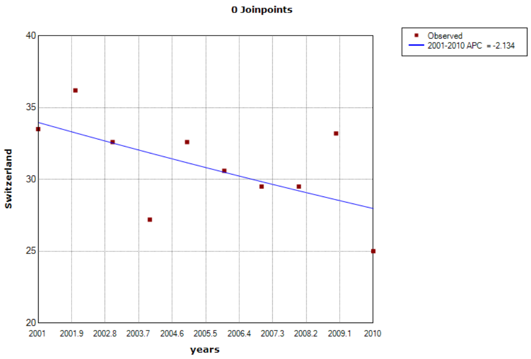

Southern Europe

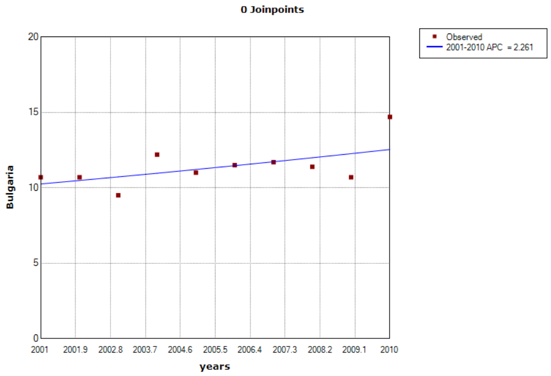

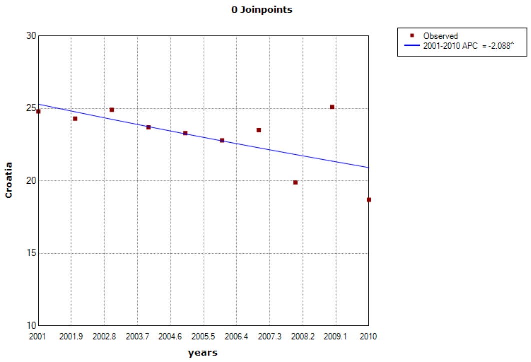

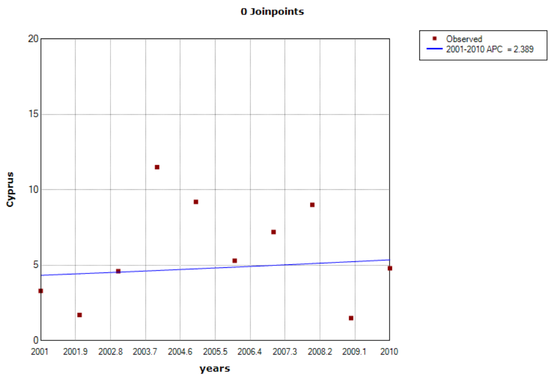
0 Joinpoints
Observed
2001-2010 APC = 2.389
Cyprus
years
2001 2001.9 2002.8 2003.7 2004.6 2005.5 2006.4 2007.3 2008.2 2009.1 2010
0 5 10 15 20

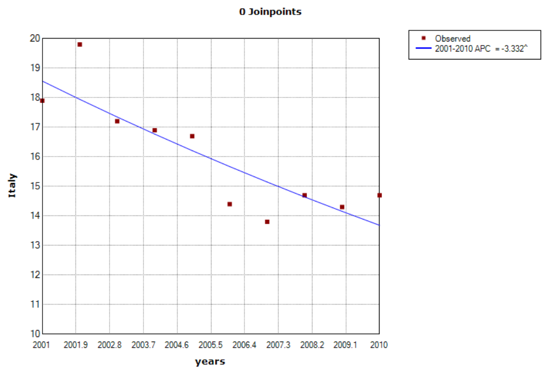
0 Joinpoints
Observed
2001-2010 APC = -3.332^
Italy
years
2001 2001.9 2002.8 2003.7 2004.6 2005.5 2006.4 2007.3 2008.2 2009.1 2010
10 11 12 13 14 15 16 17 18 19 20

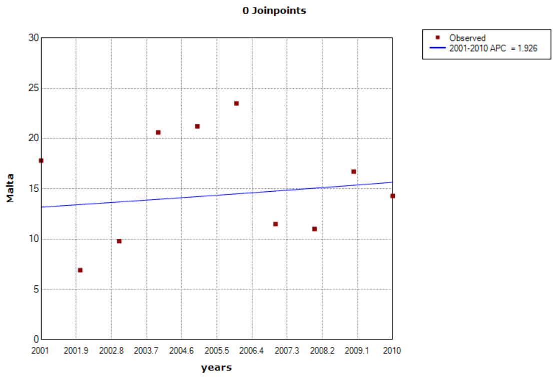
0 Joinpoints
Observed
2001-2010 APC = 1.926
Malta
years
2001 2001.9 2002.8 2003.7 2004.6 2005.5 2006.4 2007.3 2008.2 2009.1 2010
0 5 10 15 20 25 30

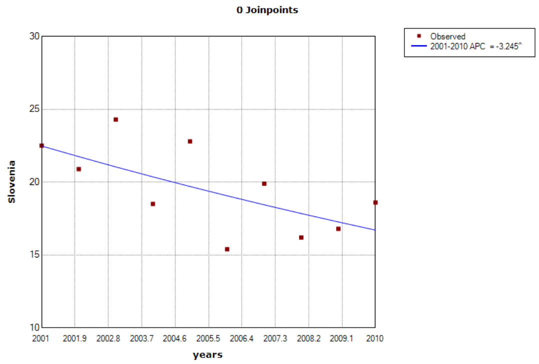
0 Joinpoints
Observed
2001-2010 APC = -3.245^
Slovenia
years
2001 2001.9 2002.8 2003.7 2004.6 2005.5 2006.4 2007.3 2008.2 2009.1 2010
10 15 20 25 30

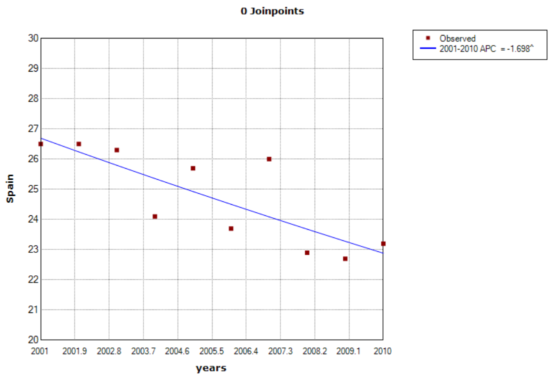
0 Joinpoints
Observed
2001-2010 APC = -1.698^
Spain
years
2001 2001.9 2002.8 2003.7 2004.6 2005.5 2006.4 2007.3 2008.2 2009.1 2010
20 21 22 23 24 25 26 27 28 29 30

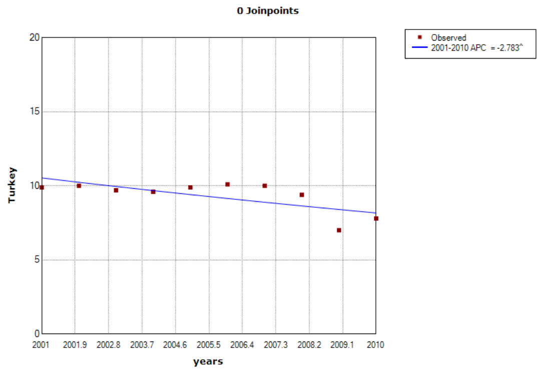
0 Joinpoints
Observed
2001-2010 APC = -2.783^
Turkey
years
2001 2001.9 2002.8 2003.7 2004.6 2005.5 2006.4 2007.3 2008.2 2009.1 2010
0 5 10 15 20

Eastern Europe

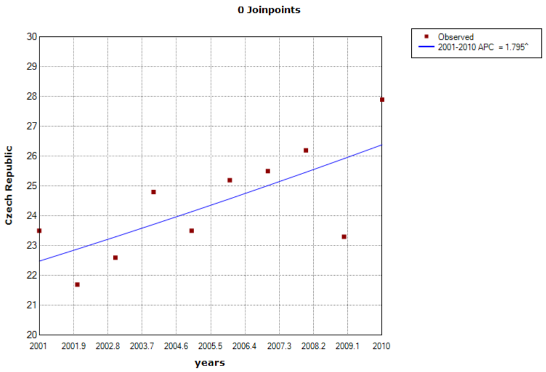

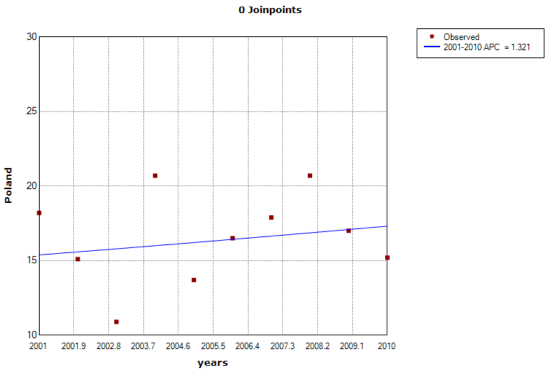

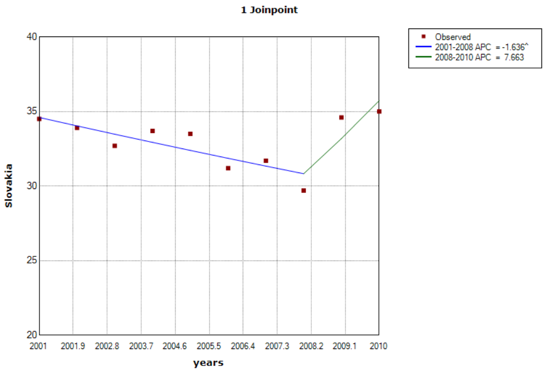

Africa

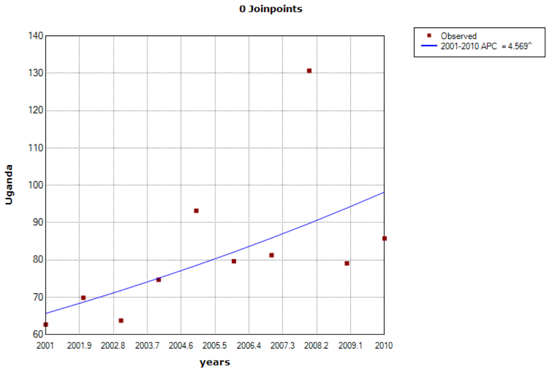

h.) Incidence female aged above 50 years old

Asia

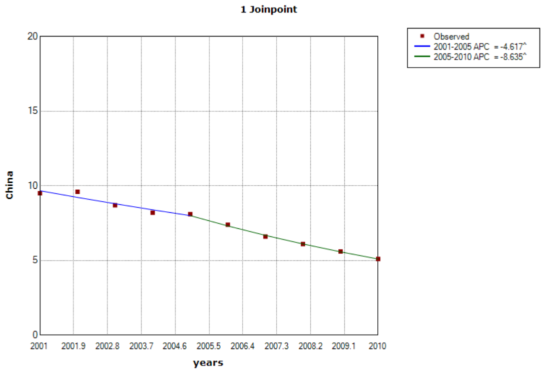

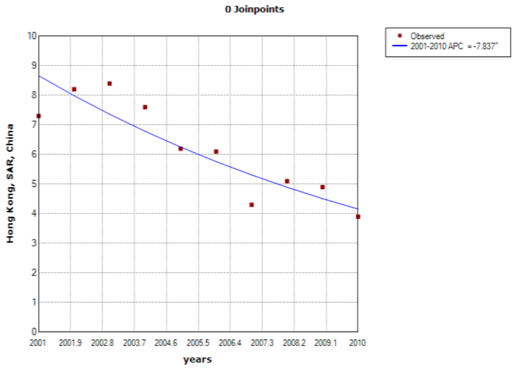

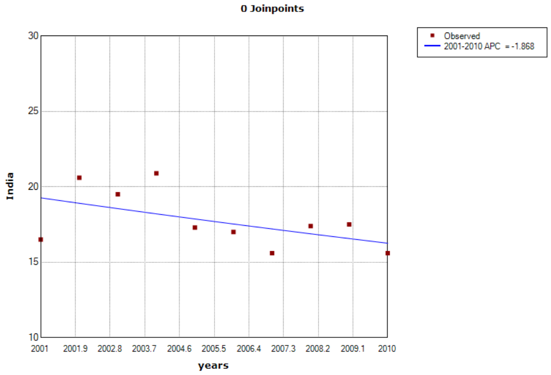

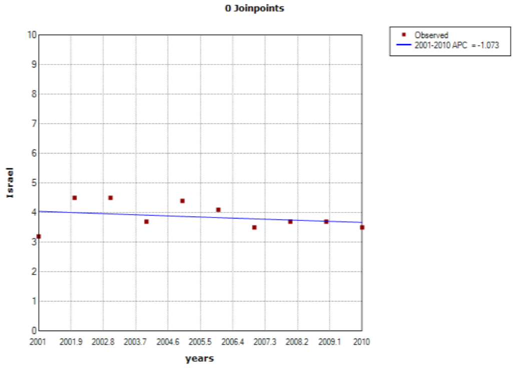

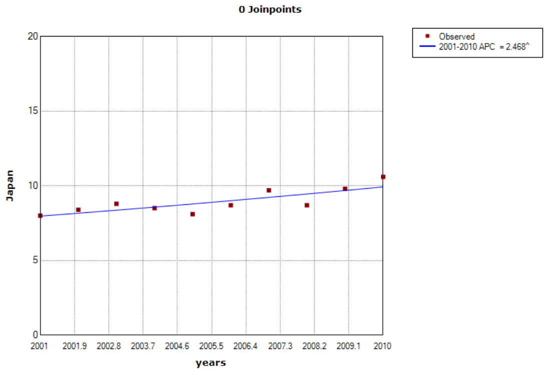

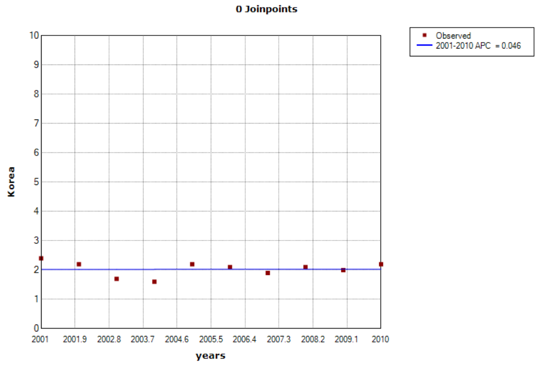

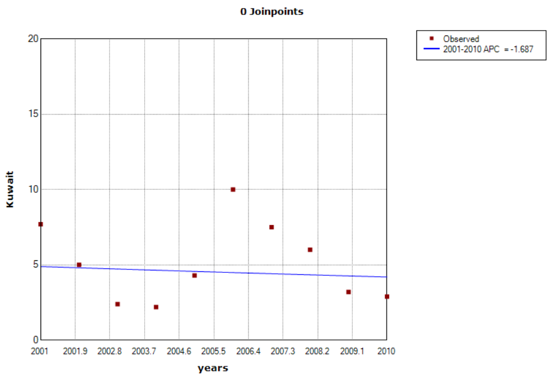
0 Joinpoints
Kuwait
years
Observed
2001-2010 APC = -1.687
0
5
10
15
20
2001
2001.9
2002.8
2003.7
2004.6
2005.5
2006.4
2007.3
2008.2
2009.1
2010

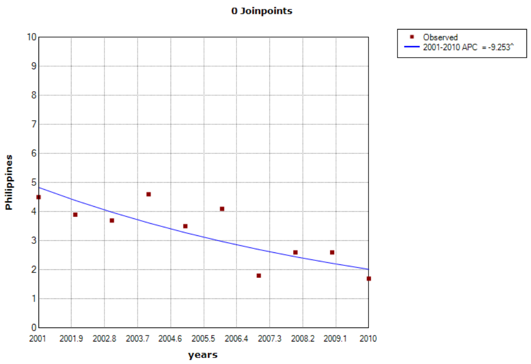
0 Joinpoints
Philippines
years
Observed
2001-2010 APC = -9.253^
0
1
2
3
4
5
6
7
8
9
10
2001
2001.9
2002.8
2003.7
2004.6
2005.5
2006.4
2007.3
2008.2
2009.1
2010

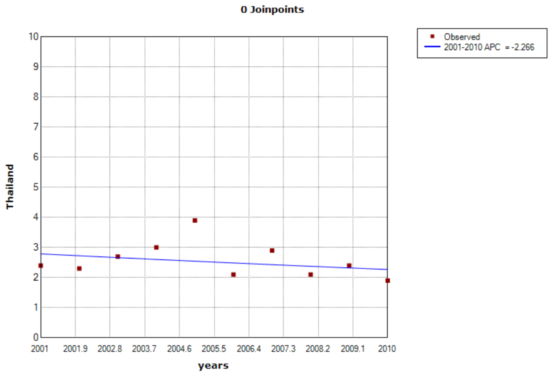
0 Joinpoints
Thailand
years
Observed
2001-2010 APC = -2.266
0
1
2
3
4
5
6
7
8
9
10
2001
2001.9
2002.8
2003.7
2004.6
2005.5
2006.4
2007.3
2008.2
2009.1
2010

Oceania

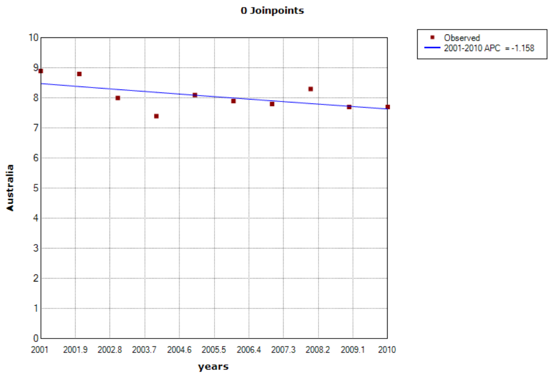
0 Joinpoints
Australia
years
Observed
2001-2010 APC = -1.158
0
1
2
3
4
5
6
7
8
9
10
2001
2001.9
2002.8
2003.7
2004.6
2005.5
2006.4
2007.3
2008.2
2009.1
2010

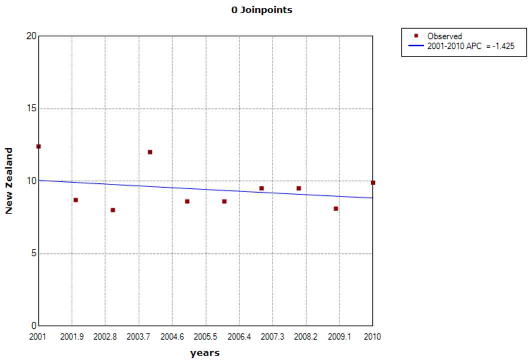
0 Joinpoints
New Zealand
years
Observed
2001-2010 APC = -1.425
0
5
10
15
20
2001
2001.9
2002.8
2003.7
2004.6
2005.5
2006.4
2007.3
2008.2
2009.1
2010

Northern America

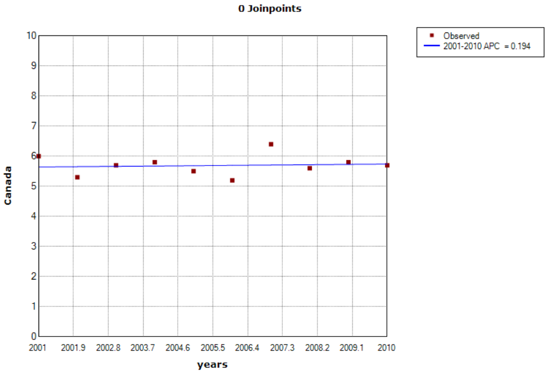

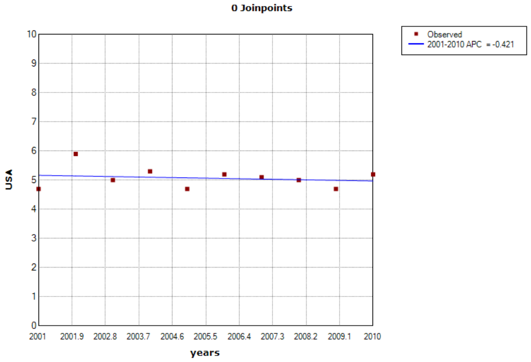

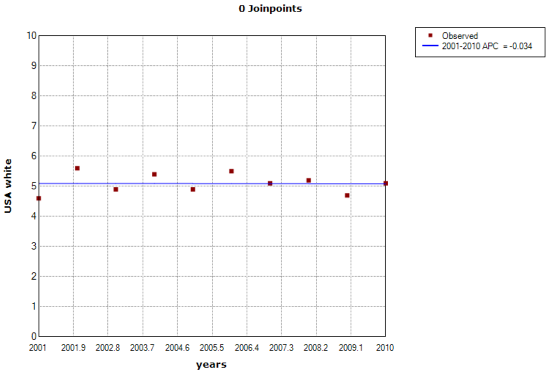

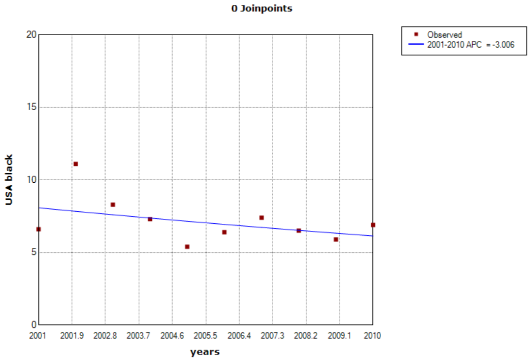

Southern America

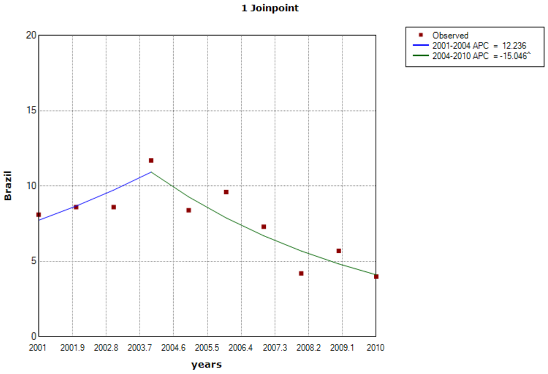

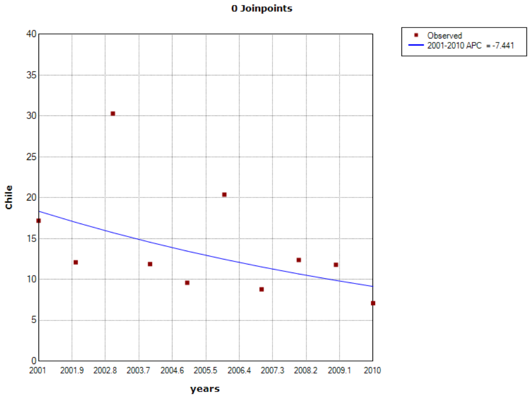

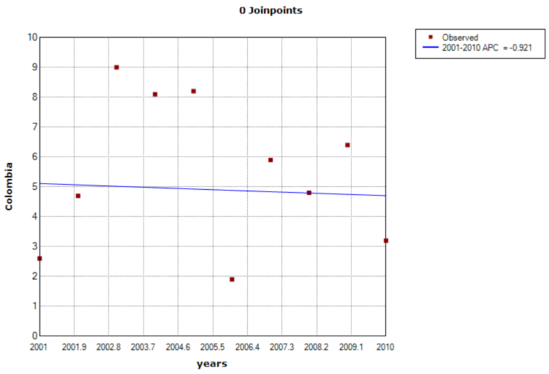

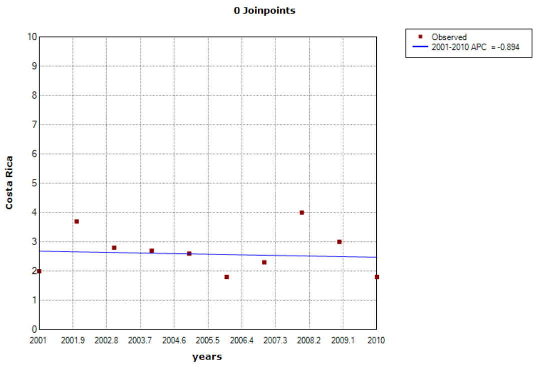

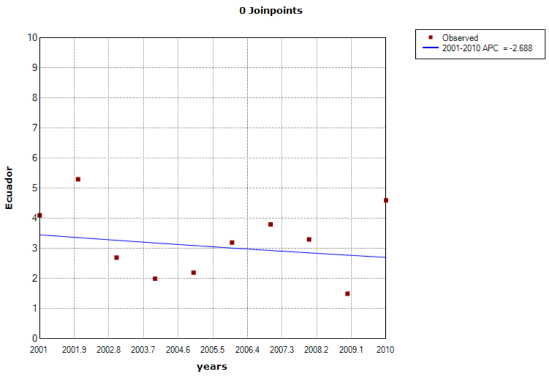

Northern Europe

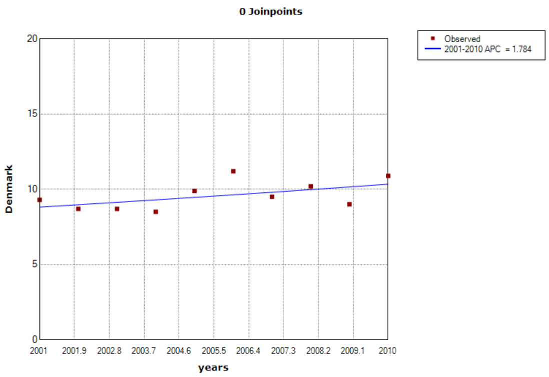

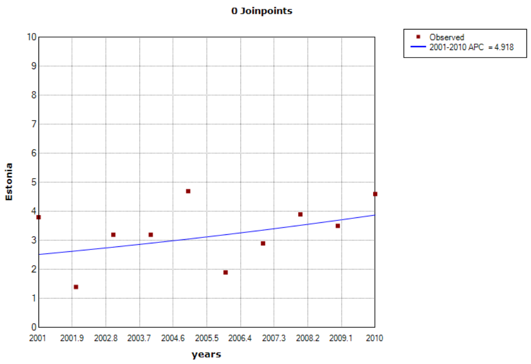

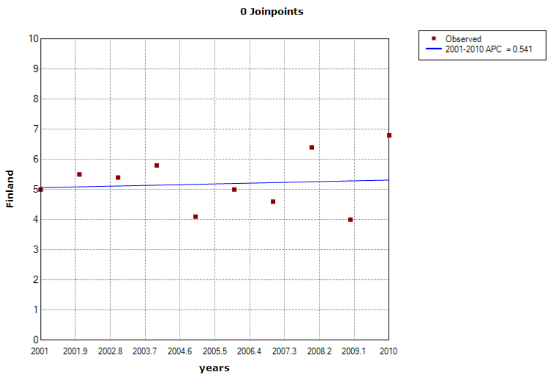
0 Joinpoints
Observed
2001-2010 APC = 0.541
Finland
years

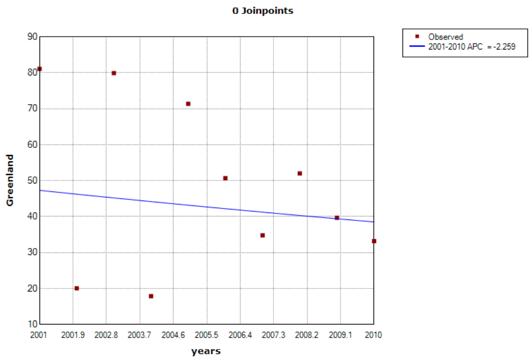
0 Joinpoints
Observed
2001-2010 APC = -2.259
Greenland
years

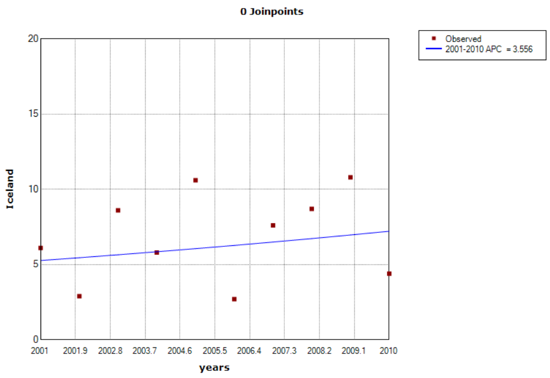
0 Joinpoints
Observed
2001-2010 APC = 3.556
Iceland
years

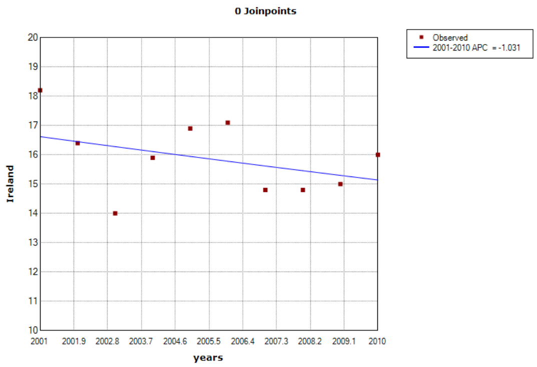
0 Joinpoints
Observed
2001-2010 APC = -1.031
Ireland
years

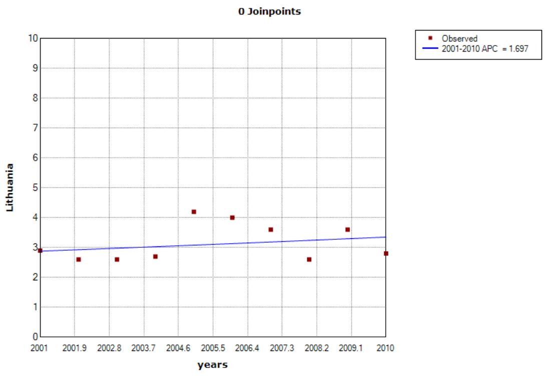
0 Joinpoints
Observed
2001-2010 APC = 1.697
Lithuania
years

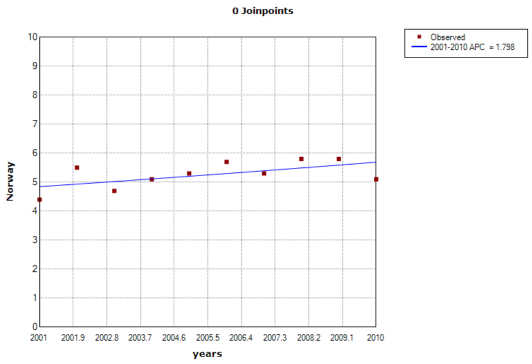
0 Joinpoints
Observed
2001-2010 APC = 1.798
Norway
years

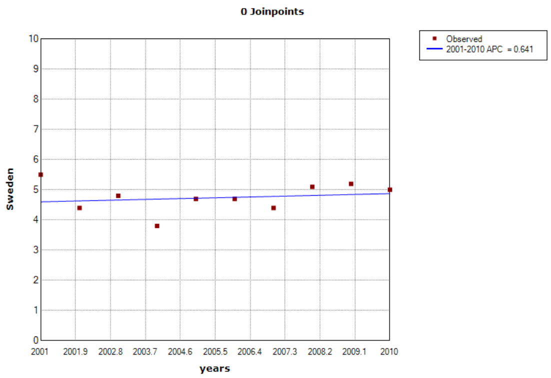

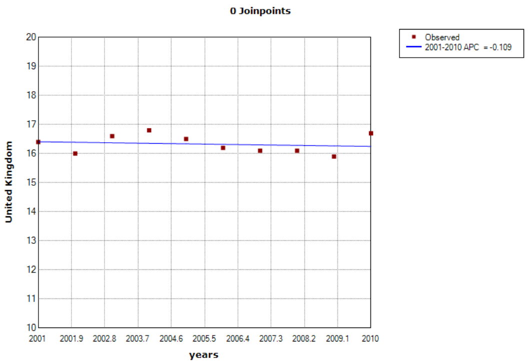

Western Europe

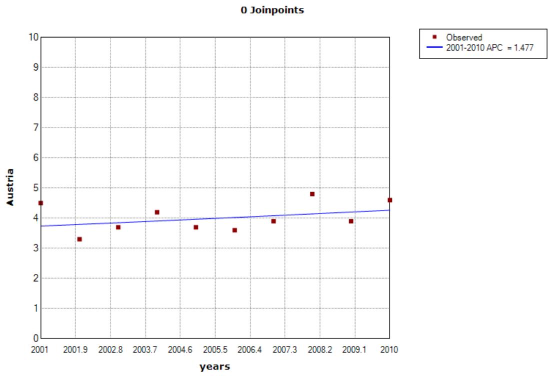

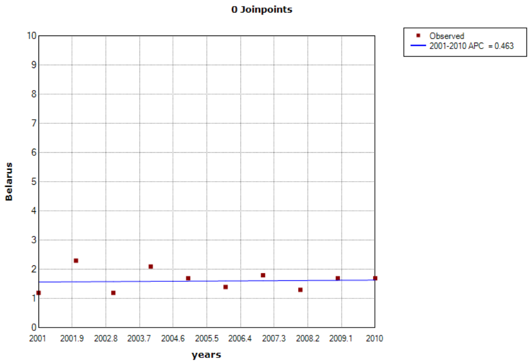

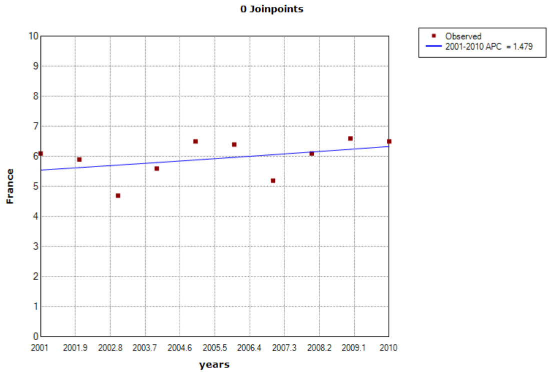

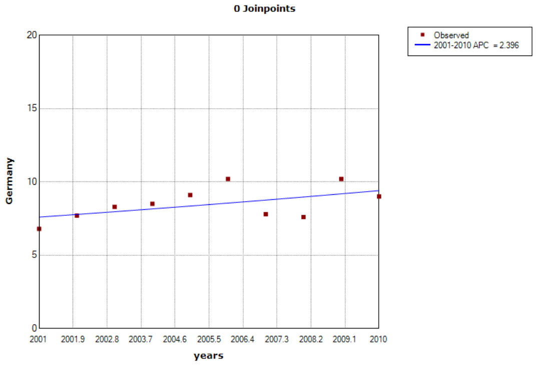

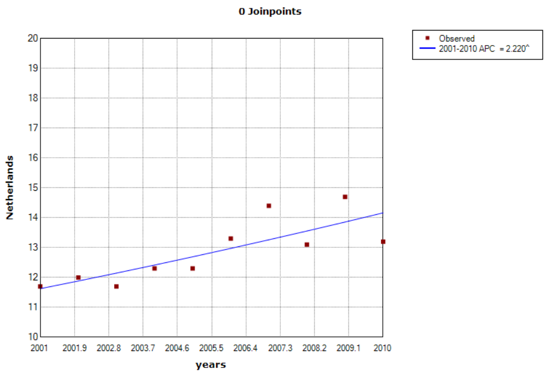

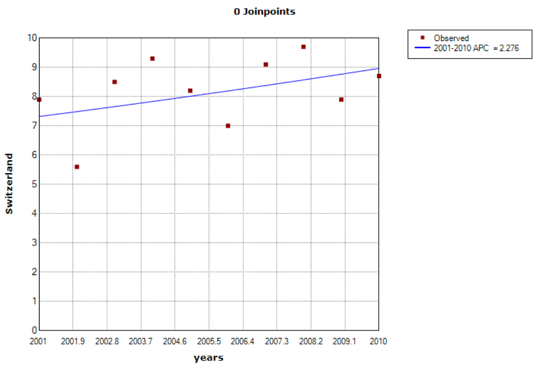

Southern Europe

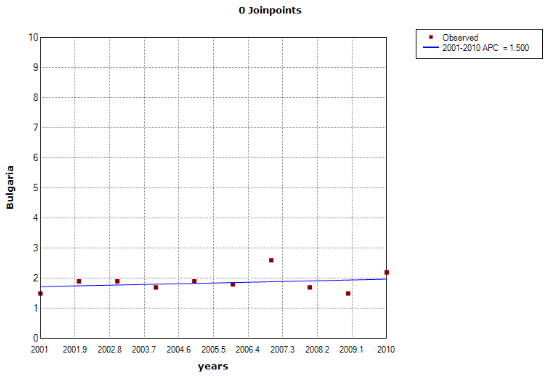

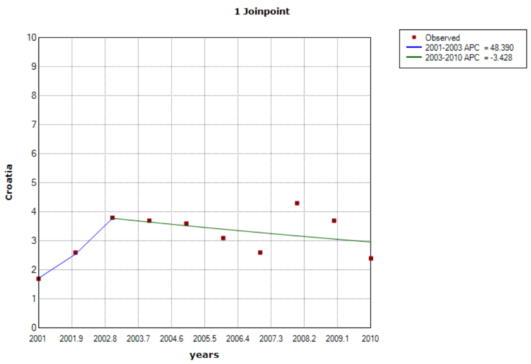

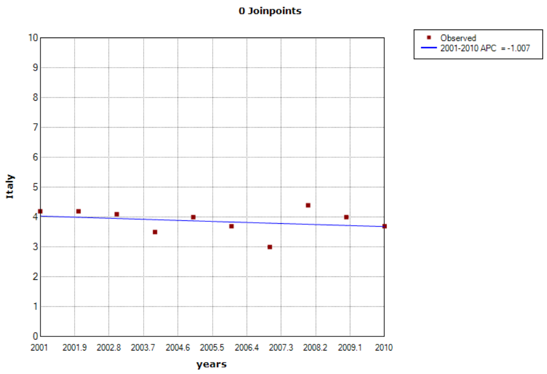

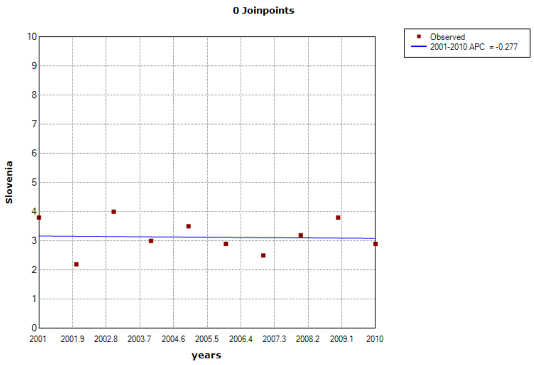

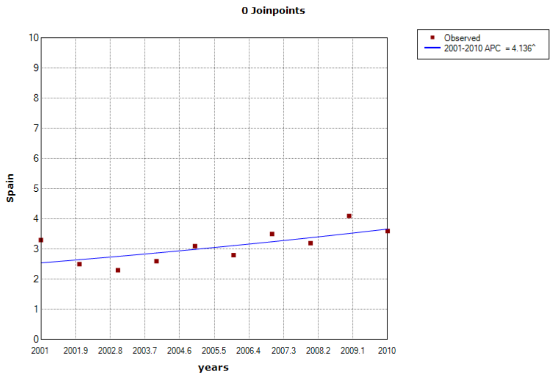

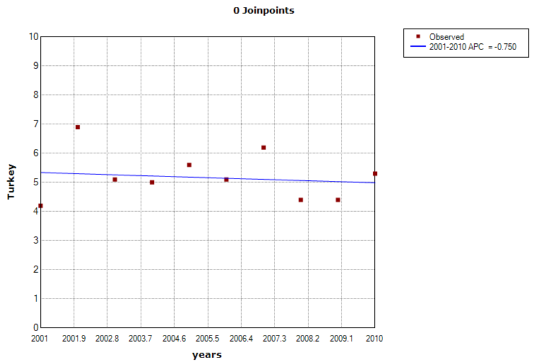

Eastern Europe

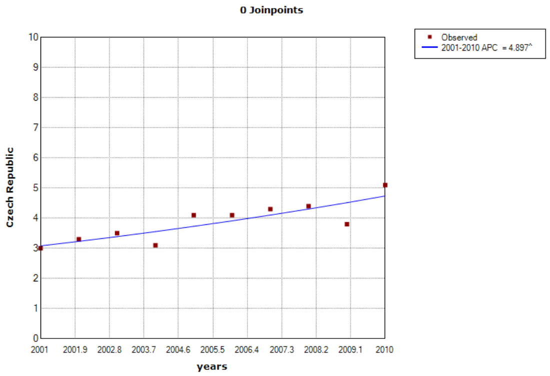

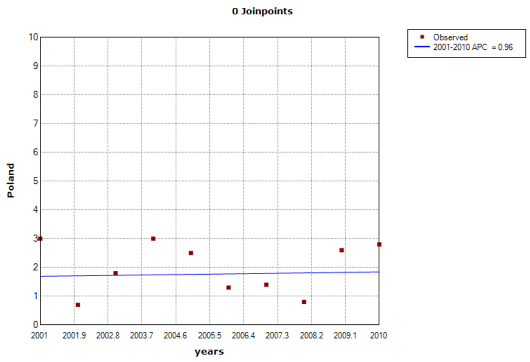

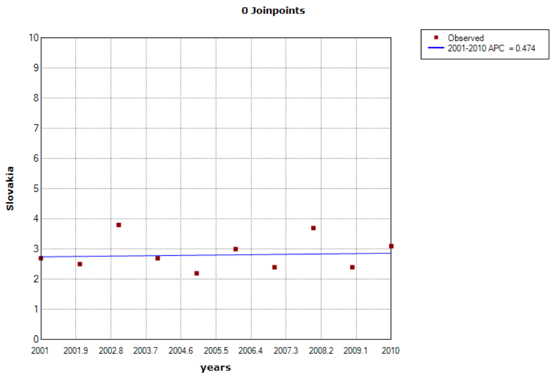

Africa

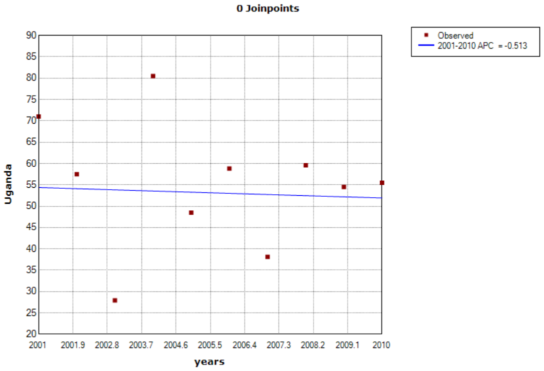
0 Joinpoints
Observed
2001-2010 APC = -0.513
Uganda
years
90
85
80
75
70
65
60
55
50
45
40
35
30
25
20
2001
2001.9
2002.8
2003.7
2004.6
2005.5
2006.4
2007.3
2008.2
2009.1
2010

i.) mortality male aged from 0 to 85 years old

Asia

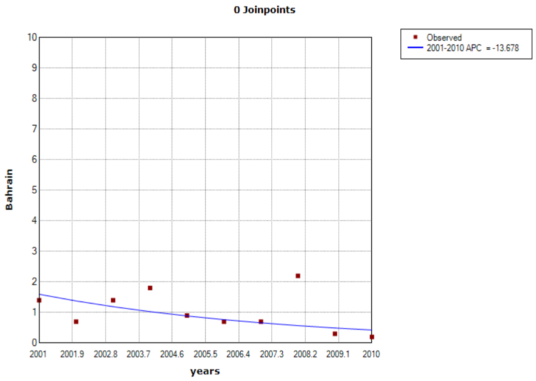

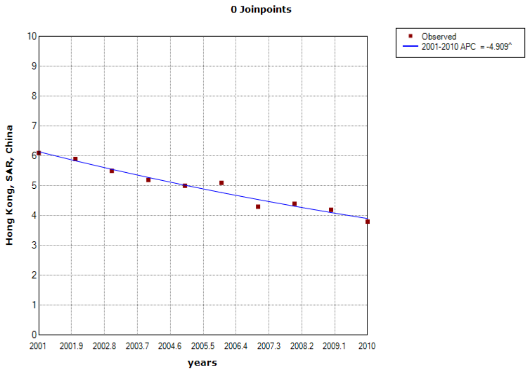

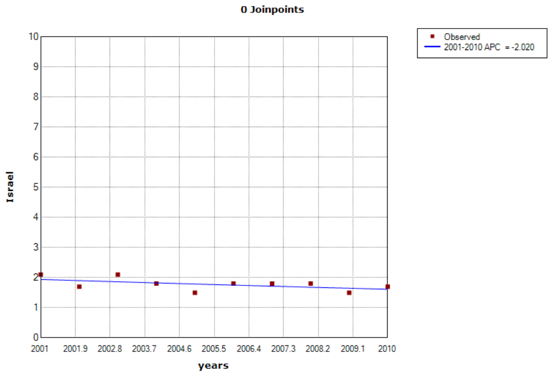

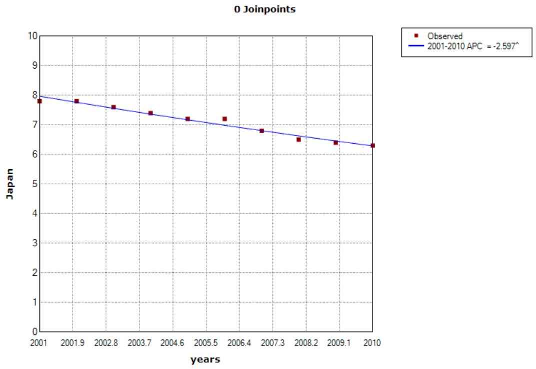

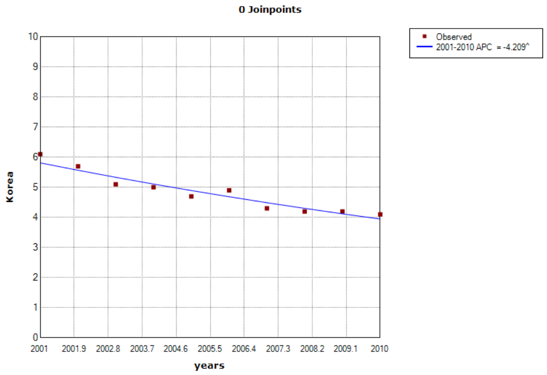

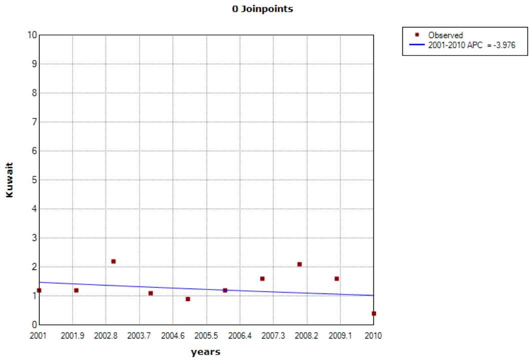

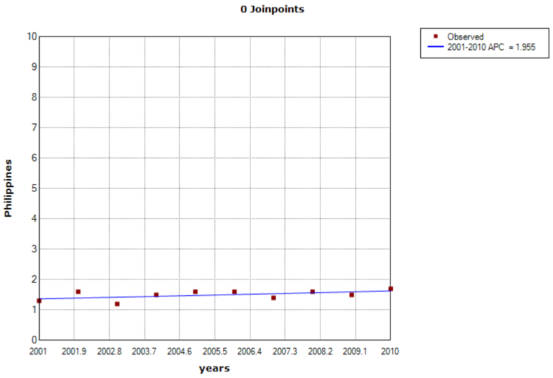
0 Joinpoints
Observed
2001-2010 APC = 1.955
Philippines
years

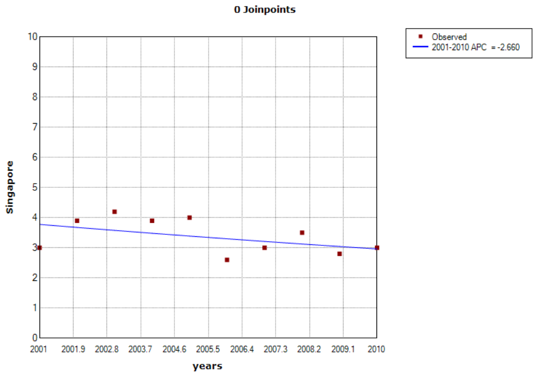
0 Joinpoints
Observed
2001-2010 APC = -2.660
Singapore
years

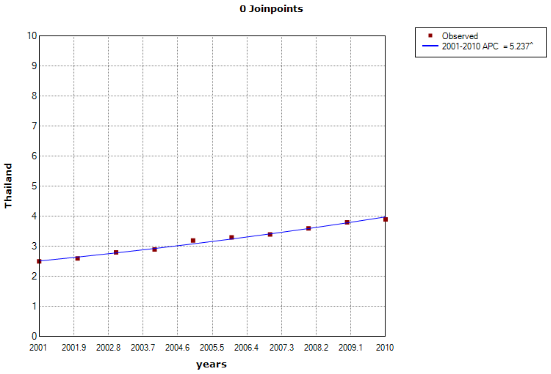
0 Joinpoints
Observed
2001-2010 APC = 5.237^
Thailand
years

Oceania

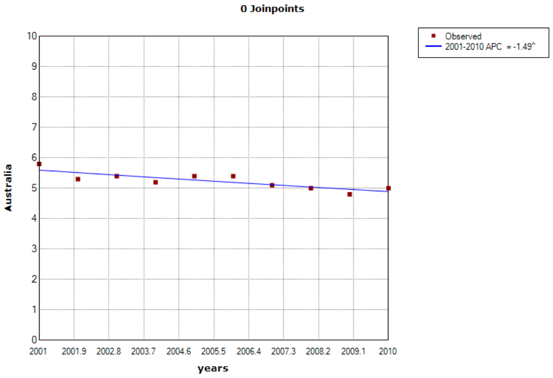
0 Joinpoints
Observed
2001-2010 APC = -1.49^
Australia
years

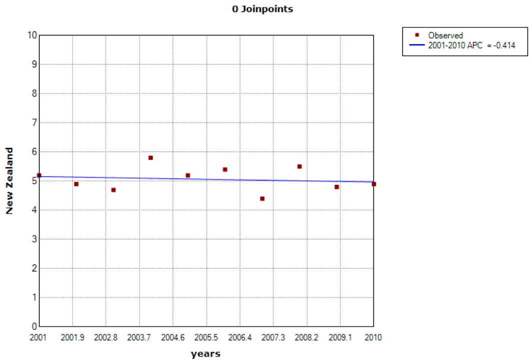
0 Joinpoints
Observed
2001-2010 APC = -0.414
New Zealand
years

Northern America

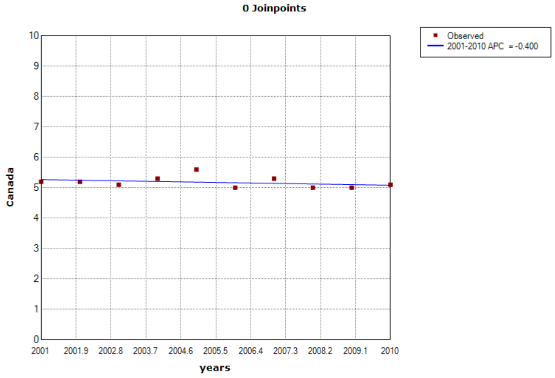

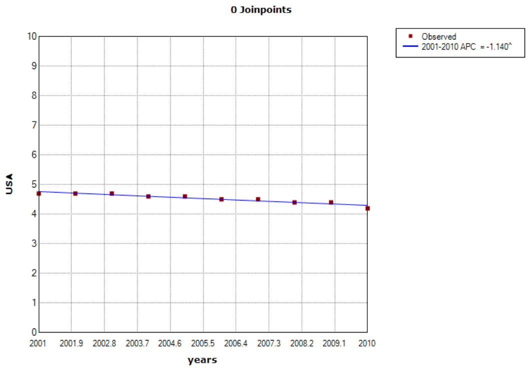

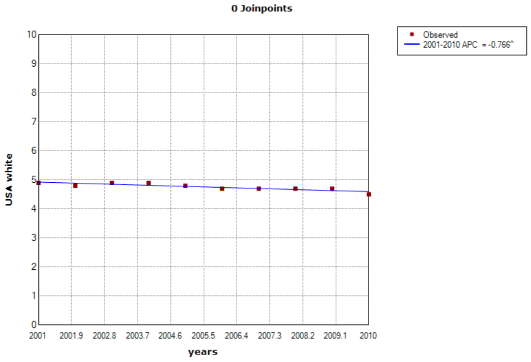

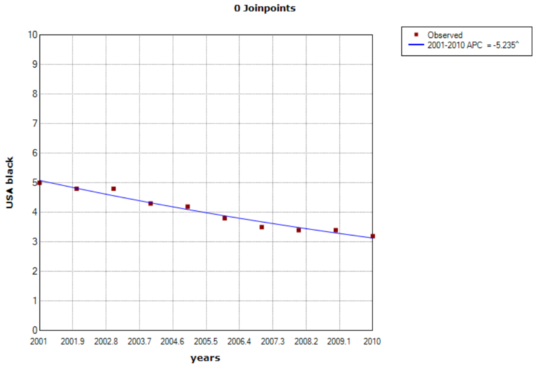

Southern America

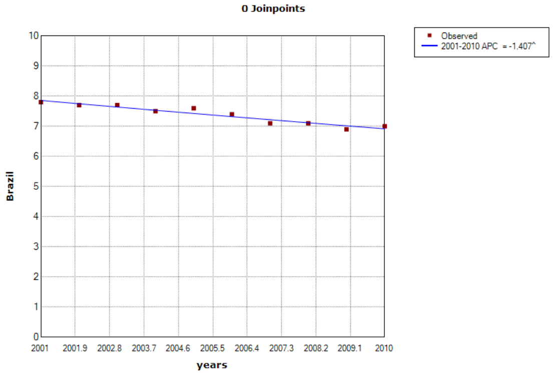

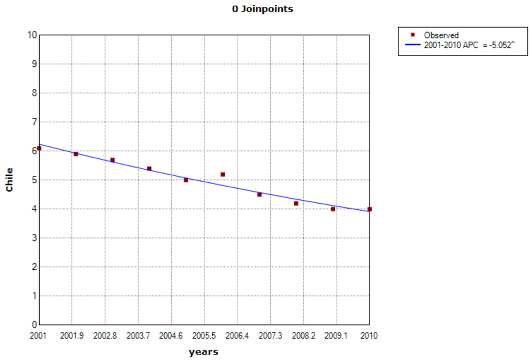

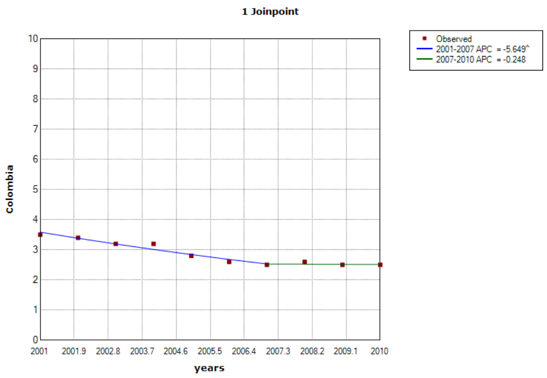

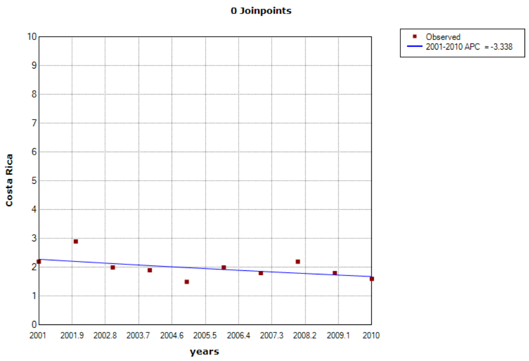

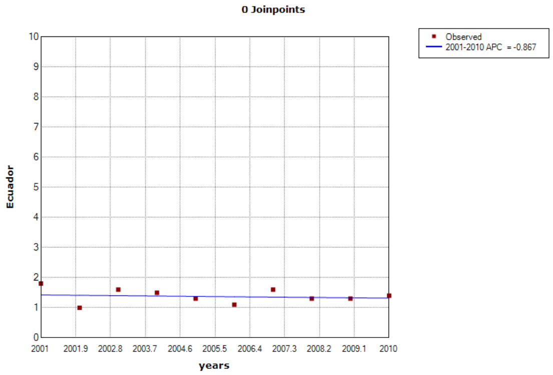

Northern Europe

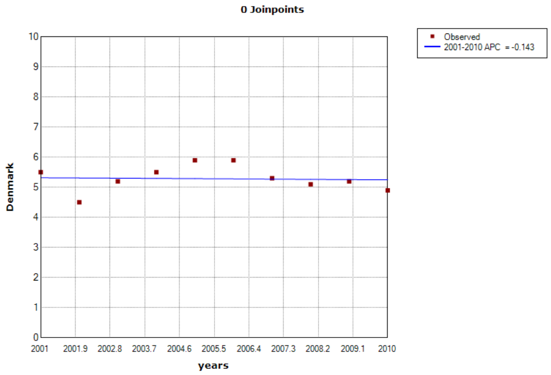

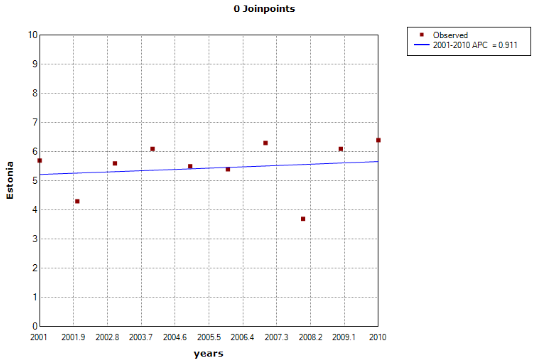

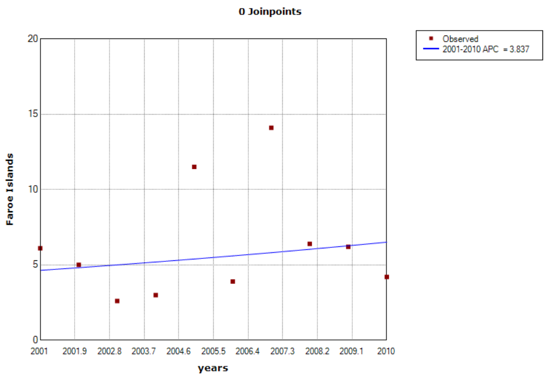
0 Joinpoints
Observed
2001-2010 APC = 3.837
Faroe Islands
years

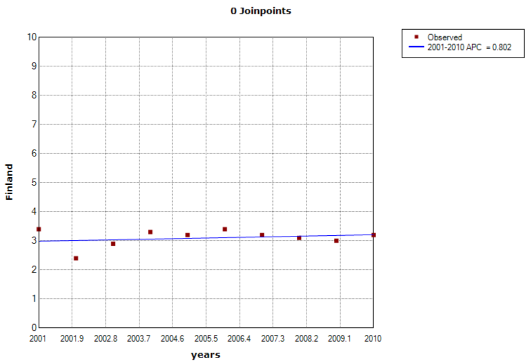
0 Joinpoints
Observed
2001-2010 APC = 0.802
Finland
years

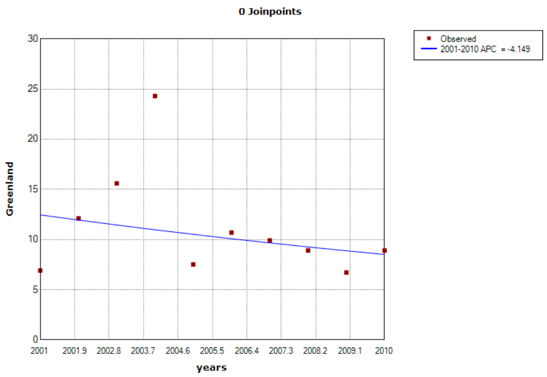
0 Joinpoints
Observed
2001-2010 APC = -4.149
Greenland
years

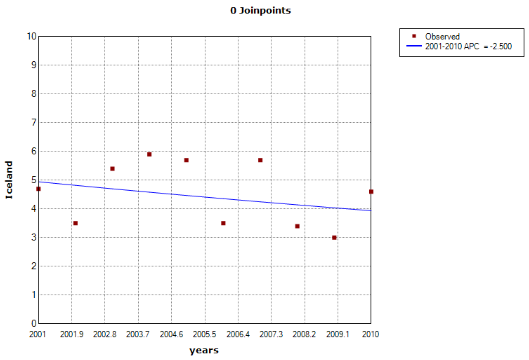
0 Joinpoints
Observed
2001-2010 APC = -2.500
Iceland
years

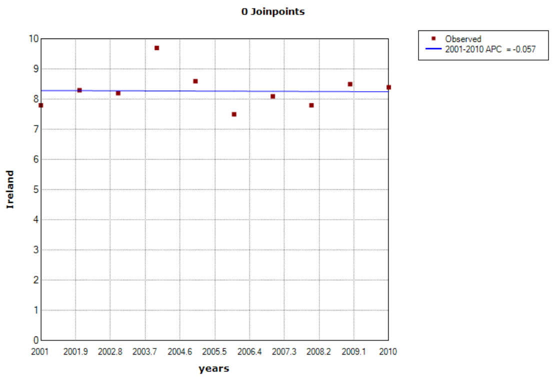
0 Joinpoints
Observed
2001-2010 APC = -0.057
Ireland
years

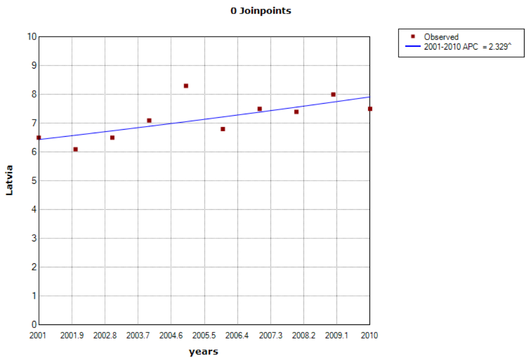
0 Joinpoints
Observed
2001-2010 APC = 2.329^
Latvia
years

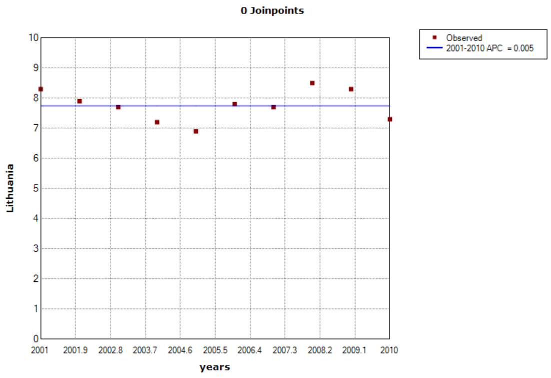

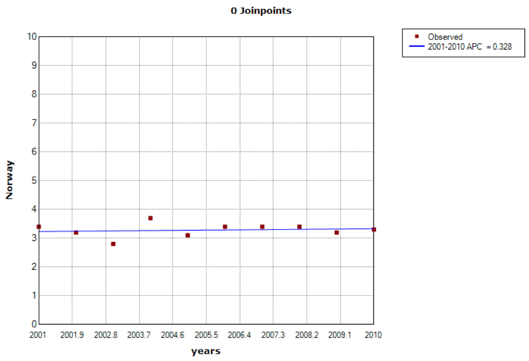

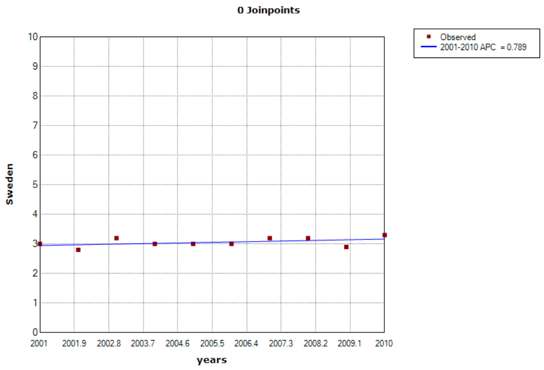

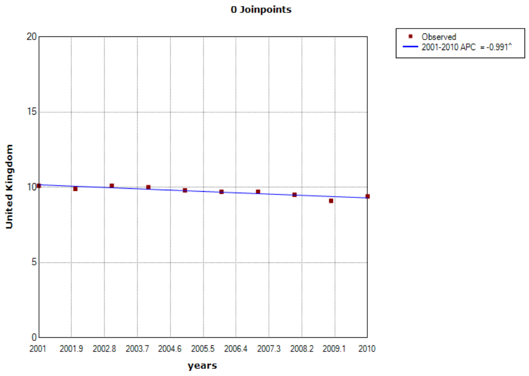

Western Europe

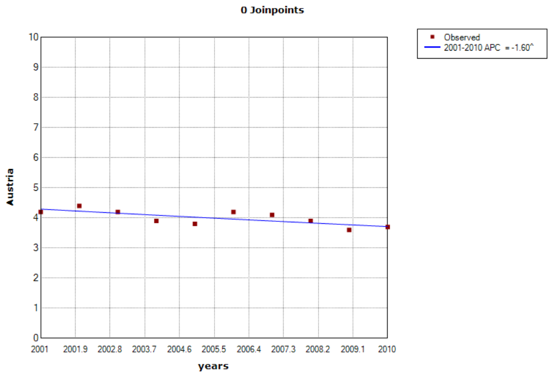

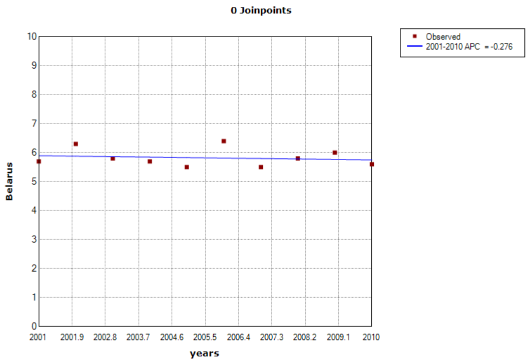

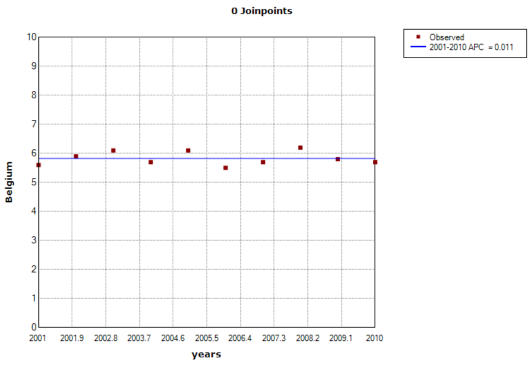

0 Joinpoints
Observed
2001-2010 APC = 0.011
Belgium
years
2001
2001.9
2002.8
2003.7
2004.6
2005.5
2006.4
2007.3
2008.2
2009.1
2010

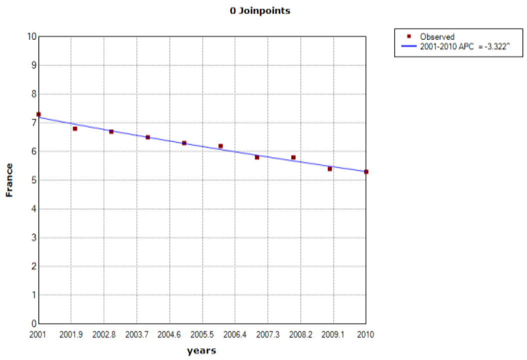

0 Joinpoints
Observed
2001-2010 APC = -3.322^
France
years
2001
2001.9
2002.8
2003.7
2004.6
2005.5
2006.4
2007.3
2008.2
2009.1
2010

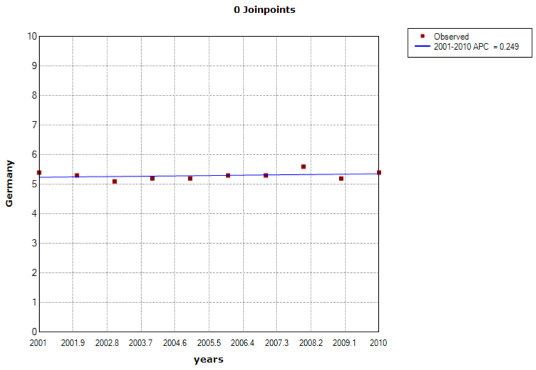

0 Joinpoints
Observed
2001-2010 APC = 0.249
Germany
years
2001
2001.9
2002.8
2003.7
2004.6
2005.5
2006.4
2007.3
2008.2
2009.1
2010

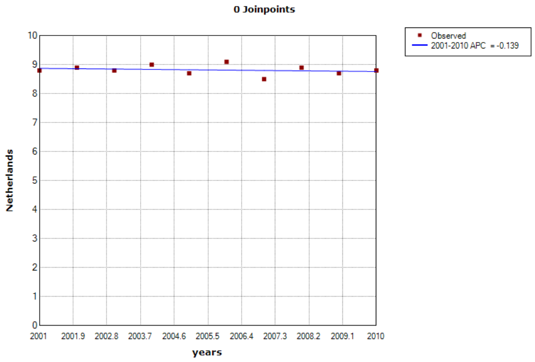

0 Joinpoints
Observed
2001-2010 APC = -0.139
Netherlands
years
2001
2001.9
2002.8
2003.7
2004.6
2005.5
2006.4
2007.3
2008.2
2009.1
2010

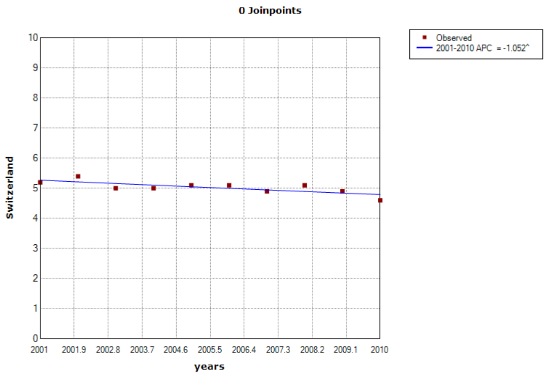

0 Joinpoints
Observed
2001-2010 APC = -1.052^
Switzerland
years
2001
2001.9
2002.8
2003.7
2004.6
2005.5
2006.4
2007.3
2008.2
2009.1
2010

Southern Europe

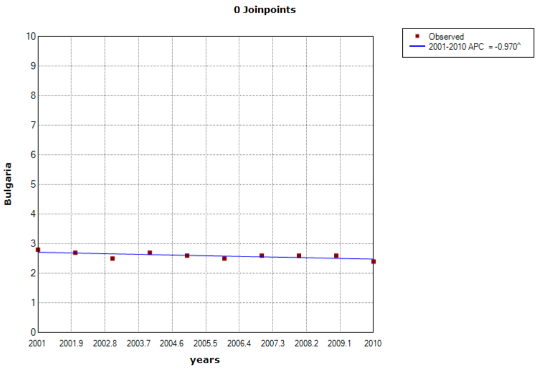

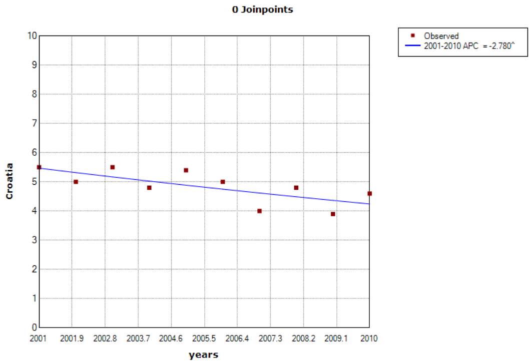

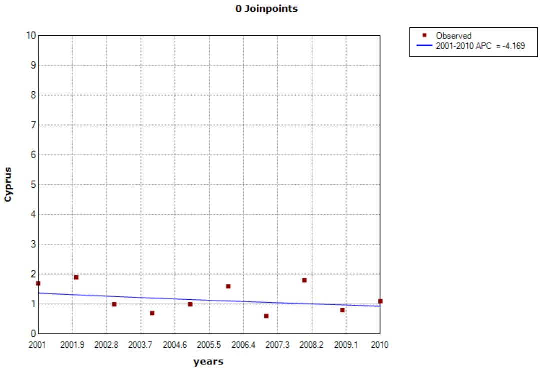

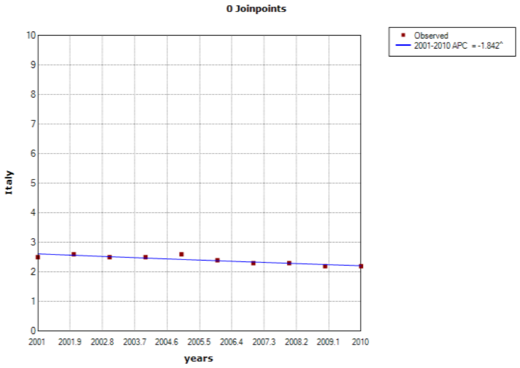

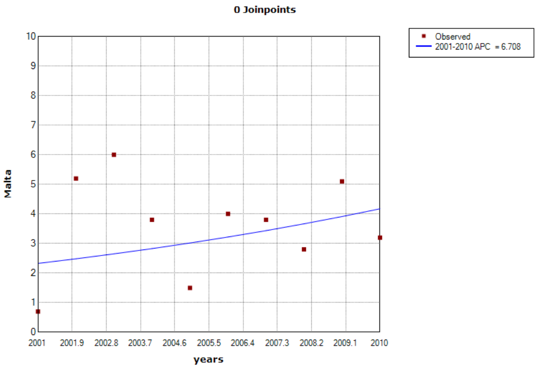

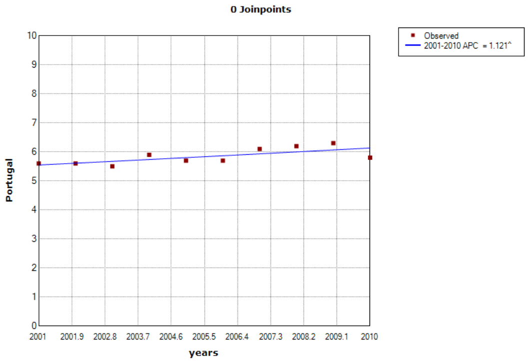

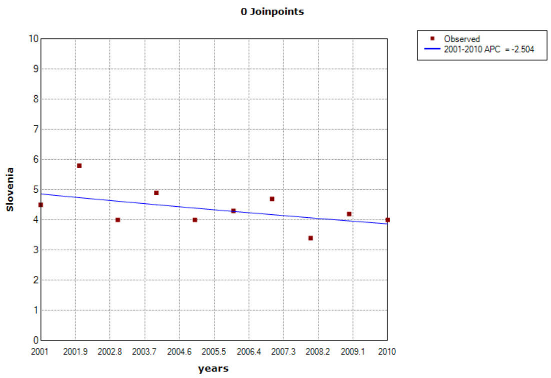

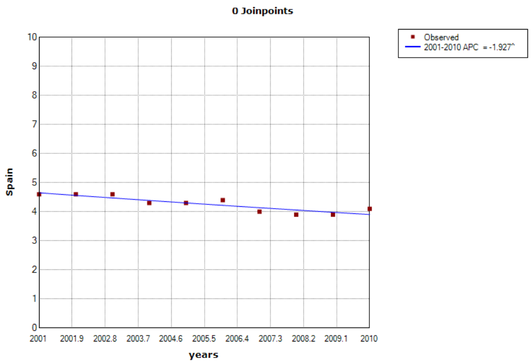

Eastern Europe

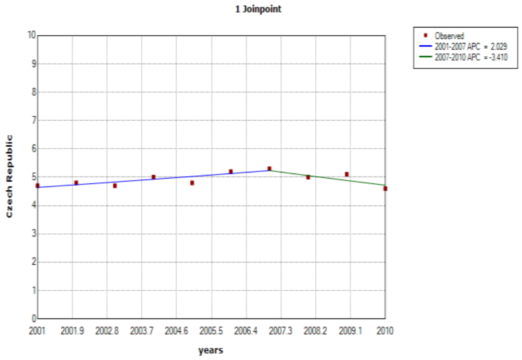

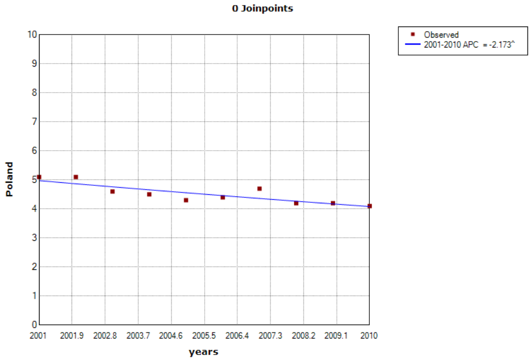

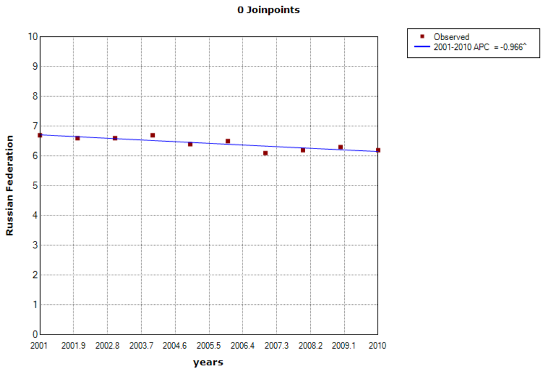

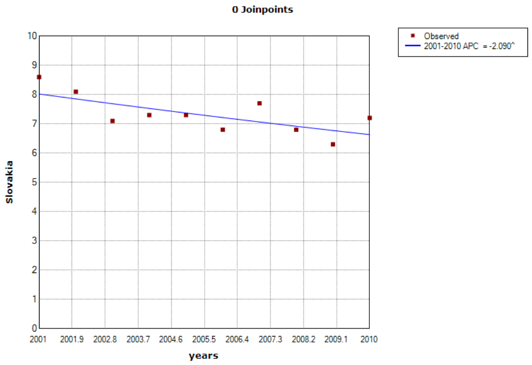

j.) mortality female aged from 0 to 85 years old

Asia

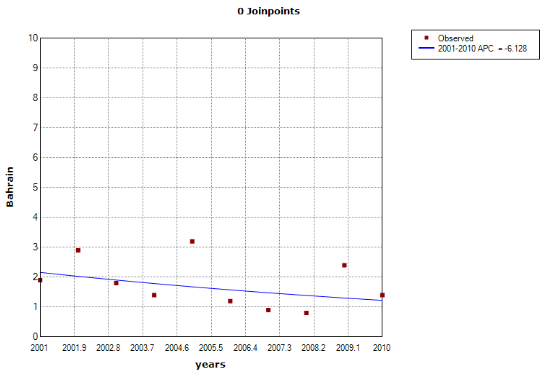

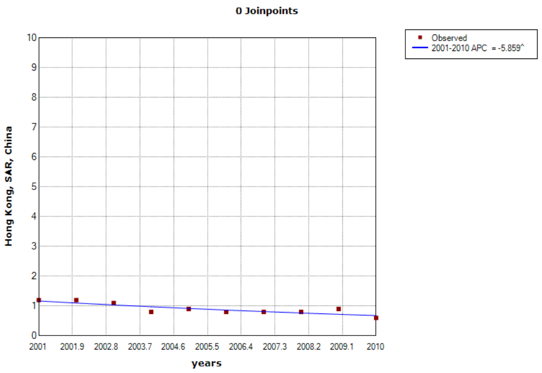

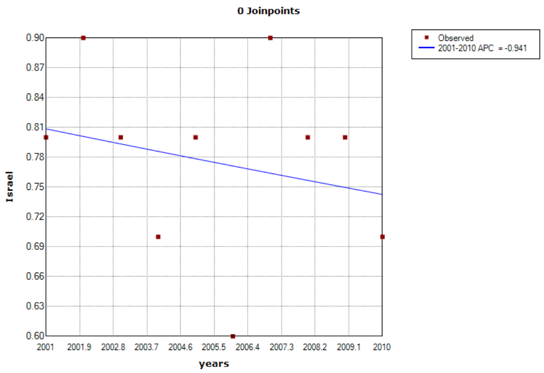

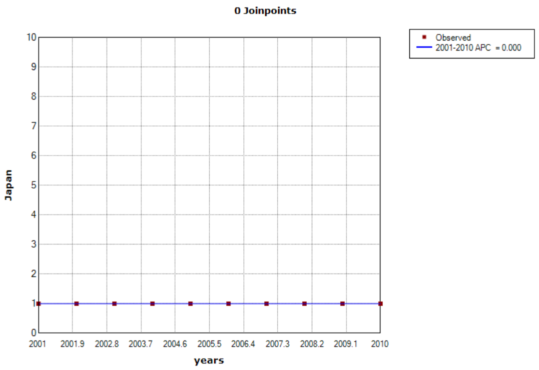

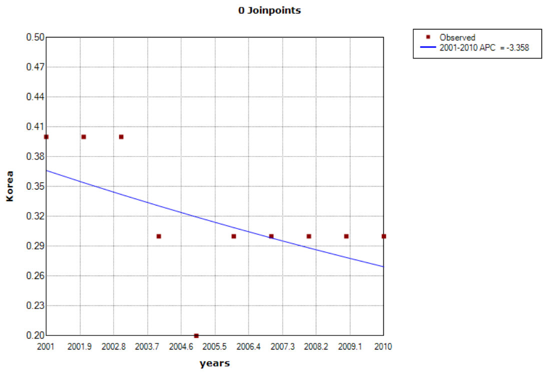

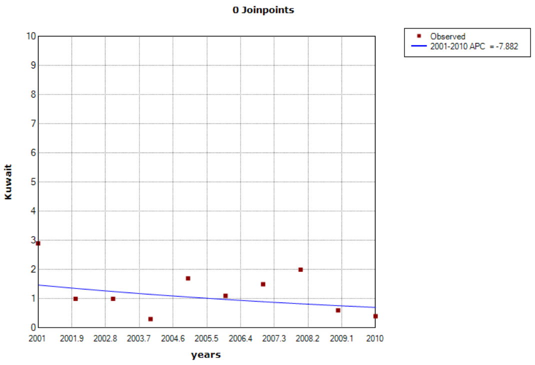

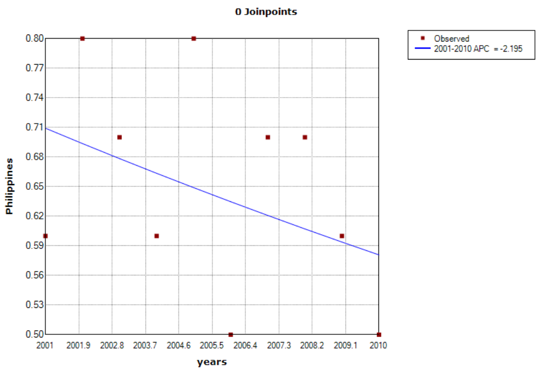
0 Joinpoints
Observed
2001-2010 APC = -2.195
Philippines
years

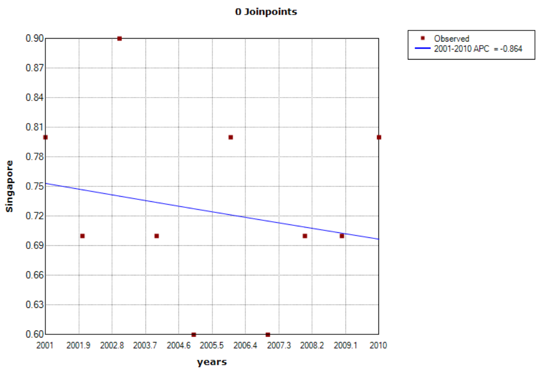
0 Joinpoints
Observed
2001-2010 APC = -0.864
Singapore
years

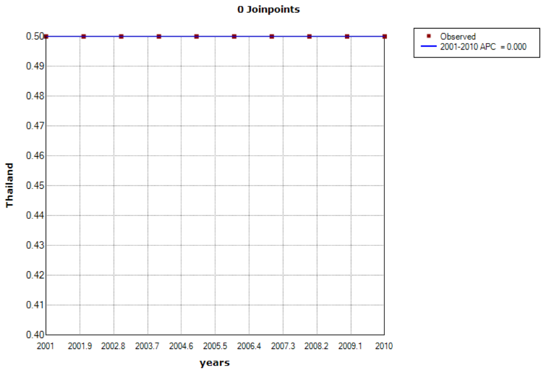
0 Joinpoints
Observed
2001-2010 APC = 0.000
Thailand
years

Oceania

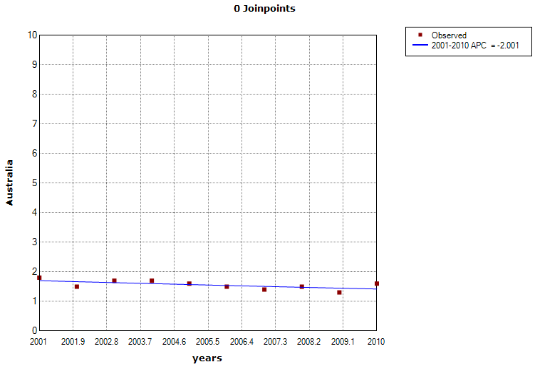
0 Joinpoints
Observed
2001-2010 APC = -2.001
Australia
years

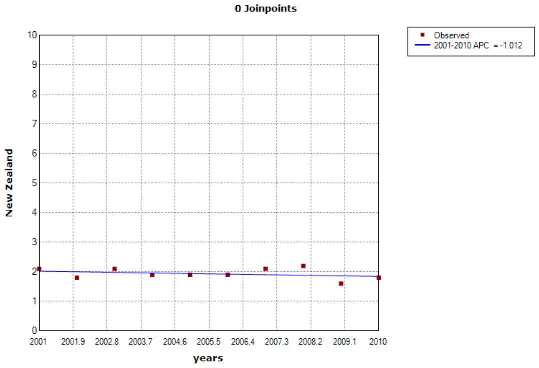
0 Joinpoints
Observed
2001-2010 APC = -1.012
New Zealand
years

Northern America

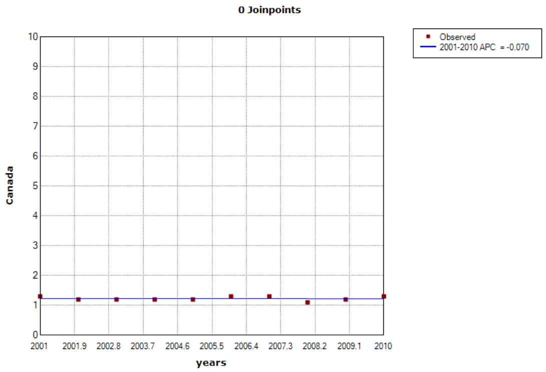

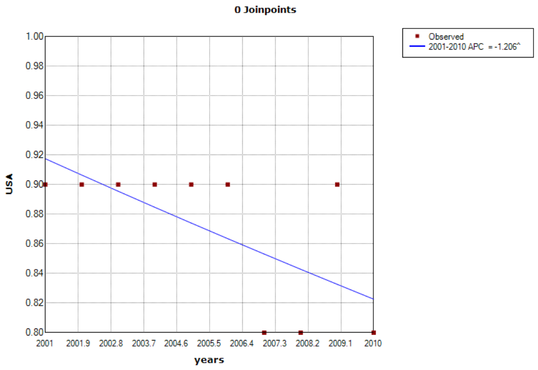

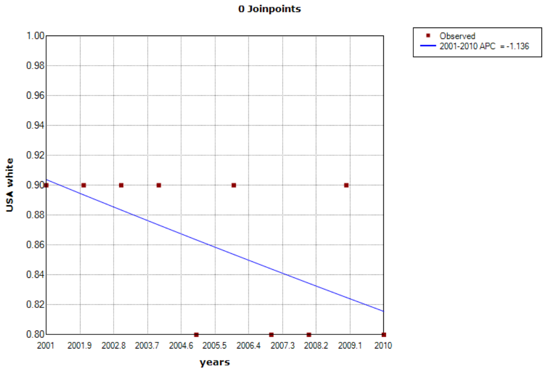

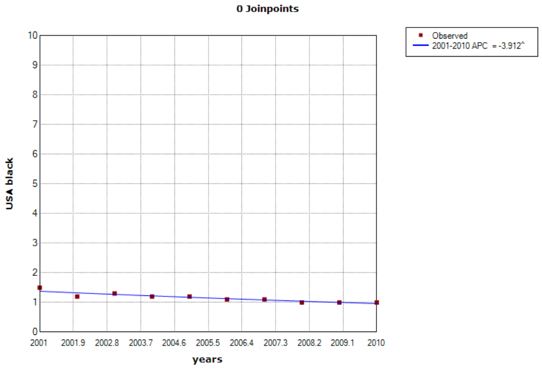

Southern America

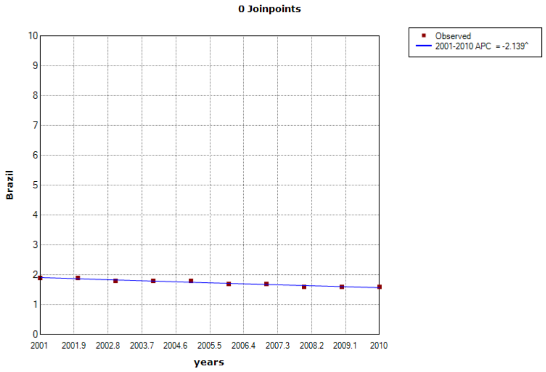

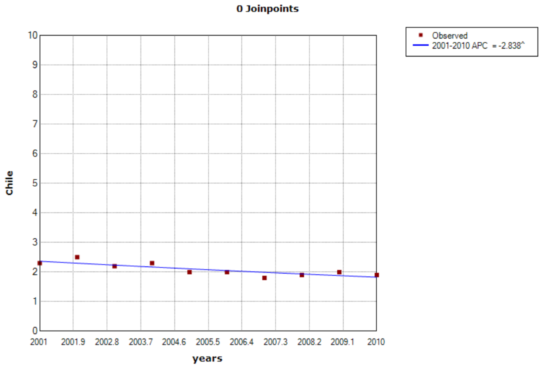

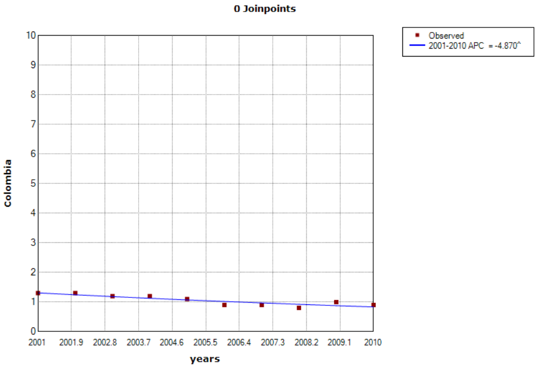

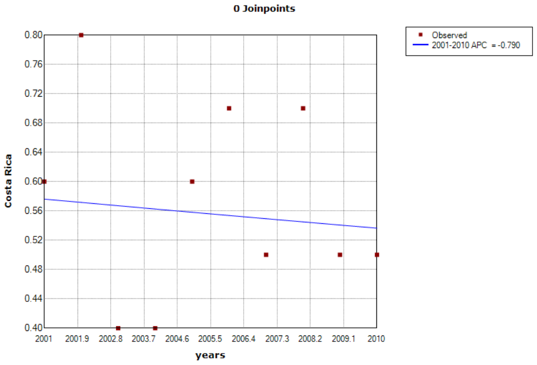

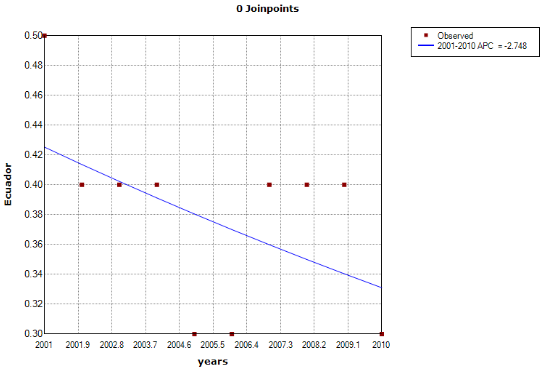

Northern Europe

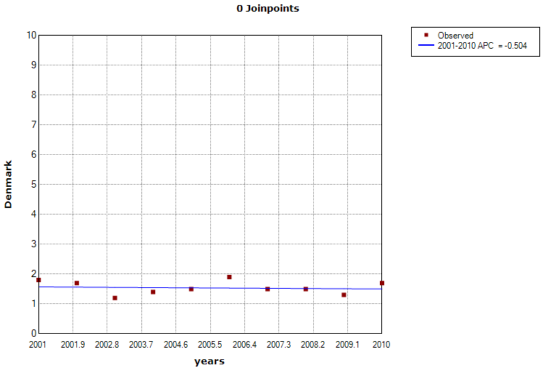

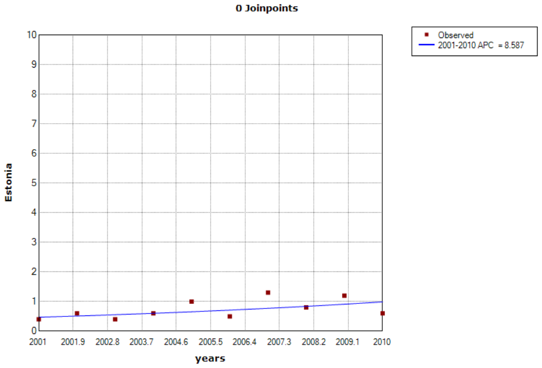

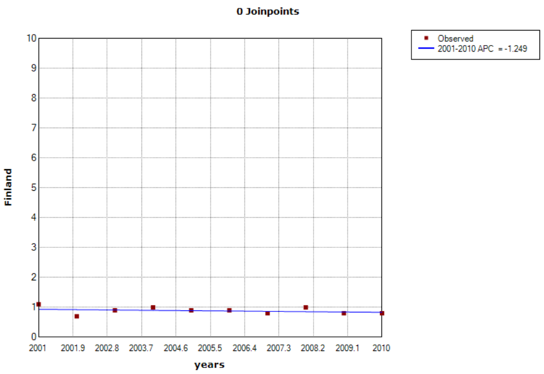
0 Joinpoints
Observed
2001-2010 APC = -1.249
Finland
years

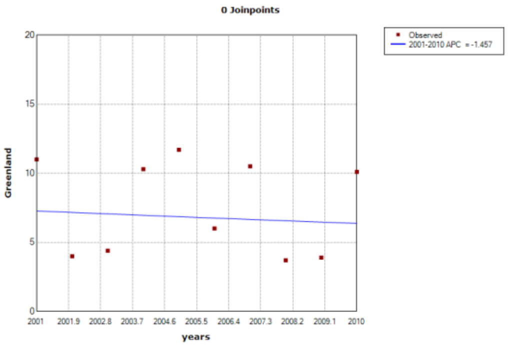
0 Joinpoints
Observed
2001-2010 APC = -1.457
Greenland
years

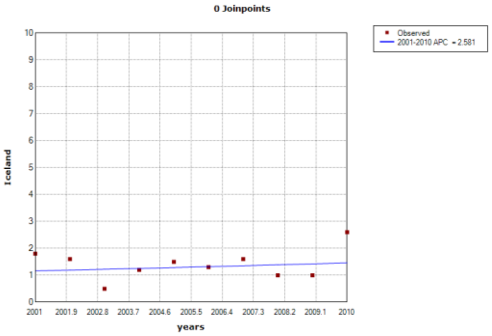
0 Joinpoints
Observed
2001-2010 APC = 2.581
Iceland
years

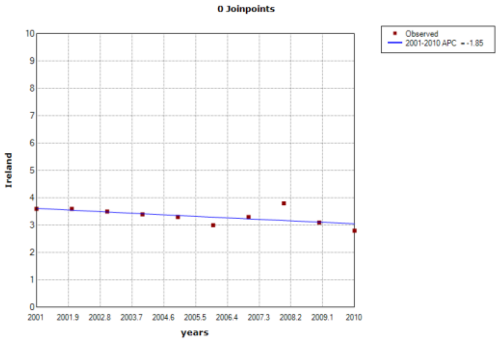
0 Joinpoints
Observed
2001-2010 APC = -1.85
Ireland
years

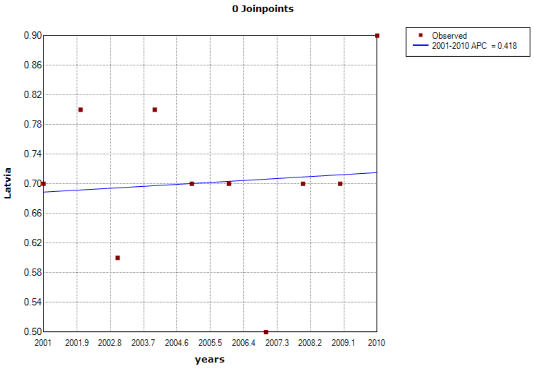
0 Joinpoints
Observed
2001-2010 APC = 0.418
Latvia
years

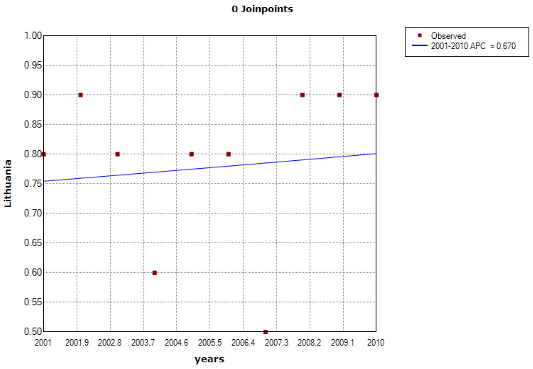
0 Joinpoints
Observed
2001-2010 APC = 0.670
Lithuania
years

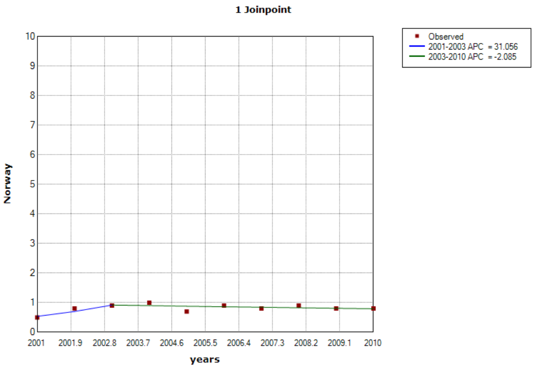
1 Joinpoint
Observed
2001-2003 APC = 31.056
2003-2010 APC = -2.085
Norway
years

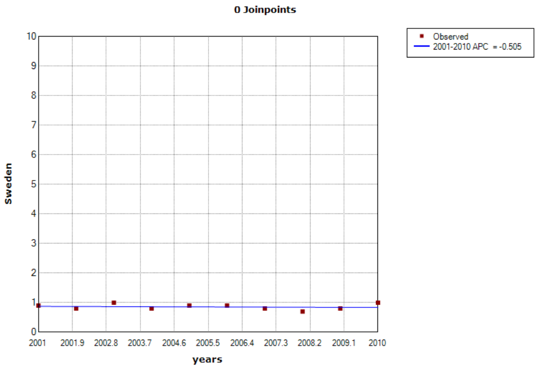
0 Joinpoints
Observed
2001-2010 APC = -0.505
Sweden
years

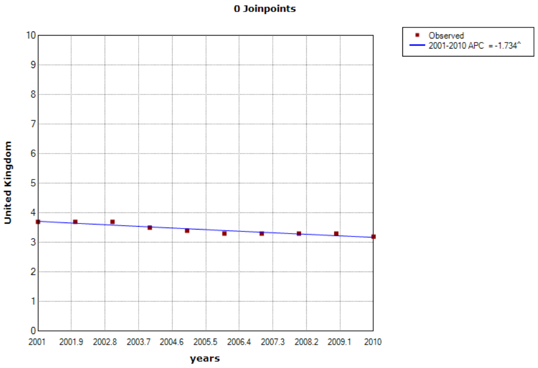

Western Europe

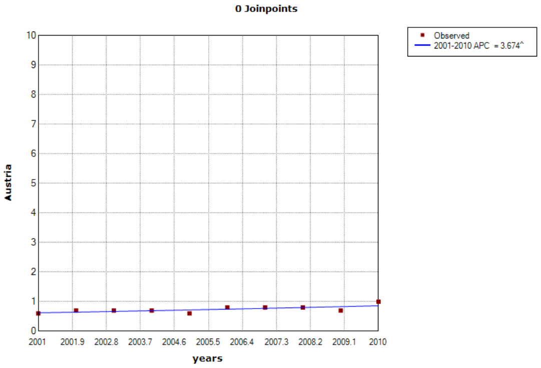

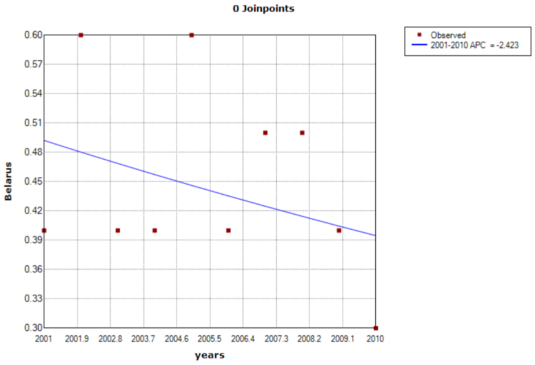

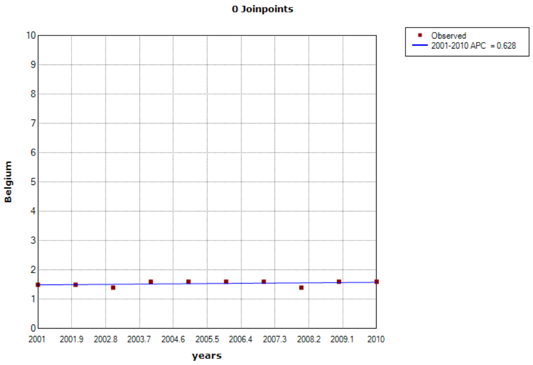

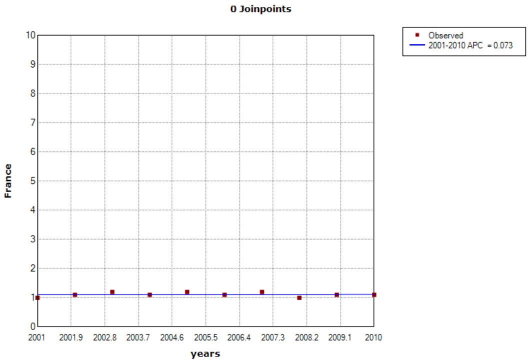

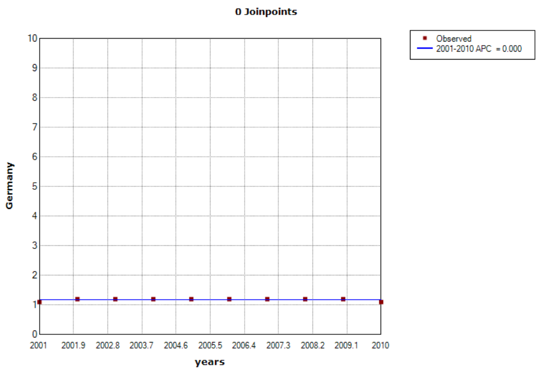

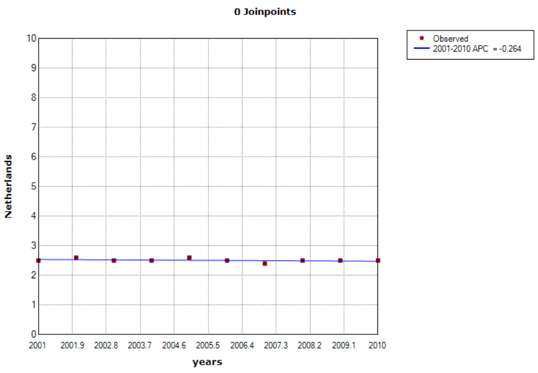

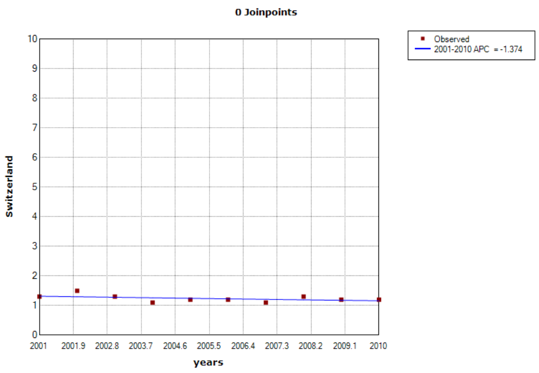

Southern Europe

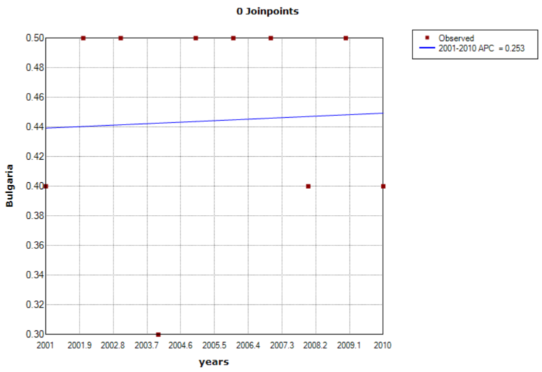

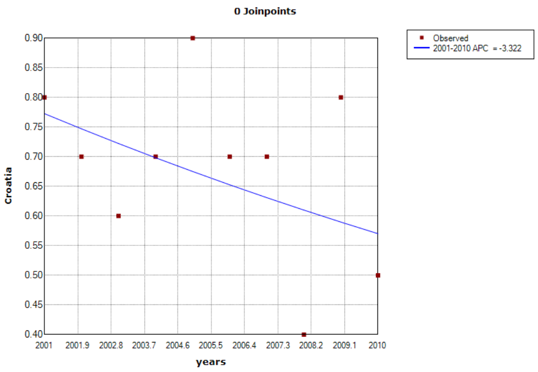

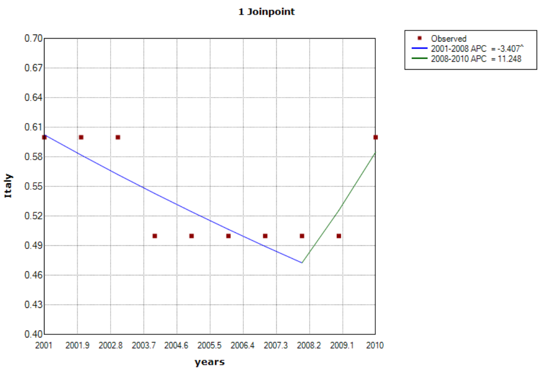
1 Joinpoint
Observed
2001-2008 APC = -3.407^
2008-2010 APC = 11.248
Italy
years

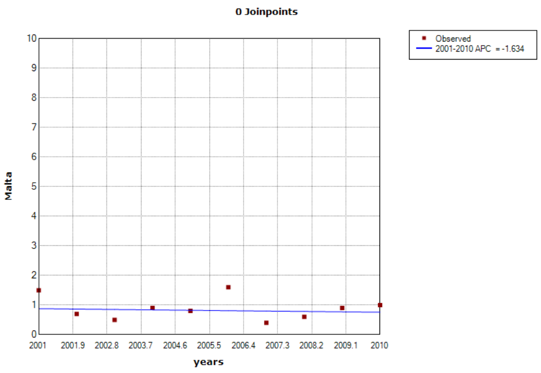
0 Joinpoints
Observed
2001-2010 APC = -1.634
Malta
years

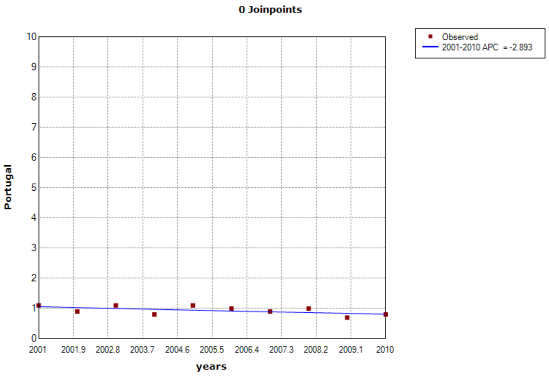
0 Joinpoints
Observed
2001-2010 APC = -2.893
Portugal
years

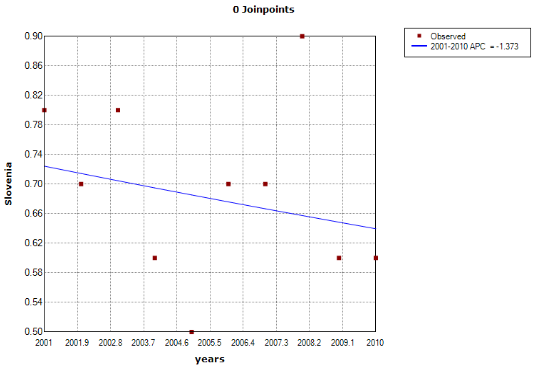
0 Joinpoints
Observed
2001-2010 APC = -1.373
Slovenia
years

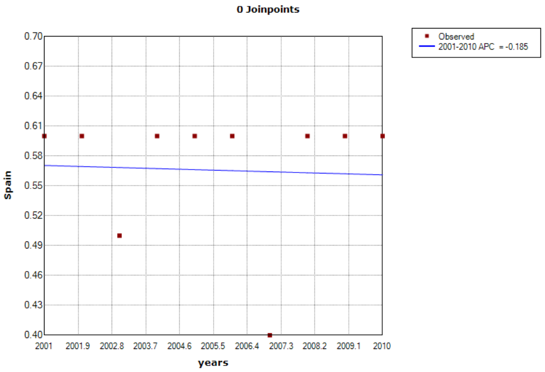
0 Joinpoints
Observed
2001-2010 APC = -0.185
Spain
years

Eastern Europe

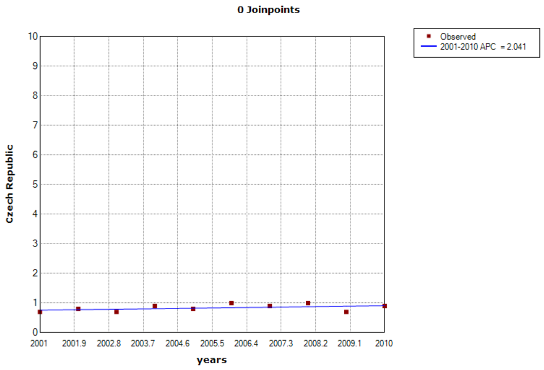

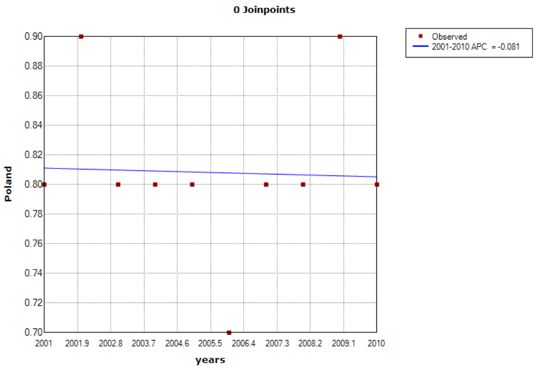

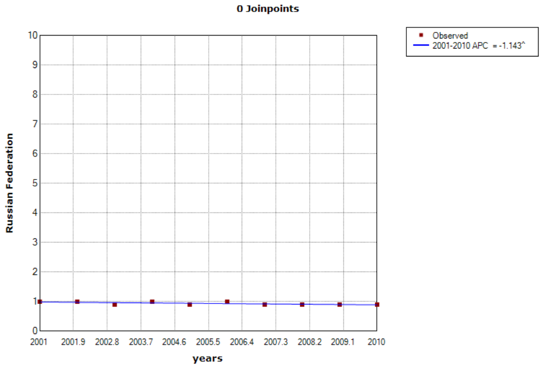

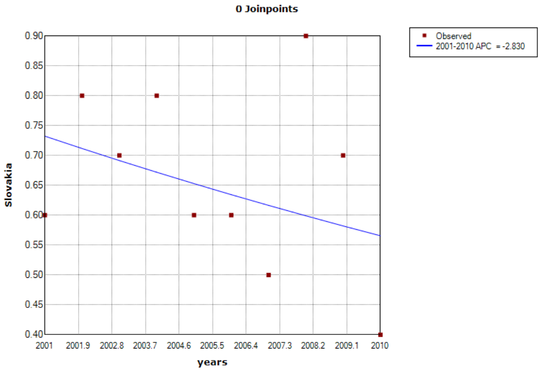

Figure S3. The AAPC of the incidence of esophageal cancer in individuals aged < 50 years

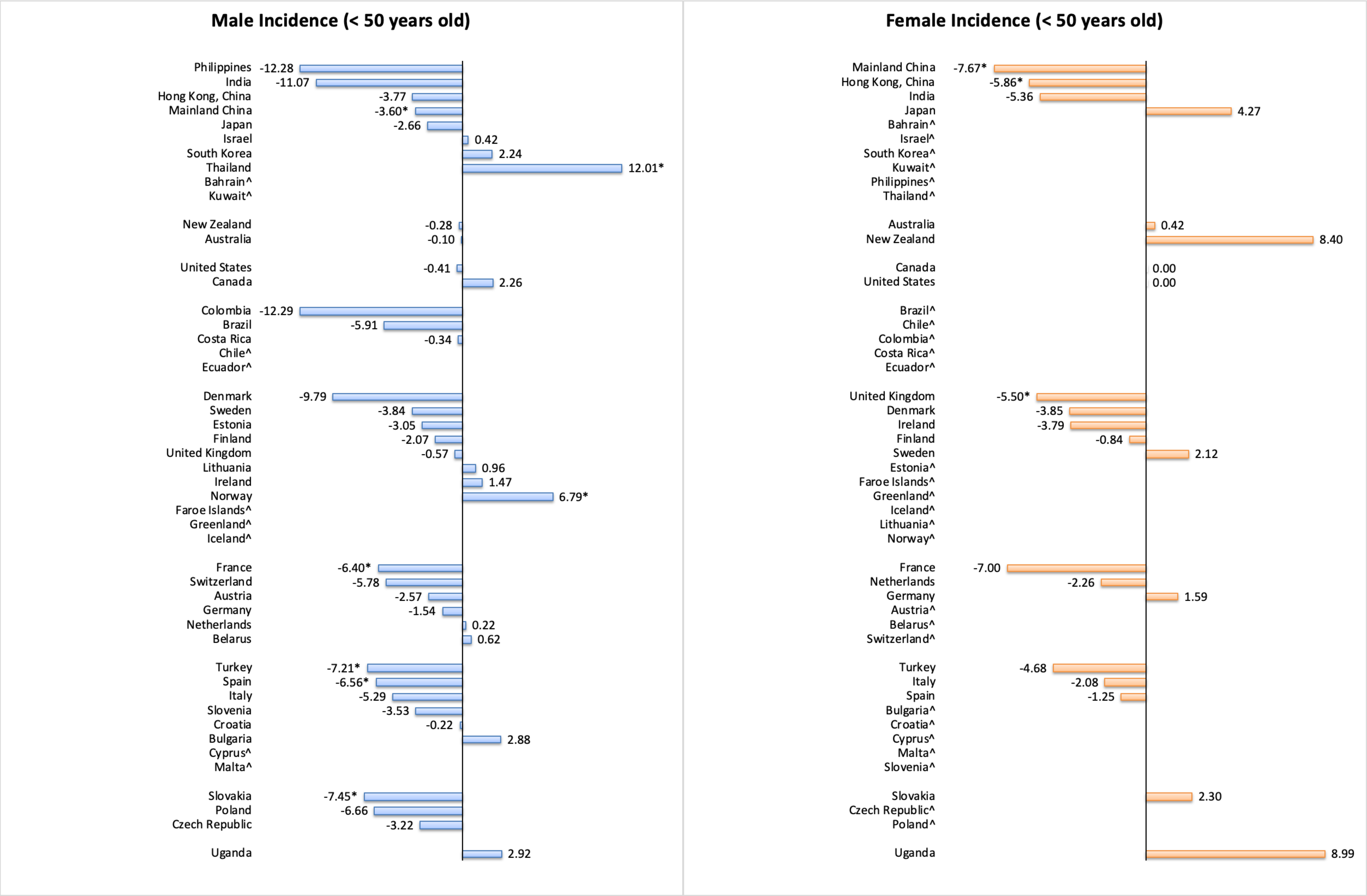

AAPC, annual percentage change; **p* values less than 0.05; ^AAPC for these countries could not be generated as zero or missing values were identified in any year of trend analysis; The 95% confidence intervals and *p* values for the tests of AAPC were presented in Table S5.
